# Rational Design of Persistent Phosphorus-Centered Singlet Tetraradicals and Their Use in Small-Molecule Activation

**DOI:** 10.1021/jacs.3c03928

**Published:** 2023-06-14

**Authors:** Edgar Zander, Jonas Bresien, Vladimir V. Zhivonitko, Johannes Fessler, Alexander Villinger, Dirk Michalik, Axel Schulz

**Affiliations:** † Institut für Chemie, Universität Rostock, Albert-Einstein-Straße 3a, 18059 Rostock, Germany; ‡ NMR Research Unit, University of Oulu, P.O. Box 3000, 90014, Oulu, Finland; § Leibniz-Institut für Katalyse e.V., Albert-Einstein-Straße 29a, 18059 Rostock, Germany

## Abstract

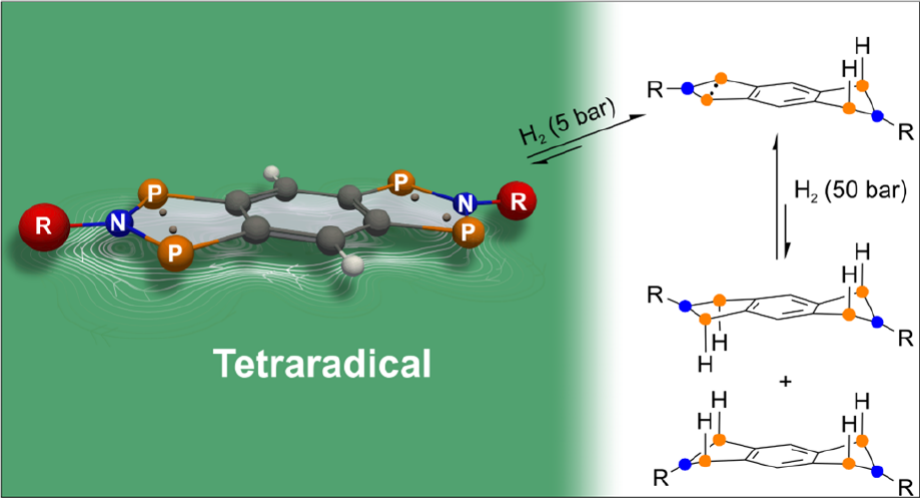

Biradicals are important intermediates in the process of bond formation and breaking. While main-group-element-centered biradicals have been thoroughly studied, much less is known about tetraradicals, as their very low stability has hampered their isolation and use in small-molecule activation. Herein, we describe the search for persistent phosphorus-centered tetraradicals. Starting from an *s*-hydrindacenyl skeleton, we investigated the introduction of four phosphorus-based radical sites linked by an N–R unit and bridged by a benzene moiety. By varying the size of the substituent R, we finally succeeded in isolating a persistent P-centered singlet tetraradical, 2,6-diaza-1,3,5,7-tetraphospha-*s*-hydrindacene-1,3,5,7-tetrayl (**1**), in good yields. Furthermore, it was demonstrated that tetraradical **1** can be utilized for the activation of small molecules such as molecular hydrogen or alkynes. In addition to the synthesis of P-centered tetraradicals, the comparison with other known tetraradicals as well as biradicals is described on the basis of quantum mechanical calculations with respect to its multireference character, coupling of radical electrons, and aromaticity. The strong coupling of radical electrons enables selective discrimination between the first and the second activations of small molecules, which is shown by the example of H_2_ addition. The mechanism of hydrogen addition is investigated with parahydrogen-induced hyperpolarization NMR studies and DFT calculations.

## Introduction

**Figure 1:**
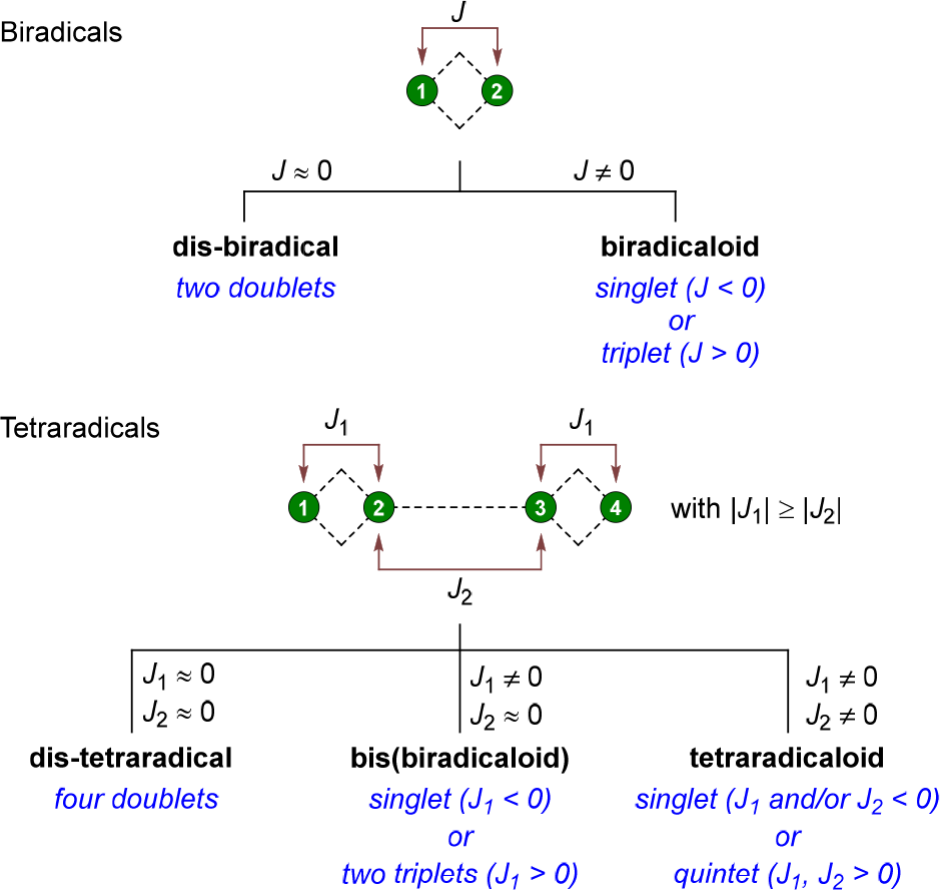
Classification of symmetrical bi-^6^ and tetraradicals^8^ by means of electron exchange coupling constants *J* (antiferromagnetic coupling: negative *J*; ferromagnetic coupling: positive *J*). A green circle corresponds to an atom with one radical electron.

Biradicals are molecules with two radical electrons in two nearly degenerate orbitals.^1−4^ A classification of biradicals is possible, for example, on the basis of the electron exchange coupling constant (*J*),^5^ which describes the interaction between the two radical electrons (Figure 1, top). Molecular systems, in which the radical electrons do not interact with each other (*J* = 0), are called dis-biradicals and are in spectroscopic terms two-doublet species.^6^ When the electrons do interact with each other (*J* ≠ 0), a biradicaloid is formed where the electrons couple either antiferromagnetically (singlet species, *J* < 0) or ferromagnetically (triplet species *J* > 0).^6,7^ However, there is no exact value for *J*, which separates biradicaloids from dis-biradicals and closed-shell molecules. For simplicity, the term biradical is used in this article, and unless otherwise stated, it refers to biradicaloids. The same applies to the term tetraradical referring to tetraradicaloids.

Extending the biradical concept, tetraradicals are molecules with four radical electrons in four nearly degenerate orbitals. Here, the interaction between the four radical electrons can be completely described by six electron exchange coupling constants. To simplify such systems, the discussion is restricted here to symmetrical tetraradicals, in which two biradicals are connected by a linker and in which the interactions can be described by only two electron exchange coupling constants (Figure 1, *J*_1_ = coupling within a biradical unit, *J*_2_ = coupling between the two biradical units, where |*J*_1_| ≥ |*J*_2_|).

With these restrictions, three different types of tetraradicals can be distinguished by considering the coupling constants (Figure 1, bottom): (a) Dis-tetraradicals, in which no interaction between the electrons occurs at all (*J*_1_ = *J*_2_ = 0, four-doublet species); (b) bis(biradicaloids), in which there is no interaction between the two sets of biradicaloids (*J*_2_ = 0), and depending on the coupling within the biradical fragments either a singlet (*J*_1_ < 0) or a two-triplet species (*J*_2_ > 0) can be present; and (c) tetraradicaloids, in which there is an interaction between the electrons of both biradical units and the species either adopts a singlet (*J*_1_ and/or *J*_2_ < 0) or quintet state (*J*_1_ and *J*_2_ > 0).

Stable cyclic biradicals have been in the focus of preparative chemistry^7,9−12^ since the synthesis of the first stable heterocyclobutene-1,3-diyl by Niecke and co-workers in 1995.^13^ Their unique reactivity with respect to bond activation is in between radicals and closed-shell molecules.^14^ For example, the activation of small molecules such as dihydrogen,^15−20^ chalcogens,^21−23^ halogenated alkanes,^24−28^ and molecules with double and triple bonds (e.g., CO,^23^ HCCH^28,29^) by cyclic main-group-element-centered biradicals has been demonstrated in many studies.^5,7,10^

**Scheme 1:**
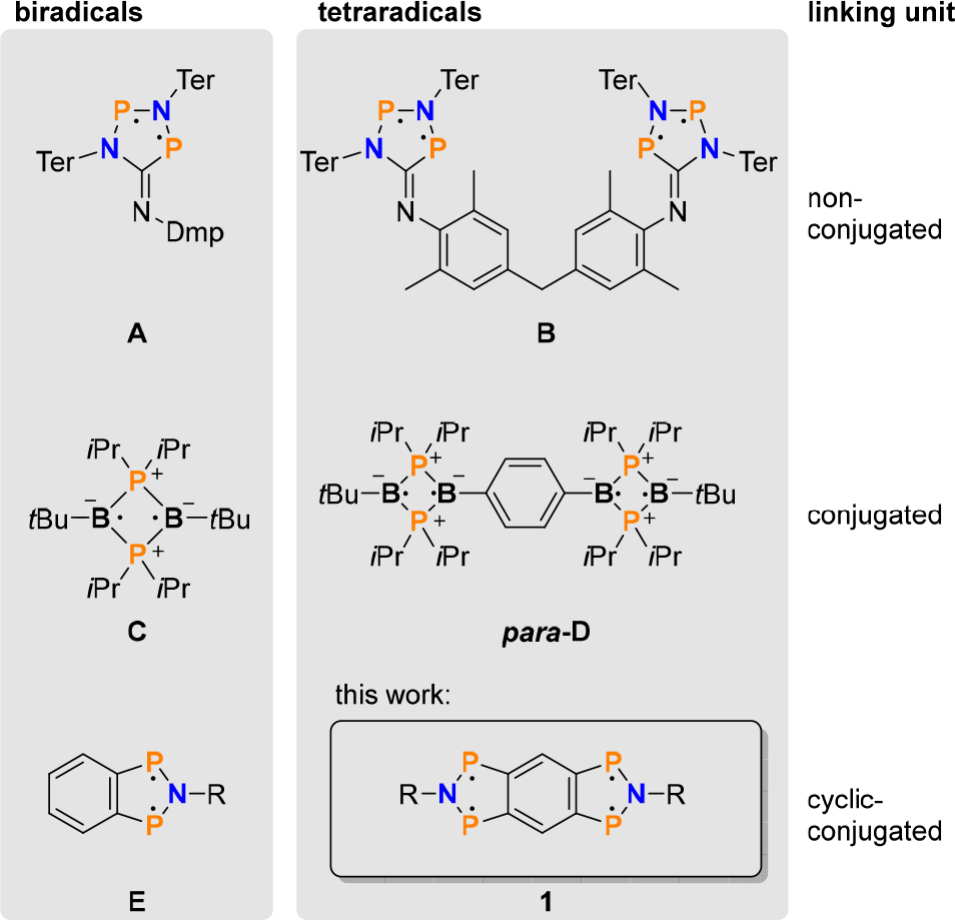
Known Main-Group-Element-Centered Tetraradicals (**B**,^28^*para***-D**^32^), Synthesized by Linking Biradical Structural Motifs (**A**,^28^**C**,^30^ and **E**(43))

Heteroatom-centered cyclic tetraradicals, on the other hand, have been the subject of only a few publications so far (Scheme 1).^28,30,31^ Conceptually, tetraradicals can be constructed by linking two biradicals (vide supra), as illustrated in Scheme 1. For example, our group was able to synthesize the tetraradical **B** by bridging two P-centered heterocyclopentane-1,3-diyls **A** with a methylenediphenyl linker.^28^ Since the non-conjugated linker does not allow any interaction between the two biradical units, it has to be considered as a bis(biradicaloid), showing the same reactivity and properties as the single, uncatenated biradicaloid **A**. Bertrand and co-workers were able to link two boron-centered heterocyclobutane-1,3-diyls^30^**C** with a *para*- and *meta*-substituted benzene (Scheme 1). Interestingly, *para*-substitution results in the formation of the tetraradical *para-***D**, while the *meta*-substituted isomer, *meta***-D**, forms a closed-shell species with transannular boron–boron bonds at ambient temperatures.^32^ It should be noted that also tetraradical *para***-D** forms a closed-shell butterfly species with two boron–boron bonds at slightly elevated temperatures. DFT calculations of *meta***-** and *para***-D** showed that both biradical units interact significantly via the conjugated linker. The interaction in *meta***-D** is smaller than in *para***-D**, which was given as a possible reason for the exclusive observation of the closed-shell species *meta***-D**.^8^ Besides main-group-element-centered tetraradicals **B** and *para***-D**, some further—however less related—compounds like cluster-^31^ or organic aminoxyl-^33−36^ and aminyl-based^37^ as well as C-centered^31,38−42^ tetraradicals are known.

We were intrigued by the idea to extend the structural motif of the recently published azadiphosphaindane-1,3-diyl (**E**, Scheme 1)^43^ to the tetraradical **1**, in which all radical electrons are part of a condensed, aromatic 14π electron ring system. Two questions were of interest in the synthesis: First, how bulky must the steric hindrance be to prevent dimerization or oligomerization of the tetraradical, because this would lead to closed-shell systems? And second, is it possible to use these tetraradicals for the stepwise activation of small molecules? Through the interaction between the radical sites, when using the benzene linker, we hoped to achieve a kinetic separation between the addition of a first and second equivalent of a small molecule to the formal biradical units in **1**. For bis(biradicaloids), no kinetic separation between the two activation steps is expected due to the missing interaction and large spatial separation, so that both biradical subunits react independently.

## Results and Discussion

### Synthesis

**Scheme 2:**
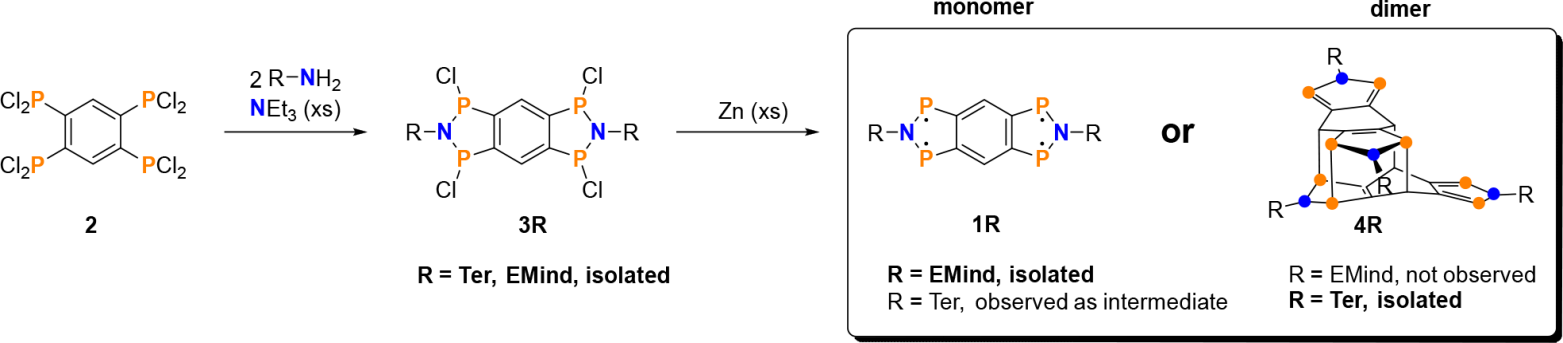
Synthesis of Differently Substituted 2,6-Diaza-1,3,5,7-tetraphospha-*s*-hydrindacene-1,3,5,7-tetrayls (**1R**) Their stability toward oligomerization depends on the steric demand of the substituent R (= Ter, EMind), as depicted on the right.

To form a type **1** tetraradical, several aspects must be considered in the design. First, it needs sufficiently large steric protection, which can be tuned via the substituent at the nitrogen atom. Second, it needs suitable precursors for the construction of two PNP units connected to the central benzene ring. To this end, we identified 1,2,4,5-tetrakis(dichlorophosphino)benzene **2** (Scheme 2) as suitable starting material. Tetraphosphane **2** was recently published by our group and is easily prepared in very good yield (91%) by complete chlorination of the corresponding tetraphosphane with PCl_5_.^44^

### Synthesis of Starting Materials

With tetrakis(dichlorophosphino)benzene **2** in hand, double ring closure on both sides of the benzene ring was attempted by reaction with two equivalents of a primary amine, H_2_N-R, in a dehydrochlorination reaction (Scheme 2; R = Ter, ^*t*Bu^Bhp, Mes*, and EMind; Ter = 2,6-dimesitylphenyl,^45,46^^*t*Bu^Bhp = 2,6-bis(benzhydryl)-4-*tert*-butylphenyl,^43,47^ Mes* = 2,4,6-tri-*tert*-butylphenyl,^48^ and EMind = 1,1,7,7-tetraethyl-3,3,5,5-tetramethyl-*s*-hydrindacenyl).^49−51^ In the case of the Ter and EMind substituents, this reaction led to the formation of the tricyclic, 4-fold chlorinated ring system **3R** in good yields (**3Ter**: 62%, **3EMind**: 78%), whereas the Mes* and ^*t*Bu^Bhp derivatives were only formed in low yields and/or could not be isolated in acceptable purity (cf. Supporting Information (SI), p S44 ff.).

**Figure 2:**
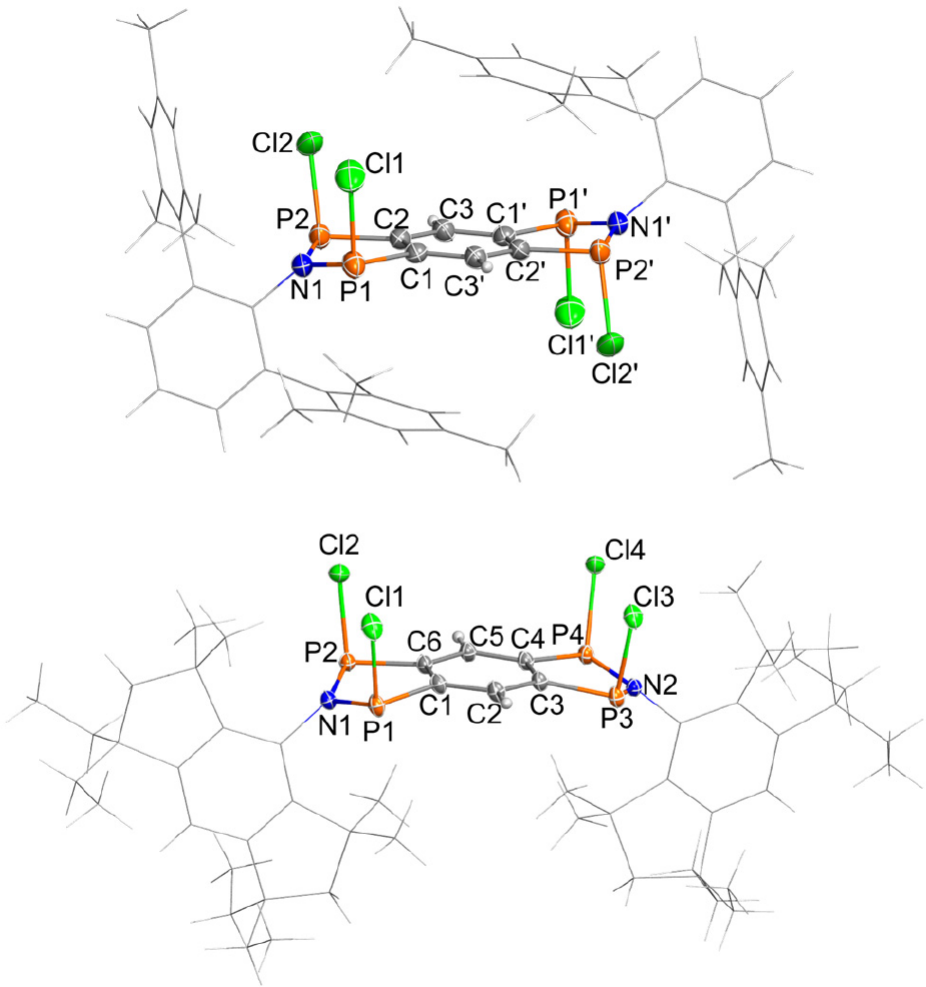
Molecular structures of **3Ter** (top) and **3EMind** (bottom) in the crystal (*T* = 203 K, ellipsoids at 50% probability). Selected bond lengths [Å] and angles [deg]: **3Ter**: P1–Cl1 2.102(1), P1–P2 2.964(1), P1–C1 1.821(2), P2–Cl2 2.084(1), P2–C2 1.821(2), N1–P1–P2–C2–160.6(2), symmetry code: (′) = 1–*x*, 1–*y*, 1–*z*; **3EMind**: P4–Cl4 2.0981(8), P3–Cl3 2.1089(7), P2–Cl2 2.1066(7), P1–Cl1 2.0954(7), P4–C4 1.828(2), P3–C3 1.822(2), P2–C6 1.820(2), P1–C1 1.831(2), P3–P4 2.9733(7), P1–P2 2.9642(7), P1–N1–P2 119.57(7), P3–N2–P4 120.32(7), N1–P1–P2–C6–159.4(1), C3–P3–P4–N2–164.3(1).

Both **3Ter** and **3EMind** are thermally very stable up to about 360–380 °C. They dissolve well in CH_2_Cl_2_, from which colorless single crystals were obtained (Figure 2). Interestingly, for **3Ter**, the *trans* isomer is found (the two chlorine atoms on each side are *trans* to the two on the other side), while for **3EMind** all chlorine atoms are on one side of the three condensed rings, resulting in the formation of a *cis* isomer in the crystal. In both compounds, the central benzene ring remains planar, while the two outer five-membered rings are notably puckered (envelope conformation). This effect is much more pronounced in **3EMind**. The mean P–C (**3Ter**: 1.821(2), **3EMind**: 1.825(3) Å) and P–N bonds (**3Ter**: 1.706(3), **3EMind**: 1.715(4) Å) are in the typical range of polarized single bonds (cf. ∑*r*_cov._(C–P) = 1.86 Å, ∑*r*_cov._(N–P) = 1.82 Å).^52^ As expected, the ^31^P NMR spectra showed a singlet signal at 143 (**3Ter**) and 145 ppm (**3EMind**), respectively.

### Attempted Synthesis of **1Ter**: Isolation of the Dimer **4Ter**

**Figure 3:**
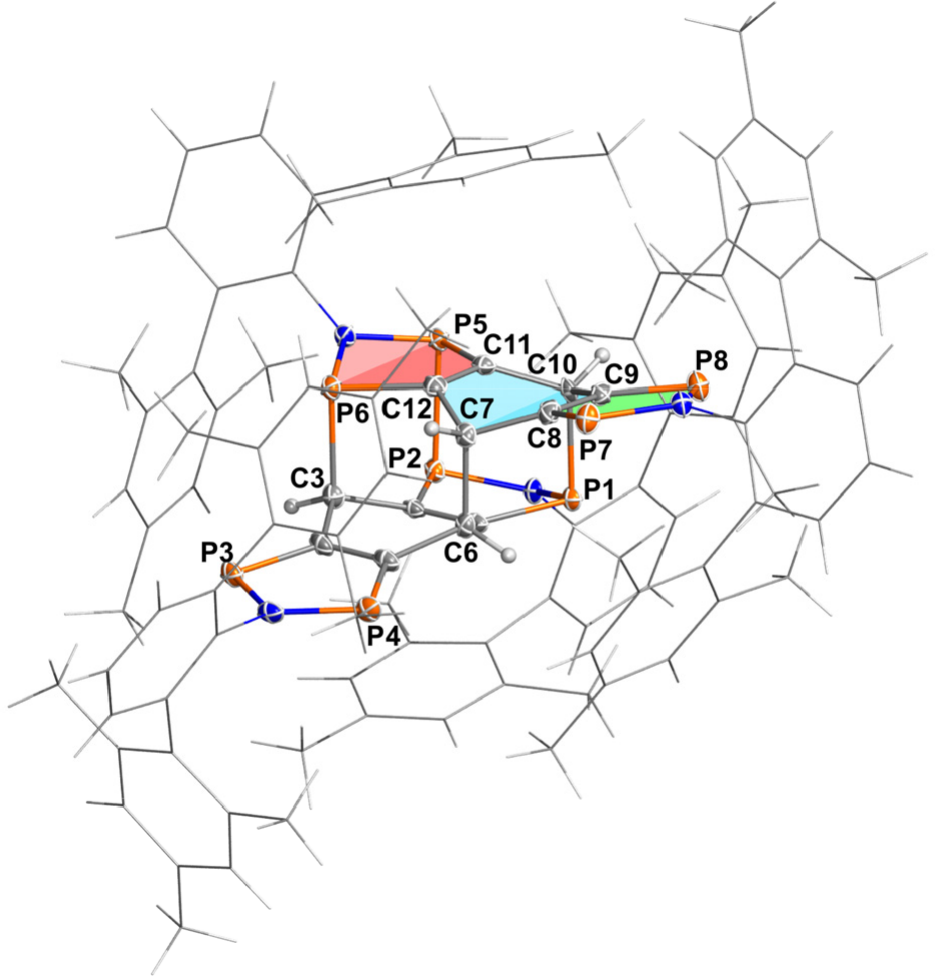
Molecular structure of **4Ter** in the crystal. Ellipsoids are set at 50% probability (123 K). Selected distances [Å]: P3–P4 2.943(1), P5–P6 2.928(1), P1–C10 1.945(3), P2–P5 2.3364(9), P7–C8 1.703(3), P8–C9 1.714(3), P5–C11 1.802(3), P6–C12 1.817(3), P6–C3 1.942(3), C6–C7 1.625(3).

The reduction of **3Ter** with elemental zinc dust in tetrahydrofuran (THF) did not lead to the desired tetraradical **1Ter** but selectively to a dimer (**4Ter**, Scheme 2) as unequivocally proven by single-crystal X-ray diffraction (SCXRD) (Figure 3).

Interestingly, the reaction mixture turned green at the start, the color of the targeted tetraradical (see below), but then slowly changed to brown. Yellow single crystals of **4Ter** could be isolated from this brown solution. It was not possible to isolate the green intermediate, which is probably **1Ter**, but it could be trapped by derivatization (vide infra). Dimer **4Ter** decomposes above 180 °C in the solid state and is long-term stable in solution at ambient temperatures, as shown by NMR experiments. In the ^31^P{^1^H} NMR spectrum, **4Ter** features an AA′BB′XX′YY′ spin system due to its *C*_2_ symmetry (see Figure S19). However, the NMR spectrum is simplified by the fact that there is only one larger coupling constant (^3^*J*(P3,P6) = ^3^*J*(P1,P8) = 25 Hz, assignment as depicted in Figure 3). The divalent P atoms (δ(P4/P7) = 273 ppm; δ(P3/P8) = 280 ppm) are significantly deshielded in comparison to the trivalent P atoms (δ(P2/P5) = 120 ppm; δ(P1/P6) = 136 ppm), and their chemical shifts are similar to those of structurally related compounds (cf. **E**: 285 ppm in Scheme 1).^43^ By means of temperature-dependent ^31^P{^1^H} NMR measurements we investigated whether **4Ter** can dissociate into its tetraradical monomers (**1Ter**). Above 100 °C, decomposition of **4Ter** occurred, but formation of the monomeric **1Ter** was not observed (see Figure S20).

Yellow crystals of **4Ter** crystallized in the triclinic space group *P*1̅ with one molecule and three cocrystallized solvent molecules in the unit cell. As depicted in Figure 3, dimer **4Ter** is generated by the formation of one P–P, two C–P, and one C–C single bond between the two monomeric units, the desired tetraradical **1Ter**, thus forming a cage compound in a very unusual addition reaction. In cage compound **4Ter**, two ethylene units are oriented orthogonally to each other and linked at four points via chains of two atoms. The parent hydrocarbon compound (tricyclo[5.5.0.0^4,10^]dodeca-1(7),4(10)-diene) corresponding to this structural motif has not been isolated so far.^53^ The bonds between the atoms connecting the monomeric units (*d*(P2–P5) = 2.3364(9) Å, *d*(P1–C10) = 1.945(3) Å, *d*(P6–C3) = 1.942(3), *d*(C6–C7) = 1.625(3) Å) are all elongated by approximately 0.1 Å, compared to the sum of the covalent radii for the corresponding single bonds (∑*r*_cov._(P–P) = 2.22 Å, ∑*r*_cov._(C–P) = 1.86 Å, ∑*r*_cov._(C–C) = 1.50 Å).^52^ In addition, dimerization also removes the planarity of the monomer, since the three condensed rings are no longer aromatic (cf. planar structure of **1EMind**, see below). Therefore, all rings involved in the dimerization (red and blue rings in Figure 3) are significantly puckered (red: 25.3(2)°, along P6···P5; blue: 37.2(2)°, along C7···C10), while the third ring remains nearly planar (green: 2.4(3)°, along P5···P8). The C–P bond lengths in this planar five-membered ring (*d*(C–P) = 1.711(3) Å) indicate double bonds (∑*r*_cov._(P=C) = 1.69 Å).^52^ This new bonding situation that arises during dimerization is shown in Scheme 2. It is worthy to note that a comparable type of trimerization was previously observed in the attempted synthesis of the Ter derivative of biradical **E**.^43^ Thus, the steric demand of the Ter substituent is apparently not sufficient to stabilize these bi/tetraradical structures.

### Trapping of Tetraradical **1Ter**

**Scheme 3:**
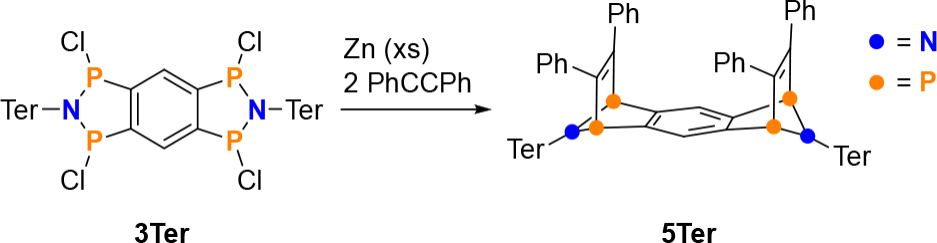
Trapping of Tetraradical **1Ter** by Addition of Tolan

**Figure 4:**
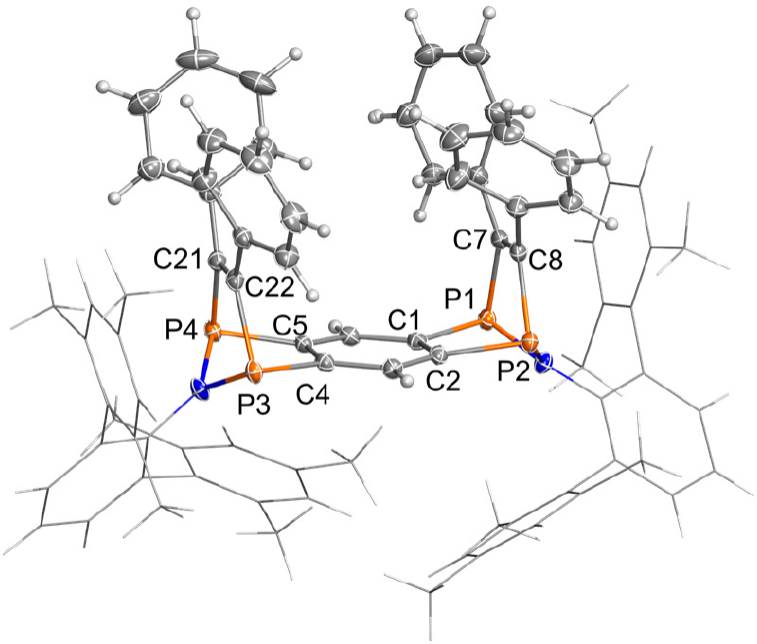
Molecular structure of **5Ter** in the crystal. Ellipsoids are set at 50% probability (123 K). Selected distances [Å] and angles [deg]: P1–C1 1.857(2), P1–C7 1.885(2), P2–C2 1.845(2), P1–P2 2.7877(7), P2–C8 1.879(2), P3–C4 1.865(2), P3–C22 1.878(2), C7–C8 1.344(3), C22–C21 1.347(3), P2–C8–C7 114.0(2), P3–C22–C21 111.4(1).

Since we observed the *in situ* formation of tetraradical **1Ter** at the beginning of the reaction (green color, ^31^P NMR shift at 287 ppm), we investigated the possibility of trapping it by adding an alkyne during the reduction process of **3Ter** with zinc dust. In previous studies, we have shown that cyclic four- and five-membered P-centered biradicals can readily add alkynes such as tolan.^6,7,22^ For example, it was demonstrated that tolan is a suitable trapping reagent for unstable biradicals of the type [^•^E(μ-NTer)]_2_ (E = Sb, Bi),^54^ bridging two radical centers in a formal [2+2] addition. Indeed, *in situ* generated **1Ter** is also capable of activating tolan. When zinc is added to the chlorinated species **3Ter** (Scheme 3), a new compound is slowly formed as observed by ^31^P NMR experiments (δ(^31^P) = 100 (s)), which, after recrystallization from THF, leads to the deposition of colorless crystals. SCXRD revealed the formation of the double addition product **5Ter** (Figure 4). Although evidence (green color, ^31^P NMR data, vide infra) for in situ generation of **1Ter** is available, we do want to stress that the synthesis of **5Ter** is not direct proof of the presence of **1Ter**, as it is also conceivable that the reduction of the P–Cl units proceeds stepwise, forming only a biradical in the first step, followed by direct addition of tolan.

The tolan addition product **5Ter** is thermally stable up to over 360 °C and then decomposes. No reversible elimination of tolan was observed. The molecular structure of **5Ter** in the crystal proves that the two outer five-membered rings are strongly bent upon addition of tolan and incorporate only single bonds (Figure 4). The central six-membered ring remains planar and the former C–C triple bonds of the two added tolan molecules are now in the region of double bonds (C7–C8: 1.344(3), C22–C21: 1.347(3), cf. ∑*r*_cov._(C=C) = 1.34 and ∑*r*_cov._(C≡C) = 1.2 Å).^52^

### Synthesis of Tetraradical **1EMind**

**Figure 5:**
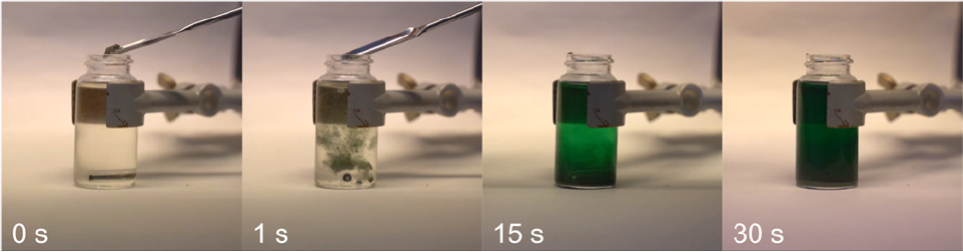
Addition of elemental zinc powder to a solution of **3EMind** leads to the formation of the intensively green colored tetraradical **1EMind.** The reduction was carried out in the glovebox in an argon atmosphere.

The synthesis of the tetraradical **1EMind** was achieved by reduction of **3EMind** with elemental zinc dust in THF at ambient temperatures in a rather slow reaction. Upon addition of the reducing agent, the colorless solution of **3EMind** immediately turned green (Figure 5).

However, complete conversion was detected only after 6 days according to ^31^P NMR studies. The course of the reduction process could easily be traced by ^31^P NMR spectroscopy, where the singlet signal of **3EMind** slowly disappeared at 145 ppm, while a new singlet signal at 289 ppm appeared and became more intense. Crystallization of **1EMind** from benzene after separation of ZnCl_2_ yielded remarkably temperature stable (*T*_dec._ = 365 °C) green crystals in gram scale in good yields (η = 67%). The UV–vis spectrum of **1EMind** showed two absorption bands in the visible region (λ_max_ = 396 and 667 nm), which explain the green color (Figure 5). In the ^31^P{^1^H} NMR spectrum, **1EMind** shows a single sharp resonance at 289 ppm (cf. biradical **E**: 285 ppm)^43^ and is EPR silent, suggesting a singlet ground state (see Electronic Structure section).

**Figure 6:**
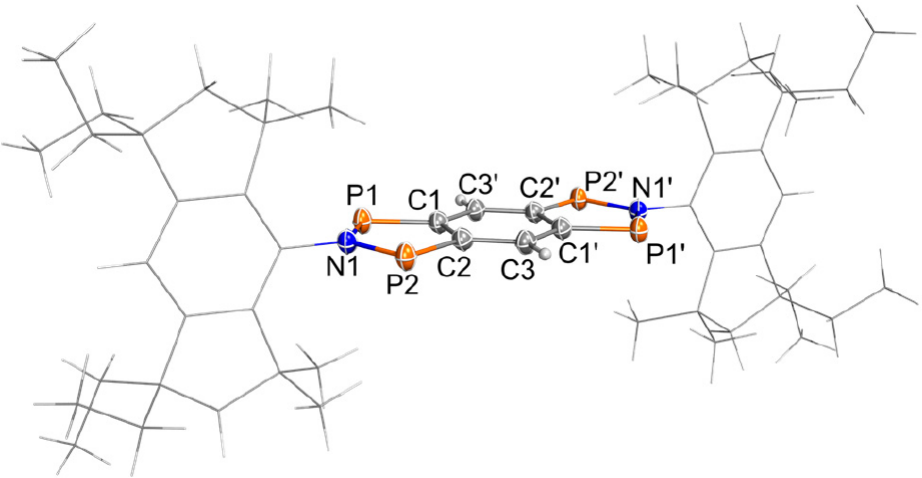
Molecular structure of **1EMind** in the crystal. Ellipsoids are set at 50% probability (123 K). Selected bond lengths [Å] and angles [deg]: P2–P1 2.9702(9), P2–C2 1.751(2), P1–C1 1.752(2), N1–P1 1.694(2), N1–P2 1.698(2), C1–C2 1.449(3), C2–C3 1.400(3), C3′–C1 1.400(3), P1–N1–P2 122.3(1), C1–P1–N1 93.14(9), C2–P2–N1 93.11(9), C2–C1–P1 115.8(1), C1–C2–P2 115.7(1), N1–P1–P2–C2 179.9(1), P1–C1–C2–C3 179.8(2). Symmetry code (′): (1–*x*, 1–*y*, 2–*z*).

The solid-state structure was determined by SCXRD (Figure 6). **1EMind** crystallized in the triclinic space group *P*1̅ with one centrosymmetric molecule and four cocrystallized benzene molecules in the unit cell (Figure 6). There are no significant intermolecular interactions in the solid-state structure. In contrast to **4Ter**, the annulated tricyclic ring system is planar. The EMind substituents are oriented almost orthogonally to the tricyclic ring system (∠ = 83.1(4)°). All C–C bond lengths are in the range between 1.400(3) (C1–C3′) and 1.449(3) Å (C1–C2), indicating partial double bond character (cf. ∑*r*_cov._(C–C) = 1.50, ∑*r*_cov._(C=C) = 1.34 Å).^52^ The P–C bonds (*d*(P1–C1) = 1.752(2) and *d*(P2–C2) = 1.751(2) Å) are slightly longer than a typical P–C double bond (cf. ∑*r*_cov._(P=C) = 1.69 Å)^52^ but significantly shorter than a single bond (cf. ∑*r*_cov._(P–C) = 1.86 Å), indicating some double-bond character, in agreement with the Lewis resonance scheme of **1** (see Electronic Structure section). The transannular P1···P2 distance amounts to 2.9702(9) Å, which is much too long for a covalent P–P interaction (cf. ∑*r*_cov._(P–P) = 2.2 Å),^52^ but significantly shorter than the sum of the van der Waals radii (cf. ∑*r*_vdW_(P···P) = 3.8 Å).^55^ Together with the computed electronic structure of **1** (see Electronic Structure section), this is indicative of a singlet biradical-type interaction between each pair of P atoms, as expected for a tetraradical of type **1**.

## Theoretical Aspects of Tetraradical vs Dimer Formation

**Scheme 4:**
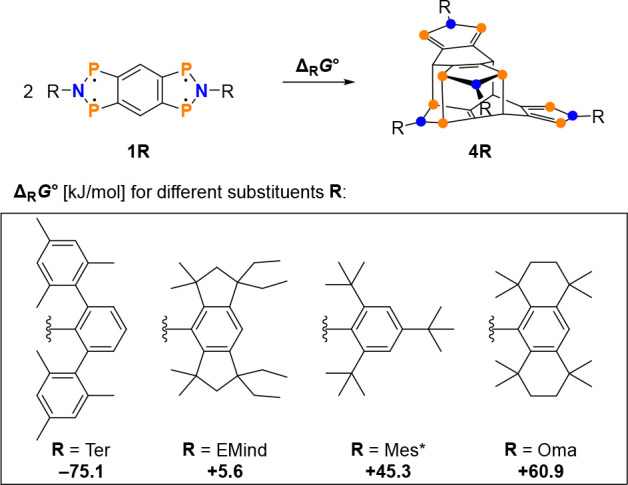
Gibbs Free Energies (Δ_R_*G*°) in Toluene (SMD Solvation Model)^59^ for the Dimerization of **1R** to **4R**, Calculated at PBE-D3/def2-TZVP Level of Theory

To gain access to the desired stable tetraradical **1R**, the sterically demanding substituent at the nitrogen had to be modified, since in the case of R = Ter we could only isolate the dimer (see above, section on Synthesis). The steric requirement of a suitable candidate must be large enough to stabilize the tetraradical against dimerization, but small enough to allow activation of small molecules (see below, section on H_2_Activation). Varying the substituent is very time consuming from a preparative point of view, so we decided to perform quantum mechanical calculations at the PBE-D3/def2-TZVP^56−58^ level of theory to select a suitable substituent for synthesis after we found that **1Ter** dimerizes, making isolation of **1Ter** impossible. For this reason, we examined theoretically four different bulky substituents (R = Ter, EMind, Mes*, and Oma; Scheme 4) in more detail in terms of steric requirements and thermodynamics of dimerization.

### Steric Influence

Good measures of the bulkiness of a substituent are the cone angle^60−62^ and the concept of buried volume,^63−65^ which can be used to rationalize and illustrate steric hindrance of the substituent in **1R**. The calculated cone angles^66^ in **1R** (computed at the optimized N–C distance, see SI) increase along the series R = Ter (222°) < EMind (246°) < Mes* (265°) = Oma (265°). A similar situation was found for the buried volumes. When the center of the transannular P–P axis is chosen as the spherical center in the computation of the buried volume, the radical centers (region between 2 and 3 Å) are best sterically shielded in **1R** for R = Oma (32.6%) > Mes* (30.9%) > EMind (27.5%) > Ter (24.2%).

**Figure 7:**
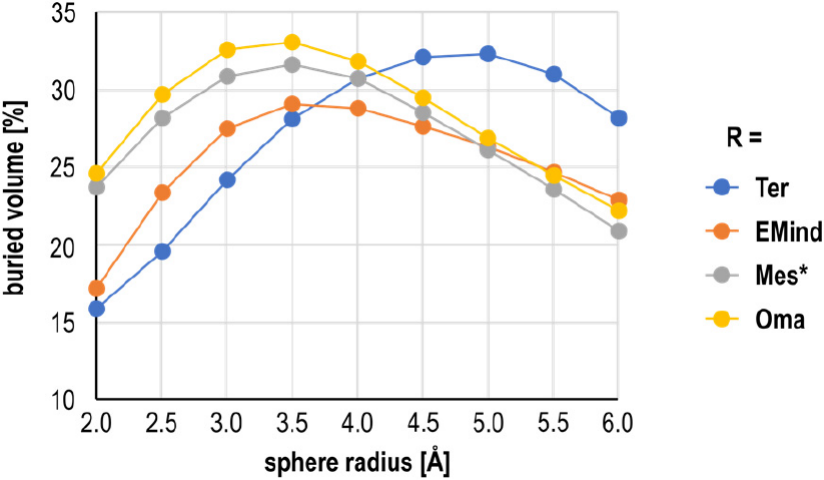
Buried volume as a function of the sphere radius in **1R** (R = Ter, EMind, Mes*, and Oma).

Interestingly, this order changes with increasing spherical radius, as can be seen in Figure 7 (see also SI, Table S8). At a large radius > 4 Å, the steric shielding of the terphenyl substituent becomes significantly larger compared to the other three substituents, which now have similar values (see SI, Table S7). However, the steric shielding at large sphere radii seems to be of secondary importance for the stability of the tetraradicals **1R** with respect to dimerization.

### Thermodynamics of Dimerization

In agreement with experiment, the dimerization of **1Ter** to **4Ter** in toluene (SMD solvation model)^59^ is considerably exergonic at the level of theory applied (Δ_R_*G*° = −75.1 kJ/mol, Scheme 4). However, for all three other substituents, the monomer is energetically preferred over the dimer. From these thermodynamic considerations in combination with the studies on steric influences, we decided to use the EMind substituent for the preparation of **1** since the starting materials, in particular the amine EMindNH_2_, can be easily prepared (see SI, p S34 ff).

## Electronic Structure of Tetraradical 1

The electronic structure of the tetraradical was studied using both a proton-substituted model system (**1H**) as well as the actual system **1EMind** (optimized at the PBE-D3/def2-TZVP^56−58^ level of theory). As the EMind substituent is oriented orthogonally to the ring plane of the central annulated ring system and therefore does not allow delocalization of the π electron system into the substituent, the results obtained for **1H** and **1EMind** do not deviate significantly. Thus, for reasons of clarity, we will discuss only the results for the model system here; further information about **1EMind** can be found in the SI (p S77 ff).

### NBO Picture

**Scheme 5:**
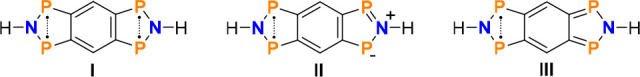
Formal Lewis Representations of **1H** Derived from NBO/NLMO Analysis Lone pairs are omitted for clarity. Only one Lewis structure per type is shown. Due to symmetry there are two type **I**, eight type **II**, and two type **III** structures. The radical electrons are localized in a π*-type orbital, which is indicated by the dotted lines.

NBO analysis finds Lewis representation **I** (Scheme 5) as the energetically most favorable Lewis structure for **1H**. Structure **I** describes a P-centered tetraradical with a benzene linker that has six delocalized π electrons. Together with one lone pair of electrons on both nitrogen atoms and the four radical electrons at the P atoms, all of which are localized in p-atomic orbitals, this results in a total of 14 π electrons. No formal charges are needed. The formal radical electrons are localized in two π*(P–P) NBOs (Figure S30), in agreement with the CASSCF wave function (vide infra). The biradical subunits in **1H** may therefore be classified as type-II biradicals according to the scheme of Abe,^5^ analogously to other known P-centered biradicals.^16,23,28,43,67^ Other important Lewis structures are those in which one five-membered ring is a formal biradical, whereas the remaining two rings are described using C=C, P=C, or P=N double bonds (Lewis formulas of type **II** and **III**; for a depiction of the corresponding NLMOs = Natural localized molecular orbital, see Figure S30). The delocalization of the lone pair on the N atom leads to formal charges and thus to zwitterionic character.

### MO Picture

First, we investigated the order of electronic spin and excited states of the tetraradical to verify the singlet ground state postulated on the basis of the sharp NMR resonances of **1EMind** and absence of signals in the EPR spectrum. In general, for a system with four electrons in four (frontier) orbitals, there are 20 possible singlet as well as 15 triplet states and one quintet state. For the description of **1H** we performed NEVPT2^68−70^/CAS(14,12)^71−79^/def2-TZVP^58,80^ calculations, which take into account all π-orbitals of the central ring system. According to these calculations, **1H** possesses a singlet ground state. The first excited state is the triplet state with Δ*E*_ST_ = *E*_S_ – *E*_T_ = −93 kJ/mol (**1EMind**: – 92 kJ/mol), which is significantly lower than in **E**^*t***Bu**^**Bhp** (Δ*E*_ST_ = −126 kJ/mol).^43^Table 1 contains the first 10 excited states of **1H** including the quintet state in their energetic order. The quintet state is clearly above the ground state with Δ*E*_SQ_ = −311 kJ/mol (**1EMind**: −313 kJ/mol). This clearly differs from an ideal tetraradical (or dis-tetraradical), in which the first six states (2× singlet, 3× triplet, 1× quintet) are degenerate, and indicates that the radical electrons of **1H** are rather strongly coupled. The interaction of two radical electrons (*i*, *j*) in polyradicals can be described by the electron-exchange coupling constants *J*_*ij*_, which result from the phenomenological Heisenberg–Dirac–van Vleck Hamiltonian^81,82^ (*Ŝ* = spin operator):
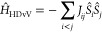
1

**Table 1:** Exited States of Model System **1H** (NEVPT2/CASSCF(14,12)/def2-TZVP)

excited state	term symbol	Δ*E* [eV]	Δ*E* [kJ/mol]
ground state	^1^*A*_*g*_	0.00	0
1	^3^*B*_2*u*_	0.97	93
2	^3^*B*_3*g*_	1.59	154
3	^1^*B*_2*u*_	1.95	188
4	^1^*A*_*g*_	2.66	256
5	^3^*B*_1*u*_	2.84	274
6	^3^*B*_3*g*_	3.00	290
7	^1^*B*_3*g*_	3.11	300
8	^3^*B*_2*u*_	3.11	300
9	^1^*B*_3*g*_	3.16	305
10	^5^*A*_*g*_	3.22	311

The larger the value of *J*, the stronger the interaction between two electrons. A positive value indicates a ferromagnetic coupling, while a negative value indicates antiferromagnetic coupling. To describe systems of four electrons, a maximum of six coupling constants is needed. However, **1H** can be described by only three coupling constants due to its *D*_2*h*_ symmetry (vide supra).

**Figure 8:**
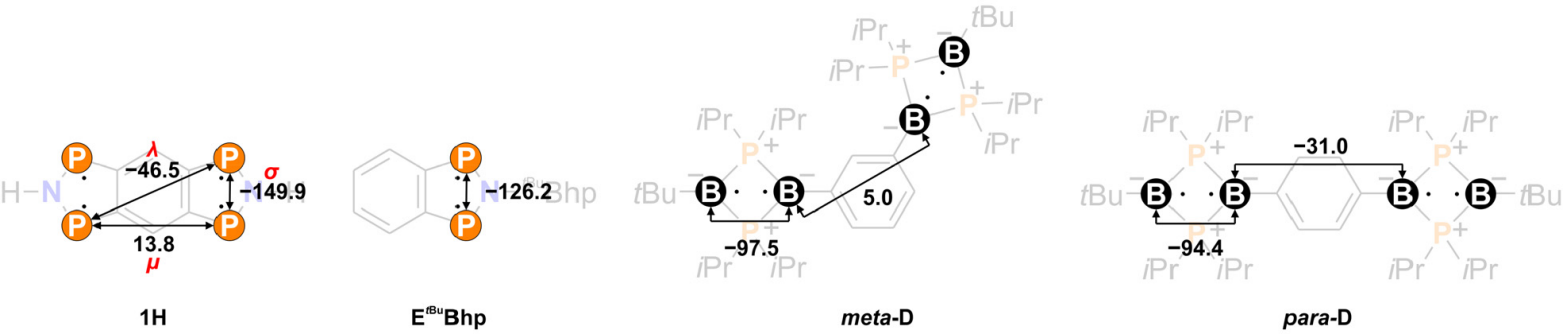
Electron exchange coupling constants in **1H**, **E**^*t***Bu**^**Bhp**, *meta***-D**, and *para***-D** in kJ/mol. **1H**: NEVPT2/CASSCF(14,12)/def2-TZVP, **E**^*t***Bu**^**Bhp**: value taken from ref (43), *meta***-D** and *para***-D**: values taken from ref (8).

In general, compound **1H** can be understood as a benzene, formally substituted by four radical units. By analogy with the work of Head-Gordon, Casanova, and co-workers,^8,83^ the coupling constant between the radical centers in *ortho* position to each other is called σ (short), in *meta* position μ (medium), and in *para* position λ (long-range coupling). The relationship between the energies of the excited states (Table 1) and the coupling constants can be derived from eq 1 and is shown in Table 2 (a derivation can be found in the SI, p S89 ff). Using a least-square fit, the coupling constants for **1H** can be obtained (Figure 8). They can be compared to the respective *ortho*-, *meta*-, and *para*-couplings of other bi- and tetraradically substituted benzenes.

**Table 2:** Energetic Dependency of the Electron Exchange Coupling Constants σ, μ, and λ

state	symbol	*E*_rel._ =
Q_1_	*A*_*g*_	–σ/2 – μ/2 – λ/2
T_3_	*B*_1*u*_	–σ/2 + μ/2 + λ/2
S_1_	*A*_*g*_	–σ/2 + μ + λ
T_2_	*B*_3*g*_	+σ/2 + μ/2 – λ/2
T_1_	*B*_2*u*_	+σ/2 – μ/2 + λ/2
S_0_	*A*_*g*_	+3/2σ

In **1H**, σ = −149.9 kJ/mol (**1EMind**: −150.8 kJ/mol), which shows a strong antiferromagnetic coupling between the radical electrons within each of the five-membered rings, similar to that in the five-membered biradical **E**^*t***Bu**^**Bhp** (−126.2 kJ/mol) and also on the same order of magnitude as found in the four-membered rings of the tetraradicals *meta***-D** (−97.5 kJ/mol) and *para***-D** (−94.4 kJ/mol). The *meta*-coupling μ = 13.8 kJ/mol (**1EMind**: 14.4 kJ/mol) is the only ferromagnetic coupling in **1H** and similar to the *meta*-coupling in ***meta-D*** (5.0 kJ/mol). The *para*-coupling λ = −46.5 kJ/mol (**1EMind**: –49.9 kJ/mol) shows a significant antiferromagnetic interaction between the electrons of the two formal biradicals and is slightly larger than in *para***-D** (−31.1 kJ/mol). Thus, it can be concluded that there are significant couplings between all radical electrons of **1H**, and these couplings are similar to analogous coupling pathways in **E**^*t***Bu**^**Bhp**, *meta***-D**, and *para***-D**.

### Excursus: How to Understand the Tetraradical Character

**Figure 9:**
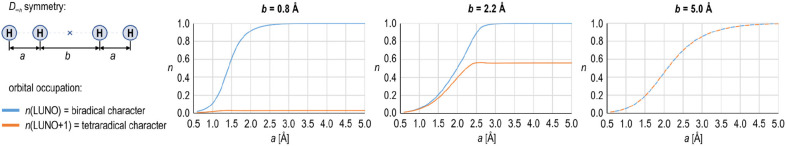
Bi- [*n*(LUNO)] and tetraradical character [*n*(LUNO+1)] in a chain of four H atoms in *D*_∞*h*_ symmetry at different distances *a* and *b*.

Another way to characterize tetraradicals and, in particular, quantify the polyradical character is to examine the occupancy of the LUNO (biradical character; LUNO = lowest unoccupied natural orbital) and LUNO+1 (tetraradical character). Before we come to discuss these values for **1H**, however, we want to explain the relationship between the two values by using a simple model system of four hydrogen atoms in *D*_∞*h*_ symmetry. It should be noted that the model is only loosely connected to the tetraradical **1H** (*D*_2*h*_ symmetry), but it is an intuitive model for tetraradicals in general, especially if they are to be understood as molecules with two biradical subunits. The system is defined by two variables, *a*, the distance between an outer H atom and its neighboring H atom, and *b*, the distance between the inner H atoms (Figure 9). Variation of *a* and *b* changes the interaction between the hydrogen atoms and thus *n*(LUNO) and *n*(LUNO+1). The occupancies were determined by simple CASSCF(4,4) calculations and are illustrated in Figure 9 as a function of *a* at three distances *b* (*b* = 0.8, 2.2, 5.0 Å).

The following conclusions and trends can be derived from this: (i) **Small*****a*** and ***b***: closed-shell system with minimal bi- and tetraradical character. (ii) **Increasing*****a*** at **small*****b*** (0.8 Å): H_2_ molecule in the middle, with radical hydrogen atoms on the outside, moving away from each other; the biradical character increases to perfect biradical; the tetraradical character remains small due to the strong interaction between the inner H atoms. (iii) **Increasing*****a*** at **medium*****b*** (2.2 Å): The initial situation describes two H_2_ molecules with medium distance to each other (minimal bi- and tetraradical character); with increasing *a*, the bi- and tetraradical character increase together, but the tetraradical character is limited at larger *a* by the interaction of the central H atoms; the final state describes two biradicals with significant interaction between each other. (iv) **Increasing*****a*** at **large*****b*** (5.0 Å): Parallel dissociation of two separated H_2_ molecules, biradical character = tetraradical character due to missing interaction between the biradical units. (v) **Large*****a*** and ***b***: maximum bi- and tetraradical character; no interaction between the H atoms, all valence orbitals are degenerate.

From these simple considerations it follows that the biradical character limits the tetraradical character, so discussing both values independently is not meaningful. The system of four H atoms can be transferred to the systems described in the Introduction, in which there are two sets of biradicals whose interaction depends on the type of linker (e.g., length, conjugation of the electrons, etc.).

### Tetraradical Character of **1**

**Figure 10:**
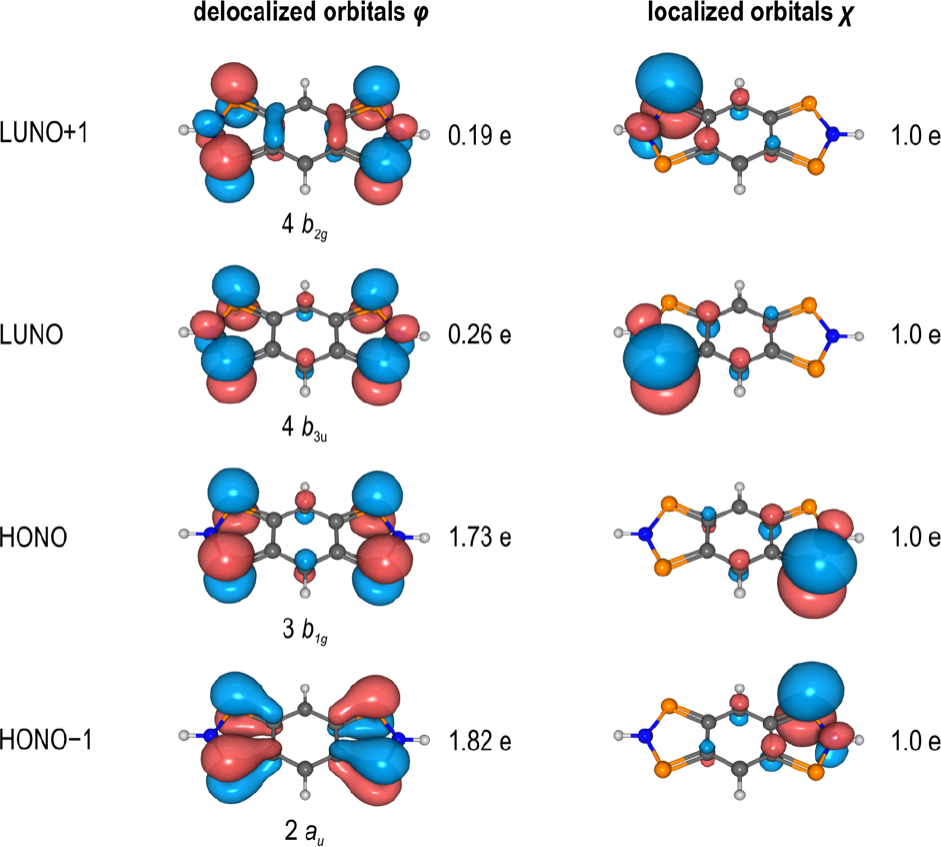
Delocalized and localized frontier orbitals of **1H** (CASSCF(4,4)/def2-TZVP).

In order to describe the bi- and tetraradical character of **1H**, a CASSCF(4,4) calculation, which does not take into account the dynamic correlation within the π-system, was performed. The orbitals of the active space are shown in Figure 10. HONO and HONO–1 (HONO = highest occupied natural orbital) describe a transannular antibonding situation between the P atoms within each five-membered ring, while LUNO and LUNO+1 are transannular bonding. The LUNO has an occupancy of 0.26 (26% biradical character, Table 3); the LUNO+1, of 0.19 (19% tetraradical character). The biradical character is significantly increased compared to **E**^*t***Bu**^**Bhp** (18%, CASSCF(2,2)),^43^ which shows that the ring extension to **1H** has a clear influence on the multireference character. This would not be the case if two biradicals were linked to form a bis(biradicaloid). The amount of biradical character is similar to nonaromatic P-centered biradicals such as [P(μ-NTer)]_2_ (28%) or **A** (28%, Scheme 1)^43^ By linear combination of the delocalized CASSCF(4,4) orbitals φ, localized orbitals χ are obtained (Figure 10), which show that the radical electrons are mainly localized at the P atoms, so that it is justified to speak of a P-centered tetraradical.

**Table 3:** Occupation Numbers *n* of the LUNO, LUNO+1, and LUNO+2 to Quantify the Bi- and Tetraradical Character for **1H** in Comparison with **E**

compound	active space	*n*(LUNO)	*n*(LUNO+1)	*n*(LUNO+2)
**E**^a^	CASSCF(2,2)	0.18		
	CASSCF(10,9)	0.21	0.09	0.09
**1H**	CASSCF(4,4)	0.26	0.19	
	CASSCF(14,12)	0.32	0.19	0.09

a Values taken from ref (43).

Additionally, the occupation numbers *n*(LUNO) and *n*(LUNO+1) of **1H** were determined using a CASSCF(14,12) calculation that describes all π-orbitals of the central ring system and the electrons contained therein (Table 3). In contrast to the CASSCF(4,4) calculation, dynamic and nondynamic correlation in the π-system are thus considered. The dynamic correlation leads to a significant increase in the occupation of the LUNO, while the occupation of LUNO+1 remains unaffected. The small LUNO+2 occupation (<10%) shows that a description of the system as tetraradical is sufficient due to the negligible hexaradical character, which is mainly attributable to dynamic correlation.

### Aromaticity of **1**

**Figure 11:**
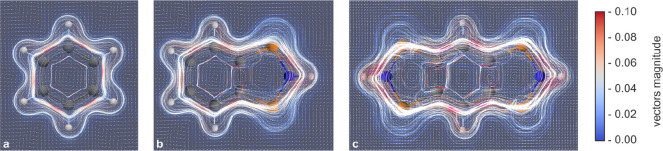
Streamline plot of the current density susceptibility^84^ for benzene (a), **EH** (b), and **1H** (c).

The influence of the π-system on the multireference character as well as the strong antiferromagnetic coupling of the radical electrons prompted us to investigate the aromaticity of **1H**, which we would like to discuss on the basis of magnetic parameters (magnetically induced ring current,^84,85^ NICS values^86−88^). Benzene and **EH** are used as comparison in this discussion (data of further compounds see SI p S98 f).^43,89^ The current density susceptibilities of benzene, **EH**, and **1H** are visualized in Figure 11 by streamline plots. All compounds show a clear diatropic ring current surrounding the ring systems above and below the ring plane, which is typical for aromatic compounds. By integration of the current density along vertical ring sections to the respective ring center, the net induced ring current can be derived (Table 4).^84,85,90,91^

**Table 4:** Net Induced Currents and NICS(1)_*zz*_ Values of Benzene, **EH**, and **1H**^a^

	C_6_H_6_	**EH**	**1H**
net induced current [nA/T]	12.1	13.5 (⬟)	13.2 (⬟)
		11.2 (⬟)	13.5 (⬟)
NICS(1)_*zz*_ [ppm]	–30.2	–31.1 (⬣)	–29.5 (⬣)
		–24.9 (⬢)	–29.4 (⬢)

a For fused ring systems, values are given for the five-membered (⬟) and six-membered part (⬢). Further information can be found in the SI, p S98 f.^43,89^

According to these computations, the ring currents are very similar (>11 nA/T) and positive in all rings, which means that the diatropic part prevails over the paratropic one.^84^ The opposite is true for antiaromatic compounds (negative sign), while nonaromatic compounds have ring currents around 0 nA/T. The determined NICS(1)_*zz*_ values between −24.9 and −31.1 also indicate the aromaticity of the compounds considered (Table 4; for NICS(0), NICS(0)_*zz*_, and NICS(1) see SI, p S98).^43^

## H_2_ Activation

### Theoretical Aspects

**Scheme 6:**
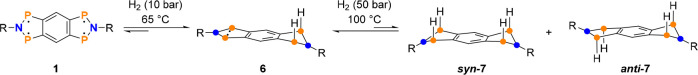
Activation of One Equivalent of H_2_ by the Tetraradical **1** Led to the Formation of **6**; Additionally, the Diadducts *syn-***7** Were Formed Depending on the Reaction Temperature and Pressure of H_2_

Metal-free activation of molecular hydrogen is an important challenge in molecular chemistry and succeeded in seminal works, for example, by conversion with FLPs,^92^ CAACs,^93^ or multiple bonds of heavy elements.^94^ For **1EMind**, first reactivity studies toward molecular hydrogen were carried out, with the aim to chemically prove the existence of the interactions between the two biradical units discussed above. We began our investigations with calculations in which the H_2_ activation occurs analogously to known four-,^15−17^ five-,^17^ and six-membered^18−20^ biradicals via the addition of H_2_ to the radical centers. In contrast to these biradicals, tetraradical **1EMind** enables not only the activation of one equivalent of H_2_ under the formation of **6** but additionally the activation of a second equivalent of H_2_ (*syn***-7H** and *anti***-7H**, Scheme 6).

The calculations for the model system **1H** show (Table 5) that the addition of the first equivalent of H_2_ is strongly exergonic (Δ_R_*G°* = −52.2 kJ/mol), whereas the addition of the second equivalent is only slightly exergonic (Δ_R_*G°*: *syn***-7H** = −12.4, *anti-***7H** = −12.7 kJ/mol). The activation barrier for the first activation step is 80.0 kJ/mol, while that for the second reaction step is significantly higher (Δ_R_*G*^⧧^: *syn***-7H** = 104.3, *anti-***7H** = 103.6 kJ/mol). The differences in the energy profiles for the first and second activation steps indicate that there is a significant interaction between the radical centers of **1H**. The formal removal of a biradical unit reduces the biradical character from 26% in **1H** (CASSCF(4,4), cf. Table 3) to 17% in **6H** (CASSCF(2,2), see SI p S90) and thus the reactivity toward molecular hydrogen. In contrast, for a bis(biradicaloid), the energy profiles for the first and the second activation would be identical. In the model system, steric effects are reduced as much as possible due to the smallest possible substituent (R = H) and the smallest possible molecule to be activated (H_2_).

**Table 5:** Δ_R_*G*^⧧^ and Δ_R_*G*° (DLPNO-CCSD(T)/def2-TZVP) for the Activation of H_2_ by **1EMind** and **6EMind** (Values in Parentheses for **1H** and **6H**, Respectively)

reaction			
from	to	Δ_R_*G*^⧧^ [kJ/mol]	Δ_R_*G°* [kJ/mol]
**1** + H_2_	**6**	100.9 (80.0)	–37.7 (−52.2)
**6** + H_2_	*syn-***7**	118.1 (104.3)	–2.4 (−12.4)
**6** + H_2_	*anti-***7**	119.5 (103.6)	–1.3 (−12.7)

**Figure 12:**
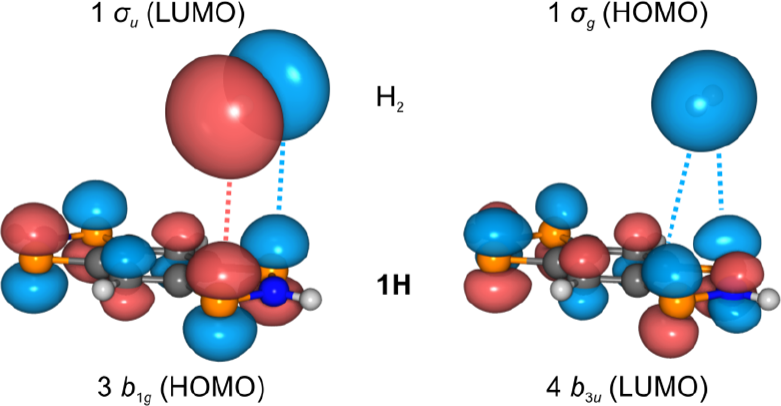
Schematic representation of the interaction of the frontier MOs of the model system **1H** (*D*_2*h*_ symmetry) and H_2_ (*D*_∞*h*_).

The calculations for the EMind-substituted tetraradical (**1EMind**) show larger activation barriers and decreased energy gains, indicating that hydrogen activation is made more difficult upon introduction of bigger substituents. This is due to bending of the ring system during H_2_ addition, which leads to an energetically disfavored spatial proximity of the two EMind substituents. The reactions are predicted to proceed in a concerted mechanism as [2+2] cycloadditions, which may be nicely rationalized by the HOMO–LUMO interactions shown in Figure 12, analogous to the H_2_ activation with [P(μ-NTer)]_2_.^15^

### Experimental Aspects

**Figure 13:**
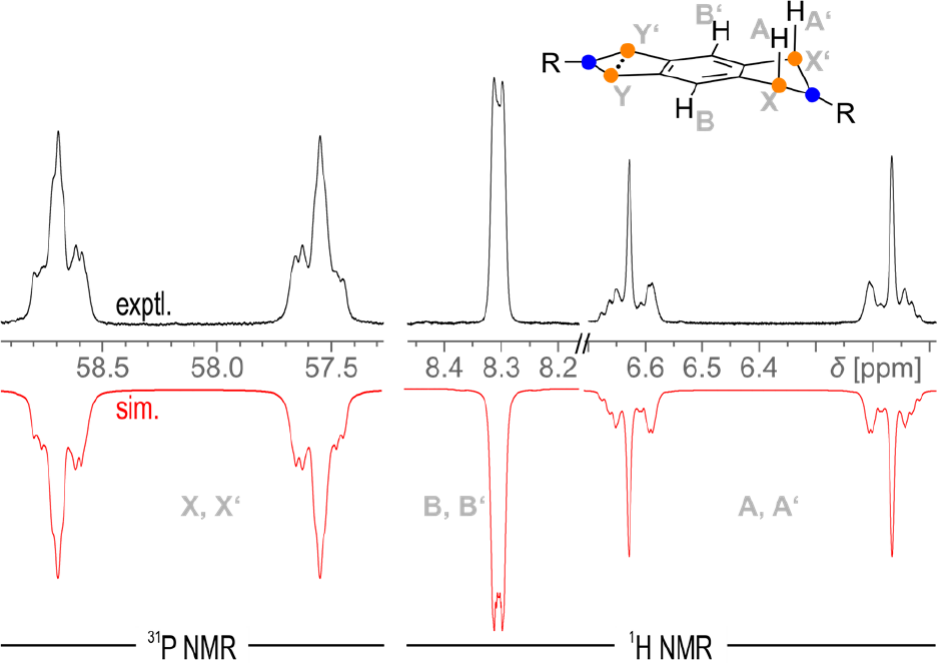
Experimental and simulated ^1^H and ^31^P NMR spectra of **6EMind**. The resonances of P_Y_ and P_Y′_ appear as a singlet and are not shown here.

To validate the calculations (e.g., with regard to the postulated mechanism) and to select suitable reaction conditions for the synthesis of **6EMind** and **7EMind**, NMR investigations were carried out. First, an NMR tube with a solution of **1EMind** was pressurized with 5 bar H_2_ at 67 °C for 20 min. The ^31^P and ^1^H NMR spectra of the experiment showed a 75% conversion to **6EMind** (Figure 13), with ^31^P resonances at 287 ppm (P_Y_) and 58 ppm (P_X_). The subsequent increase in the temperature to 87 °C led to an almost complete conversion after 10 min time. The corresponding ^1^H and ^31^P NMR spectra fit well to an AA′BB′XX′YY′ spin system (A, B = ^1^H; X, Y = ^31^P) based on calculated values (PBE-D3/def2-TZVP). Coupling constants > |10 Hz| can be found between H_A_–P_X_ (183 Hz) and P_X_···P_X′_ (−26 Hz; for all exptl. and calcd. parameters see SI, p 68 ff).

**Figure 14:**
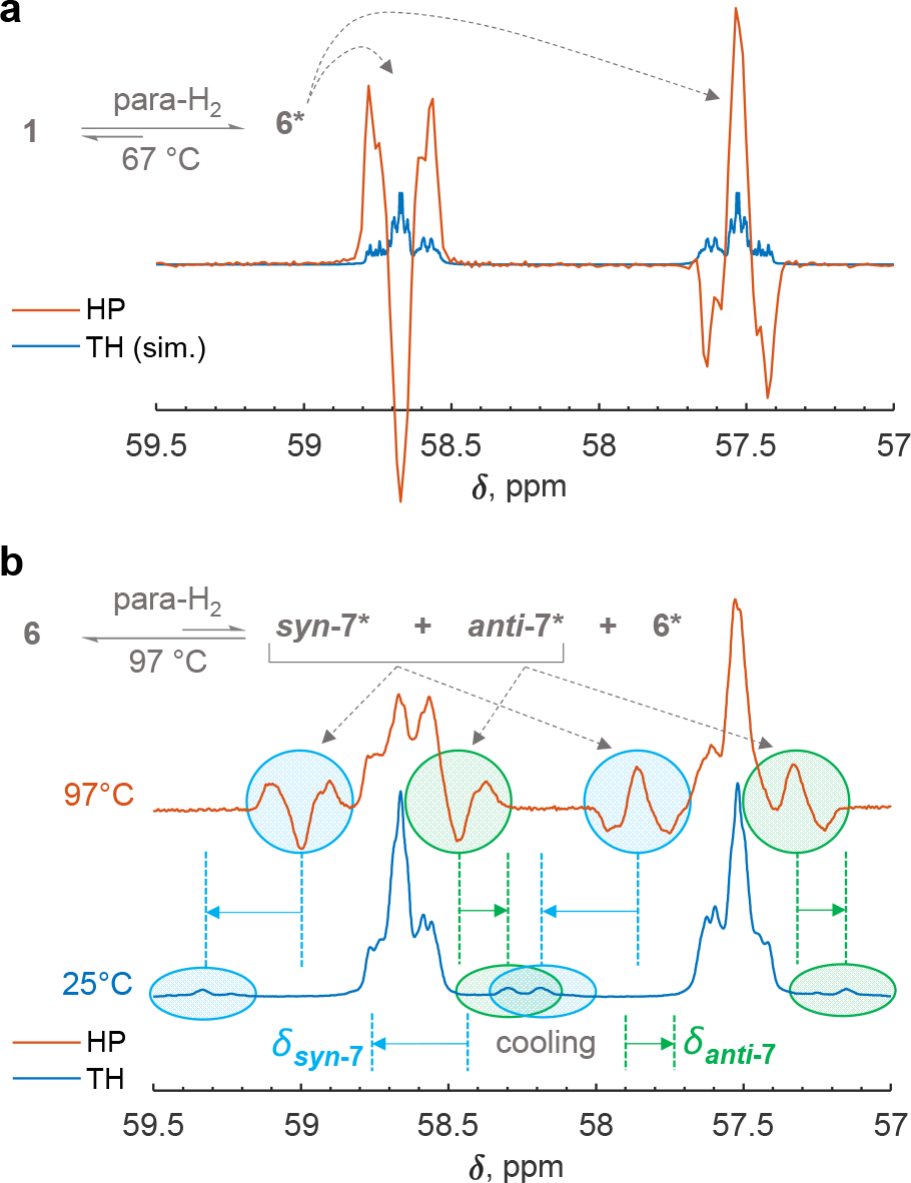
^31^P NMR spectra measured during para-H_2_ activation by (a) **1EMind** and (b) **6EMind** at 67 and 97 °C, respectively. A 5 bar para-H_2_ pressure was used in the experiments. (a) The formation of hyperpolarized 6EMind* was observed in the reaction of **1EMind** and para-H_2_. A simulated thermal spectrum of **6EMind** is depicted to show the qualitative alternation of the XX′ ^31^P multiplet (see Figure 13) with the hyperpolarization. (b) The reaction of **6EMind** and para-H_2_ resulted in hyperpolarized *syn-*7EMind* and *anti-*7EMind* species. In addition, **6EMind** itself became slightly hyperpolarized. The resonances of *syn-***7EMind** and *anti-***7EMind** drifted apart under cooling to 25 °C, likely indicating the chemical exchange between the isomers. For the sake of simplicity, “EMind” was omitted from the endings of the compound names in the figure. Asterisk (*) denotes hyperpolarized species, HP = hyperpolarized, TH = thermal.

The hydrogen activation was then repeated with parahydrogen (para-H_2_), the spin-0 isomer of H_2_, inside a 9.4 T NMR spectrometer. In this case, a concerted activation would lead to a several orders of magnitude signal enhancement of the NMR resonances (^1^H and ^31^P) due to the strong nuclear spin hyperpolarization. This phenomenon,^95−97^ generally known as parahydrogen-induced polarization (PHIP),^98,99^ has already been used by us to verify concerted reaction mechanisms for the activation of H_2_ by other biradicals (e.g., [P(μ-NTer)]_2_, **A**).^17,100^Figure 14 shows ^31^P NMR spectra of the resulting hyperpolarized species after para-H_2_ activation (5 bar) by **1EMind** and **6EMind** at 67 and 97 °C, respectively. At 67 °C, only the resonances of hyperpolarized 6EMind* are visible (Figure 14a). For comparison, a simulated thermal spectrum is also presented in the figure to highlight qualitative amplitude alternations in the XX′ ^31^P NMR multiplet of **6EMind** (see Figure 13 for the notation). The observation of PHIP-enhanced resonances in this experiment proves the concerted reaction mechanism of H_2_ activation by **1EMind**. In turn, the experiment at 97 °C showed that **6EMind** also activates H_2_ in a concerted manner, since the two isomers of the double addition product, *syn-***7EMind** and *anti***-7EMind**, were also hyperpolarized by PHIP (Figure 14b). As from the NMR point of view both species have the same symmetry, it cannot be elucidated directly from the NMR spectra which of the two sets of XX′ ^31^P NMR resonances centered at 58.4 and 57.9 ppm corresponds to which isomer of **7EMind**. The tentative assignment of signals in Figure 14 is based on calculated shifts (see SI, p S72 ff). It is worth noting, however, that *syn-***7EMind** and *anti***-7EMind** most likely interconvert into each other since their resonances drifted apart (ca. 0.5 ppm) when the sample was cooled to room temperature, indicating the presence of dynamic exchange at 97 °C. In addition, this para-H_2_ experiment revealed a significant distortion of the thermal signal multiplet corresponding to **6EMind**, implying that some portion of this compound was converted into the hyperpolarized form, 6EMind*, via the reversible dissociation into **1Emind** and H_2_ at 97 °C.

The conditions for the attempted synthesis and isolation of **6EMind** and **7EMind** were chosen on the basis of the NMR experiments. Thus, **6EMind** is formed exclusively at moderate H_2_ pressures and low temperatures, whereas **7EMind** is present only in small amounts at high temperatures, so that the pressure of H_2_ must be increased significantly to achieve a higher reaction conversion.

**Figure 15:**
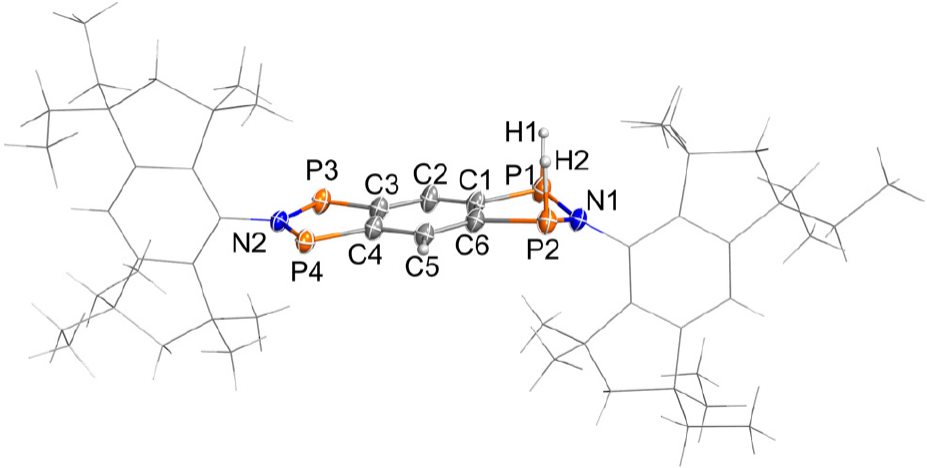
Molecular structure of **6EMind** in the crystal. Ellipsoids are set at 50% probability (173 K). Selected bond lengths [Å] and angles [deg]: C1–P1 1.825(1), C6–P2 1.832(1), C3–P3 1.737(1), C4–P4 1.739(1), P1–P2 2.9350(6), P3–P4 2.9566(6), C1–P1–P2–N1 150.06(9), C3–P3–P4–N2 179.6(1).

**6EMind** was therefore synthesized in the reaction of **1EMind** in toluene with H_2_ (10 bar) at 65 °C within 2.5 h (alternatively with 1 atm H_2_ at ambient temperature within 4 weeks) and was crystallized as yellow crystals from 1,2-dichlorobenzene. The solid-state structure was determined by SCXRD. **6EMind** crystallized in the triclinic space group *P*1̅ with two molecules and two cocrystallized solvent molecules in the unit cell (Figure 15). Compared to the structure of **1EMind**, the H_2_-substituted five-membered ring in **6EMind** is strongly altered, while the unsubstituted ring remains almost unchanged. The first ring is bent along the P atoms (along P1···P2: 30.6°), and the C–P distances are in the range of single bonds (*d*(C1–P1) = 1.825(1), *d*(C6–P2) = 1.832(1) Å, cf. Σ*r*_cov._(C–P) = 1.86 Å),^52^ whereas the second still biradical ring is almost planar (along P3···P4: 0.7(5)°) and the C–P distances are in the range of a double bond (*d*(C3–P3) = 1.737(1), *d*(C4–P4) = 1.739(1) Å, cf. ∑*r*_cov._(P=C) = 1.69 Å).^52^ Furthermore, the H_2_ addition leads to a shortening of the transannular P–P distance (*d*(P1–P2) = 2.9350(6) vs *d*(P3–P4) = 2.9566(6) Å).

The formation of **6EMind** is reversible, in agreement with the para-H_2_ NMR experiments. Thus, **6EMind** turned green in the solid at 120 °C, the color of **1EMind**. An analysis of a sample of **6EMind** heated to 120 °C for 1 h in a vacuum showed a significant re-formation of the tetraradical **1EMind** as evidenced by ^31^P NMR spectroscopy.

Our attempts to isolate the doubly substituted compounds *syn-***7EMind** and *anti-***7EMind** were not successful. The hydrogenation of **1EMind** (50 bar H_2_, 100 °C in toluene) over a period of 3 h resulted in the formation of a mixture of **6EMind** (76%), *syn***-7EMind** (12%), and *anti-***7EMind** (12%, determined by ^31^P{^1^H} NMR spectroscopy). The ratio of the diadducts (**7EMind**) could not be increased by extending the reaction time to 48 h, so the reaction was already in equilibrium after 3 h.

## Conclusion

In summary, we report the successful synthesis of **1R**, an isolable singlet tetraradicaloid species with radical centers localized at the four P atoms. Theoretical and experimental investigations of different substituents show that EMind is a suitable substituent for the stabilization of **1R**, whereas the Ter-substituted derivative dimerizes to the unusual cage compound **4Ter**. The tetraradical **1EMind** is a compound stable at high temperatures (*T*_dec._ = 365 °C) and can be synthesized in gram scale. Theoretical studies indicate that the radical electrons interact with each other to a considerable extent. Despite the coupling of the radical electrons, **1EMind** has a significant bi- (26%) and tetraradical character (19%) according to CASSCF(4,4) calculations and is aromatic. The interaction of the radical electrons additionally affects the reactivity of **1EMind**. In the reaction with H_2_, the addition of the first equivalent is much quicker than the second. The hydrogen activation proceeds in a concerted [2+2] cycloaddition, which has been proven by PHIP-NMR studies.

## Experimental Section

Experimental section, preparation of starting materials and compounds, structure elucidation, additional spectroscopic details, and computational details can be found in the Supporting Information.

Computations were carried out using Gaussian09,^101^ ORCA 4.2.1^102^ or ORCA 5.0.3,^103^ and the standalone version of NBO 6.0.^104−107^

## Supporting Information Available

Additional experimental information, computational details (PDF)
