# Supplementary material for: Rational Design of Persistent Phosphorus-Centered Singlet Tetraradicals and Their Use in Small-Molecule Activation
Source: J Am Chem Soc. 2023 Jun 14;145(26):14484–97. doi: 10.1021/jacs.3c03928 (PMC10368346; doi:10.1021/jacs.3c03928)
Supplement: Supplementary file 1 — ja3c03928_si_001.pdf [file ja3c03928_si_001.pdf]

SUPPORTING INFORMATION

Rational Design of Persistent Phosphorus-Centered Singlet Tetraradicals and Their Use in Small-Molecule Activation

Edgar Zander, Jonas Bresien, Vladimir V. Zhivonitko, Johannes Fessler, Alexander Villinger, Dirk Michalik, Axel Schulz**

This file includes:

1 Experimental S4

2 Structure elucidation S8

3 Syntheses of starting materials S17

3.1 TerI S17

3.2 $TerNH_2$ S21

3.3 1,3-Bis(1-chloro-1-methylethyl)benzene S24

3.4 EMindH S28

3.5 $EMindNO_2$ S31

3.6 $EMindNH_2$ S35

4 Syntheses of compounds S38

4.1 **3Ter** S38

4.2 **3EMind** S41

4.3 **3Mes*** S44

4.4 **3^{tBu}Bhp** S46

4.5 **4Ter** S47

4.6 Thermal stability of **4Ter** S52

4.7 **5Ter** S53

4.8 **1EMind** S56

4.9 **6EMind** S61

4.10 **7EMind** S67

5 Additional spectroscopic details S69

5.1 ^{31}P NMR data of **6EMind** S69

5.2 ^{31}P NMR data of **7EMind** S72

6 Parahydrogen experiments S75

6.1 General information S75

6.2 Reaction of **1EMind** with para-H_2 ..... S75

6.3 Reaction of **6EMind** with para-H_2 ..... S76

7 Computational details S77

7.1 General remarks S77

7.2 Summary of calculated data S80

7.3 Steric demand (cone angle, buried volume) in **1R** S82

7.4 NBOs and NLMOs of **1H** S85

7.5 CASSCF computations of **1H**, **1EMind** and **6H** S86

7.6 Exited states and electron exchange coupling constants S91

7.7 Induced ring currents S98

7.8 Optimized structures (.xyz-files) S100

8 References S168

1 Experimental

General Information. If not stated otherwise, all manipulations were carried out under oxygen- and moisture-free conditions in an inert argon atmosphere using standard Schlenk or drybox techniques. All glassware was heated three times *in vacuo* using a heat gun (650 °C) and cooled under argon atmosphere. Autoclaves with glass inlet and Teflon stir bar were heated on a heating plate to 150 °C and were evacuated and filled with argon three times. Solvents were transferred using syringes, which were purged three times with argon prior to use. Solvents and reactants were either obtained from commercial sources or synthesized as detailed in Table S1.

Table S1: Origin and purification of solvents and reactants.

**Substance**	**Origin**	**Purification**
Ac_2O	vwr	used as received
AcOH	vwr	used as received
benzene	Carl Roth, ≥99.5%	dried over Na/benzophenone<br>freshly distilled prior to use
CH_2Cl_2	local trade	*non-inert gas conditions:*<br>used as received<br><br>*inert gas conditions:*<br>purified according to literature procedure[1]<br>dried over P_4O_{10}, stored over CaH_2<br>freshly distilled and degassed (freeze-pump-thaw)<br>(for the synthesis of EMindH CH_2Cl_2 was not degassed)
1,2-dichlorobenzene	Fisher Scientific	dried over CaH_2<br>freshly distilled prior to use
Et_2O, THF	local trade	*non-inert gas conditions:*<br>used as received<br><br>*inert gas conditions:*<br>dried over Na/benzophenone<br>freshly distilled prior to use
i-PrOH, EtOH	local trade	used as received
n-hexane	local trade	used as received

Substance	Origin	Purification
toluene	local trade	dried over Na/benzophenone<br>freshly distilled prior to use
$CDCl_3$	euriso-top	used as received
CD_2Cl_2	euriso-top	dried over P_4O_{10} and CaH_2<br>freshly distilled prior to use
C_6D_6	euriso-top	dried over Na<br>freshly distilled prior to use
THF-d_8	euriso-top	dried over Na<br>distilled and stored over molecular sieves (4 Å)
toluene-d_8	euriso-top	dried over Na<br>distilled and stored over molecular sieves (4 Å)
BCl_3 (1 M in hexane)	Thermo scientific (septum bottle)	used as received
1,3-bis(1-hydroxy-1-methylethyl)benzene	TCI, >98.0%	used as received
$CaCl_2$	local trade	used as received
1,3-dichlorobenzene	TCI, ≥95%	used as received
2-ethyl-1-butene	TCI, >97%	Dried over molecular sieves, filtered, recondensed and degassed.
H_2	Air Liquide, 99,999%	passed through Sicapent®
HCl (gas)	Linde, 99.8%	used as received
HNO_3 (65 %)	Chemsolute, p.a.	used as received
I_2	old stock	used as received
$LiAlH_4$	abcr, 97%	used as received, stored under Ar in the glovebox
Mes*NH_2	synthesized[2]	purified as described in the literature
MesBr	TCI, ≥99.0	used as received
Mg	old stock	used as received
$MgSO_4$	Local trade, 99%	used as received
Na_2CO_3	old stock	used as received
$NaHCO_3$	Fisher Scientific, ≥99 %	used as received
NaOH	Fisher Scientific	used as received
Na_2SO_3	vwr	used as received
n-BuLi (2.5 M in hexane)	Acros Organics	used as received
NEt_3	Sigma Aldrich, 99 %	dried over Na<br>freshly distilled prior to use
TMSCl	Aldrich, purified by redistillation, ≥99 % (septum bottle)	used as received

Substance	Origin	Purification
tolan	abcr	sublimed
tosyl azide	synthesized[3]	purified according to literature procedure
^{tBu}Bhp-NH_2	synthesized[4]	re-crystallized as described in the literature
Zn	dust, Acros Organics, 98+%	stored under Ar, activated by stirring with glass stirring bar for several weeks
**2**	synthesized[5]	re-crystallized as described in the literature

NMR spectra were recorded on Bruker spectrometers (AVANCE 250, AVANCE 300 or AVANCE 500) and were referenced internally to the deuterated solvent (^{13}C: $CDCl_3$ δ_{ref} = 77.0 ppm, CD_2Cl_2 δ_{ref} = 54.0 ppm, C_6D_6 δ_{ref} = 128.4 ppm, THF-d_8 $\delta_{ref,1}$ = 25.4 ppm, $\delta_{ref,2}$ = 67.6 ppm, toluene-d_8 $\delta_{ref,1}$ = 20.4 ppm, $\delta_{ref,2}$ = 125.5 ppm, $\delta_{ref,3}$ = 128.3 ppm, $\delta_{ref,4}$ = 129.2 ppm, $\delta_{ref,5}$ = 137.9 ppm), to protic impurities in the deuterated solvent (^{1}H: $CHCl_3$ δ_{ref} = 7.27 ppm, $CHDCl_2$ δ_{ref} = 5.32 ppm, C_6HD_5 δ_{ref} = 7.16 ppm, THF-d_7 $\delta_{ref,1}$ = 1.73 ppm, $\delta_{ref,2}$ = 3.58 ppm, toluene-d_7 $\delta_{ref,1}$ = 2.09 ppm, $\delta_{ref,2}$ = 6.98 ppm, $\delta_{ref,3}$ = 7.00 ppm, $\delta_{ref,4}$ = 7.09 ppm) or externally (^{31}P: 85% H_3PO_4 δ_{ref} = 0 ppm). All measurements were carried out at ambient temperature unless denoted otherwise. NMR signals were assigned using experimental data (e.g. chemical shifts, coupling constants, integrals where applicable) in conjunction with computed NMR data (GIAO method, *cf.* Computational details, p. S77). The signs of $^{n}J(^{31}P,^{31}P)$ coupling constants were derived from the calculated spectra.

For NMR spectra simulation, the calculated and experimental ^{31}P NMR spectra were transferred to gNMR.[6] The full lineshape iteration procedure of gNMR was applied to match the calculated to the experimental spectrum.

IR spectra of crystalline samples were recorded on a Bruker Alpha II FT-IR spectrometer equipped with an ATR unit at ambient temperature under argon atmosphere.

Raman spectra of crystalline samples were recorded using a LabRAM HR 800 Horiba Jobin YVON Raman spectrometer equipped with an Olympus BX41 microscope with variable lenses. The samples were excited by a red laser (633 nm, 17 mW, air-cooled

HeNe laser) or a green laser (532 nm, 50 mW, air-cooled, frequency-doubled Nd:YAG solid-state laser). All measurements were carried out at ambient temperature unless stated otherwise.

Elemental analyses were obtained using an Elementar vario Micro cube CHNS analyser.

Melting points (uncorrected) were determined using a Stanford Research Systems EZ Melt at a heating rate of 20 °C/min.

DSC analyses were carried out at a heating rate of 5 °C/min using a Mettler-Toledo DSC 823e.

Mass spectra were recorded on a Thermo Electron MAT 95-XP sector field mass spectrometer using crystalline samples.

UV-Vis spectra were acquired on an Agilent Technologies Cary 60 UV-Vis spectrometer.

2 Structure elucidation

X-ray Structure Determination: X-ray quality crystals were selected in Fomblin YR-1800 perfluoroether (Alfa Aesar) at ambient temperature. The samples were cooled to 123(2) K during measurement. The data were collected on a Bruker D8 Quest diffractometer or a Bruker Kappa Apex II diffractometer using Mo K_α radiation (λ = 0.71073 Å). The structures were solved by iterative methods (SHELXT)[7] and refined by full matrix least squares procedures (SHELXL).[8] Semi-empirical absorption corrections were applied (SADABS).[9] All non-hydrogen atoms were refined anisotropically, hydrogen atoms were included in the refinement at calculated positions using a riding model.

The structure of $EMindNH_2$ (*vide infra*) is almost identical to a the structure previously published by Zhang et al.[10]

Table S2: Crystallographic details.

Compound	EMindNH$_2$	**3Ter**	**3EMind**
Chem. Formula	$C_{24}H_{39}N$	$C_{54}H_{52}Cl_4N_2P_4{\cdot}4(C_6H_6)$	$C_{54}H_{76}Cl_4N_2P_4{\cdot}(C_6H_6)$
Formula weight [g/mol]	341.56	1307.08	1096.95
Color	colorless	colorless	colorless
Crystal system	triclinic	orthorhombic	monoclinic
Space group	$P\bar{1}$	*Pnma*	$P2_1/c$
a [Å]	9.3097(7)	25.299(6)	14.2338(5)
b [Å]	9.8978(8)	20.051(4)	22.9776(9)
c [Å]	12.2291(9)	14.158(3)	35.5326(13)
α [°]	89.489(3)	90	90
β [°]	70.101(3)	90	91.7230(10)
γ [°]	76.386(3)	90	90
V [Å^3]	1026.68(14)	7182(3)	11616.0(7)
Z	2	4	8
$\rho_{calcd.}$ [g/cm^3]	1.105	1.209	1.254
μ [mm^{-1}]	0.062	0.297	0.353
T [K]	123(2)	203(2)	123(2)
Measured reflections	33420	193390	487175
Independent reflections	5467	8047	40297
Reflections with $I > 2\sigma(I)$	3947	6767	30641
R_{int}	0.0592	0.0437	0.0570
F(000)	280	2744	4672
$R_1(R[F^2>2\sigma(F^2)])$	0.0486	0.0524	0.0463
w$R_2(F^2)$	0.1389	0.1334	0.1259
GooF	1.022	1.120	1.059
No. of Parameters	242	529	1.059
CCDC #	2251386	2251387	2251388

Compound	**3Mes***	**4Ter**	**5Ter**
Chem. Formula	$C_{42}H_{60}Cl_4N_2P_4$	$C_{108}H_{102}N_4P_8 \cdot 3(C_4H_8O)$	$C_{82}H_{72}N_2P_4$ $\cdot$ 0.82(C_4H_8O) $\cdot$ 0.095(CH_2Cl_2)
Formula weight [g/mol]	1.266	1922.02	1276.52
Color	colorless	yellow	colorless
Crystal system	monoclinic	triclinic	monoclinic
Space group	$P2_1/c$	$P\bar{1}$	$P2_1/n$
a [Å]	19.041(2)	15.0075(10)	16.8459(9)
b [Å]	10.2103(6)	18.9963(13)	24.4440(15)
c [Å]	11.8800(7)	19.9380(13)	17.0426(10)
α [°]	90	96.998(3)	90
β [°]	102.823(2)	107.392(3)	98.562(2)
γ [°]	90	100.596(3)	90
V [Å^3]	2252.1(2)	5236.1(6)	6939.6(7)
Z	2	2	4
$\rho_{calcd.}$ [g/cm^3]	1.266	1.219	1.222
μ [mm^{-1}]	0.436	0.188	0.165
T [K]	123(2)	123(2)	123(2)
Measured reflections	4183	38256	237652
Independent reflections	4183	38256	20223
Reflections with $I > 2\sigma(I)$	3086	30089	11936
R_{int}	0.1069	0.0616	0.1683
F(000)	908	2040	2699
$R_1(R[F^2>2\sigma(F^2)])$	0.0490	0.0546	0.0555
w$R_2(F^2)$	0.1031	0.1495	0.1404
GooF	1.048	1.026	1.024
No. of Parameters	244	1273	1032
CCDC #	2251389	2251390	2251391

Compound	**1EMind**	**6EMind**
Chem. Formula	$C_{54}H_{76}N_2P_4 \cdot 4(C_6H_6)$	$C_{54}H_{78}N_2P_4 \cdot C_6H_4Cl_2$
Formula weight [g/mol]	1189.47	1026.05
Color	green	yellow
Crystal system	triclinic	triclinic
Space group	$P\bar{1}$	$P\bar{1}$
a [Å]	9.6149(6)	10.7480(4)
b [Å]	11.8393(7)	13.9965(5)
c [Å]	16.5611(11)	19.5174(8)
α [°]	101.581(2)	77.8180(10)
β [°]	95.953(2)	80.874(2)
γ [°]	107.090(2)	87.811(2)
V [Å^3]	1738.43(19)	2833.61(19)
Z	1	2
$\rho_{calcd.}$ [g/cm^3]	1.136	1.203
μ [mm^{-1}]	0.152	0.267
T [K]	123(2)	173(2)
Measured reflections	90830	217722
Independent reflections	8392	18023
Reflections with $I > 2\sigma(I)$	5626	14015
R_{int}	0.1097	0.0518
$F(000)$	642	1100
$R_1(R[F^2>2\sigma(F^2)])$	0.0483	0.0471
$wR_2(F^2)$	0.1328	0.1547
GooF	1.035	1.057
No. of Parameters	400	708
CCDC #	2251392	2251393

Figure S1: Molecular structure of $EMindNH_2$. Ellipsoids are set at 50% probability (123 K). Selected bond lengths [Å] and angles [°]: N1–C1 1.403(2), C1–C2 1.404(2), C2–C3 1.398(2), C3–C4 1.389(2), C2–C7 1.531(2), C7–C8 1.551(2), C8–C9 1.547(2), C3–C9 1.523(2), C1–C2–C3 120.5(2), C1–C2–C7 128.5(2), C3–C2–C7 111.0(1), C2–C7–C8 101.4(6), C7–C8–C9 108.8(1), C3–C9–C8 101.2(1), C1–C2–C3–C9 −178.9(2).

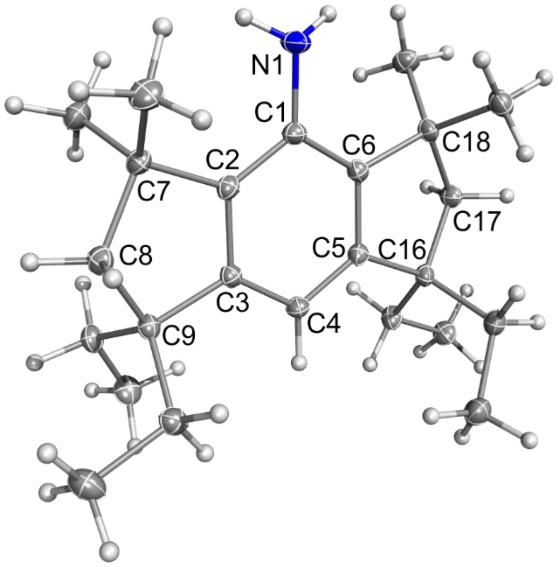

Figure S2: Molecular structure of **3Ter**. Ellipsoids are set at 50% probability (203 K). Selected bond lengths [Å] and angles [°]: P1–Cl1 2.102(1), P1–P2 2.964(1), P1–C1 1.821(2), P2–Cl2 2.084(1), P2–C2 1.821(2), N1–P1–P2–C2 −160.6(2), symmetry code: (') = 1−*x*, 1−*y*, 1−*z*.

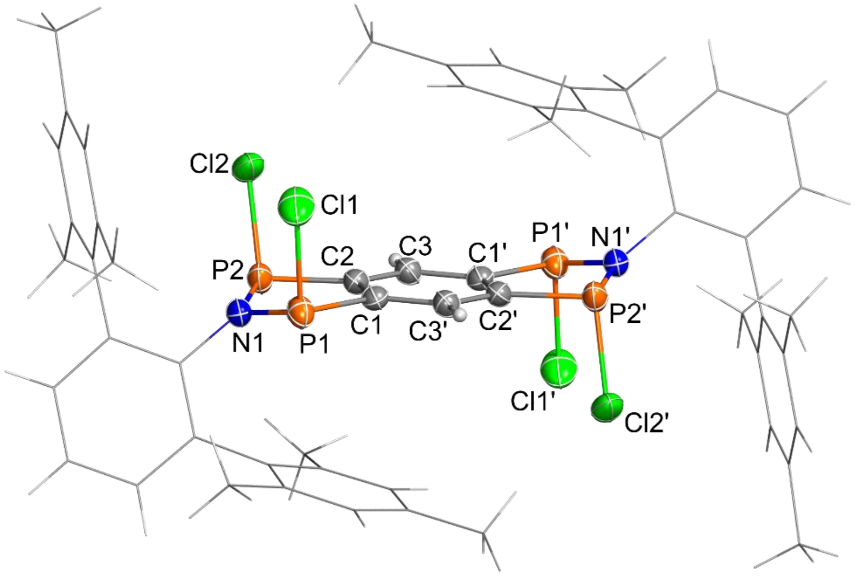

Figure S3: Molecular structure of **3EMind**. Ellipsoids are set at 50% probability (203 K). Selected bond lengths [Å] and angles [°]: P4–Cl4 2.0981(8), P3–Cl3 2.1089(7), P2–Cl2 2.1066(7), P1–Cl1 2.0954(7), P4–C4 1.828(2), P3–C3 1.822(2), P2–C6 1.820(2), P1–C1 1.831(2), P3–P4 2.9733(7), P1–P2 2.9642(7), P1–N1–P2 119.57(7), P3–N2–P4 120.32(7), N1–P1–P2–C6 −159.4(1), C3–P3–P4–N2 −164.3(1).

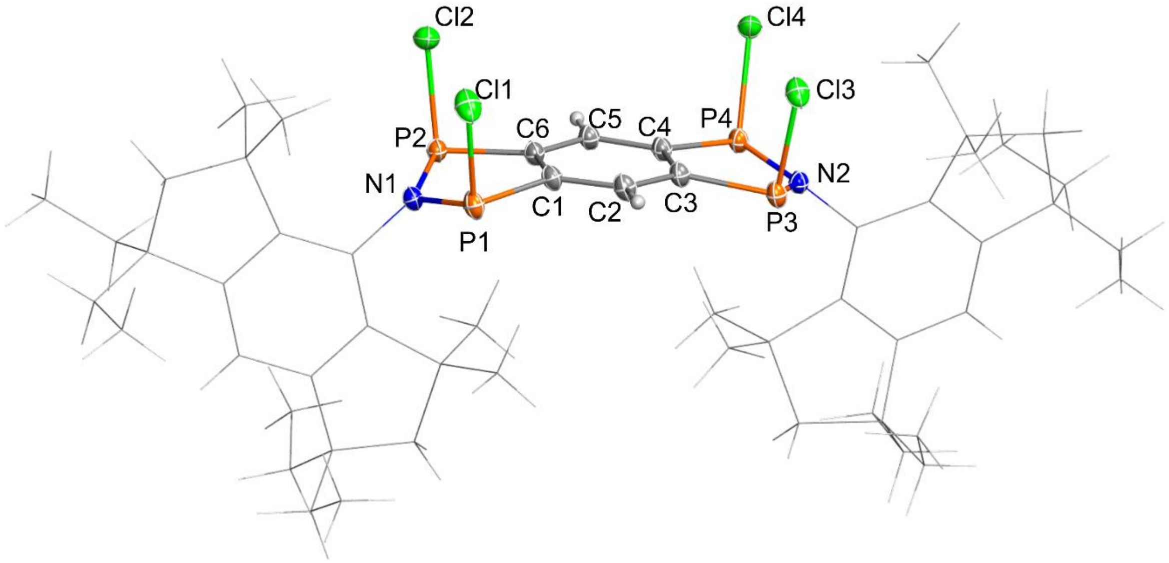

Figure S4: Molecular structure of **3Mes***. Ellipsoids are set at 50% probability (203 K). Selected bond lengths [Å] and angles [°]: P1–Cl1 2.097(2), P1–P2 2.969(1), P1–C1 1.822(3), P2–Cl2 2.094(1), P2–C2 1.823(3), N1– P1–P2–C2 170.5 (2), symmetry code: *i* = 1−*x*, 1−*y*, 1−*z*.

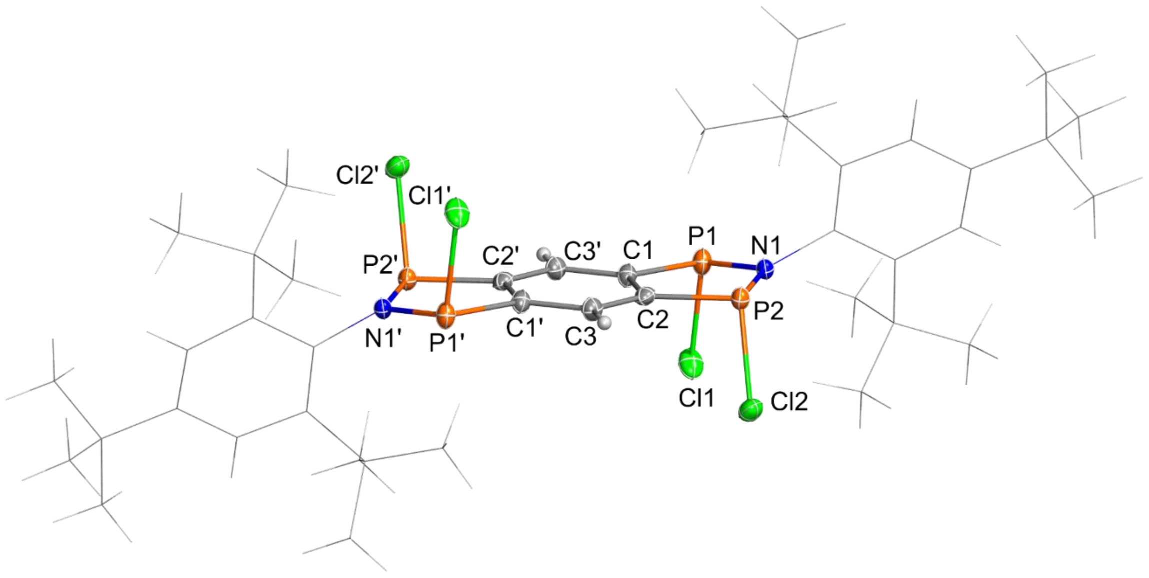

Figure S5: Molecular structure of **4Ter**. Ellipsoids are set at 50% probability (123 K). Selected bond lengths [Å] and angles [°]: P1–C1 1.826(3), P1–C10 1.945(3), P2–C2 1.811(3), P2–P5 2.3364(9), P3–C4 1.711(3), P4–C5 1.712(3), P7–C8 1.703(3), P8–C9 1.714(3), P5–C11 1.802(3), P6–C12 1.817(3), P6–C3 1.942(3), C1–C2 1.348(4), C1–C6 1.515(4), C2–C3 1.501(4), C3–C4 1.511(4), C4–C5 1.397(4), C5–C6 1.509(4), C6–C7 1.625(3), C7–C8 1.502(4), C7–C12 1.513(4), C8–C9 1.405(4), C9–C10 1.507(4), C10–C11 1.506(4), C11–C12 1.356(4), P1–N1–P2 116.4(1), P3–N2–P4 119.4(2), P5–N3–P6 116.1(2), P7–N4–P8 118.9(2), N3–P6–P5–C11 155.2(2), P5–C11–C12–C7 158.5(2), C12–C7–C10–C9 144.4(3), C10–C9–C8–P7 178.1(3), C8–P7–P8–N4 −176.8(3).

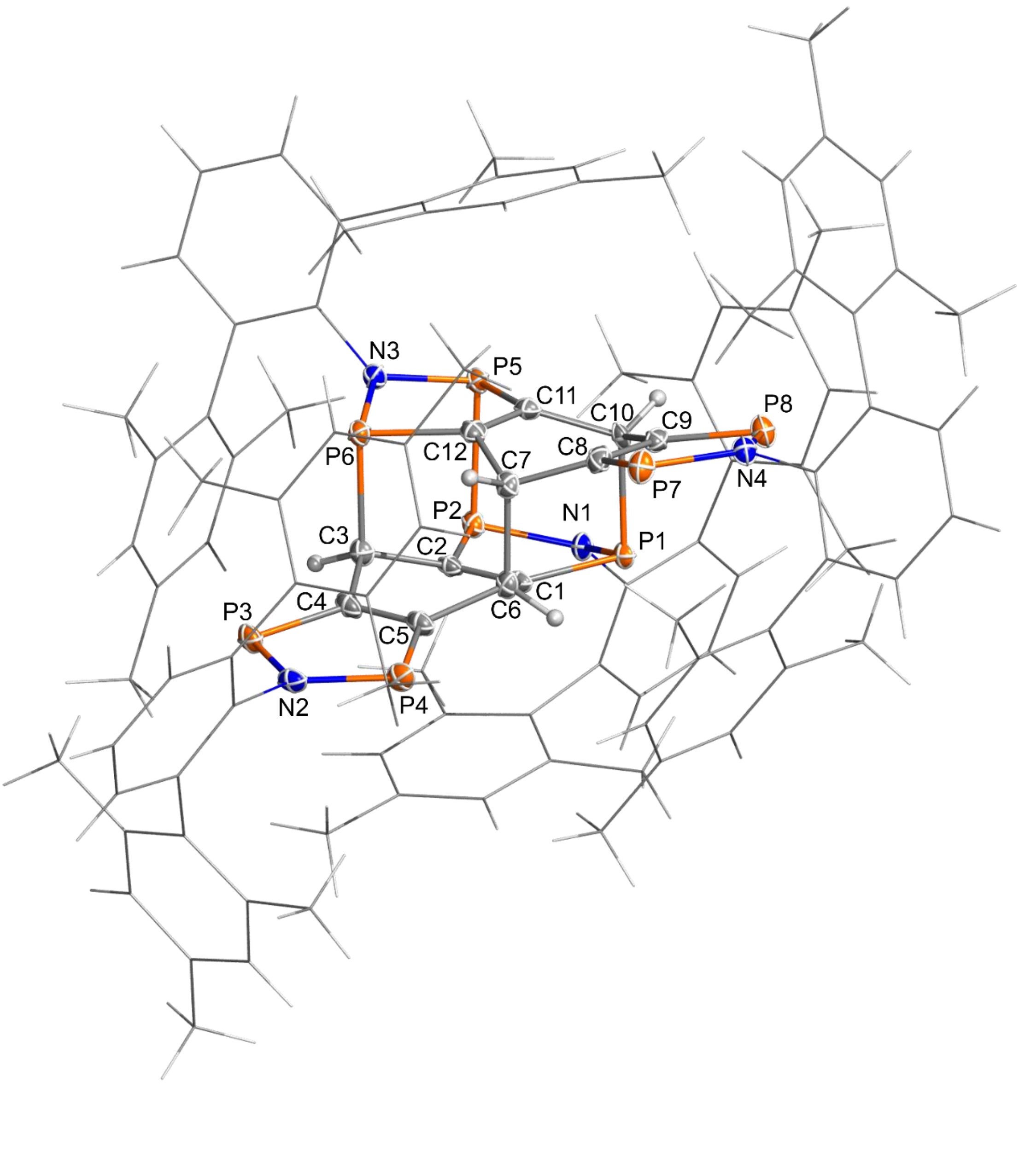

Figure S6: Molecular structure of **5Ter**. Ellipsoids are set at 50% probability (123 K). Selected bond lengths [Å] and angles [°]: P1–C1 1.857(2), P1–C7 1.885(2), P2–C2 1.845(2), P1–P2 2.7877(7), P2–C8 1.879(2), P3–C4 1.865(2), P3–C22 1.878(2), P3–P4 2.7939(7), P4–C5 1.850(2), P4–C21 1.870(2), C22–C21 1.347(3), C8–C7 1.344(3), P1–C7–C8 111.1(2), P2–C8–C7 114.0(2), P3–C22–C21 111.4(1), P4–C21–C22 114.0(2), C2–P2–P1–N1 −127.4(2), C4–P3–P4–N1 127.7(2).

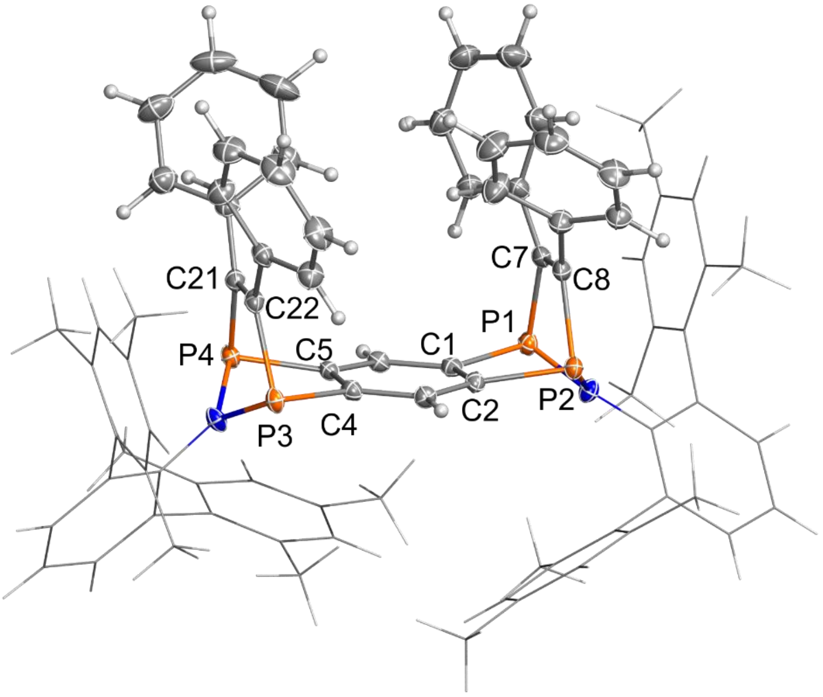

Figure S7: Molecular structure of **1EMind**. Ellipsoids are set at 50% probability (123 K). Selected bond lengths [Å] and angles [°]: P2–P1 2.9702(9), P2–C2 1.751(2), P1–C1 1.752(2), N1–P1 1.694(2), N1–P2 1.698(2), C1–C2 1.449(3), C2–C3 1.400(3), C3'–C1 1.400(3), P1–N1–P2 122.3(1), C1–P1–N1 93.14(9), C2–P2–N1 93.11(9), C2–C1–P1 115.8(1), C1–C2–P2 115.7(1), N1–P1–P2–C2 −179.9(1), P1–C1–C2–C3 179.8(2). Symmetry code ('): (1-x, 1-y, 2-z).

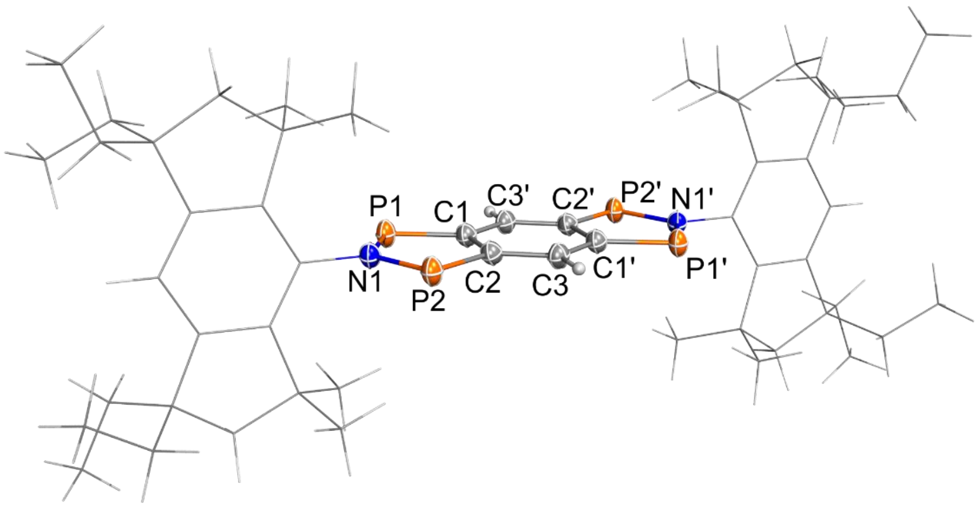

Figure S8: Molecular structure of **6EMind**. Ellipsoids are set at 50% probability (123 K). Selected bond lengths [Å] and angles [°]: P4–P3 2.9566(6), P2–P1 2.9350(6), P4–C4 1.739(1), P3–C3 1.737(1), C6–P2 1.832(1), C1–P1 1.825(1), N2–P4 1.698(1), N2–P3 1.698(1), P2–N1 1.726(1), P1–N1 1.717(1), C3–C4 1.437(2), C1–C6 1.440(2), P4–N2–P3 121.05(7), C4–P4–N2 93.62(6), C3–P3–N2 93.48(6), P4–C4–C3 115.7(1), C4–C3–P3 116.1(1), P2–C6–C1 113.7(1), C6–C1–P1 114.5(1), P1–N1–P2 116.97(7), N2–P4–P3–C3 179.6(1), C6–P2–P1–N1 −148.86(9).

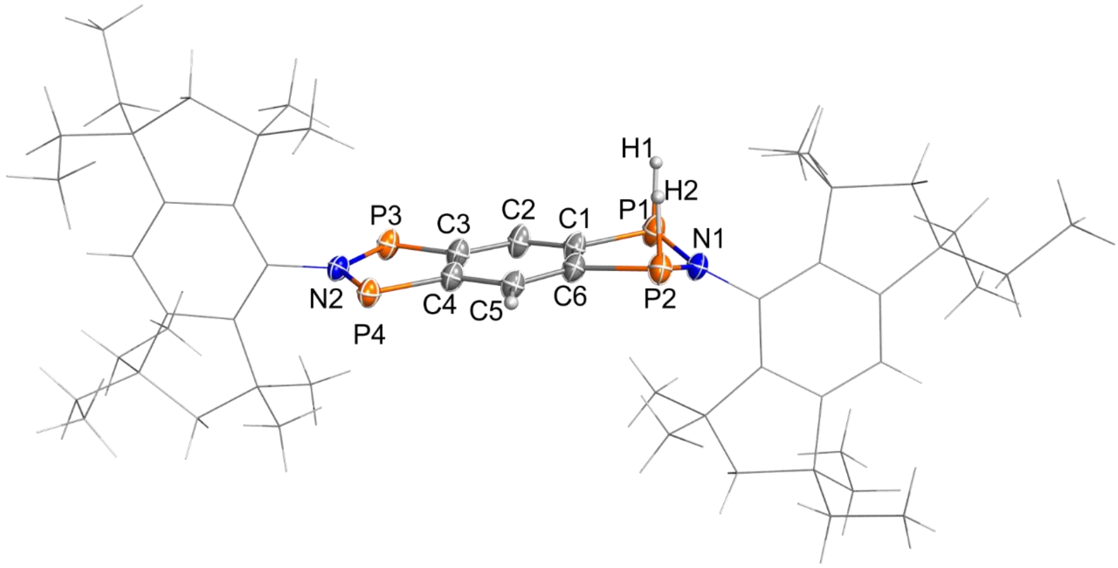

3 Syntheses of starting materials

3.1 TerI

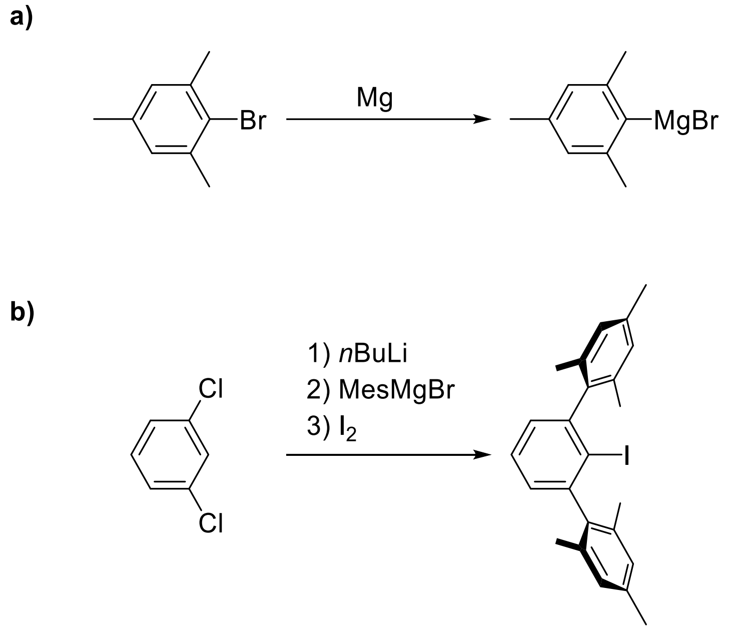

TerI was synthesized according to optimized, previously reported literature procedures.[3,11] The synthesis began with the preparation of a MesMgBr solution, which was used in the further synthesis at the same day.

a) Mg tunings (12.31 g, 506 mmol) were suspended in THF (150 mL) in a 1 L three-necked flask equipped with a long reflux condenser (length cooling coil: 40 cm) and a pressure relief valve. A solution of MesBr (91.61 g, 460.1 mmol) in THF (250 mL) was slowly added at ambient temperature *via* dropping funnel, until a color change to brown and warming of the reaction mixture was observed. At that time, an ice bath was placed under the flask and the remaining MesBr solution was added over a period of 30 min. The ice bath was removed and the Grignard solution was carefully warmed to ambient temperature. The reaction mixture was refluxed for 2.5 h (heating mantle) and was subsequently cooled to room temperature. Deviation of the reflux time of +1 h

had no effect on the further reaction steps and were sometimes necessary due to delays in the lithiation of 1,3-dichlorobenzene which was carried out in the meantime:

b) *This reaction part was performed parallel to reaction part **a)** (above) and was started when the Grignard solution began to reflux.* 1,3-dichlorobenzene (26.56 g, 180.7 mmol) was dissolved in THF (600 mL) and cooled down to −80 °C. *n*BuLi (73 mL, 2.5M in *n*-hexane, 182.5 mmol) was added over a period of 30 min. The reaction mixture was stirred for 10 min at −80 °C and then the Grignard solution from part **a)** was added *via* cannula transfer over a period of 20 min at −80 °C. The reaction was warmed to ambient temperature overnight under stirring. On the next day, the reaction mixture was refluxed for 2 h (heating mantle) and subsequently cooled down to 0 °C (ice bath). I_2 (84.56 g, 333.2 mol) was added portionwise until the reaction mixture stayed consistently brown (for approx. 15 min). The workup proceeded under non-inert conditions. Therefore the reaction was quenched by adding a solution of Na_2SO_3 (37.73 g, 299.3 mmol) in water (300 mL). The aqueous phase was separated and extracted 3 times with Et_2O (200 mL each). The organic phases were combined and all volatile compounds were removed using a rotary evaporator. The residue was placed in a funnel filter with sintered glass disc and was washed with cold EtOH (−110 °C) until the product remained as colorless solid. The product was dried *in vacuo* (1×10^{-3} mbar) at 50 °C (water bath) for 2 h. Yield: 56.07 g (127.3 mmol, 70%).

Mp. 228 °C (lit. 225-226 °C)[3,11]. **CHN** calc. (found) in %: C 65.46 (65.68), H 5.72 (5.85). **^{1}H NMR** (CD_2Cl_2, 250.1 MHz): δ = 1.97 (s, 12 H, *o*-C*H*$_3$), 2.34 (s, 6 H, *p*-C*H*$_3$), 6.95 (s, 4 H, C*H* (Mes)), 7.48 (m, 1 H, *p*-C*H* (Ter)), 7.57 (m, 2 H, *m*-C*H* (Ter)). **^{13}C{^{1}H} NMR** (CD_2Cl_2, 62.9 MHz): 20.5 (s, *o*-CH_3), 21.5 (s, *p*-CH_3), 108.0 (s, $C_{quart.}$), 128.5 (s, CH), 128.4 (s, CH), 129.5 (s, CH), 135.9 (s, $C_{quart.}$), 137.7 (s, $C_{quart.}$), 142.7 (s, $C_{quart.}$), 147.7 (s, $C_{quart.}$). **IR** (ATR, 32 scans, cm^{-1}): $\tilde{\nu}$ = 420 (w), 546 (m), 573 (s), 583 (m), 701 (m), 738 (vs), 777 (m), 800 (s), 849 (s), 1001 (m), 1012 (m), 1030 (w), 1094 (w), 1179 (w), 1265 (w), 1377 (m), 1447 (m), 1574 (w), 1611 (w), 1733 (vw), 1875 (vw), 1943 (vw), 2728 (w), 2850 (w), 2912 (m), 2943 (w), 2965 (w). **Raman** (633 nm, 10 s, 20 scans, cm^{-1}): $\tilde{\nu}$ = 133 (10), 163 (3), 225 (2), 241 (2), 261 (2), 267 (2), 285 (6), 337 (2), 422 (2), 465 (1), 509 (1), 523 (3), 560 (7), 574

(6), 584 (2), 705 (1), 739 (2), 801 (1), 853 (1), 944 (1), 1003 (2), 1014 (1), 1094 (1), 1099 (1), 1167 (1), 1181 (1), 1267 (1), 1287 (1), 1306 (6), 1379 (2), 1384 (3), 1437 (1), 1485 (1), 1561 (1), 1571 (2), 1576 (2), 1613 (3), 2730 (2), 2856 (3), 2914 (6), 3016 (4), 3045 (3). **MS** (EI, 70 eV, m/z, rel. int. > 10%): 441 (12) $[M]^+$, 440 (52) $[M]^+$, 312 (44) $[M-HI]^+$, 298 (29) $[M-MeI]^+$.

Figure S9: NMR, IR and Raman spectra of TerI (solvent signals indicated by asterisks).

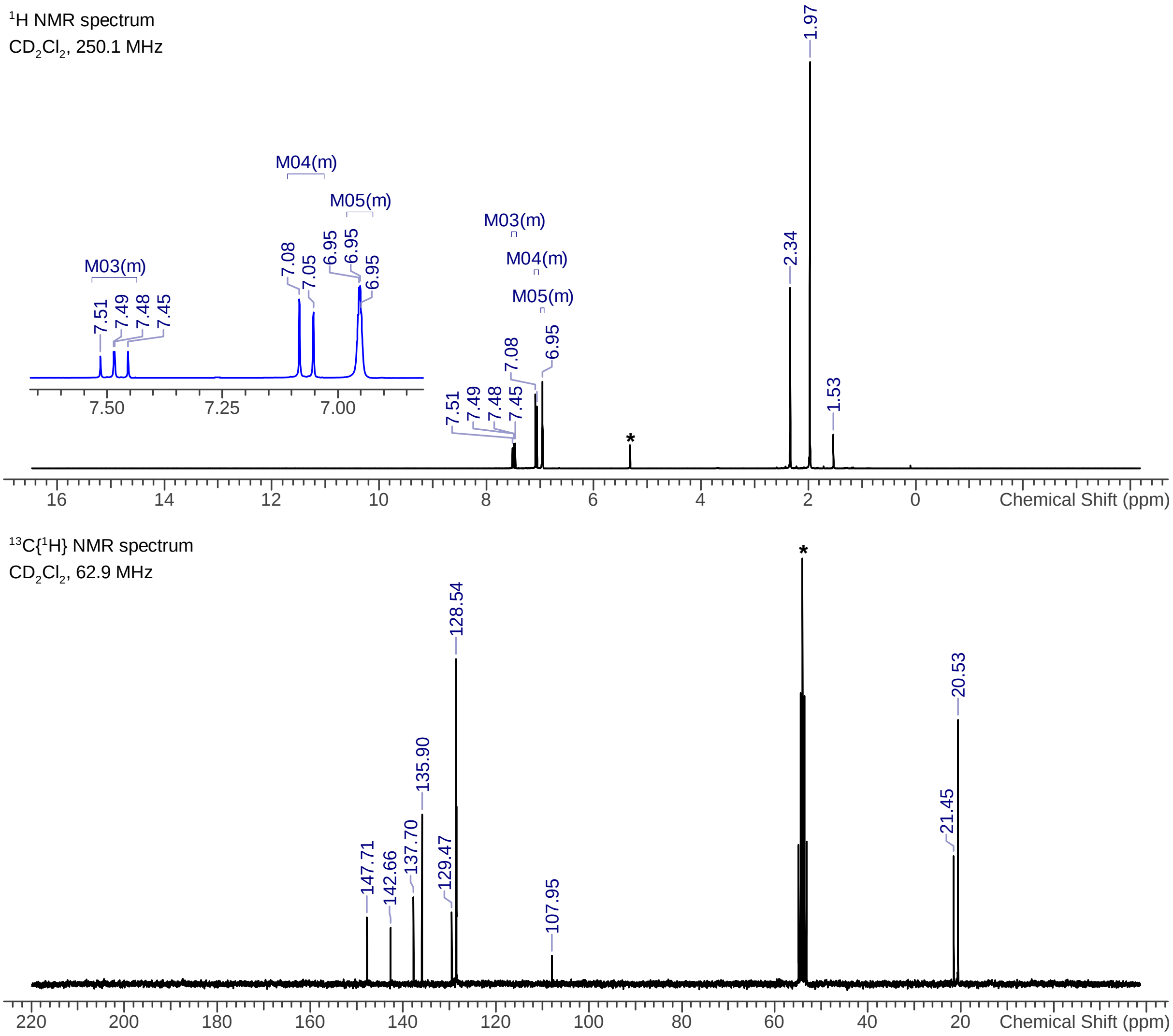

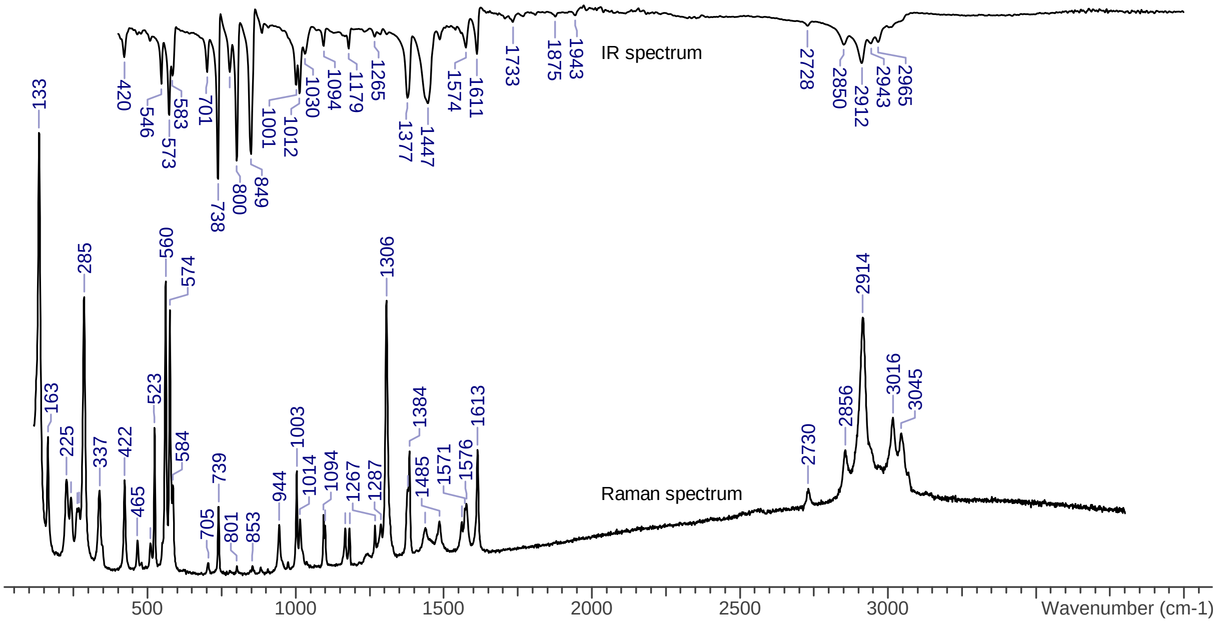
IR spectrum
420
546
573
583
701
738
800
849
1001
1012
1030
1094
1179
1265
1377
1447
1574
1611
1733
1875
1943
2728
2850
2912
2943
2965
Raman spectrum
133
163
225
285
337
422
465
523
560
574
584
705
739
801
853
944
1003
1014
1094
1267
1287
1306
1384
1485
1571
1576
1613
2730
2856
2914
3016
3045
500
1000
1500
2000
2500
3000
Wavenumber (cm-1)

3.2 $TerNH_2$

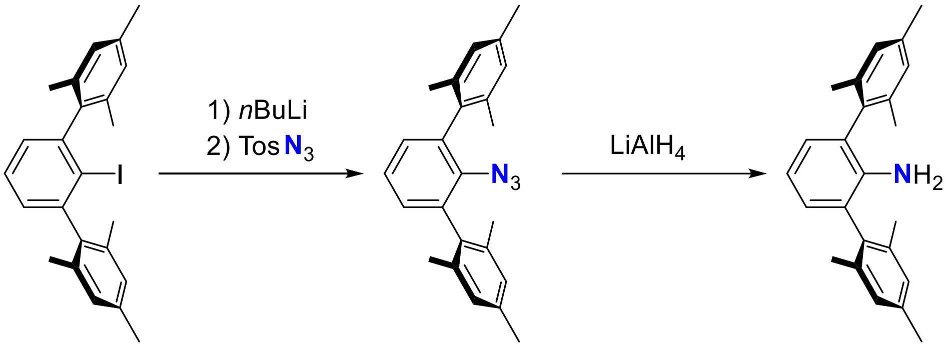

$TerNH_2$ was synthesized according to slightly modified literature procedures without isolation of the intermediately formed $TerN_3$.[3,11] To a suspension of TerI (43.29 g, 98.3 mmol) in Et_2O (500 mL) *n*BuLi (48 mL, 2.5M in *n*-hexane, 120 mmol) was added over a period of 15 min at 0 °C (ice bath). The reaction mixture was stirred for 30 min. Afterwards tosyl azide (20.00 g, 101.4 mmol, neat) was added over a period of 5 min. The reaction mixture was warmed to ambient temperature and was stirred for 3 h. The workup proceeded under non-inert conditions. Therefore, the reaction was quenched by adding an aqueous solution of NaOH (0.05 M, 200 mL). The organic phase was separated and washed with aqueous NaOH (0.05 M, 200 mL). The combined aqueous phases were extracted twice with Et_2O (200 mL each). All organic phases were combined and dried over $MgSO_4$. All volatile compounds were removed *in vacuo* (rotary evaporator).

The further reaction steps were done under inert conditions again. The remaining crude $TerN_3$ and $LiAlH_4$ (7.314 g, 193 mmol) were placed in a 1 L three-necked flask equipped with a reflux condenser and a pressure relief valve. The solids were cooled down to −80 °C and were stirred while cold (−80 °C) Et_2O (400 mL) was added rapidly. *Attention: The solvation of $LiAlH_4$ is extremely exothermic and cooling of the solvent therefore essential!* The reaction mixture was warmed to ambient temperature and was refluxed for 3 h (heating mantle) until no further evolution of N_2 was observed. The reaction mixture was cooled to 0 °C (ice bath) and was quenched with a minimal amount of water. The addition of water was stopped as soon as no more hydrogen was formed.

The further workup proceeded under non-inert conditions. Et_2O (400 mL) was added and solid by-products were removed by filtration. The filtrate was extracted with water (150 mL) to ensure that all $LiAlH_4$ was quenched. The organic phase was separated and dried with $MgSO_4$. Afterwards it was concentrated (rotary evaporator) and the product was crystallized from the solution overnight at 5 °C (fridge). The product was collected in two fractions of colorless crystals and was dried *in vacuo* (1×10^{-3} mbar) for 2 h at 50 °C (water bath). Yield: 22.68 g (51.51 mmol, 70%).

Mp. 144 °C (lit. 145-146)[3,11]. **CHN** calc. (found) in %: C 87.49 (86.68), H 8.26 (8.70), N 4.25 (3.65); deviations probably due to incomplete combustion, repeated measurements with and without oxidizing agents did not result in better agreement **^{1}H NMR** (CD_2Cl_2, 250.1 MHz): δ = 2.05 (s, 12 H, *o*-CH_3), 2.32 (s, 6 H, *p*-CH_3), 3.15 (s, 2 H, NH_2), 6.84-6.92 (superimposed signals, 3 H, *meta*- and *para*-C*H* (Ter)), 6.98 (m, 4 H, C*H* (Mes)). **^{13}C{^{1}H} NMR** (CD_2Cl_2, 62.9 MHz): δ = 20.4 (s, *o*-CH_3), 21.4 (s, *p*-CH_3), 118.5 (s, *C*H), 126.6 (s, $C_{quart.}$), 128.9 (s, *C*H), 129.2 (s, *C*H), 135.9 (s, $C_{quart.}$), 137.4 (s, $C_{quart.}$), 141.5 (s, $C_{quart.}$). **IR** (ATR, 32 scans, cm^{-1}): $\tilde{\nu}$ = 492 (m), 550 (m), 567 (s), 626 (m), 715 (m), 748 (vs), 773 (m), 795 (m), 832 (m), 847 (vs), 1006 (m), 1036 (m), 1072 (w), 1156 (w), 1216 (w), 1259 (m), 1304 (w), 1374 (m), 1447 (vs), 1488 (w), 1574 (w), 1601 (s), 1719 (vw), 2728 (w), 2854 (w), 2914 (m), 2945 (w), 2969 (w), 3013 (w), 3046 (w), 3382 (w), 3474 (w). **Raman** (633 nm, 10 s, 20 scans, cm^{-1}): $\tilde{\nu}$ = 167 (2), 172 (2), 176 (2), 191 (1), 209 (2), 226 (2), 278 (2), 331 (1), 394 (2), 477 (0), 494 (1), 512 (1), 521 (3), 529 (1), 554 (1), 568 (4), 578 (10), 631 (4), 745 (1), 835 (1), 846 (0), 945 (1), 1008 (2), 1075 (1), 1165 (1), 1218 (0), 1243 (0), 1260 (0), 1285 (1), 1304 (8), 1381 (3), 1438 (1), 1453 (1), 1487 (1), 1579 (1), 1612 (4), 2731 (1), 2858 (2), 2917 (6), 2945 (2), 2973 (2), 3018 (3), 3055 (2), 3382 (2), 3479 (1). **MS** (EI, 70 eV, m/z, rel. int. > 10%): 330 (33) $[M]^+$, 229 (100) $[M]^+$, 314 (40) $[M-Me]^+$, 299 (14) $[C_{22}H_{21}N]^+$.

Figure S10: NMR, IR and Raman spectra of $TerNH_2$ (solvent signals indicated by asterisks).

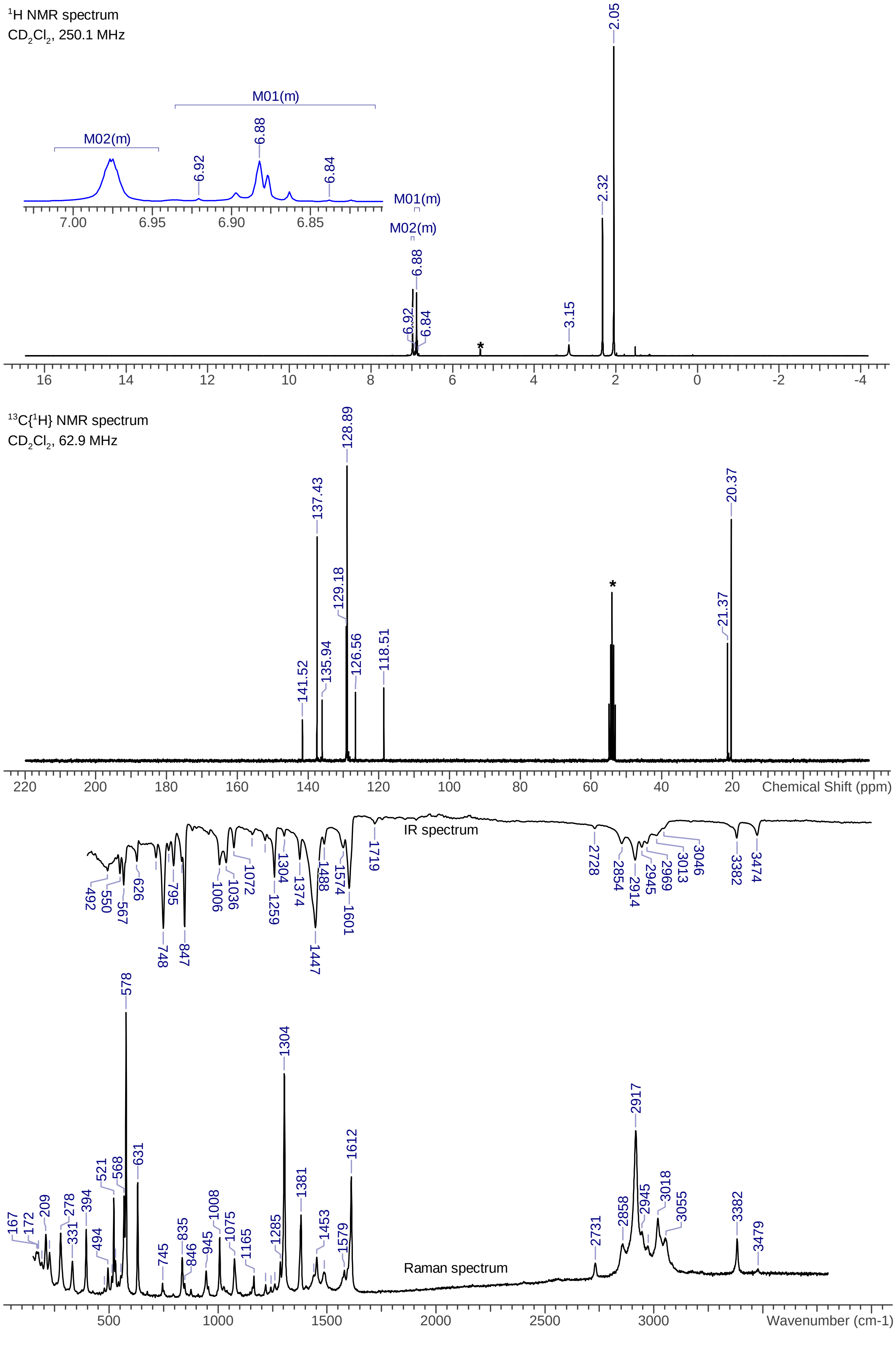

3.3 1,3-Bis(1-chloro-1-methylethyl)benzene

Procedure 1

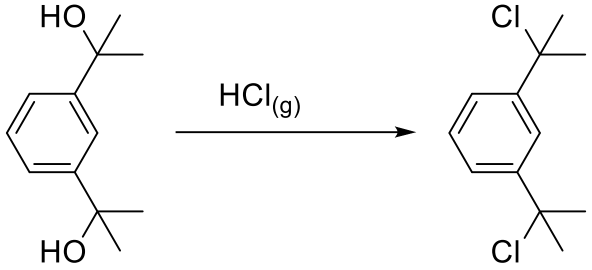

1,3-Bis(1-chloro-1-methylethyl)benzene was synthesized according to a slightly modified literature procedure.[12] The synthesis was done under non-inert conditions. Powdered $CaCl_2$ (~50 g) was suspended in CH_2Cl_2 (500 mL). To this suspension was added 1,3-bis(1-hydroxy-1-methylethyl)benzene (50.5 g, 260 mmol). Afterwards dry HCl gas was bubbled for through the suspension over a period of 2 h at 0 °C (ice bath). The reaction was checked for full conversion by ^{1}H NMR spectroscopy. The reaction mixture was purged with argon for 2 h to remove HCl. Afterwards $CaCl_2$ was removed by filtration. The filtrate was concentrated *in vacuo* until a yellowish oil remained. The oil was dried *in vacuo* (1×10^{-3} mbar) at 45 °C (water bath) for 45 min. The product was used without further purification (see impurities in ^{1}H NMR spectrum). Yield: 54.2 g (234 mmol, 90%).

Annotation: Compared to the second synthesis method (see below), the product synthesized with HCl is more viscous. This leads to a more difficult work-up in the synthesis of EMindH (p. S28ff.).

^{1}H NMR (CD_2Cl_2, 300.1 MHz): δ = 1.99 (s, 12 H, Me), 7.35 (t, $^{3}J(^{1}H, ^{1}H)$ =7.9 Hz, 1 H, *p*-C*H*), 7.51 (dd, $^{3}J(^{1}H, ^{1}H)$ =7.9 Hz, $^{4}J(^{1}H, ^{1}H)$ =2.0 Hz, 2 H, m-C*H*), 7.83 (t, $^{3}J(^{1}H, ^{1}H)$ =2.0 Hz, 1 H, *i*-C*H*).

Figure S11: ^{1}H NMR spectrum of 1,3-bis(1-chloro-1-methylethyl)benzene, synthesized with HCl (solvent signals indicated by asterisks).

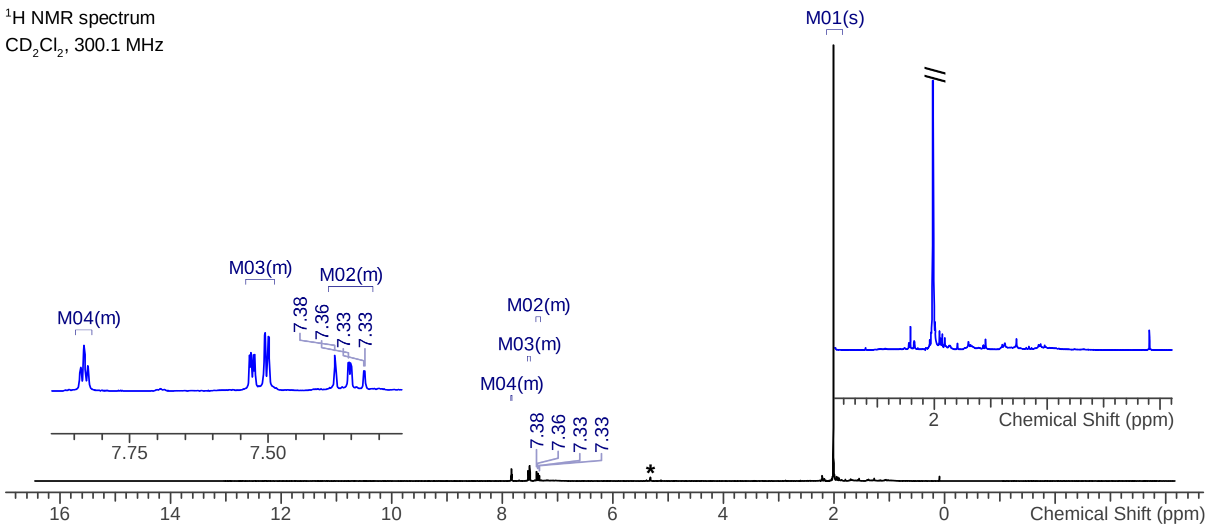

Procedure 2

Alternatively, the synthesis of 1,3-bis(1-chloro-1-methylethyl)benzene was carried out according to a modified literature procedure using TMSCl as chlorinating reagent:[13]

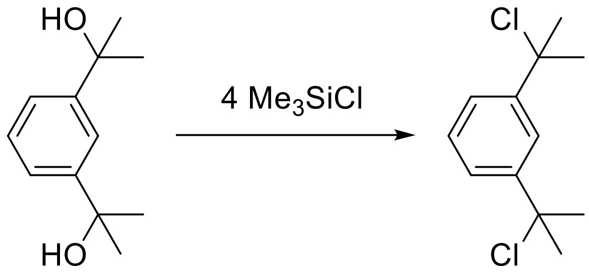

1,3-Bis(1-hydroxy-1-methylethyl)benzene (151.87 g, 781.73 mmol) was placed in a 1 L three-necked flask equipped with a pressure relief valve. TMSCl (434 mL, 3420 mmol, neat) was added over a period of 10 min at ambient temperature. HCl gas evolution was observed within the first 30 min after the addition of TMSCl. The reaction mixture was stirred overnight, resulting in a colorless two-phase system. Volatile compounds were removed *in vacuo* (1×10^{-3} mbar). Further workup proceeded under non-inert conditions. The residual oil was dissolved in *n*-pentane (720 mL) and was first washed with a mixture of brine (200 mL) and water (200 mL) and afterwards with water only (200 mL) to remove residual HCl. The organic phase was separated and dried over Mg_2SO_4. Volatile compounds were removed *in vacuo* (1×10^{-3} mbar) and the residual

colorless oil was further dried for 2 h at 50 °C (water bath). Yield: 175.35 g (758.57 mmol, 97%).

CHN calc. (found) in %: C 62.35 (62.31), H 6.98 (6.47). **^{1}H NMR** (CD_2Cl_2, 500.1 MHz): δ = 2.00 (s, 12 H, Me), 7.35 (t, $^3J(^1H, ^1H)$ = 7.9 Hz, 1 H, *p*-C*H*), 7.50 (dd, $^3J(^1H, ^1H)$ = 7.9 Hz, $^4J(^1H, ^1H)$ =2.0 Hz, 2 H, *m*-C*H*), 7.83 (t, $^3J(^1H, ^1H)$ =2.0 Hz, 1 H, *i*-C*H*). **^{13}C{^{1}H} NMR** ($CDCl_3$, 125.8 MHz): δ = 34.6 (s, CH_3), 70.4 (s, *C*(Cl)Me_2), 123.4 (s, *C*H), 125.3 (s, *C*H), 128.7 (s, *C*H), 146.9 (s, aromatic $C_{quart.}$). **IR** (ATR, 32 scans, cm^{-1}): $\tilde{\nu}$ = 406 (w), 416 (w), 437 (w), 486 (w), 515 (m), 610 (s), 649 (w), 699 (vs), 793 (s), 820 (w), 890 (w), 915 (w), 938 (w), 1008 (vw), 1045 (vw), 1082 (m), 1105 (m), 1123 (m), 1170 (vw), 1185 (vw), 1234 (m), 1276 (w), 1368 (m), 1385 (m), 1420 (w), 1457 (m), 1488 (w), 1585 (vw), 1601 (w), 1704 (vw), 1881 (vw), 1949 (vw), 2868 (vw), 2928 (w), 2978 (m), 3027 (vw), 3070 (vw). **Raman** (633 nm, 20 s, 20 scans, cm^{-1}): $\tilde{\nu}$ = 69 (2), 78 (4), 110 (6), 125 (7), 213 (2), 249 (1), 286 (1), 356 (3), 439 (1), 516 (2), 529 (4), 598 (2), 620 (2), 652 (4), 710 (1), 818 (1), 907 (1), 914 (1), 937 (1), 1001 (10), 1083 (1), 1107 (1), 1135 (2), 1172 (1), 1185 (1), 1278 (2), 1387 (1), 1442 (1), 1457 (1), 1586 (1), 1604 (2), 1630 (1), 2724 (1), 2765 (1), 2860 (1), 2936 (4), 2980 (3), 2990 (3), 3047 (1), 3071 (2). **MS** (EI, 70 eV, m/z, rel. int. > 10%): 234 (24), 233 (100), 232 (56), 205 (22) $[M-MeI]^+$, 155 (12) $[M-C_3H_5Cl]^+$, 100 (10) $[C_{10}H_{14}Cl_2]^+$.

Figure S12: NMR, IR and Raman spectra of 1,3-bis(1-chloro-1-methylethyl)benzene (solvent signals indicated by asterisks).

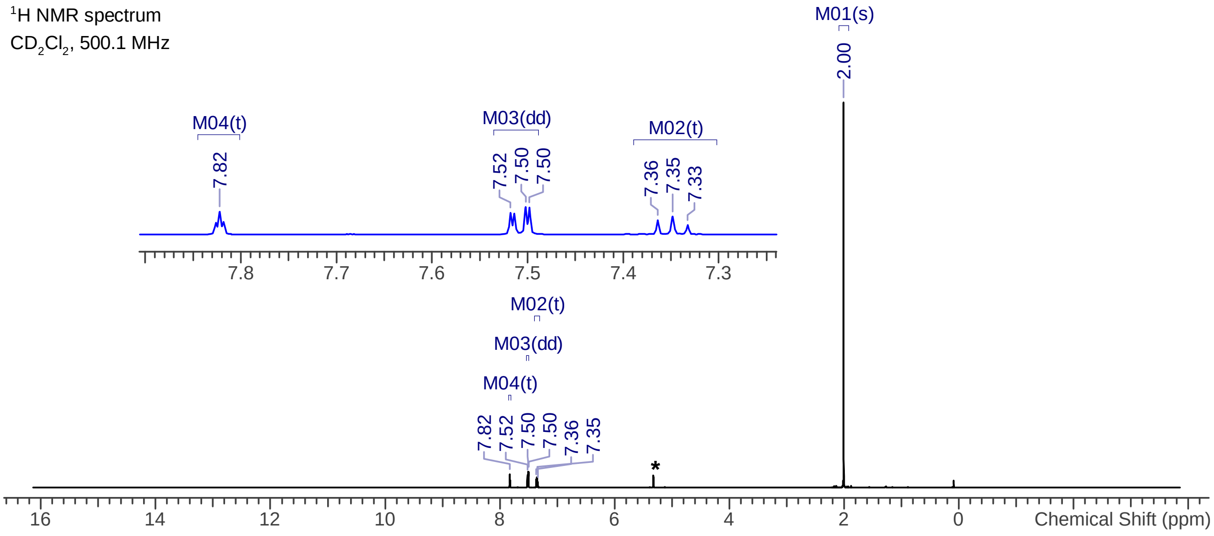

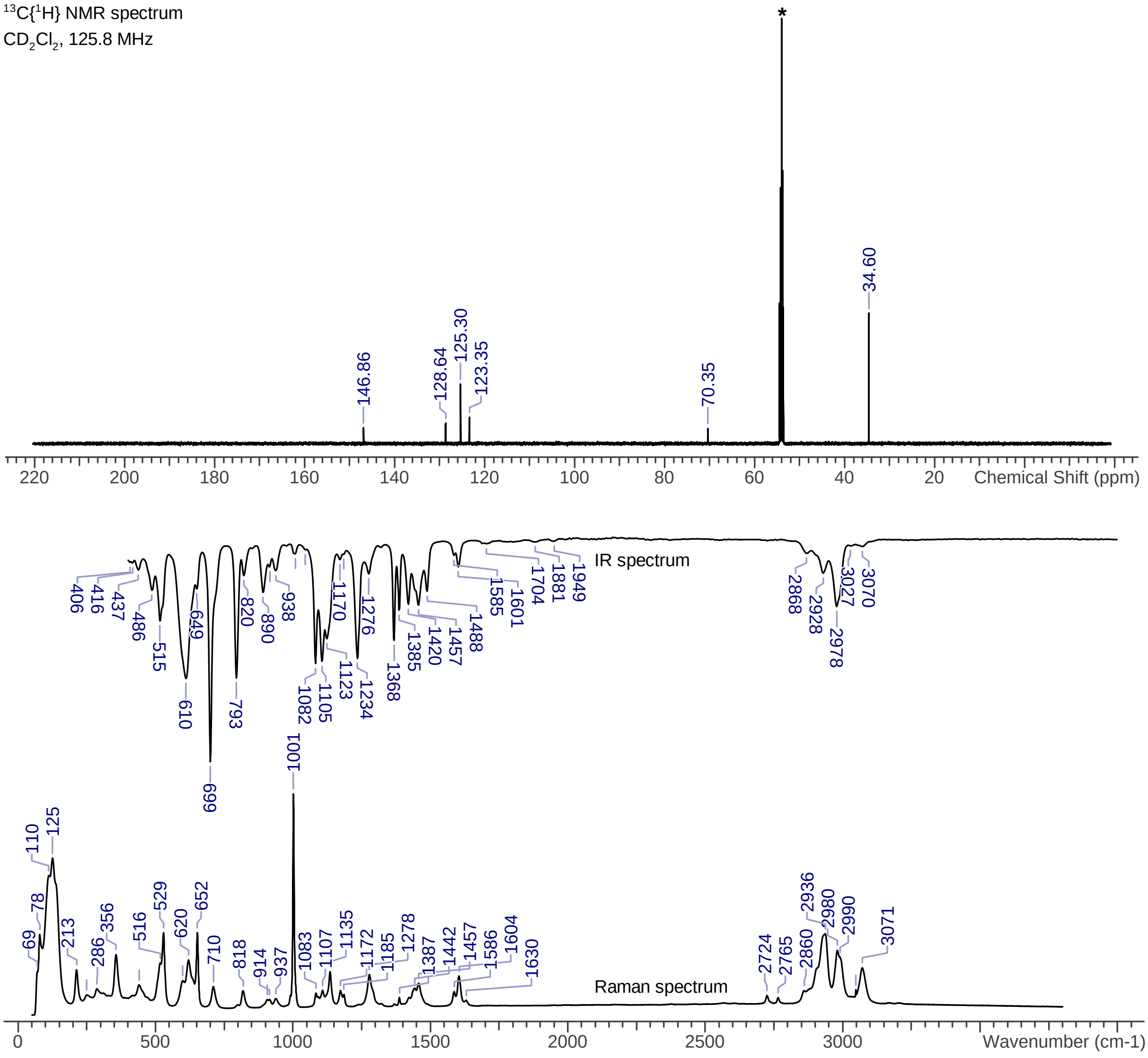
13C{1H} NMR spectrum
CD2Cl2, 125.8 MHz
146.86
128.64
125.30
123.35
70.35
34.60
Chemical Shift (ppm)
IR spectrum
Raman spectrum
Wavenumber (cm-1)

3.4 EMindH

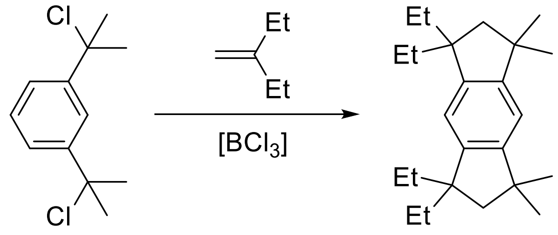

EMindH was synthesized according to modified literature procedures[13,14] 1,3-Bis(1-chloro-1-methylethyl)benzene (129.48 g, 560.13 mmol) and 2-ethyl-1-butene (106.92 g, 1270.4 mmol) were dissolved in CH_2Cl_2 (800 mL) in a 2 L three-necked flask equipped with a pressure relief valve. The reaction mixture was cooled to −80 °C (*i*-PrOH/N_2 bath) and BCl_3 (1.0 M in hexane, 50 mL, 50 mmol) was added via dropping funnel over a period of 20 min. The reaction mixture was stirred for 2 h at −80 °C and was afterwards warmed to ambient temperature overnight. The workup was done under non-inert conditions. The reaction was cooled to 0 °C (ice bath) and was quenched with a saturated aqueous solution of $NaHCO_3$ (700 mL). *Caution: CO_2 evolution*. The organic phase was separated and the aqueous phase was extracted with CH_2Cl_2 (150 mL). The combined organic fractions were dried over $MgSO_4$, which was removed by filtration afterwards. The filtrate was concentrated *in vacuo* until a yellowish oil remained. To purify the crude product, the residue was first dissolved in *n-hexane* which was then carefully placed on funnel filter (⌀ ≈ 5.5 cm) with a sintered glass and a plug of silica (height ≈ 6 cm) and was than filtered while *n*-hexane (450 mL) was continuously added. This acts as a lazy man's cullum . The filtrate was concentrated *in vacuo* (approx. 50 mL) and *i*-PrOH (200 mL) was added. The solution was cooled to 5 °C overnight yielding colorless crystals of EMindH. The product was separated by filtration and was washed with cold (−80 °C) *i*-PrOH (200 mL). The filtrate and washing solution were combined and were concentrated *in vacuo* (approx. 255 mL) The solution was cooled to 5 °C overnight to obtain a further crop of product. By further concentration of the supernatant a third crop of product could be crystallized. *If the product does not crystallize, either the temperature can be further decreased (−30 °C) or*

the i-PrOH content can be increased. The use of seed crystals is also very helpful. Yield: 113.94 g (348.91 mmol, 62 %).

Mp. 81.0 °C. (lit. 63-65 °C)[14] **CHN** calc. (found) in %: C 88.27 (87.37), H 11.73 (11.39). **^{1}H NMR** (CD_2Cl_2, 300.1 MHz): δ = 0.79 (dd, $^3J(^1H,^1H)$ = 7.6 Hz, $^3J(^1H,^1H)$ = 7.3 Hz, 12 H, CH_3 (Et)), 1.29 (s, 12 H, CH_3 (Me)), 1.59 (dq, $^2J(^1H,^1H)$ = 13.7 Hz, $^3J(^1H,^1H)$=7.3 Hz, 4 H, C(*H*)H (Et)), 1.65 (dq, $^2J(^1H,^1H)$ = 13.7 Hz, $^3J(^1H,^1H)$=7.6 Hz, 4 H, C(H)*H* (Et)), 1.87 (s, 4 H, CH_2 (cyclopent.)), 6.66 (s,1 H, C*H*), 6.82 (s, 1 H, C*H*). **$^{13}C\{^1H\}$ NMR** (CD_2Cl_2, 75.5 MHz): δ = 9.6 (s, CH_3), 32.5 (s, CH_3), 33.7 (s, CH_2 (Et)), 42.2 (s, $C_{quart.}$), 50.2 (s, $C_{quart.}$), 51.7 (s, CH_2 (cyclopent.)), 116.6 (s, CH), 119.3 (s, CH), 147.2 (s, aromatic $C_{quart.}$), 151.8 (s, aromatic $C_{quart.}$). **IR** (ATR, 32 scans, cm^{-1}): $\tilde{\nu}$ = 511 (m), 760 (w), 785 (m), 795 (w), 882 (m), 938 (w), 954 (w), 983 (w), 1008 (w), 1032 (w), 1076 (w), 1098 (w), 1175 (w), 1222 (m), 1276 (m), 1288 (m), 1300 (w), 1321 (m), 1356 (m), 1377 (m), 1414 (w), 1457 (s), 1758 (vw), 2856 (s), 2875 (s), 2922 (vs), 2930 (vs), 2955 (vs), 3002 (w). **Raman** (633 nm, 10 s, 10 scans, cm^{-1}): $\tilde{\nu}$ = 179 (1), 210 (1), 241 (1), 306 (2), 326 (1), 347 (1), 549 (1), 586 (1), 726 (10), 852 (1), 927 (2), 934 (1), 1031 (1), 1061 (1), 1074 (1), 1089 (1), 1126 (1), 1255 (1), 1304 (1), 1335 (1), 1379 (1), 1440 (2), 1461 (2), 2705 (1), 2732 (1), 2746 (1), 2857 (2), 2875 (2), 2897 (2), 2913 (2), 2927 (2), 2932 (2), 2966 (2), 3024 (2). **MS** (EI, 70 eV, m/z, rel. int. > 10%): 298 (45) $[M-Et]^+$, 297 (100) $[M-Et]^+$, 213 $[C_{16}H_{21}]^+$.

Figure S13: NMR, IR and Raman spectra of EMindH (solvent signals indicated by asterisks).

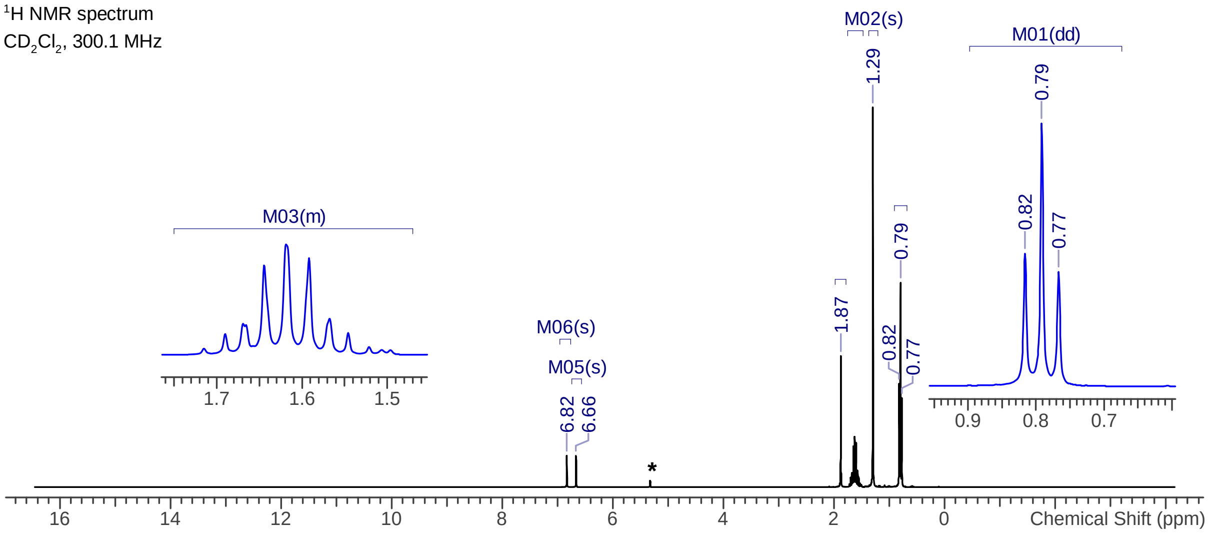

experimental and simulated ^{1}H NMR spectrum
CD_2Cl_2, 300.1 MHz

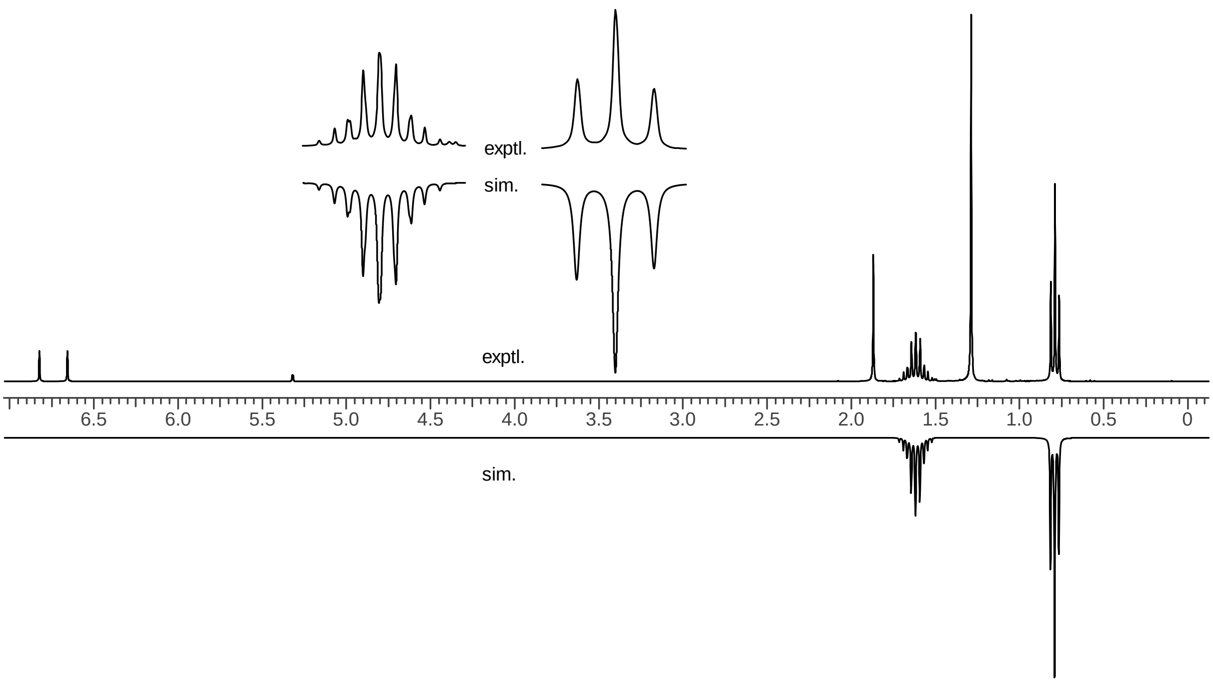

$^{13}C\{^{1}H\}$ NMR spectrum
CD_2Cl_2, 300.1 MHz

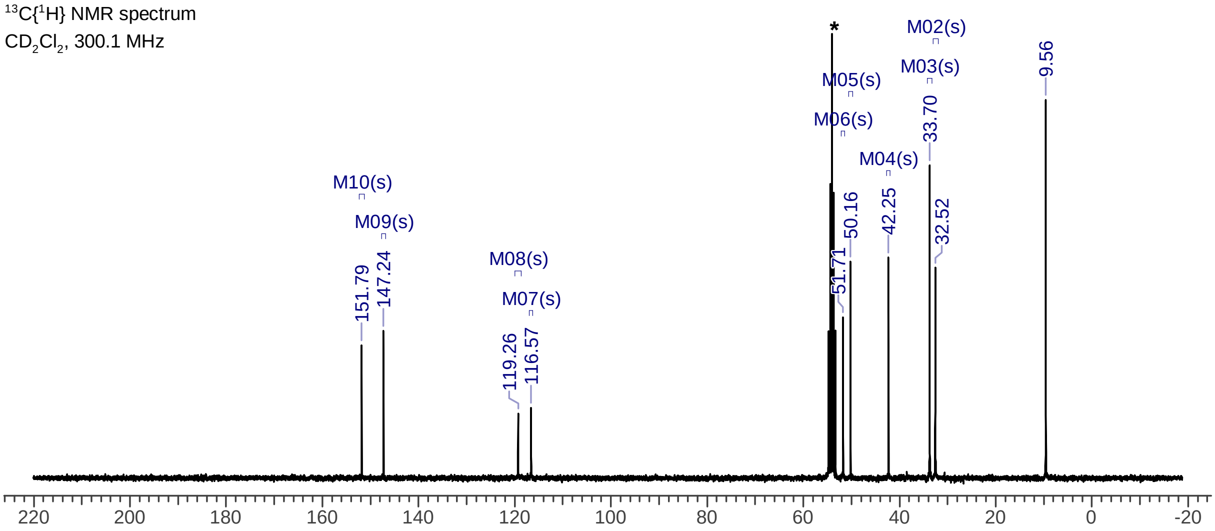

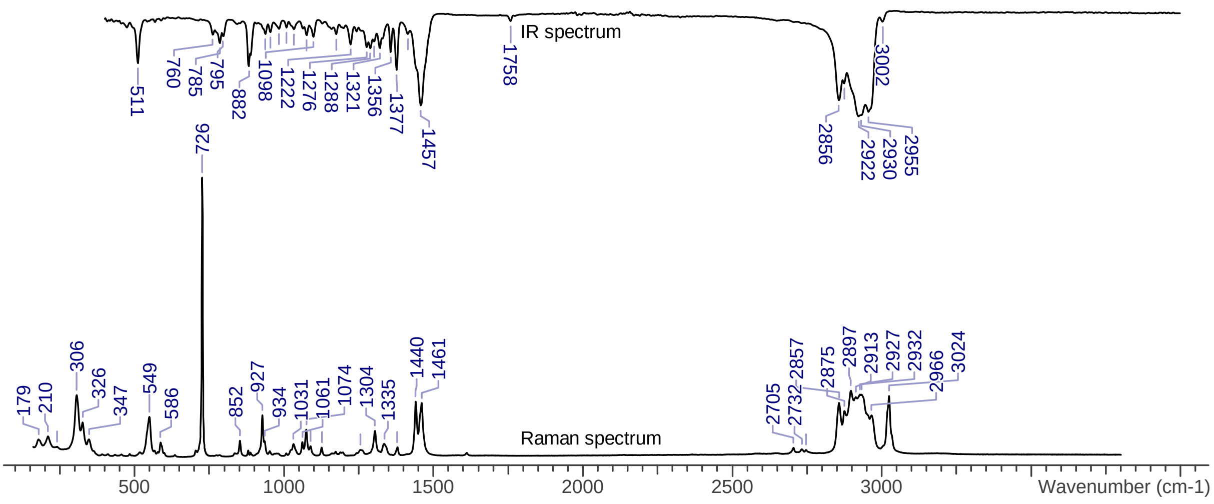

3.5 EMindNO$_2$

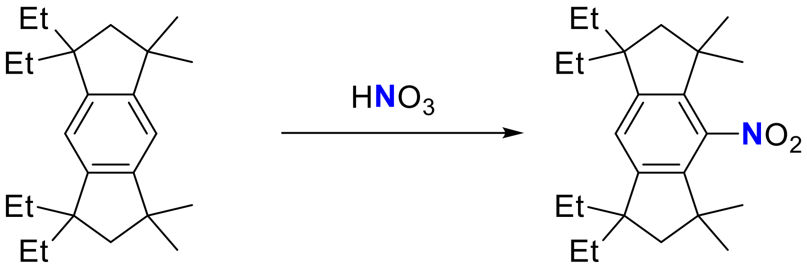

EMindNO$_2$ was synthesized according to a slightly modified literature procedure.[15] The synthesis was carried out under non-inert conditions. EMindH (41.4 g, 126 mmol) was dissolved in a mixture of CH_2Cl_2 (500 mL), Ac_2O (340 mL) and AcOH (250 mL). To this solution aq. HNO_3 (65%, 86 mL, 1.23 mol) was added with a dropping funnel over a period of 2 h at 0 °C (ice bath). The reaction mixture was stirred for 1 h at 0 °C. Afterwards, it was quenched by addition of water (500 mL) and subsequently extracted with CH_2Cl_2 (400 mL). The organic phase was separated and washed with water (3×300 mL), a mixture of aqueous Na_2CO_3 (5% (w/w), 900 mL) and finally again with water (300 mL). The organic phase was dried over $MgSO_4$. The $MgSO_4$ was removed by filtration. The filtrate was concentrated *in vacuo* using a rotary evaporator until yellowish solids remained. The solids were placed in a funnel filter with a sintered glass disc and was washed with *n*-hexane (3×50 mL) until the product remained as colorless solid. The product was dried *in vacuo* (1×10^{-3} mbar) at 50 °C (water bath) for 2 h. Yield: 24.4 g (65.7 mmol, 52%).

Annotation: An attempt was made to extract further product from the washing solution. The solvent was removed using a rotary evaporator. The residue was recrystallized from boiling *n*-hexane, yielding a 1:1 mixture of the desired EMindNO$_2$ and its *para*-substituted isomer. Whether the product can be separated by flash chromatography has not been tested.

Mp. 162 °C (lit.: 120 °C)[15]. **CHN** calc. (found) in %: C 77.58 (77.06), H 10.04 (10.16), N 3.77 (3.93). **^{1}H NMR** ($CDCl_3$, 500.1 MHz): δ = 0.81 (dd, $^3J(^1H,^1H)$ = 7.5 Hz, $^3J(^1H,^1H)$ = 7.3 Hz, 12 H, CH_3 (Et)), 1.33 (s, 12 H, CH_3 (Me)), 1.60 (dq, $^2J(^1H,^1H)$ = 13.8 Hz, $^3J(^1H, ^1H)$

= 7.3 Hz, 4 H, C(*H*)H (Et)),1.68 (dq, $^{2}J(^{1}H,^{1}H)$ = 13.8 Hz, $^{3}J(^{1}H, ^{1}H)$ = 7.6 Hz, 4 H, C(H)*H* (Et)), 1.92 (s, 4 H, CH_2 (cyclopent.)), 6.79 (s, 1H, C*H*). **^{13}C{^{1}H} NMR** ($CDCl_3$, 75.5 MHz): δ = 9.0 (s; CH_3 (Et)), 30.0 (s, CH_3 (Me)), 32.5 (s, CH_2 (Et)), 43.0 (s, $C_{quart.}$), 48.5 (s, $C_{quart.}$), 53.0 (s, CH_2 (cyclopent.)), 121.5 (s, *C*H), 138.8 (s, aromatic $C_{quart.}$), 145.4 (s, aromatic $C_{quart.}$), 151.0 (s, aromatic $C_{quart.}$). **IR** (ATR, 32 scans, cm^{-1}): $\tilde{\nu}$ = 492 (w), 554 (w), 756 (m), 791 (m), 909 (m), 940 (w), 960 (w), 1024 (w), 1090 (w), 1170 (w), 1280 (m), 1325 (m), 1364 (s), 1377 (m), 1453 (m), 1525 (vs), 1603 (vw), 2860 (m), 2877 (m), 2934 (s), 2961 (m). **Raman** (633 nm, 10 s, 10 scans, cm^{-1}): $\tilde{\nu}$ = 114 (2), 132 (3), 150 (3), 163 (2), 187 (1), 208 (1), 222 (2), 240 (1), 275 (1), 313 (2), 335 (1), 383 (1), 419 (1), 497 (1), 520 (1), 553 (5), 592 (2), 701 (1), 723 (1), 756 (8), 831 (1), 844 (1), 858 (1), 907 (1), 930 (1), 959 (1), 977 (1), 1013 (1), 1023 (1), 1036 (1), 1062 (2), 1090 (2), 1098 (1), 1128 (2), 1160 (1), 1185 (1), 1201 (1), 1224 (1), 1250 (1), 1264 (1), 1291 (1), 1303 (2), 1319 (1), 1340 (1), 1373 (2), 1379 (2), 1408 (1), 1438 (2), 1461 (4), 1469 (3), 1527 (1), 1553 (1), 1603 (1), 2719 (1), 2736 (1), 2759 (1), 2863 (4), 2877 (4), 2926 (10), 2942 (10), 2963 (5), 2973 (7). **MS** (EI, 70 eV, m/z, rel. int. > 10%): 243 (100) $[M-C_2H_4]^+$, 244 (33) $[M-C_2H_4]^+$.

Figure S14: NMR, IR and Raman spectra of EMindNO$_2$ (solvent signals indicated by asterisks).

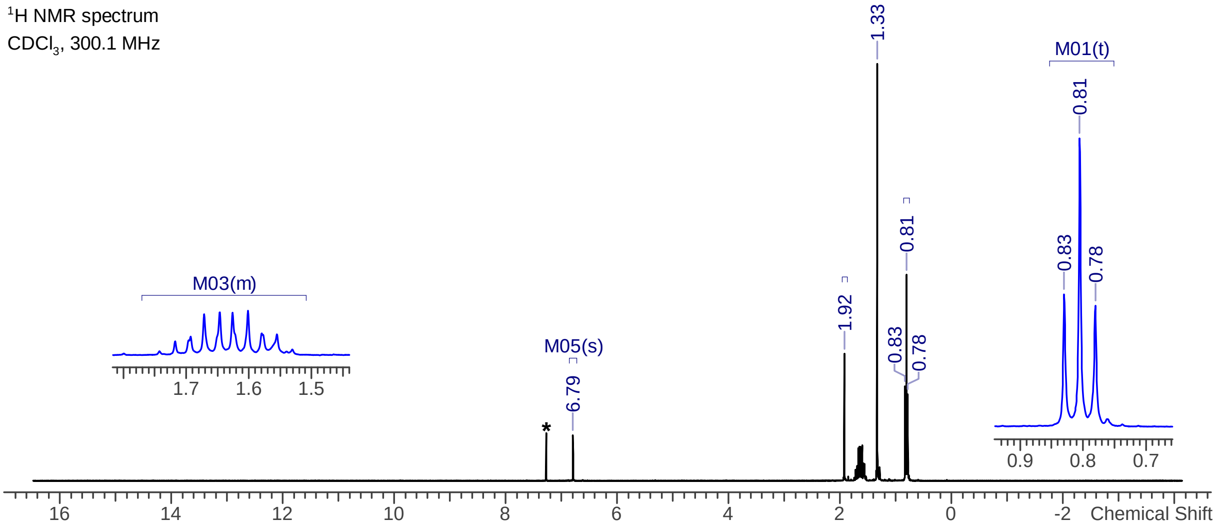

experimental and simulated ^{1}H NMR spectrum
$CDCl_3$, 300.1 MHz

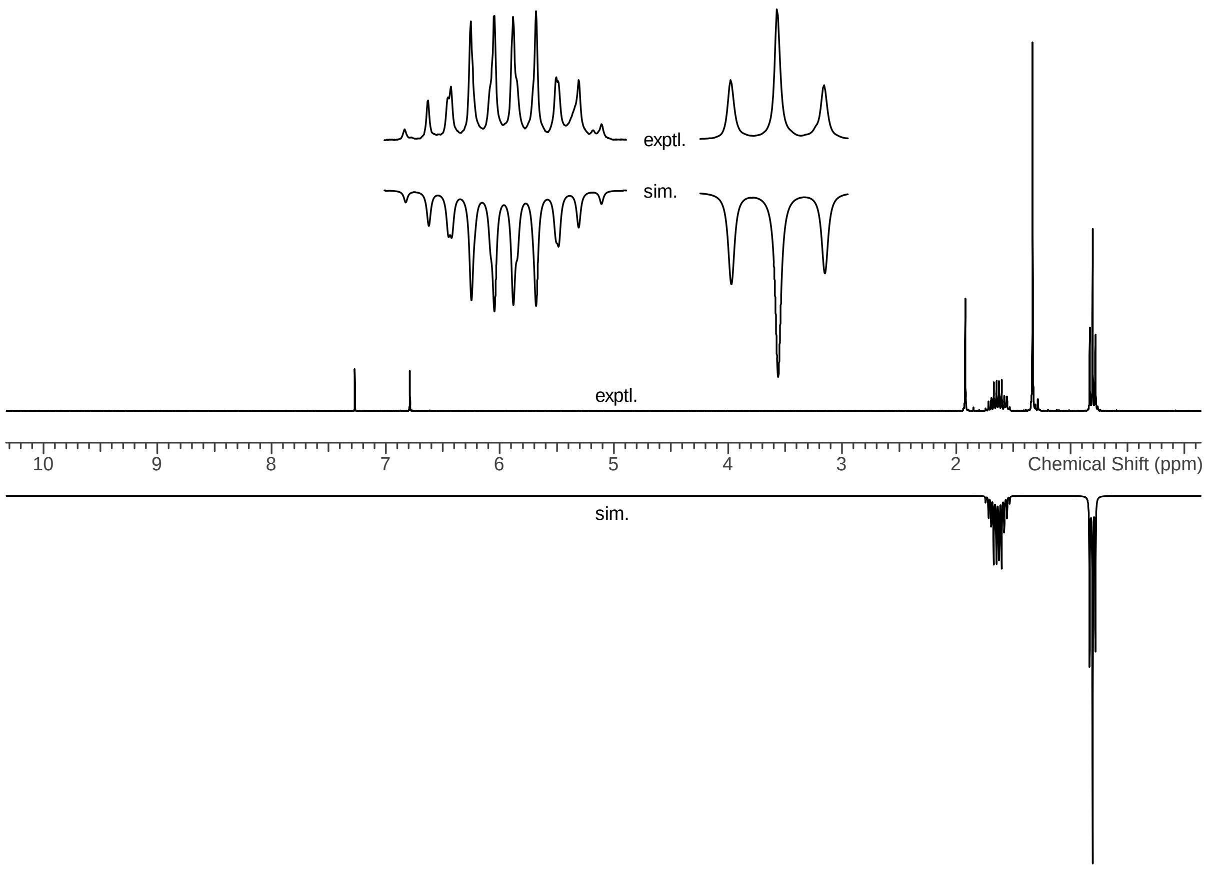

$^{13}C\{^{1}H\}$ NMR spectrum
$CDCl_3$, 75.5 MHz

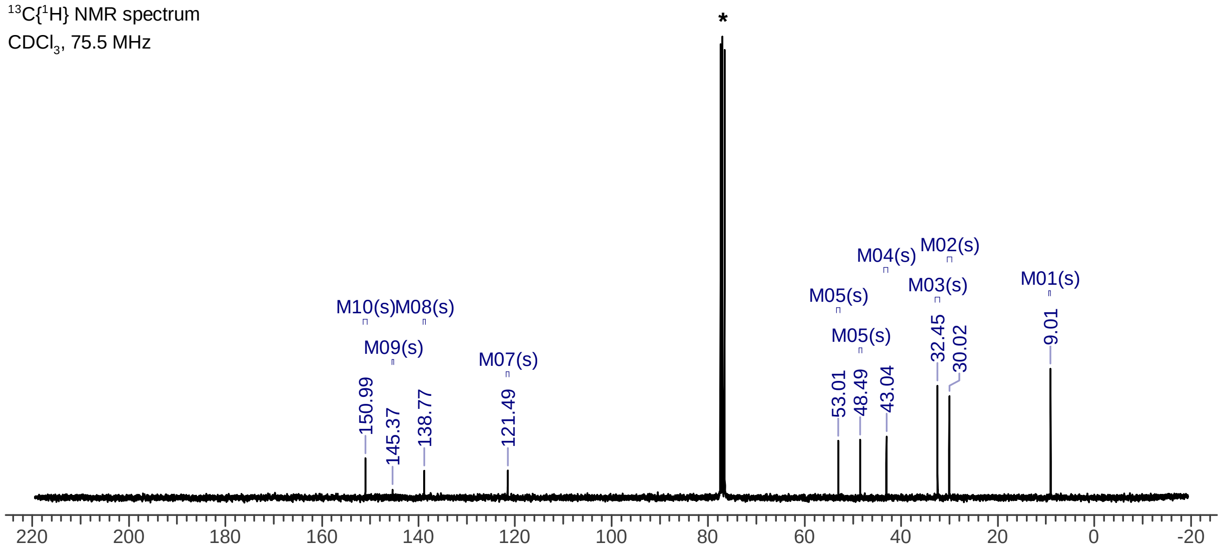

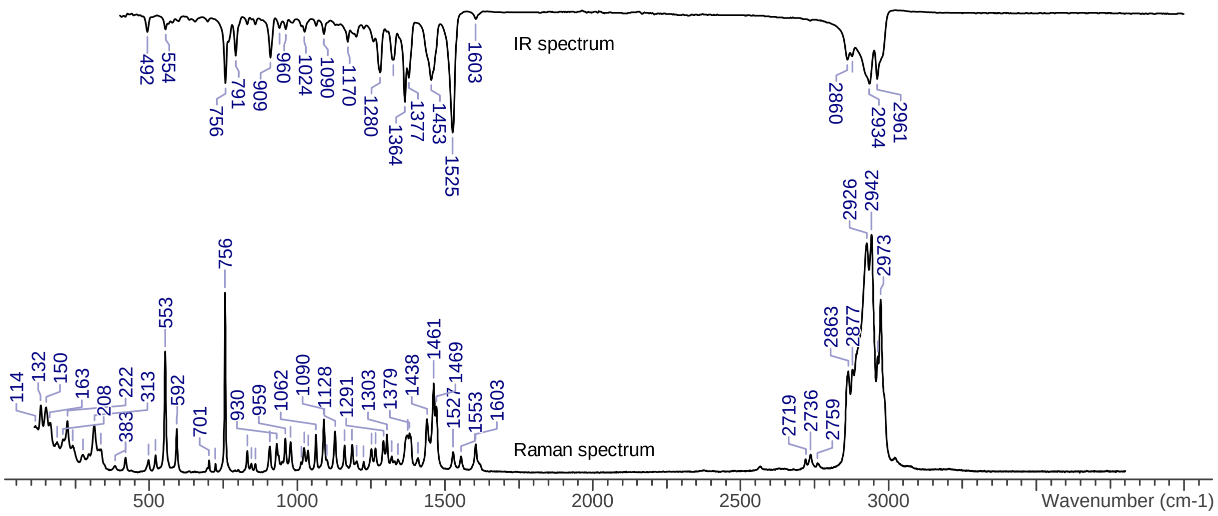
IR spectrum
492
554
756
791
909
960
1024
1090
1170
1280
1364
1377
1453
1525
1603
2860
2934
2961
Raman spectrum
114
132
150
163
208
222
313
383
553
592
701
756
930
959
1062
1090
1128
1291
1303
1379
1438
1461
1469
1527
1553
1603
2719
2736
2759
2863
2877
2926
2942
2973
500
1000
1500
2000
2500
3000
Wavenumber (cm-1)

3.6 EMindNH$_2$

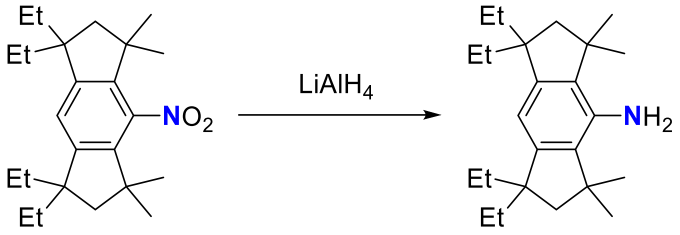

First, we tried to prepare EMindNH$_2$ according to a literature procedure in which NaBH$_4$ (with NiCl$_2$·6 H$_2$O as catalyst) was used as a reducing agent.[15] *However, the synthesis could not be reproduced (We used dichloromethane as a solvent [the original text says "**1,2**-dichloro**methane**"]). We assume that the water content of the solvent is of elementary importance in this synthesis, since we could not observe any conversion under water exclusion. When water was explicitly added, conversion to a para-chloro substituted derivative of EMindNH$_2$ was observed. For this reason, we developed a new synthesis procedure based on the previously published synthesis of Mes*NH$_2$.*[16]

EMindNO$_2$ (15.00 g, 40.37 mmol) and LiAlH$_4$ (6.129 g, 161.5 mmol) were placed in a 500 mL three-necked flask equipped with two reflux condensers (parallel arrangement), each equipped with a pressure relief valve. The mixture was cooled to −80 °C (*i*-PrOH/N$_2$ cooling bath). Cold THF (−80 °C, 250 mL) was added and the reaction mixture was stirred for 15 min. The cooling bath was removed and the reaction mixture was slowly warmed to ambient temperature. Afterwards the reaction mixture was refluxed for 3 h (heating mantle), whereupon the mixture slowly changed from a green solution to a colorless suspension. *Attention: At the beginning, the reaction is strongly exothermic, so that the heating mantle may have to be removed temporarily.* The reaction mixture was cooled to 0 °C (ice bath) and was quenched with a minimal amount of water. The addition of water was stopped as soon as no more hydrogen was formed. The further workup proceeded under non-inert conditions. Et$_2$O (700 mL) was added and solid byproducts were removed by filtration. The filtrate was extracted with water (100 mL) to ensure that all LiAlH$_4$ was quenched. The organic phase was separated and dried with MgSO$_4$. MgSO$_4$ was removed by filtration. The filtrate was

evaporated using a rotary evaporator and the solid residue was recrystallized from boiling Et_2O (20 mL). The product was collected in two fractions of colorless crystals and was dried *in vacuo* (1×10^{-3} mbar) for 2 h at 60 °C (water bath). Yield: 12.82 g (37.53 mmol, 93%).

Single crystals suitable for X-ray diffraction were grown as described in the synthesis procedure.

Mp. 95 °C (lit.: 92-93 °C)[15]. **CHN** calc. (found) in %: C 84.39 (83.76), H 11.51 (10.58), N 4.10 (3.97). **^{1}H NMR** (C_6D_6, 500.1 MHz): δ = 0.88 (t, $^3J(^1H, ^1H)$ = 7.4 Hz, 12 H, CH_3 (Et)), 1.38 (s, 12 H, CH_3 (Me)), 1.57 - 1.71 (m, 8 H, CH_2 (Et)), 1.82 (s, 4 H, CH_2) 3.37 (s, 2 H, NH_2) 6.33 (s, 1 H, C*H*). **^{13}C{^{1}H} NMR** (C_6D_6, 125.8 MHz): δ = 9.9 (s, CH_3), 29.8 (s, CH_3), 34.0 (s, CH_2 (Et)), 42.7 (s, CMe_2), 49.7 (s, CEt_2), 53.7 (s, CH_2), 110.1 (s, CH), 133.9 (s, aromatic $C_{quart.}$ (Me side)), 140.2 (s, *C*-NH_2), 149.7 (s, aromatic $C_{quart.}$ (Et side)). **IR** (ATR, 32 scans, cm^{-1}): $\tilde{\nu}$ = 433 (s), 449 (m), 472 (m), 637 (m), 764 (m), 785 (m), 800 (m), 841 (m), 868 (m), 907 (w), 936 (w), 954 (m), 1032 (m), 1061 (w), 1088 (m), 1222 (m), 1259 (w), 1276 (m), 1333 (m), 1348 (m), 1374 (s), 1422 (s), 1459 (s), 1572 (m), 1613 (s), 2860 (s), 2875 (s), 2930 (vs), 2959 (vs), 3402 (vw), 3478 (vw). **Raman** (633 nm, 10 s, 20 scans, cm^{-1}): $\tilde{\nu}$ = 114 (5), 137 (1), 245 (1), 307 (3), 329 (7), 356 (2), 388 (1), 528 (1), 591 (3), 612 (10), 644 (9), 762 (1), 825 (1), 908 (2), 924 (1), 933 (1), 1001 (1), 1031 (1), 1061 (3), 1106 (2), 1129 (1), 1161 (1), 1185 (1), 1222 (1), 1265 (1), 1273 (1), 1291 (1), 1310 (3), 1345 (3), 1372 (1), 1438 (3), 1464 (5), 1570 (2), 1613 (2), 2715 (1), 2730 (1), 2863 (4), 2878 (5), 2893 (5), 2896 (5), 2931 (7), 2940 (6), 2943 (6), 2958 (4), 2964 (4), 3028 (2), 3075 (2), 3400 (3). **MS** (EI, 70 eV, m/z, rel. int. > 10%): 342 (12) $[M]^+$, 341 (52) $[M]^+$, 326 (44) $[M-Me]^+$, 313 (29) $[M-Et]^+$, 312 (100) $[M-Et]^+$.

Figure S15: NMR, IR and Raman spectra of EMindNH$_2$ (solvent signals indicated by asterisks).

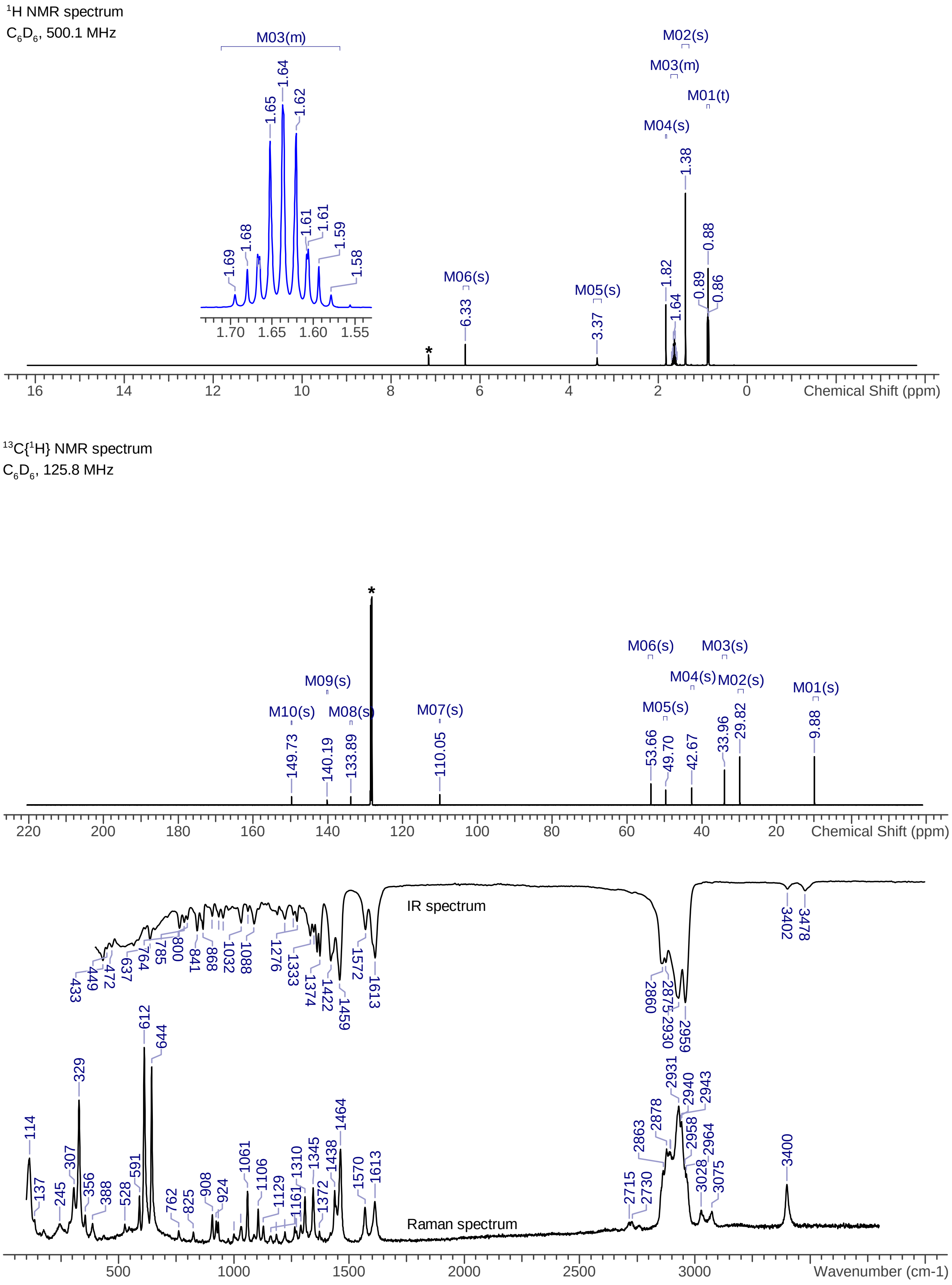

4 Syntheses of compounds

4.1 3Ter

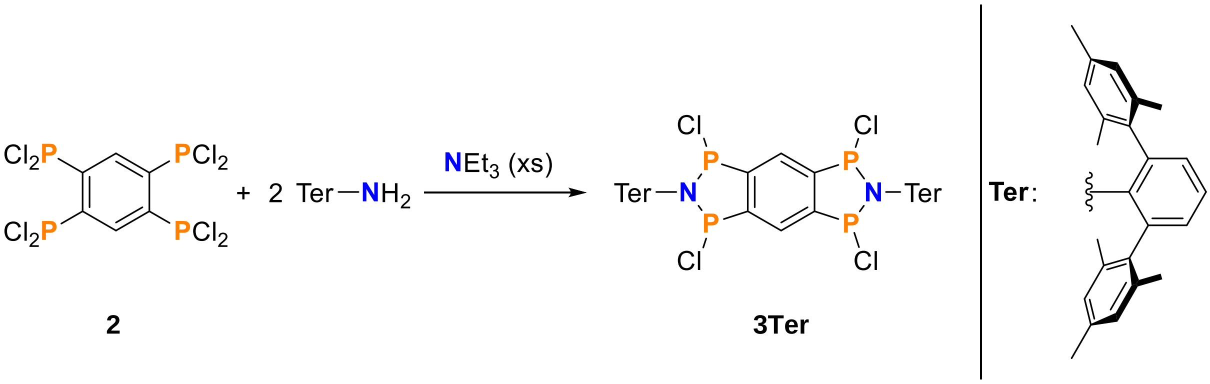

2 **3Ter**

To a solution of **2** (2 923 mg, 6.070 mmol) in CH_2Cl_2 (40 mL), NEt_3 (19.4 mL, 140 mmol) was added at 0 °C (ice bath). Afterwards a solution of $TerNH_2$ (4 000 mg, 12.14 mmol) in CH_2Cl_2 (40 mL) was added over a period of 2 min at 0 °C. The ice bath was removed. After stirring for 24 h at ambient temperature, all volatile components were removed *in vacuo* (1×10^{-3} mbar) and the residue was dried *in vacuo* (1×10^{-3} mbar) for 2 h at 60 °C (water bath). The solids were extracted with Et_2O (125 mL) in a Soxhlet apparatus (under argon atmosphere) for 5 days. The solvent of the extract was removed *in vacuo* and replaced by benzene (80 mL). The solution was concentrated to approx. 50 mL and was cooled from 60 °C to ambient temperature in a water bath overnight yielding colorless block shaped crystals of **3Ter**. The supernatant was removed by syringe and the crystals were washed twice with a minimal amount of cold benzene (5 °C, 2 x 1 mL). The supernatant and washing solution were combined and concentrated, resulting in a second crop of colorless crystals, which were treated in the same manner. The crystals of the first and second crop were combined and dried *in vacuo* (1×10^{-3} mbar) for 2 h at 60 °C (water bath). Yield: 3.725 g (3.745 mmol, 62%).

Single crystals suitable for X-ray diffraction were grown as described in the synthesis procedure.

Mp. 380 °C (dec.). **CHN** calc. (found) in %: C 65.20 (65.29), H 5.27 (5.38), N 2.82 (2.79). **^{31}P{^{1}H} NMR** (CD_2Cl_2, 202.5 MHz): δ = 142.6 (s, 4 P). **^{1}H NMR** (CD_2Cl_2, 500.1 MHz): δ = 1.6 - 2.5 (superimposed signals, 36 H, C*H*3), 6.3 - 7.0 (superimposed signals, 8 H, C*H* Mes), 7.14 (bs, 4 H, *m*-C*H* (Ter)) 7.42 (m, 2H, *p*-C*H* (Ter)), 7.53 (m, 2 H, C*H* ($C_6H_2P_4$)).**^{13}C{^{1}H} NMR** (CD_2Cl_2, 125.8 MHz): δ = 21.5-22.0 (CH_3 (Ter)), 125.3 (t, $^{2}J(^{1}H, ^{13}C)$ = 38 Hz, *C*H ($C_6H_2P_4$)), 128.4 (s, *p*-*C*H (Ter)), 128.9 (s, *m*-*C*H (Mes)), 131.3 (bs, m-*C*H, Ter), 137.2 (bs, $C_{quart.}$ (Mes)), 141.1 (s, $C_{quart.}$ (Ter)), 141.3 ($C_{quart.}$ (Ter)), 153.0 (dd, $^{1}J(^{1}H,^{13}C)$ = 27 Hz, $^{2}J(^{1}H,^{13}C)$ = 9 Hz, $C_{quart.}$ ($C_6H_2P_4$)), two of the $C_{quart.}$(Mes) could not be found by two dimensional NMR spectra. **IR** (ATR, 32 scans, cm^{-1}): $\tilde{\nu}$ = 433 (s), 474 (vs), 495 (s), 521 (m), 532 (m), 561 (vw), 596 (m), 693 (w), 736 (w), 754 (w), 804 (m), 847 (m), 895 (s), 1080 (w), 1105 (w), 1193 (w), 1266 (w), 1375 (w), 1412 (w), 1453 (w), 1610 (w), 2851 (vw), 2914 (w), 2941 (w), 2966 (vw). **Raman** (633 nm, 15 s, 20 scans, cm^{-1}): $\tilde{\nu}$ = 148 (1), 179 (1), 235 (1), 252 (1), 269 (1), 277 (1), 329 (1), 391 (1), 405 (1), 441 (1), 481 (1), 523 (1), 561 (3), 576 (2), 608 (1), 704 (2), 984 (1), 992 (10), 1006 (1), 1081 (1), 1135 (1), 1165 (1), 1174 (1), 1305 (1), 1376 (1), 1383 (1), 1567 (1), 1586 (1), 1612 (1), 2858 (1), 2918 (1), 2949 (1), 3013 (1), 3035 (1), 3063 (3). **MS** (CI, pos., isobutene, m/z): 1003-993 $[M+H]^+$, 967-958 $[M+H-Cl]^+$, 930-923 $[M+H-Cl_2]^+$, 894-889 $[M+H-Cl_3]^+$.

Figure S16: NMR, IR and Raman spectra of **3Ter** (solvent signals indicated by asterisks).

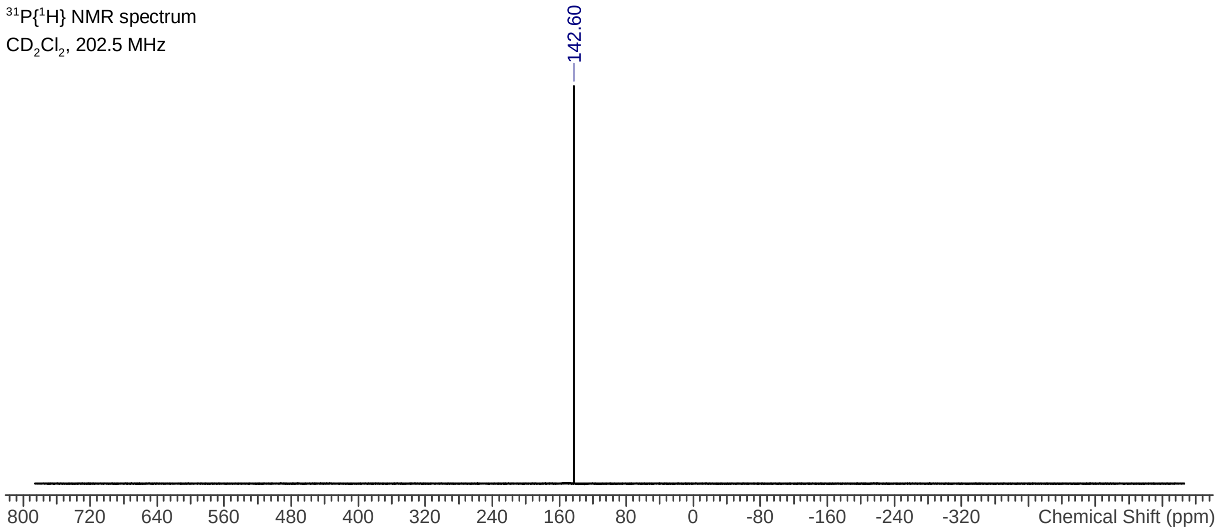

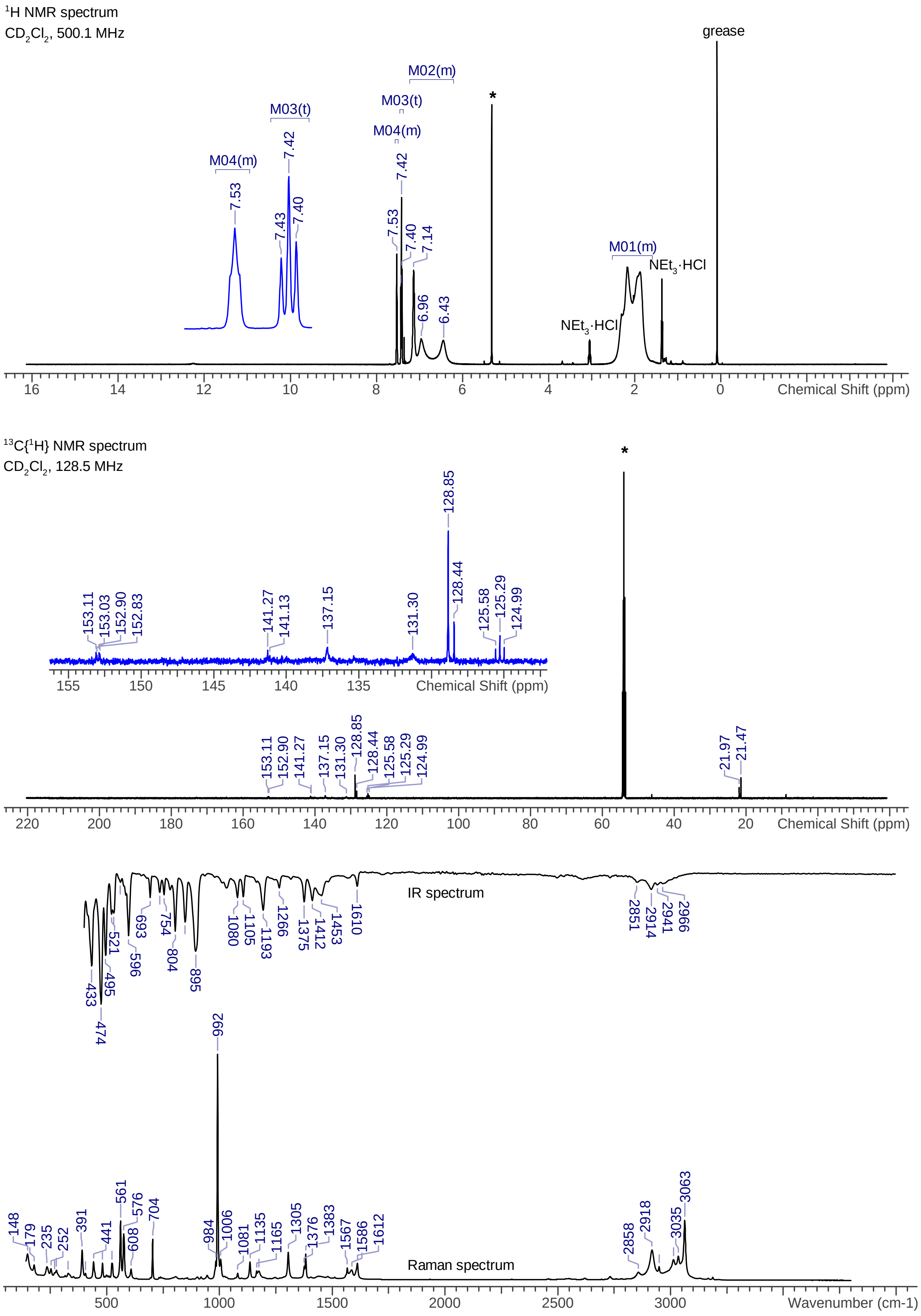

1H NMR spectrum
CD2Cl2, 500.1 MHz
grease
M04(m)
M03(t)
M02(m)
M01(m)
NEt3·HCl
Chemical Shift (ppm)
13C{1H} NMR spectrum
CD2Cl2, 128.5 MHz
IR spectrum
Raman spectrum
Wavenumber (cm-1)

4.2 3EMind

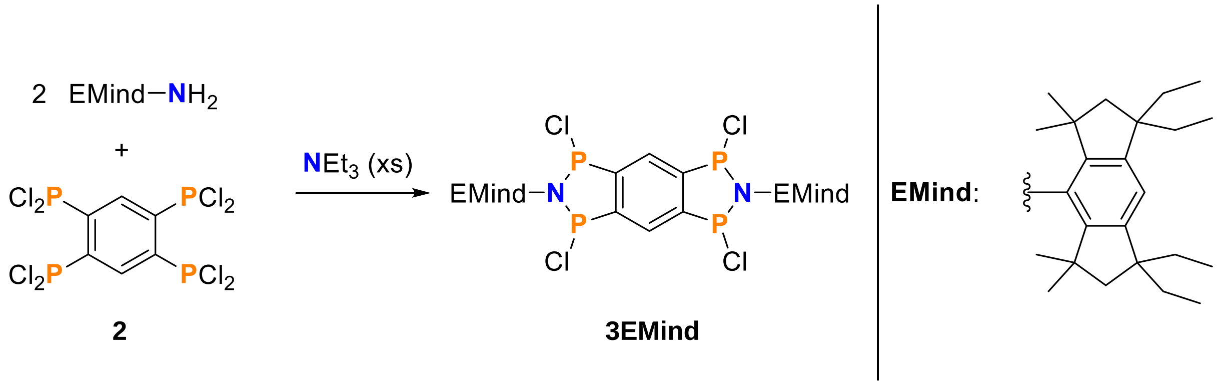

To a solution of **2** (4.619 g, 9.591 mmol) in CH_2Cl_2 (200 mL), NEt_3 (30.0 mL, 216 mmol) was added at 0 °C (ice bath). Afterwards a solution of $EMindNH_2$ (6.553 g, 19.183 mmol) in CH_2Cl_2 (80 mL) was added over a period of 2 min at 0 °C. The ice bath was removed. After stirring for 6 days at ambient temperature, all volatile components were removed *in vacuo* (1×10^{-3} mbar) and the residue was dried *in vacuo* (1×10^{-3} mbar) for 2 h at 60 °C (water bath). The residue was extracted with warm (~60 °C) benzene (400 mL) and insoluble solids were removed by filtration. The extraction was repeated twice with fresh benzene (20 mL each). The combined filtrates were re-filtered over a plug of celite. The clear orange filtrate was concentrated in the warmth (~60 °C) to incipient crystallization and was cooled to ambient temperature overnight. Colorless block shaped crystals were obtained. The supernatant was removed by syringe and was concentrated, resulting in a second crop of colorless crystals. The crystals were dried *in vacuo* (1×10^{-3} mbar) for 2 h at 60 °C (water bath). Yield: 7.591 g (7.450 mmol, 78%).

Single crystals suitable for X-ray diffraction were grown as described in the synthesis procedure.

Mp. 360-375 °C (dec.). **CHN** calc. (found) in %: 63.66 (65.49), H 7.52 (7.38), N 2.75 (2.65); deviations probably due to hydrolysis problems, repeated measurements did not result in better agreement **$^{31}P\{^{1}H\}$ NMR** (C_6D_6, 202.5 MHz): δ = 144.9 (s). **^{1}H NMR** (C_6D_6, 500.1 MHz): δ = 0.84 (m, 24 H, CH_3 (Et)), 1.14 (m, 12 H, CH_3 (Me), 1.57 (m, 8 H, CH_2 (Et)), 1.69 (m, 8 H, CH_2 (Et)), 1.76 (s, 4H, CH_2 (cyclopent.)), 1.88 (s, 4H, CH_2 (cyclopent.), 1.99

(s, 12 H, CH_3 (Me)), 6.90 (s, 2 H, C*H* (EMind)), 7.31 (m, 2 H, $C_6H_2P_4$). **$^{13}C\{^1H\}$ NMR** (C_6D_6, 125.8 MHz): δ = 8.8 (s, CH_3 (Et)), 9.0 (s, CH_3 (Et)), 32.3 (s, CH_2 (Et)), 32.5 (d, $J(^{13}C, ^{31}P)$ = 21 Hz, CH_3 (Me)), 32.9 (s, CH_3 (Me)), 32.9 (s, CH_2 (Et)), 43.6 (s, CEt_2), 44.4 (s, CEt_2), 47.1 (s, CMe_2), 47.6 (s, CMe_2), 53.2 (s, CH_2 (cyclopent.)), 55.1 (s, CH_2 (cyclopent.)), 121.0 (*C*H (EMind)), 126.7 (*C*H ($C_6H_2P_4$)), quaternary, aromatic C not reported due to low signal to noise ratio. **IR** (ATR, 32 scans, cm^{-1}): $\tilde{\nu}$ = 439 (s), 462 (m), 484 (vs), 505 (s), 528 (w), 592 (s), 616 (w), 674 (s), 734 (m), 789 (m), 855 (w), 878 (s), 938 (w), 956 (w), 1000 (m), 1033 (w), 1078 (w), 1107 (w), 1165 (w), 1202 (w), 1263 (w), 1327 (w), 1340 (w), 1360 (w), 1375 (w), 1459 (m), 1478 (w), 1552 (vw), 1595 (vw), 2873 (w), 2927 (m), 2958 (m), 3032 (vw). **Raman** (633 nm, 10 s, 20 scans, cm^{-1}): $\tilde{\nu}$ = 132 (9), 150 (4), 225 (2), 252 (2), 310 (1), 330 (1), 349 (2), 399 (4), 408 (3), 441 (2), 483 (10), 497 (2), 504 (2), 532 (1), 538 (1), 555 (1), 562 (1), 593 (1), 608 (1), 620 (1), 651 (1), 703 (1), 735 (3), 767 (1), 792 (1), 836 (1), 849 (1), 886 (1), 899 (1), 913 (1), 932 (1), 955 (1), 979 (1), 984 (1), 992 (9), 1032 (1), 1063 (1), 1078 (1), 1101 (1), 1108 (1), 1132 (2), 1143 (1), 1175 (2), 1194 (1), 1228 (1), 1272 (2), 1308 (2), 1331 (1), 1342 (1), 1383 (1), 1447 (2), 1465 (2), 1513 (1), 1556 (1), 1587 (1), 1596 (1), 1606 (1), 2528 (1), 2734 (1), 2756 (1), 2828 (1), 2862 (4), 2877 (4), 2906 (6), 2929 (8), 2978 (3), 3046 (2), 3063 (3). **MS** (CI, pos., isobutene, m/z): 988-983 $[M+2\ H-Cl]^+$, 952-948 $[M+2\ H-2\ Cl]^+$, 915-912 $[M+2\ H-3\ Cl]^+$.

Figure S17: NMR, IR and Raman spectra of **3EMind** (solvent signals indicated by asterisks).

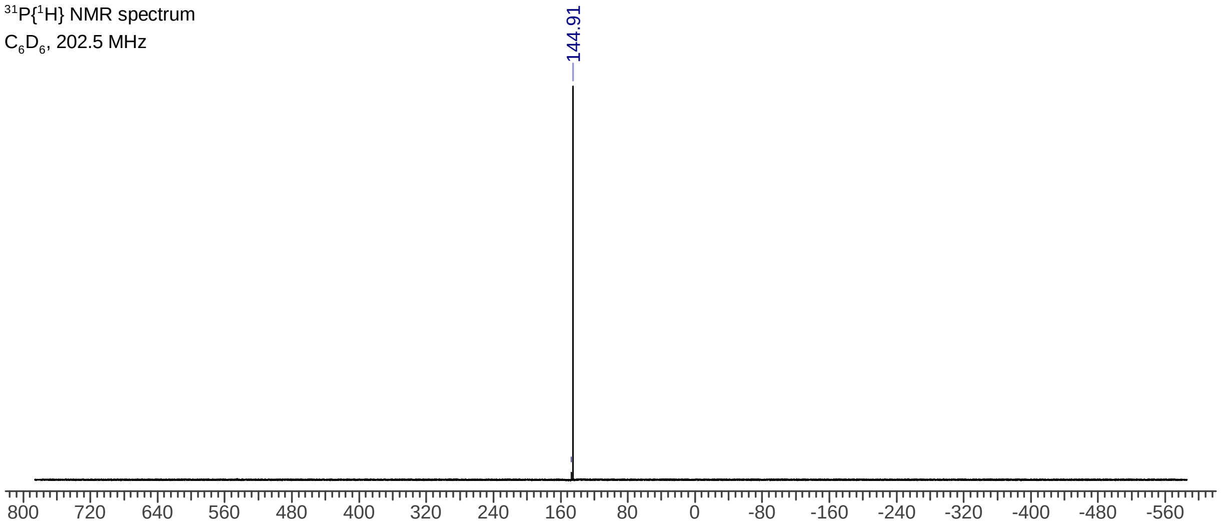

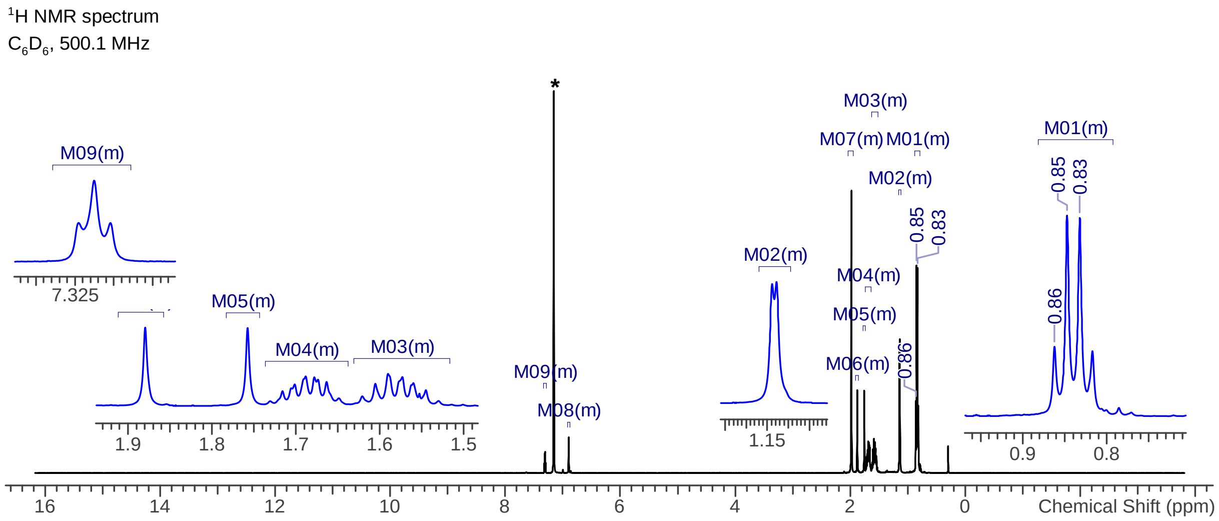
1H NMR spectrum
C6D6, 500.1 MHz
M09(m)
7.325
M05(m)
M04(m)
M03(m)
1.9
1.8
1.7
1.6
1.5
M09(m)
M08(m)
M02(m)
1.15
M03(m)
M07(m) M01(m)
M02(m)
0.85
0.83
M04(m)
M05(m)
M06(m)
0.86
M01(m)
0.85
0.83
0.86
0.9
0.8
16
14
12
10
8
6
4
2
0
Chemical Shift (ppm)

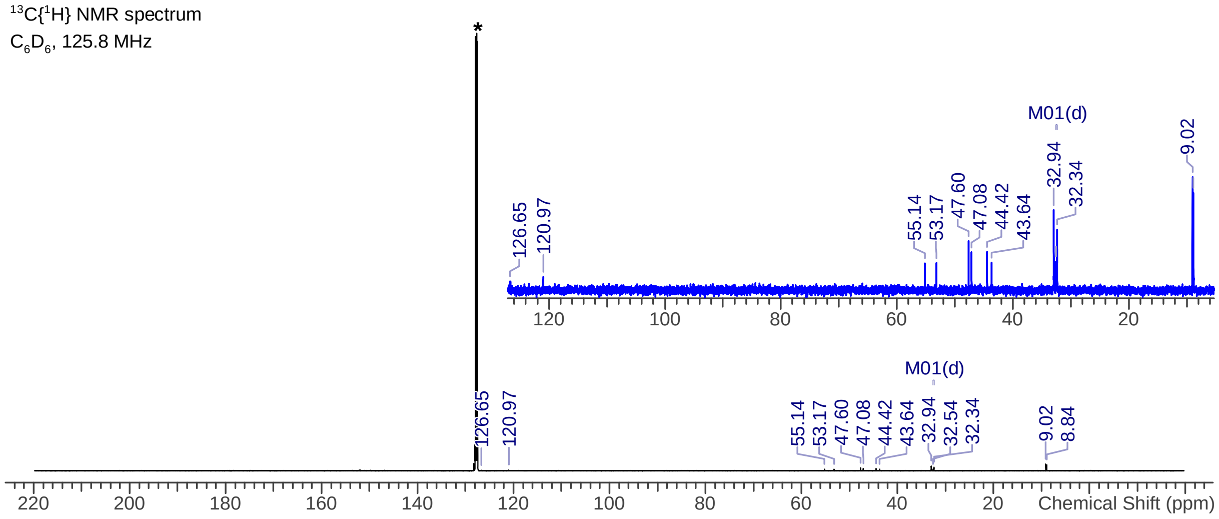
13C{1H} NMR spectrum
C6D6, 125.8 MHz
M01(d)
126.65
120.97
55.14
53.17
47.60
47.08
44.42
43.64
32.94
32.34
9.02
120
100
80
60
40
20
M01(d)
126.65
120.97
55.14
53.17
47.60
47.08
44.42
43.64
32.94
32.54
32.34
9.02
8.84
220
200
180
160
140
120
100
80
60
40
20
Chemical Shift (ppm)

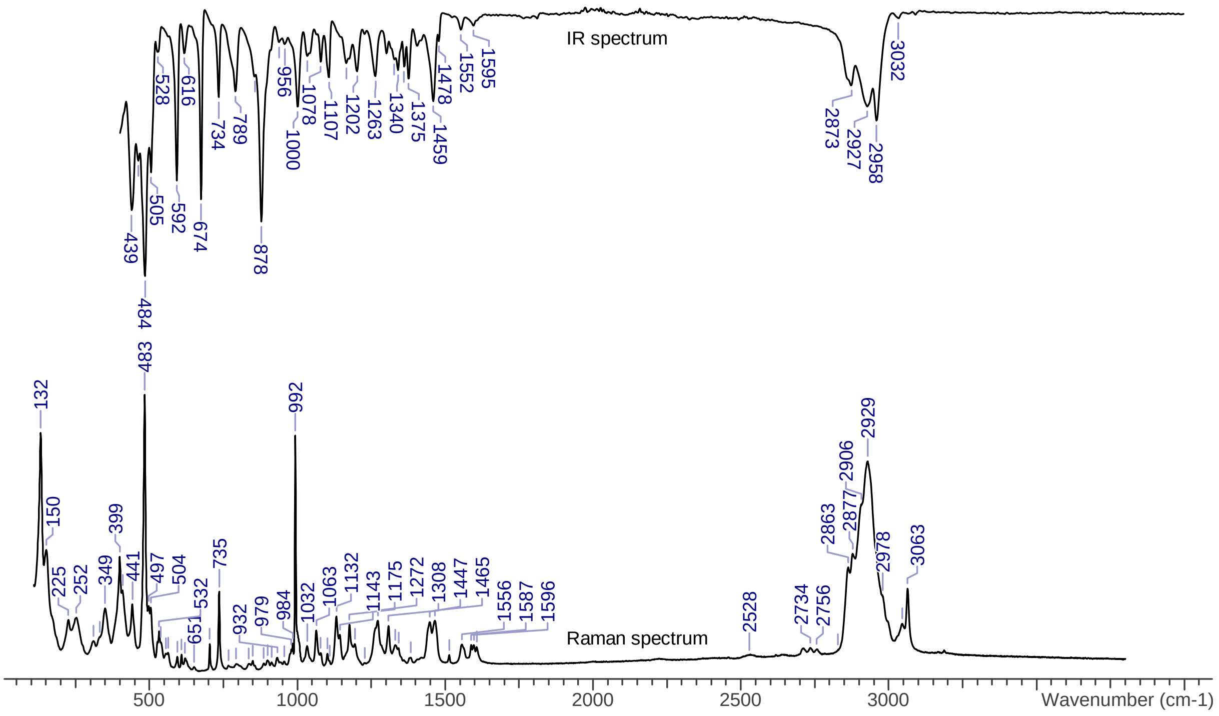
IR spectrum
528
616
734
789
956
1000
1078
1107
1202
1263
1340
1375
1459
1478
1552
1595
439
484
505
592
674
878
2873
2927
2958
3032
Raman spectrum
132
150
225
252
349
399
441
483
497
504
532
651
735
932
979
984
992
1032
1063
1132
1143
1175
1272
1308
1447
1465
1556
1587
1596
2528
2734
2756
2863
2877
2906
2929
2978
3063
500
1000
1500
2000
2500
3000
Wavenumber (cm-1)

4.3 3Mes*

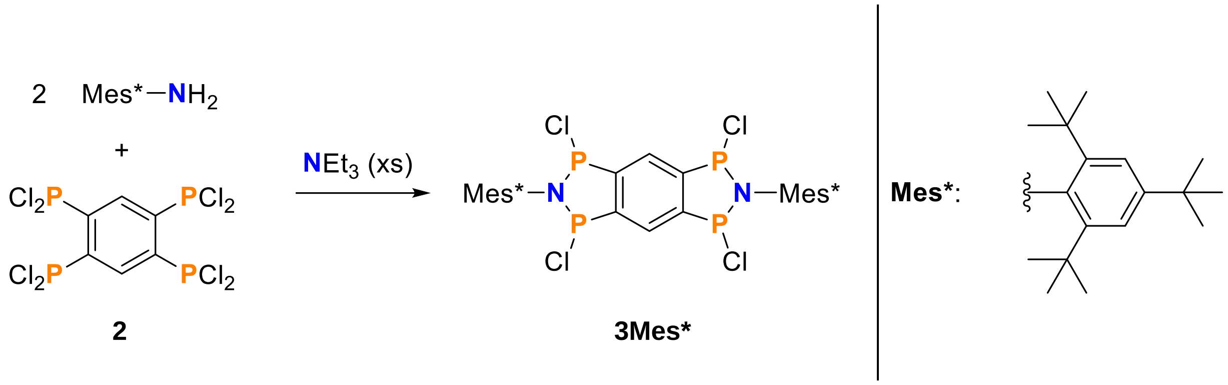

To a solution of **2** (2.763 g, 5.737 mmol) in CH_2Cl_2 (50 mL), NEt_3 (18.0 mL, 130 mmol) was added at 0 °C (ice bath). Afterwards a solution of Mes*NH_2 (3.000 g, 11.474 mmol) in CH_2Cl_2 (50 mL) was added over a period of 2 min at 0 °C. The ice bath was removed. After stirring for 6 days at ambient temperature, all volatile components were removed *in vacuo* (1×10^{-3} mbar) and the residue was dried *in vacuo* (1×10^{-3} mbar) for 3 h at 60 °C (water bath). Et_2O (100 mL) was added and the resulting suspension was extracted by repeated filtration and recondensation of the solvent (six times). Afterwards, the solvent of the filtrate was removed in vacuo (1×10^{-3} mbar). Analysis of the residue by $^{31}P\{^1H\}$ NMR spectroscopy indicated a non-selective reaction. For this reason, no further investigations were carried out with the Mes* substituent. However, a few crystals of compound **3Mes*** were obtained from dichloromethane. The structure (Figure S4) shows that at least some of the desired product was formed in the reaction.

Figure S18: $^{31}P\{^1H\}$ NMR spectrum from the residue obtained after the reaction of **2** with Mes*NH_2.

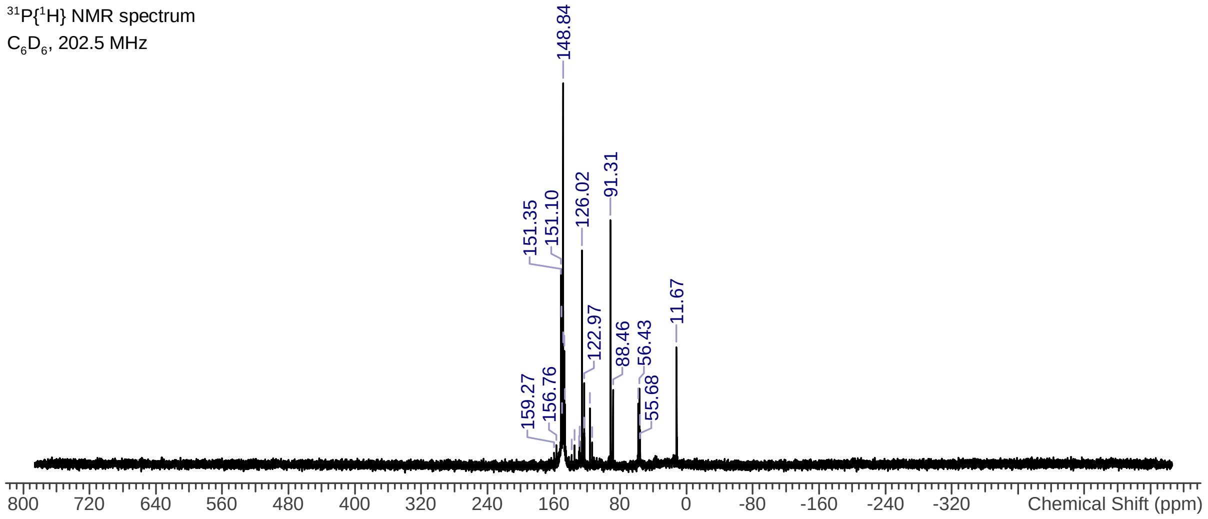

4.4 3^{tBu}Bhp

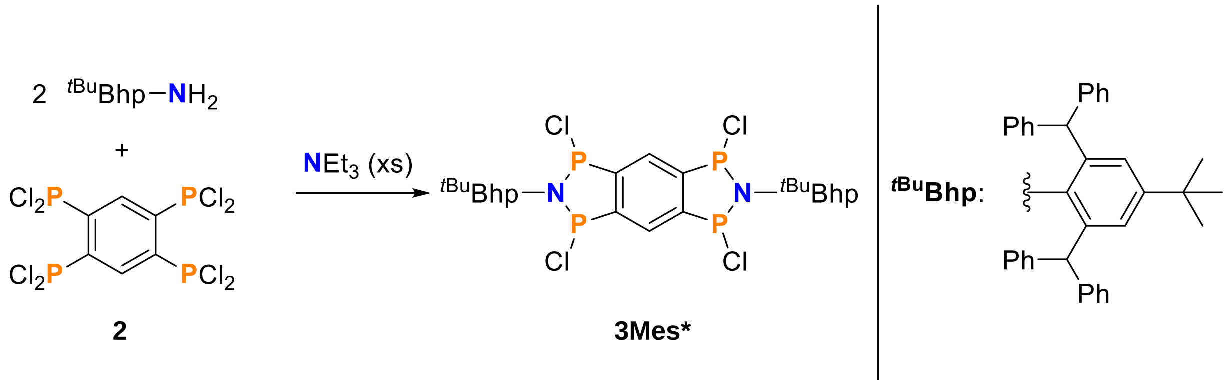

To a solution of **2** (4.000 g, 8.306 mmol) in CH_2Cl_2 (55 mL), NEt_3 (18.0 mL, 130 mmol) was added at 0 °C (ice bath). Afterwards a solution of ^{tBu}BhpNH_2 (8.002 g, 16.612 mmol) in CH_2Cl_2 (55 mL) was added over a period of 2 min at 0 °C. The ice bath was removed. After stirring for 24 h at ambient temperature, all volatile components were removed *in vacuo* (1×10^{-3} mbar) and the residue was dried *in vacuo* (1×10^{-3} mbar) for 3 h at 60 °C (water bath).

Major problems were encountered in the work-up of the product. **3^{tBu}Bhp** has a very poor solubility in non-polar solvents (benzene, Et_2O), so that a separation from triethylammonium chloride was not possible. (For example, not even a Soxhlet extraction of the residue over a period of 3 weeks led to complete extraction. The extract, however, contained **3^{tBu}Bhp** as well as triethylammonium chloride). Purification of **3^{tBu}Bhp** by selective crystallization was not successful, either. Due to these problems, research on **3^{tBu}Bhp** was abandoned.

4.5 4Ter

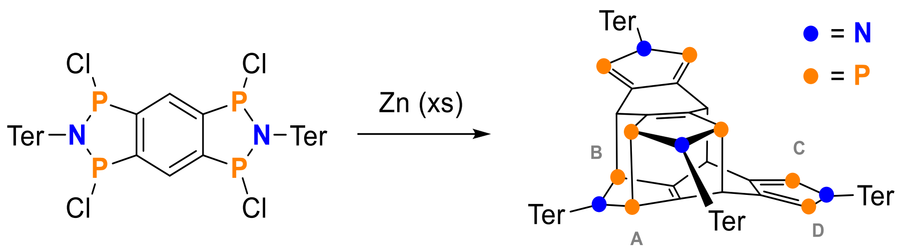

3Ter **4Ter**

Zn dust (5.61 g, 85.7 mmol) was added to a stirred (glass stir bar) solution of **3Ter** (200 mg, 0.201 mmol) in THF (10 mL). The solution first turned green and then brown as the reaction proceeded. After 2 days, Zn dust was removed by filtration. The solvent was removed *in vacuo* and the residue was dried for 2 h at 40 °C (water bath). The residue was suspended in benzene (15 mL) and insoluble material was removed by filtration. The solvent of the filtrate was removed *in vacuo* and replaced by THF (10 mL). The solution was concentrated in the warmth (~60 °C) to incipient crystallization (approx. 3 mL) and was cooled to ambient temperature overnight yielding yellow crystals. The supernatant was removed by syringe and discarded. The crystals were dried *in vacuo* (1×10^{-3} mbar) at 45 °C (water bath) for 2 h. Yield 45 mg (0.026 mmol, 26%).

Single crystals suitable for X-ray diffraction were grown as described in the synthesis procedure.

Mp. 180-200 °C (dec.). **CHN** calc. (found) for $C_{100}H_{104}N_4P_8$·1.6 THF in %: 74.08 (73.92), H 6.82 (6.78), N 3.28 (3.05). **^{31}P{^{1}H} NMR** (toluene-d_8, 202.5 MHz): δ = 119.6 (t, $J(^{31}P,^{31}P)$ = 5 Hz, 2 P, P_A), 136.0 (d, $J(^{31}P_B, ^{31}P_{D'})$ = 25 Hz, 2 P, P_B), 273.3 (s, 2 P, P_C), 280.4 (dd, $J(^{31}P_B, ^{31}P_{D'})$ = 25 Hz, $J(^{31}P,^{31}P)$ = 5 Hz, 2 P, P_D). Assignment see reaction equation above. **^{1}H NMR** (toluene-d_8, 500.1 MHz): δ = 1.73 (s, 6 H, CH_3 (Mes)), 2.02 (s, 6 H, CH_3 (Mes)), 2.13 (bs, superimposed by toluene resonance, 6 H, CH_3 (Mes)), 2.16 (bs, 6 H, CH_3 (Mes)), 2.25 (s, 6 H, CH_3 (Mes)), 2.29 (s, 6 H, CH_3 (Mes)), 2.29 (s, 6 H, CH_3 (Mes)), 2.35 (s, 6 H, CH_3 (Mes)), 2.40 (bs, 6 H, CH_3 (Mes)), 2.52 (s, 6 H, CH_3 (Mes)), 2.56

(m, 2 H, C*H* ($C_6H_2P_4$)), 3.69 (d, $J(^{31}P,^{1}H)$ = 7.9 Hz, 2 H, C*H* ($C_6H_2P_4$)), 6.17 (bs, 2 H, C*H* (Ter)), 6.63 (bs, 2 H, C*H* (Ter)), 6.67 (bs, 2 H, C*H* (Ter)), 6.76 (bs, 2 H, C*H* (Ter)), 6.90-7.15 (superimposed signals, 18 H, C*H* (Ter)), 7.23 (m, 2 H, C*H* (Ter)). **^{13}C{^{1}H} NMR** (toluene-d_8, 125.8 MHz): δ = 20.5-22.5 (signals of CH_3 groups), 53.0-54.5 (superimposed signals, *C*H ($C_6H_2P_4$)), 125.4-130.7 (signals of aromatic *C*H (Ter)), 136.0-143.2 (signals of aromatic $C_{quart.}$ (Ter)), 151.0-170.0 (superimposed signals, $C_{quart.}$ ($C_6H_2P_4$)). **IR** (ATR, 32 scans, cm^{-1}): $\tilde{\nu}$ = 418 (w), 476 (m), 501 (m), 519 (m), 530 (m), 559 (w), 571 (m), 596 (m), 616 (w), 635 (w), 674 (m), 690 (m), 736 (m), 752 (s), 787 (m), 800 (vs), 847 (vs), 884 (s), 905 (s), 983 (w), 1031 (m), 1070 (m), 1115 (w), 1162 (m), 1183 (m), 1216 (s), 1239 (m), 1307 (w), 1375 (m), 1414 (s), 1441 (m), 1610 (w), 2853 (w), 2912 (m), 2943 (w). **Raman** (633 nm, 10 s, 10 scans, cm^{-1}): $\tilde{\nu}$ = 142 (3), 171 (1), 214 (1), 239 (3), 267 (2), 279 (1), 295 (1), 312 (1), 334 (3), 357 (2), 367 (3), 378 (6), 399 (1), 418 (1), 436 (2), 479 (1), 491 (1), 503 (8), 524 (2), 543 (1), 553 (2), 560 (3), 576 (5), 585 (1), 598 (1), 635 (1), 657 (1), 674 (5), 692 (1), 741 (1), 756 (1), 814 (6), 867 (1), 907 (2), 944 (1), 1006 (1), 1029 (1), 1060 (1), 1090 (10), 1160 (4), 1185 (1), 1223 (2), 1249 (1), 1267 (1), 1288 (5), 1305 (7), 1348 (1), 1354 (1), 1364 (1), 1381 (2), 1419 (1), 1483 (1), 1513 (1), 1578 (1), 1611 (4), 2731 (1), 2856 (1), 2918 (4), 2985 (1), 3012 (1), 3048 (1). **UV-Vis** (benzene, 0.13 mmol/L, molar absorptivity given in brackets in L mol^{-1} cm^{-1}): $\lambda_{max.}$ = 345 nm (7.4×10^{3}). **MS**: Fragments of **4Ter** could not be detected via pos. CI or EI mass spectrometry.

Figure S19: NMR, IR, Raman and UV-Vis spectra of **4Ter** (* = solvent signals, (*) = product signals overlain by solvent signals).

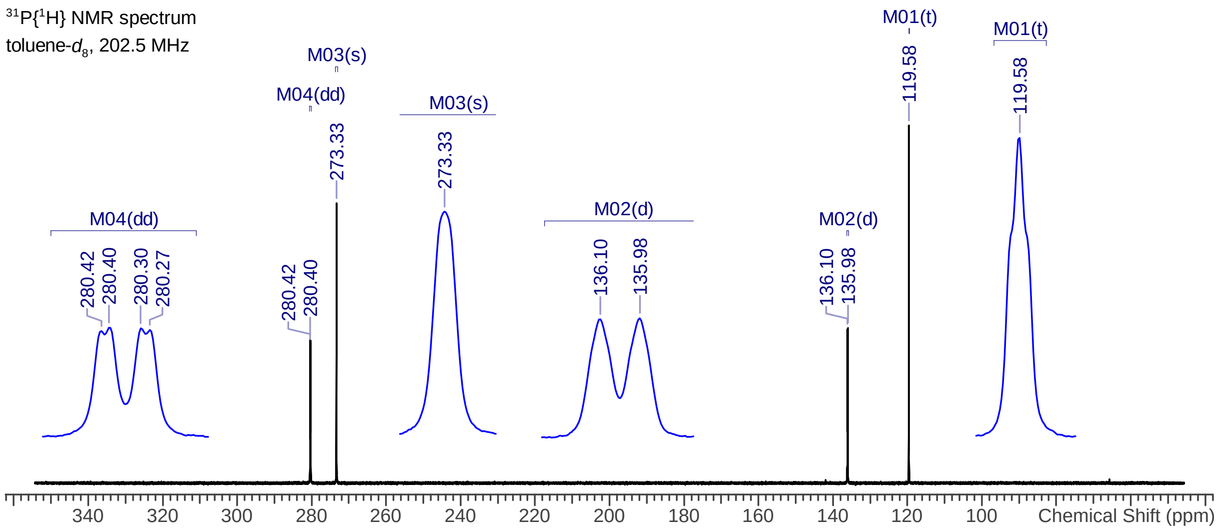

^{1}H NMR spectrum
toluene-d_8, 500.1 MHz

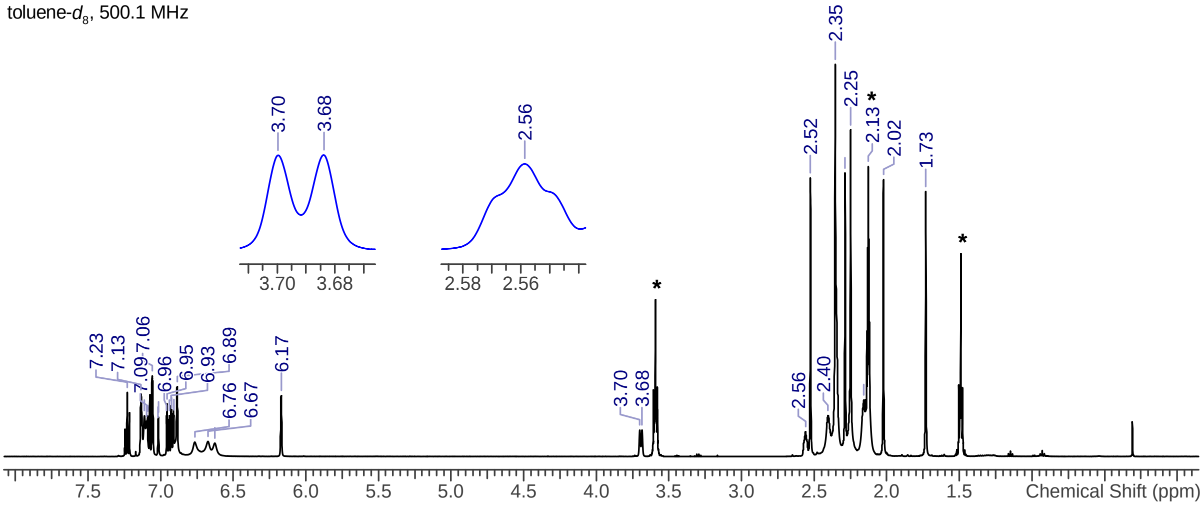

^{1}H NMR spectrum: detailed view, superimposed aromatic C*H* resonances

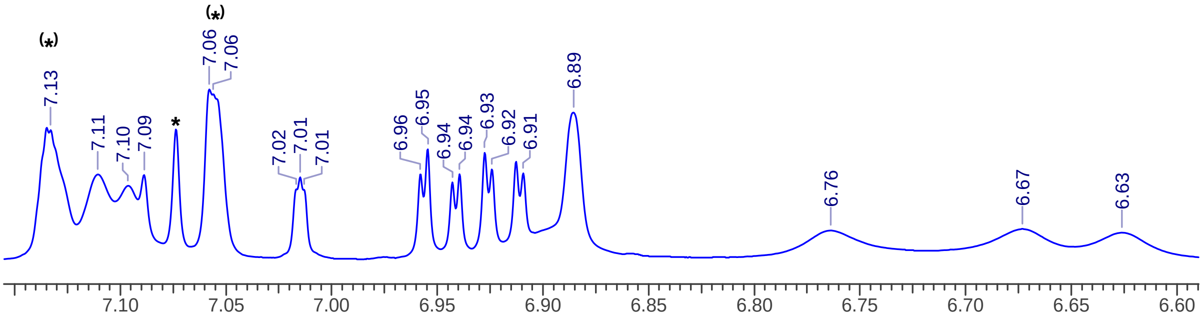

^{1}H NMR spectrum: detailed view, superimposed aliphatic C*H* resonances

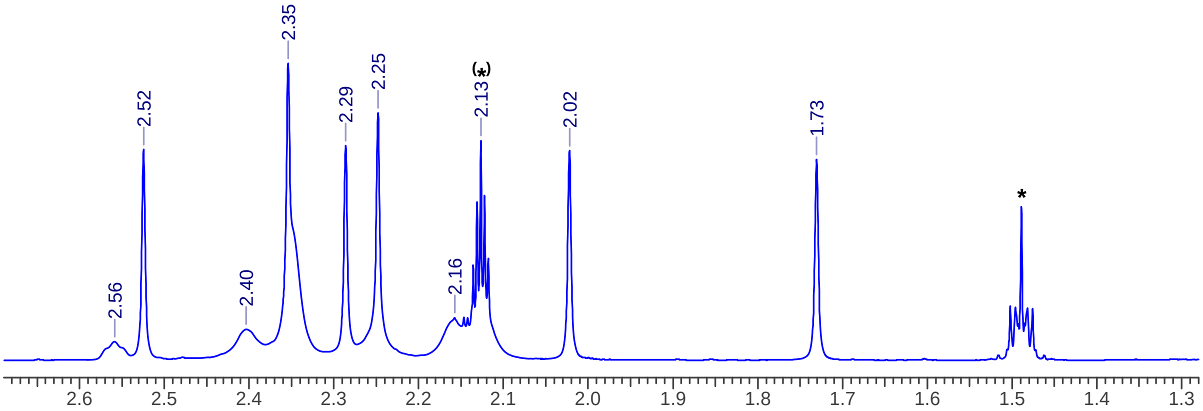

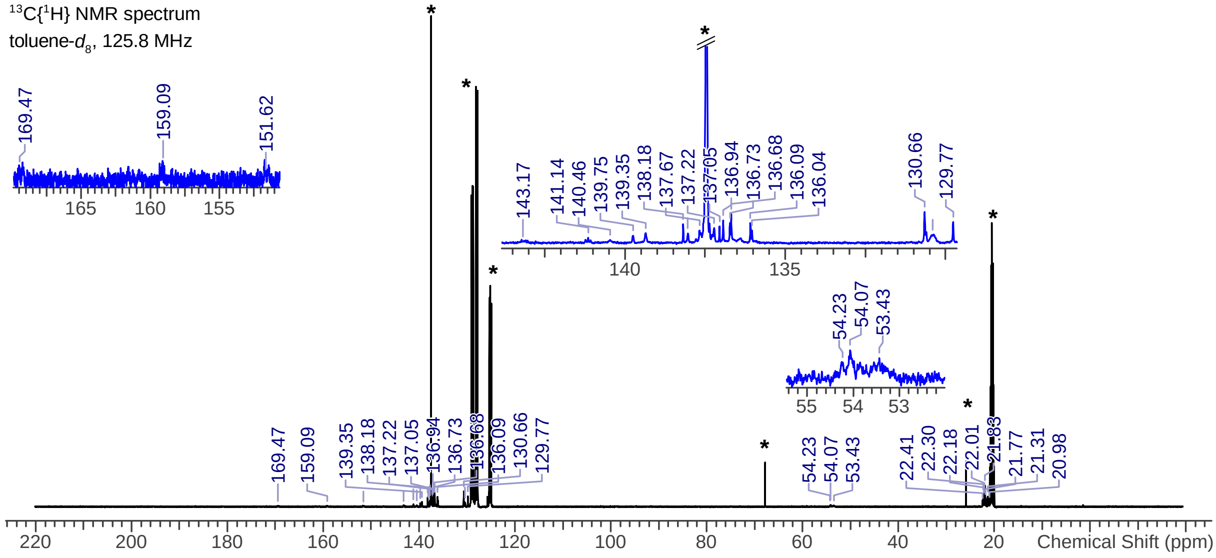
$^{13}C\{^1H\}$ NMR spectrum
toluene-d_8, 125.8 MHz
169.47
159.09
151.62
165
160
155
143.17
141.14
140.46
139.75
139.35
138.18
137.67
137.22
137.05
136.94
136.73
136.68
136.09
136.04
130.66
129.77
140
135
54.23
54.07
53.43
55
54
53
22.41
22.30
22.18
22.01
21.83
21.77
21.31
20.98
220
200
180
160
140
120
100
80
60
40
20
Chemical Shift (ppm)

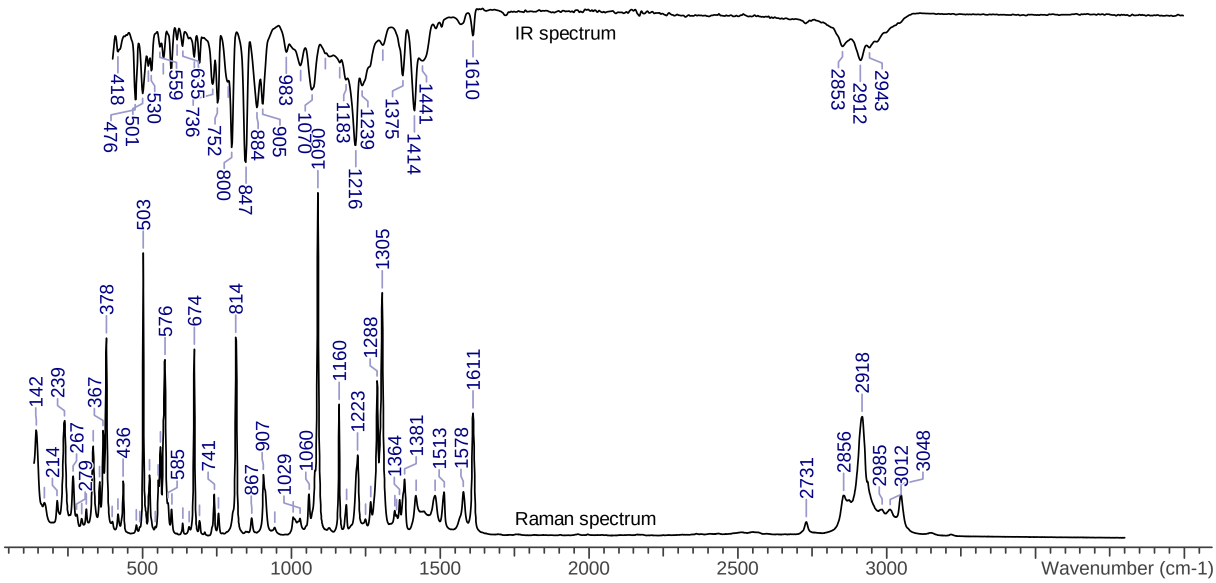
IR spectrum
418
476
501
530
559
635
736
752
800
847
884
905
983
1070
1090
1183
1216
1239
1375
1414
1441
1610
2853
2912
2943
Raman spectrum
142
214
239
267
279
367
378
436
503
576
585
674
741
814
867
907
1029
1060
1160
1223
1288
1305
1364
1381
1513
1578
1611
2731
2856
2918
2985
3012
3048
500
1000
1500
2000
2500
3000
Wavenumber (cm-1)

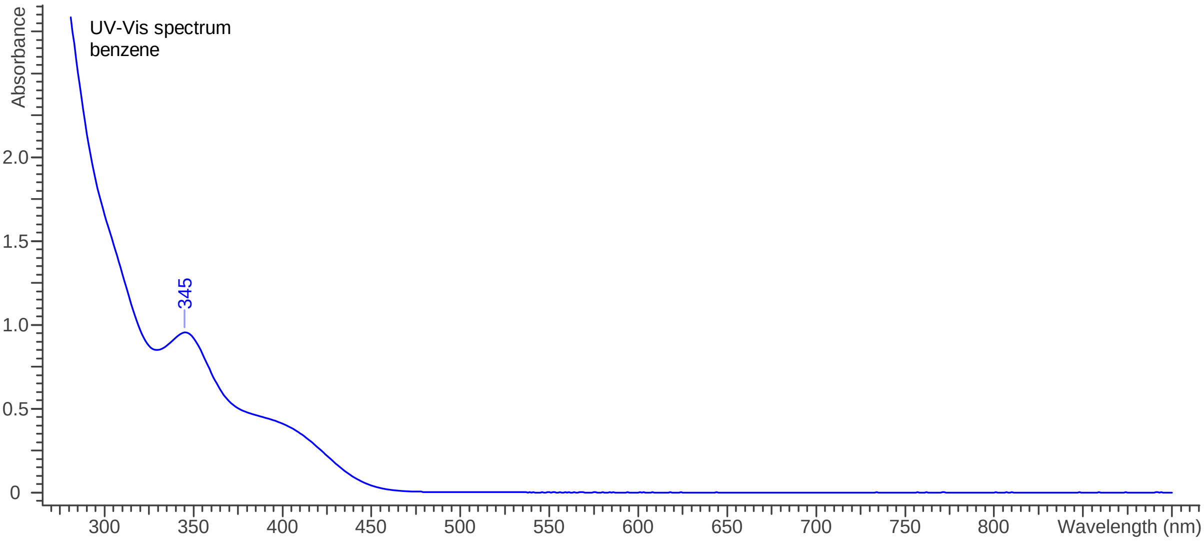
UV-Vis spectrum
benzene
Absorbance
2.0
1.5
1.0
0.5
0
345
300
350
400
450
500
550
600
650
700
750
800
Wavelength (nm)

4.6 Thermal stability of 4Ter

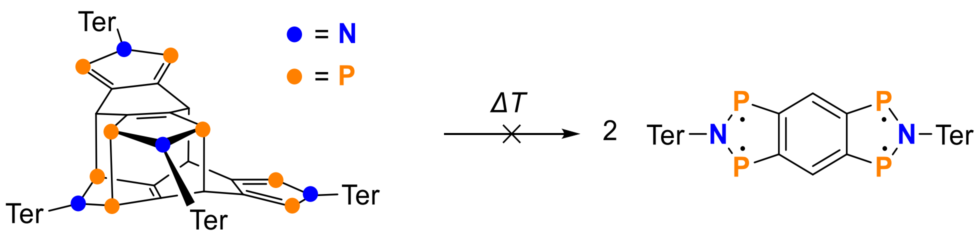

4Ter **1Ter**

4Ter (15 mg, 0.0088 mmol) was dissolved in an NMR tube in toluene-d_8 (0.5 mL). The solution was degassed (freeze-pump-thaw) and subsequently used for temperature-dependent $^{31}P\{^1H\}$ NMR spectroscopy. Initially, the temperature was gradually increased until changes in the NMR spectra were observed at 100 °C. The temperature was then maintained at 100 °C. The measurements show decomposition of the dimer but no formation of the monomer. The decomposition products were not further investigated, especially as insoluble compounds were formed at 100 °C, which explains the decrease of the overall signal intensity.

Figure S20: Temperature dependent $^{31}P\{^1H\}$ NMR spectra. red: **4Ter**, grey: decomposition products.

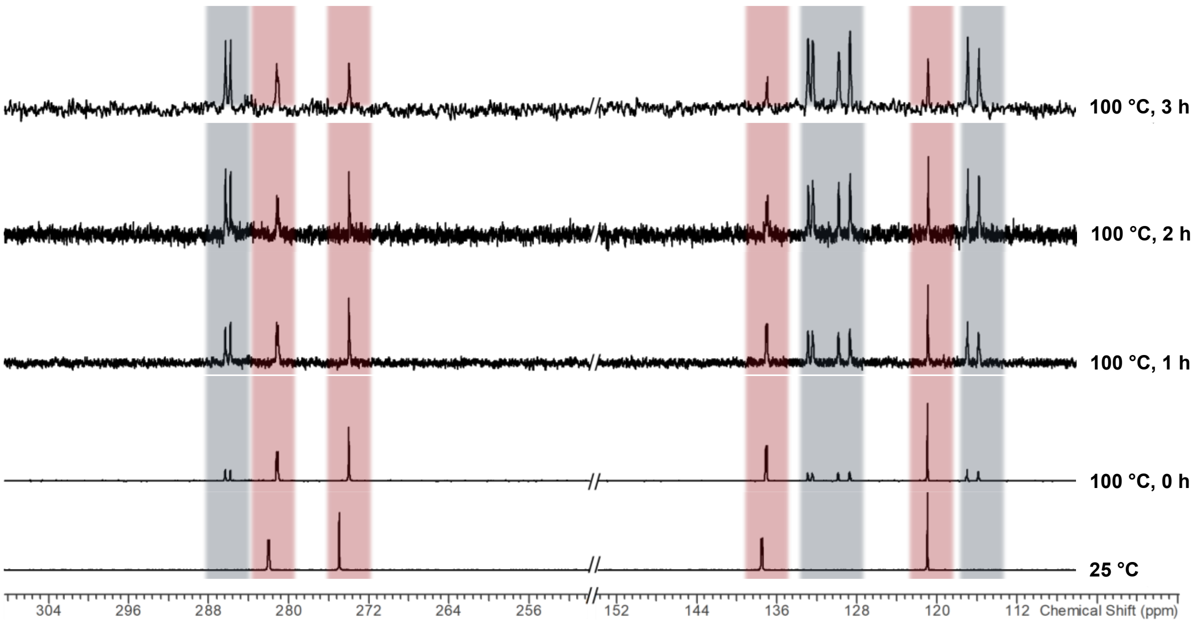

4.7 5Ter

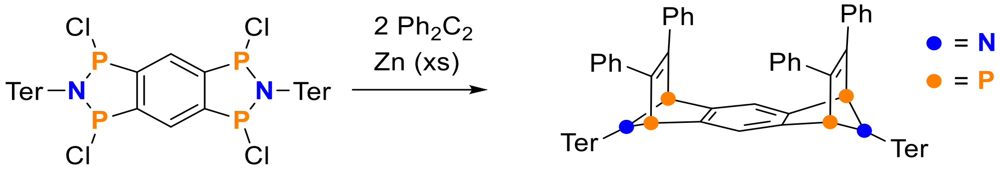

3Ter **5Ter**

Zn dust (2.63 g, 40.2 mmol) was added to a stirred (glass stir bar) solution of **3Ter** (402 mg, 0.404 mmol) and tolan (144 mg, 0.809 mmol) in THF (50 mL). After 2 days Zn dust was removed by filtration. The solvent was removed *in vacuo* and the residue was dried for 2 h at 40 °C (water bath). The residue was suspended in benzene (50 mL) and insoluble material was removed by filtration. The solvent of the filtrate was removed *in vacuo* and replaced by THF (15 mL). The solution was concentrated in the warmth (~60 °C) to incipient crystallization and was cooled to ambient temperature overnight, yielding colorless crystals. The supernatant was removed by syringe. The crystals were dried *in vacuo* (1×10^{-3} mbar) at 45 °C (water bath) for 2 h. Yield 42 mg (0.035 mmol, 9 %).

Single crystals suitable for X-ray diffraction were grown as described in the synthesis procedure.

Mp. 360-370 °C (dec.). **CHN** calc. (found) for $C_{82}H_{72}N_2P_4$ in %: 81.44 (78.04), H 6.00 (6.11), N 2.32 (2.19); deviations in the carbon content probably due to incomplete combustion, repeated measurements did not result in better agreement (single crystals were used for EA). **^{31}P{^{1}H} NMR** (C_6D_6, 202.5 MHz): δ = 100.2 (s). **^{1}H NMR** (C_6D_6, 500.1 MHz): δ = 2.15 (s, 12 H, *p*-C*H*$_3$ (Mes)), 2.25 (s, 24 H, *o*-C*H*$_3$ (Mes)), 6.53 (m, 8 H, *o*-C*H*$_3$ (Ph)), 6.71 (m, 8 H, *m*-C*H*$_3$ (Ph)), 6.77 (m, 4 H, *p*-C*H*$_3$ (Ph)), 6.83 (m, 4 H, *m*-C*H*$_3$ (Mes)), 6.97-7.05 (superimposed signals, 6 H, C*H* (Ter)), 7.35 (s, 2 H, C*H* ($C_6H_2P_4$)). **^{13}C{^{1}H} NMR** (C_6D_6, 125.8 MHz): δ = 21.5 (s, *p*-CH_3 (Mes)), 22.0 (s, *o*-CH_3 (Mes)), 124.8 (s, *p*-CH (Ter)), 127.3 (s, *p*-CH_3 (Ph)), 127.9 (m, CH ($C_6H_2P_4$)) 128.1 (m, *o*-CH (Ph)), 128.7

(s, *m*-CH_3 (Ph)), 128.9 (s, *i*-*C* (Ph)), 129.1 (s, *m*-CH (Mes)), 130.9 (s, *m*-CH (Ter)), 137.1 (s, *o*-*C* (Ter)), 137.5 (s, *i*-*C* (Ph)), 138.0 (s, *i*-*C* (Mes)), 143.3 (m, *i*-*C* (Ter)), 157.1 (m, $C_{quart.}$ ($C_6H_2P_4$)), 163.6 (m, *C* (Ph-C=C-Ph)), *p*-*C* (Mes) could not be found. **IR** (ATR, 32 scans, cm^{-1}): $\tilde{\nu}$ = 404 (m), 435 (s), 445 (s), 480 (s), 493 (m), 511 (m), 532 (m), 559 (m), 594 (m), 649 (m), 680 (s), 690 (vs), 730 (s), 752 (m), 765 (m), 798 (s), 831 (m), 847 (s), 878 (s), 905 (w), 925 (m), 961 (w), 1029 (m), 1070 (m), 1080 (w), 1237 (m), 1253 (m), 1371 (m), 1416 (s), 1439 (m), 1457 (w), 1482 (w), 1490 (w), 1573 (w), 1612 (w), 2853 (w), 2912 (w), 2941 (w), 2964 (w), 3013 (w), 3053 (w), 3075 (vw), 3096 (vw). **Raman** (633 nm, 10 s, 10 scans, cm^{-1}): $\tilde{\nu}$ = 142 (2), 178 (1), 210 (3), 242 (2), 263 (1), 273 (1), 309 (1), 322 (1), 331 (1), 359 (1), 404 (1), 424 (1), 486 (1), 520 (4), 560 (3), 575 (5), 609 (1), 627 (1), 667 (1), 700 (2), 739 (1), 767 (1), 839 (1), 1000 (9), 1084 (2), 1157 (2), 1171 (2), 1189 (1), 1258 (2), 1282 (1), 1306 (3), 1378 (1), 1421 (1), 1441 (1), 1483 (1), 1492 (1), 1501 (1), 1555 (10), 1574 (2), 1594 (6), 1613 (2), 2508 (1), 2555 (1), 2727 (1), 2856 (1), 2916 (2), 3016 (1), 3061 (3). **MS**: Fragments of **5Ter** could not be detected via pos. CI mass spectrometry.

Figure S21: NMR, IR and Raman spectra of **5Ter** (solvent signals indicated by asterisks).

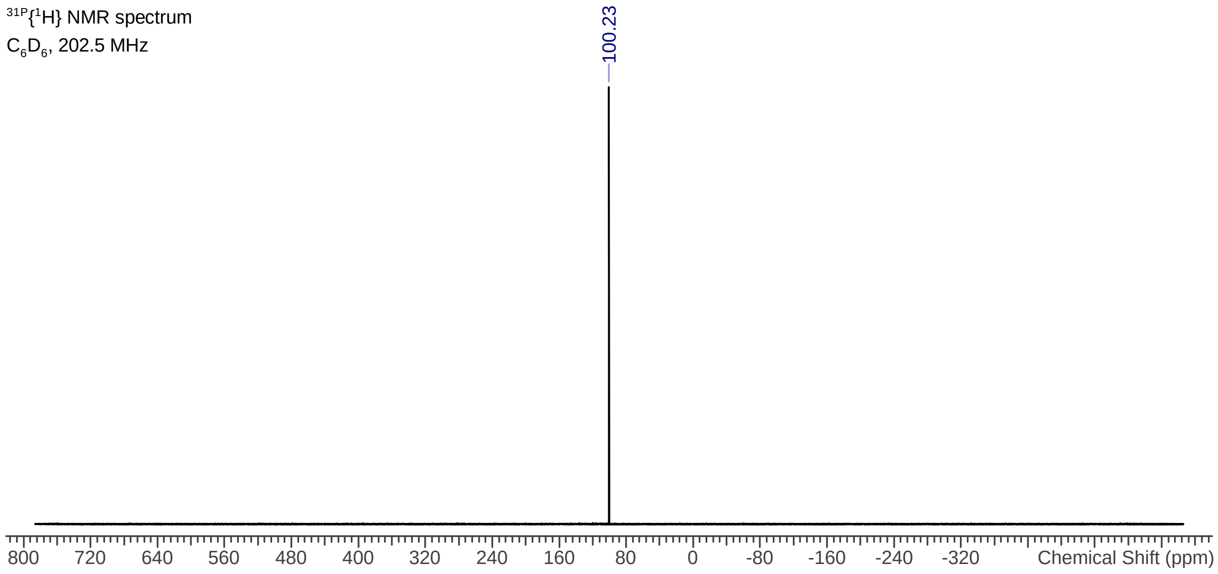

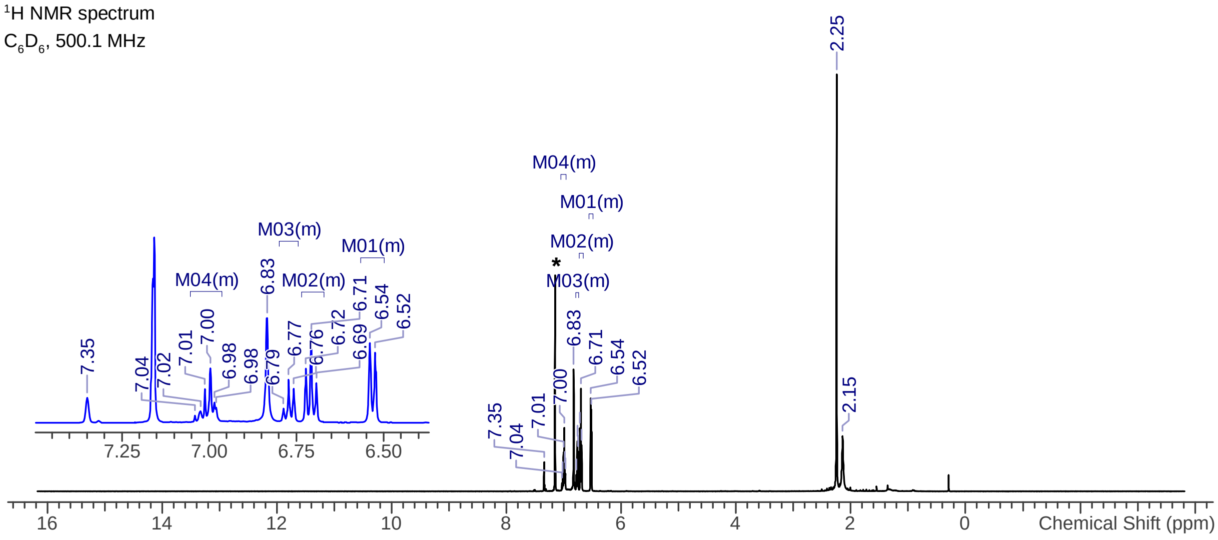
¹H NMR spectrum
C6D6, 500.1 MHz
2.25
2.15
7.35
7.04
7.01
7.00
6.83
6.71
6.54
6.52
M04(m)
M01(m)
M02(m)
M03(m)
*
7.35
7.04
7.02
7.01
7.00
6.98
6.98
6.83
6.79
6.77
6.76
6.72
6.71
6.69
6.54
6.52
7.25
7.00
6.75
6.50
Chemical Shift (ppm)

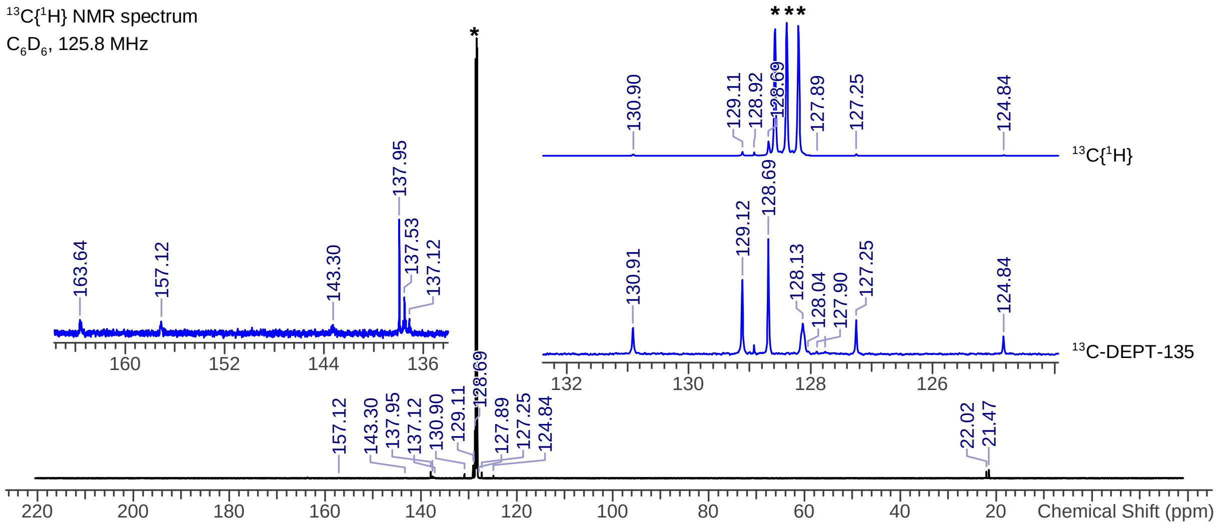
13C{1H} NMR spectrum
C6D6, 125.8 MHz
*
***
130.90
129.11
128.92
128.69
127.89
127.25
124.84
13C{1H}
130.91
129.12
128.69
128.13
128.04
127.90
127.25
124.84
13C-DEPT-135
163.64
157.12
143.30
137.95
137.53
137.12
157.12
143.30
137.95
137.12
130.90
129.11
128.69
127.89
127.25
124.84
22.02
21.47
Chemical Shift (ppm)

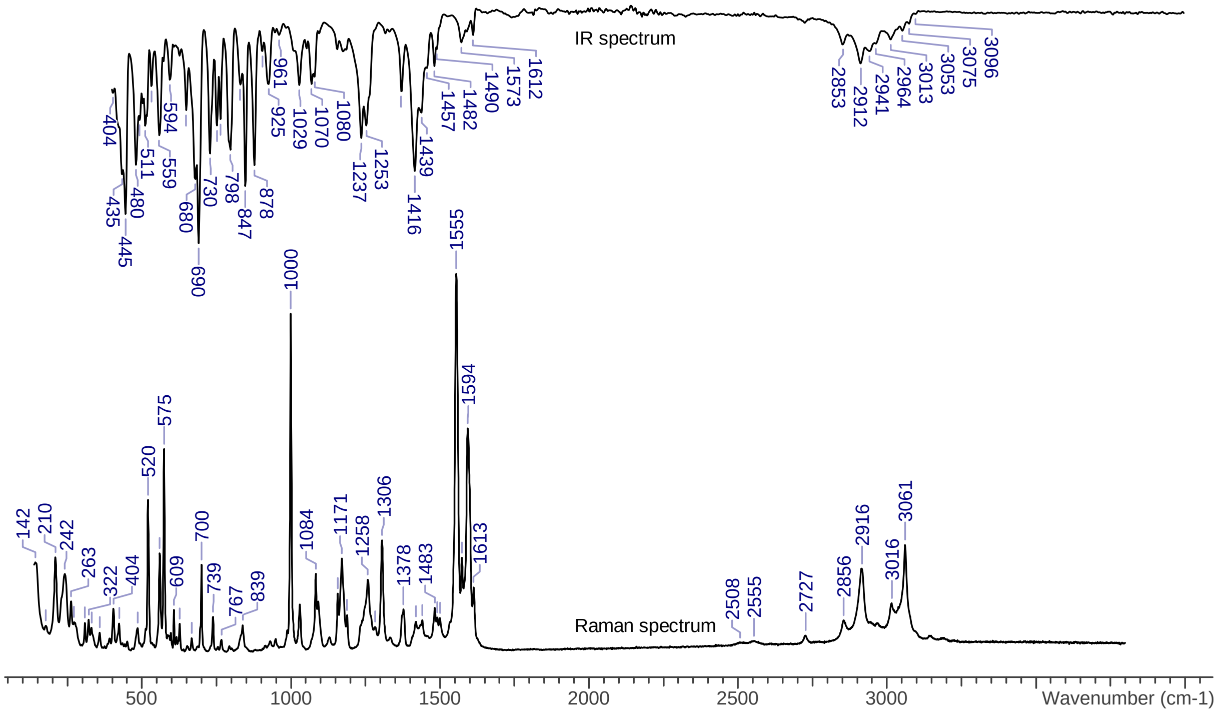
IR spectrum
404
435
445
480
511
559
594
680
690
730
798
847
878
925
961
1029
1070
1080
1237
1253
1416
1439
1457
1482
1490
1573
1612
2853
2912
2941
2964
3013
3053
3075
3096
Raman spectrum
142
210
242
263
322
404
520
575
609
700
739
767
839
1000
1084
1171
1258
1306
1378
1483
1555
1594
1613
2508
2555
2727
2856
2916
3016
3061
Wavenumber (cm-1)

4.8 1EMind

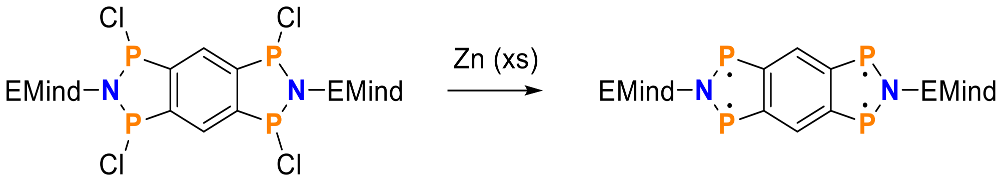

3EMind **1EMind**

Zn dust (5.775 g, 88.33 mmol) was added to a stirred (glass stir bar) solution of **3EMind** (4.500 g, 4.416 mmol) in THF (100 mL). The solution turned green immediately and became more intense as the reaction time proceeded. The reaction was traced by $^{31}P\{^1H\}$ NMR spectroscopy to ensure complete conversion. After 6 days, unreacted Zn dust was removed by filtration and the residue was washed with THF (2 × 50 mL). The solvent of the filtrate was removed *in vacuo* (1×10^{-3} mbar) and the remaining solids were dried for 2 h at 50 °C (water bath). The residue was extracted with benzene (200 mL) by filtration in the warmth (~60 °C) over a plug of silica. The extraction was repeated twice with benzene (2×150 mL) until the residue was yellow. The green filtrate was concentrated in the warmth (~80 °C) to incipient crystallization and was cooled to ambient temperature overnight yielding dark green crystals. The supernatant was removed by cannula transfer and was concentrated to get three further crops of crystals. The crystals were dried *in vacuo* (1×10^{-3} mbar) at 50 °C (water bath) for 2 h. Yield 2.595 g (2.959 mmol, 67%).

Single crystals suitable for X-ray diffraction were grown as described in the synthesis procedure.

Mp. 365 °C (dec.). **CHN** calc. (found) for $C_{100}H_{104}N_4P_8\cdot1.6$ THF in %: 74.11 (73.58), H 8.73 (8.57), N 3.17 (3.21). **^{31}P{^{1}H} NMR** (THF-d_8, 202.5 MHz): δ = 289.2 (s). **^{1}H NMR** (THF-d_8, 300.1 MHz): δ = 0.88 (dd, $^3J(^1H,^1H)$=7.4 Hz, $^3J(^1H,^1H)$=7.4 Hz, 24 H, C*H*$_3$ (Et)), 1.17 (s, 24 H, C*H*$_3$ (Me)), 1.69 (m, 8 H, C(*H*)H (Et)), 1.87 (m, 8 H, C(*H*)H (Et)),1.89 (s, 8 H, C*H*$_2$ (Cyclopent.)), 6.92 (s, 2 H, C*H* (EMind)), 9.66 (m, 2 H, C*H* ($C_6H_2P_4$)). **^{13}C{^{1}H} NMR** (THF-

d_8, 125.8 MHz): δ = 9.6 (s, CH_3 (Et)), 32.4 (s, CH_3 (Me)), 33.5 (s, CH_2 (Et)), 45.4 (s, CMe_2), 48.5 (s, CEt_2), 54.3 (CH_2 (Cyclopent.)), 121.5 (s, CH (EMind)), 128.2 (m, CH ($C_6H_2P_4$)), 138.9 (m, C_{ipso} (EMind)), 146.5 (s, C_{ortho} (EMind)), 151.0 (s, C_{meta} (EMind)), 156.4 (m, $C_{quat.}$ ($C_6H_2P_4$)). **IR** (ATR, 32 scans, cm^{-1}): $\tilde{\nu}$ = 414 (w), 441 (w), 449 (m), 462 (w), 524 (w), 614 (w), 633 (w), 676 (m), 740 (w), 767 (w), 791 (m), 847 (m), 886 (m), 913 (m), 940 (w), 954 (m), 1014 (m), 1031 (m), 1049 (m), 1105 (m), 1134 (w), 1171 (m), 1210 (s), 1263 (m), 1276 (m), 1303 (s), 1340 (m), 1360 (s), 1379 (s), 1416 (m), 1459 (s), 1558 (m), 1602 (w), 2855 (s), 2875 (s), 2931 (s), 2960 (vs). **Raman** 532 nm, 20 s, 20 scans, cm^{-1}): $\tilde{\nu}$ = 296 (1), 377 (1), 494 (3), 528 (1), 557 (1), 623 (1), 692 (1), 739 (4), 893 (7), 1016 (2), 1046 (2), 1074 (2), 1096 (3), 1238 (4), 1294 (1), 1312 (1), 1404 (2), 1526 (10), 1704 (1), 1823 (2), 2132 (1), 2146 (1), 2927 (2). **UV-Vis** (benzene, 0.055 mmol/L, molar absorptivity given in brackets in L mol^{-1} cm^{-1}): $\lambda_{max.}$ = 396 (16.9×10^3), 667 nm (11.8×10^3). **MS** (CI, pos., isobutene, m/z): 879 $[M+H]^+$, 878 $[M+H]^+$, 877 $[M]^+$.

Figure S22: NMR, IR, Raman and UV-Vis spectra of **1EMind** (solvent signals indicated by asterisks).

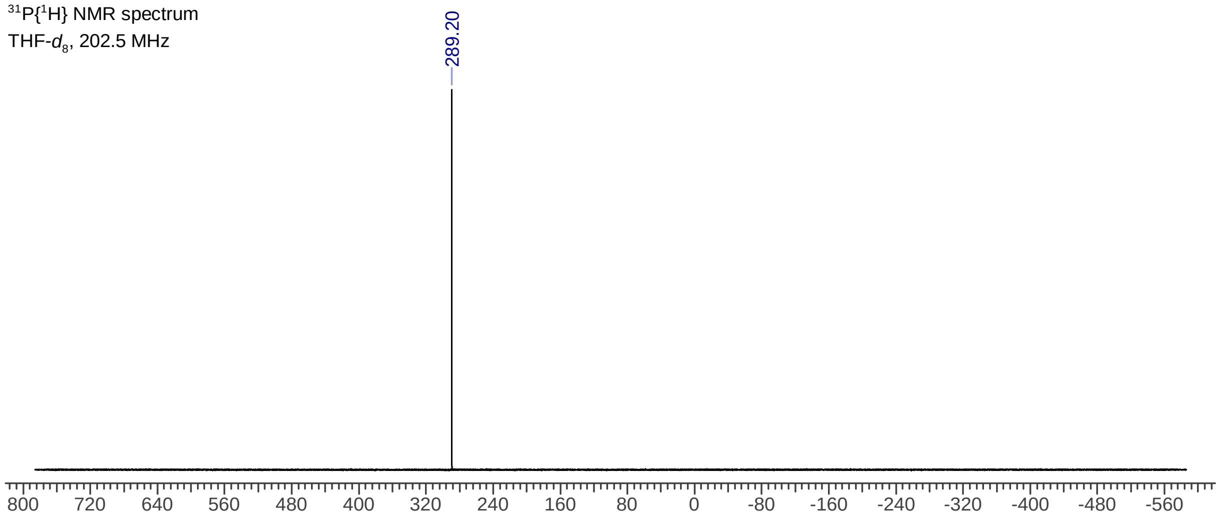

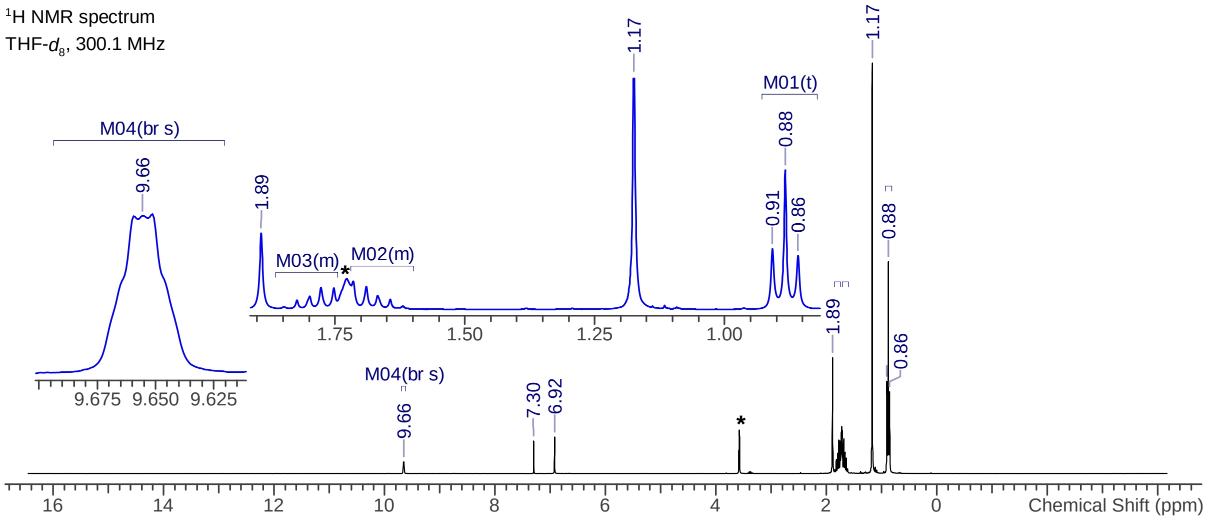

detailed zoom into the multipletts in the ^{1}H NMR spectra at diffrent spectrometer frequencies

<u>300.1 MHz:</u>

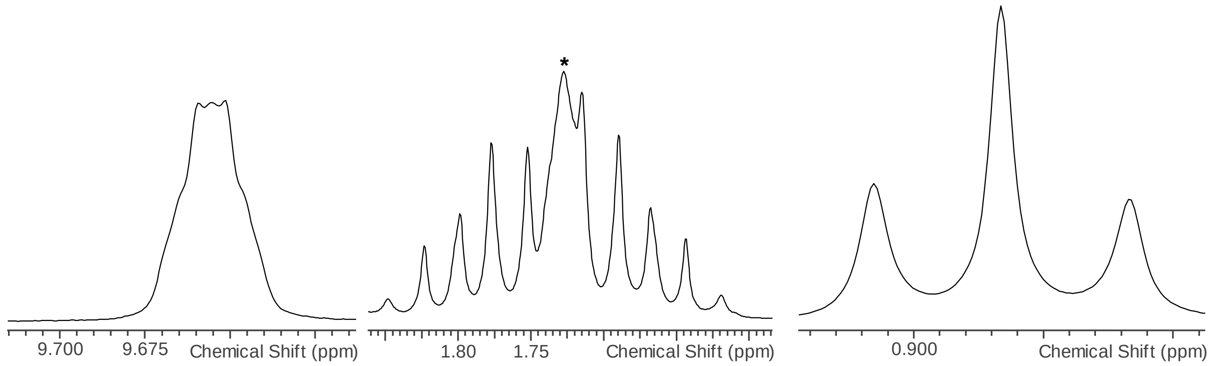

<u>500.1 MHz:</u>

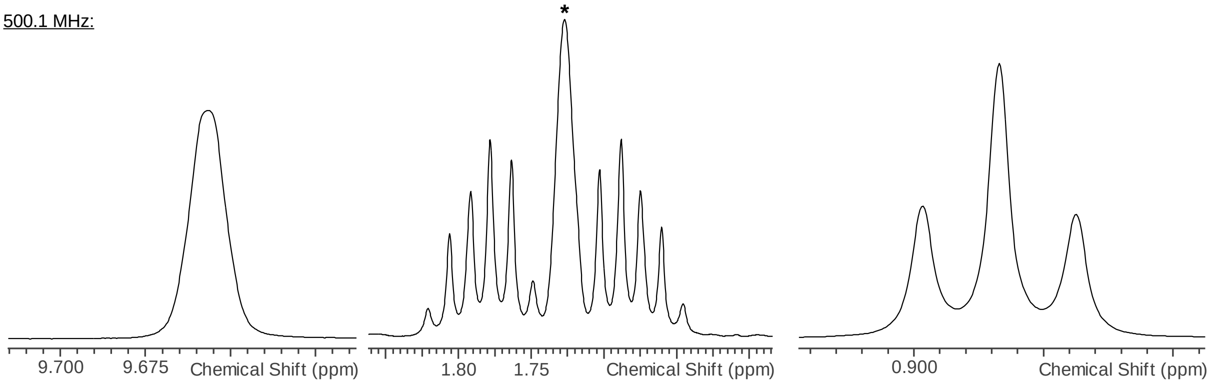

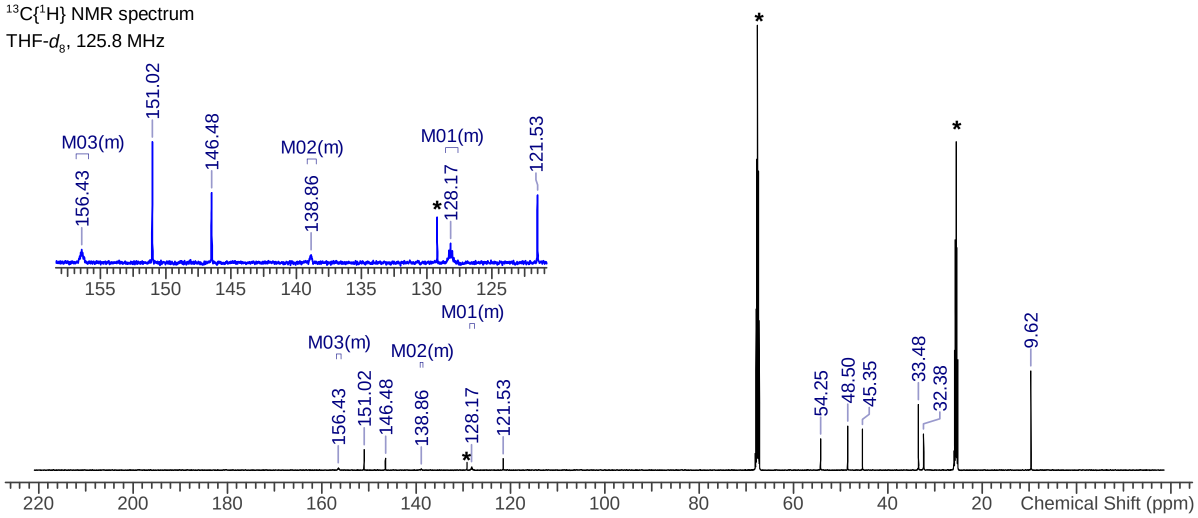

$^{13}C\{^1H\}$ NMR spectrum
THF-d_8, 125.8 MHz
M03(m)
M02(m)
M01(m)
156.43
151.02
146.48
138.86
128.17
121.53
54.25
48.50
45.35
33.48
32.38
9.62
Chemical Shift (ppm)

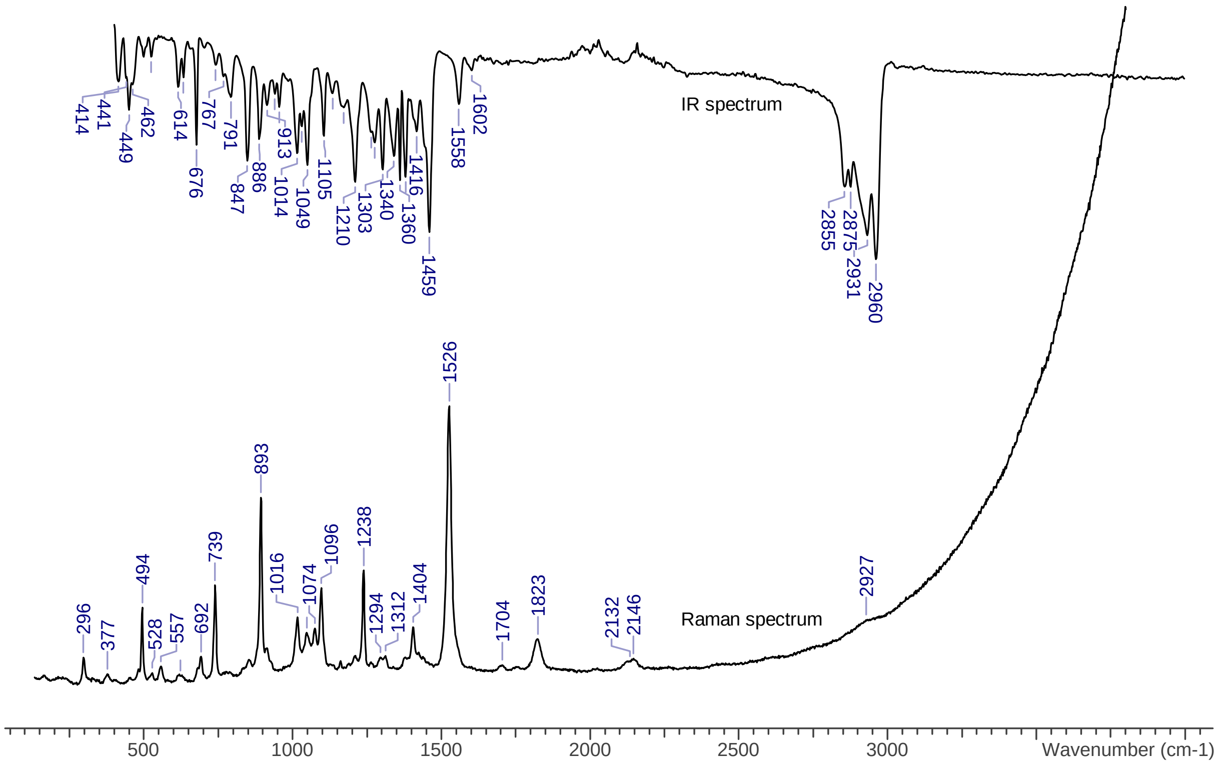

IR spectrum
414
441
449
462
614
676
767
791
847
886
913
1014
1049
1105
1210
1303
1340
1360
1416
1459
1558
1602
2855
2875
2931
2960
Raman spectrum
296
377
494
528
557
692
739
893
1016
1074
1096
1238
1294
1312
1404
1526
1704
1823
2132
2146
2927
Wavenumber (cm-1)

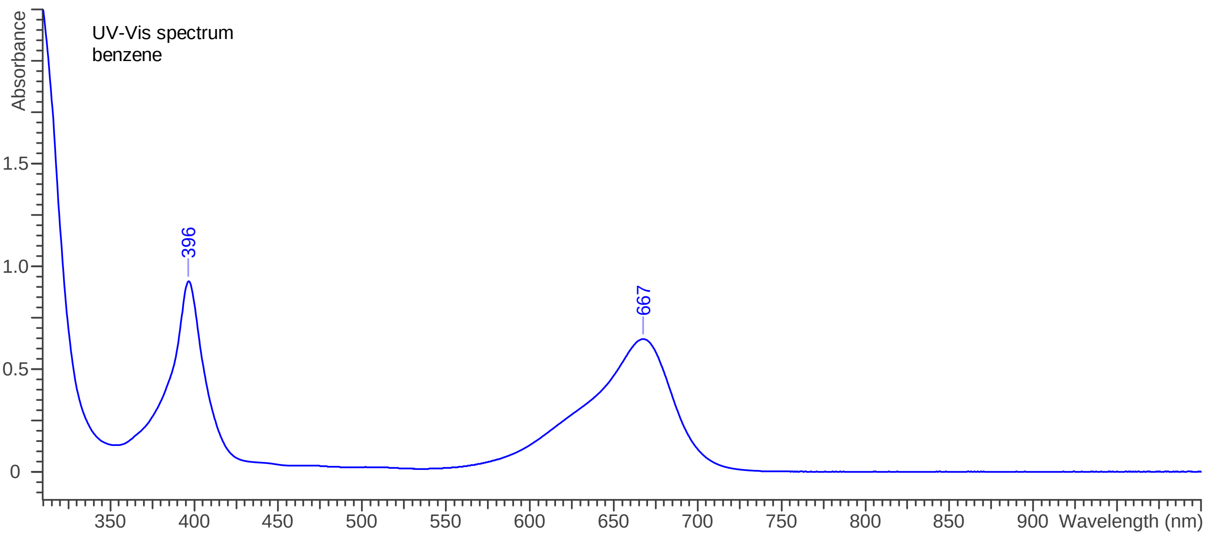
UV-Vis spectrum
benzene
Absorbance
396
667
0
0.5
1.0
1.5
350
400
450
500
550
600
650
700
750
800
850
900
Wavelength (nm)

4.9 6EMind

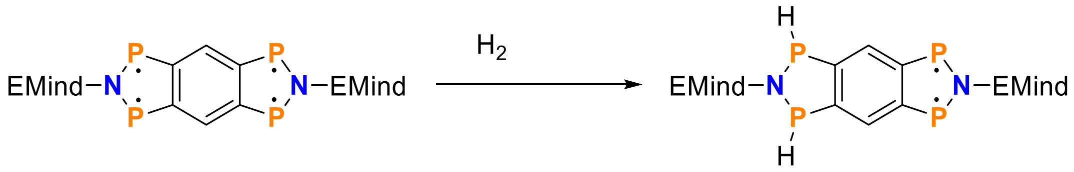

1EMind **6EMind**

Procedure 1

H_2 (1 atm) was added to a degassed solution of **1EMind** (150 mg, 0.171 mmol) in THF (2 mL) in a 50 mL Schlenk tube. Within 4 weeks the solution turned yellow, and a yellow precipitate was formed. The solvent was evaporated and the solid residue was recrystallized from hot (100 °C) 1,2-dichlorobenzene (3 mL). The solution was cooled to ambient temperature overnight, yielding yellow crystals of **6EMind**. The supernatant was removed by syringe and was discarded. The yellow crystals were dried *in vacuo* (1×10^{-3} mbar) at 40 °C (water bath) over a period of 2 h yielding **6EMind 2$C_6H_4Cl_2$**. Yield: 45 mg (0.038 mmol, 22%).

Single crystals suitable for X-ray diffraction were grown as described in the synthesis procedure. The analytical data were collected from the product obtained in Procedure 1.

Mp. 120 °C (dec.). **CHN** calc. (found) for $C_{100}H_{104}N_4P_8\cdot2\ C_6H_4Cl_2$ in %: 67.57 (67.65), H 7.39 (7.36), N 2.39 (2.63). **$^{31}P\{^1H\}$ NMR** (THF-d_8, 202.5 MHz): δ = 58.6 (s, 2 P, *P*H), 286.8 (s, 2 P, *P*·). **^{31}P NMR** (THF-d_8, 101.3 MHz): δ = 58.6 (m[1], 2 P, *P*H), 286.8 (s, 2 P, *P*·). **^1H NMR** (THF-d_8, 500.1 MHz): δ = 0.79 (dd, $^3J(^1H, ^1H)$=7.4 Hz, $^3J(^1H, ^1H)$=7.4 Hz, 6 H, CH_3 (Et)), 0.82 - 0.90 (18 H, superimposed signals of 3 different CH_3 groups (Et)), 1.10 (s, 6 H, CH_3 (Me)), 1.16 (s, 6 H, CH_3 (Me)), 1.24 (s, 6 H, CH_3 (Me)), 1.51 - 1.82 (16 H, superimposed signals of 8 different C(*H*)H and C(H)*H* protons (Et)), 1.55 (s, 6 H, CH_3

[1] For simulated multiplet and coupling constants see chap. 5.1 (p. 74).

(Me)), 1.77 (s, 2 H, CH_2 (cyclopent.)), 1.87 (s, 2 H, CH_2 (cyclopent.)), 1.90 (s, 2 H, CH_2 (cyclopent.)), 1.91 (s, 2 H, CH_2 (cyclopent.)), 6.33 (m[1], 2 H, P*H*), 6.71 (s, 1 H, C*H* (EMind)), 6.91 (s, 1 H, C*H* (EMind)), 7.27 (m, 2 C*H* (dichlorobenzene)), 7.51 (m, 2 C*H* (dichlorobenzene)), 8.74 (m, 2 H, C*H* (EMind)). **^{13}C{^{1}H} NMR** (THF-d$_8$, 125.8 MHz): δ = 9.6 (s, 2×CH_3 (Et)), 9.6 (s, 6×CH_3 (Et)), 32.0 (t, $^5J(^{13}C, ^{31}P)$ = 6 Hz, 2×CH_3 (Me)), 32.4 (m, 6×CH_3 (Me)), 33.4 (s, 2×CH_2 (Et)), 33.5 (s, 2×CH_2 (Et)), 33.6 (s, 2×CH_2 (Et)), 33.7 (s, 2×CH_2 (Et)), 44.3 (s, 1×*C*Me_2), 44.5 (s, 1×*C*Me_2), 45.1 (s, 2×*C*Me_2), 48.6 (s, 2×*C*Et_2), 48.8 (s, 1×*C*Et_2), 49.2 (s, 1×*C*Et_2), 54.1 (s, 1×CH_2 (cyclopent.)), 54.2 (s, 1×CH_2 (cyclopent.)), 54.3 (s, 1×CH_2 (cyclopent.)), 54.9 (s, 1×CH_2 (cyclopent.)), 120.0 (s, 1×*C*H (EMind)), 121.5 (s, 1×*C*H (EMind)), 125.9 (m, 2×*C*H ($C_6H_2P_4$)), 129.2 (s, 2×*C*H (dichlorobenzene)), 131.7 (s, 2×*C*H (dichlorobenzene)), 133.3 (s, 2×$C_{quart.}$ (dichlorobenzene)), 138.4 (t, $^2J(^{13}C, ^{31}P)$ = 14 Hz, 1×C_{ipso} (EMind)), 141.1 (t, $^2J(^{13}C, ^{31}P)$ = 22 Hz, 1×C_{ipso} (EMind)), 142.4 (m, 2×$C_{quart.}$ ($C_6H_2P_4$)), 146.7 (s, 1×C_{ortho} (EMind)), 146.7 (s, 1×C_{ortho} (EMind)), 148.8 (bs, 1×C_{ortho} (EMind)), 150.7 (s, 1×C_{meta} (EMind)), 151.1 (s, 1×C_{meta} (EMind)), 151.1 (s, 1×C_{meta} (EMind)), 151.1 (s, 1×C_{ortho} (EMind)), 151.9 (s, 1×C_{meta} (EMind)), 161.5 (m, 2×$C_{quart.}$ ($C_6H_2P_4$)). **IR** (ATR, 32 scans, cm^{-1}): $\tilde{\nu}$ = 416 (m), 437 (m), 449 (m), 462 (m), 501 (w), 517 (m), 528 (m), 616 (s), 660 (m), 748 (vs), 789 (s), 820 (s), 835 (m), 853 (s), 874 (vs), 886 (s), 897 (m), 913 (s), 930 (vs), 956 (w), 983 (m), 1016 (m), 1035 (s), 1057 (m), 1088 (m), 1097 (m), 1107 (m), 1127 (s), 1160 (m), 1169 (m), 1181 (m), 1187 (m), 1212 (s), 1231 (s), 1261 (s), 1301 (m), 1327 (m), 1360 (m), 1377 (m), 1408 (m), 1418 (m), 1435 (m), 1455 (s), 1558 (w), 2168 (w), 2193 (w), 2859 (m), 2873 (m), 2921 (s), 2933 (s), 2958 (s). **Raman** (532 nm, 10 s, 15 scans, cm^{-1}): $\tilde{\nu}$ = 153 (3), 201 (2), 242 (2), 301 (2), 466 (2), 491 (3), 500 (3), 516 (3), 529 (4), 563 (3), 617 (3), 660 (1), 696 (3), 742 (1), 834 (1), 859 (1), 876 (1), 935 (1), 1038 (2), 1068 (4), 1088 (6), 1108 (2), 1129 (2), 1234 (1), 1312 (4), 1408 (1), 1452 (10), 1464 (3), 1571 (1), 2173 (1), 2195 (2), 2861 (2), 2878 (3), 2906 (4), 2937 (5), 2960 (3), 3012 (3), 3070 (1). **MS** (CI, pos., isobutene, m/z): 883-880 $[M+H]^+$, 879 $[M]^+$, 878 $[M-H]^+$, 877 $[M-H_2]^+$.

Figure S23: NMR, IR and Raman spectra of **6EMind** (solvent signals indicated by asterisks).

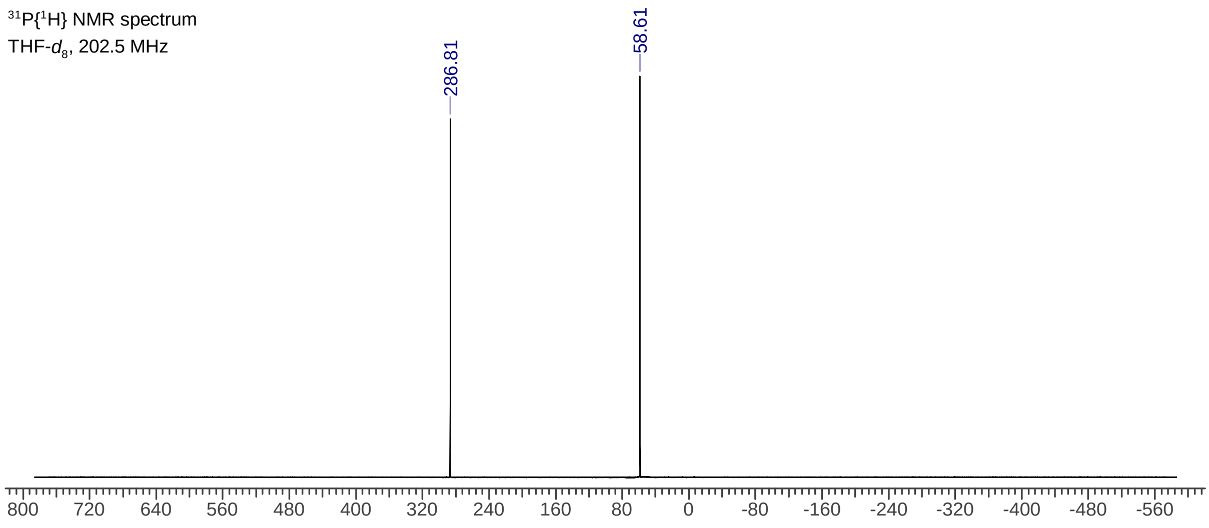

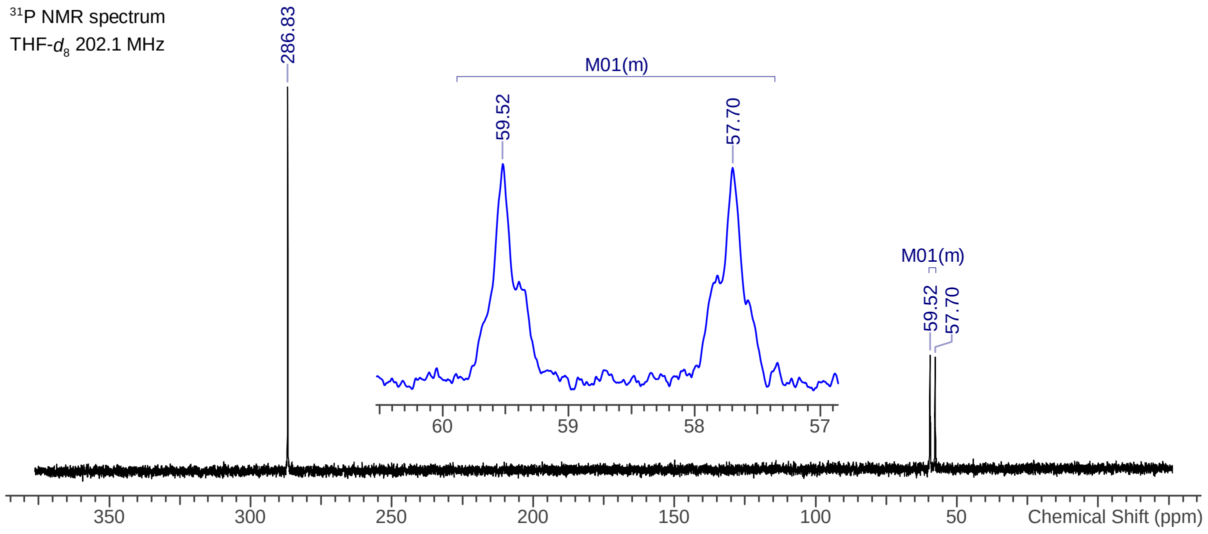

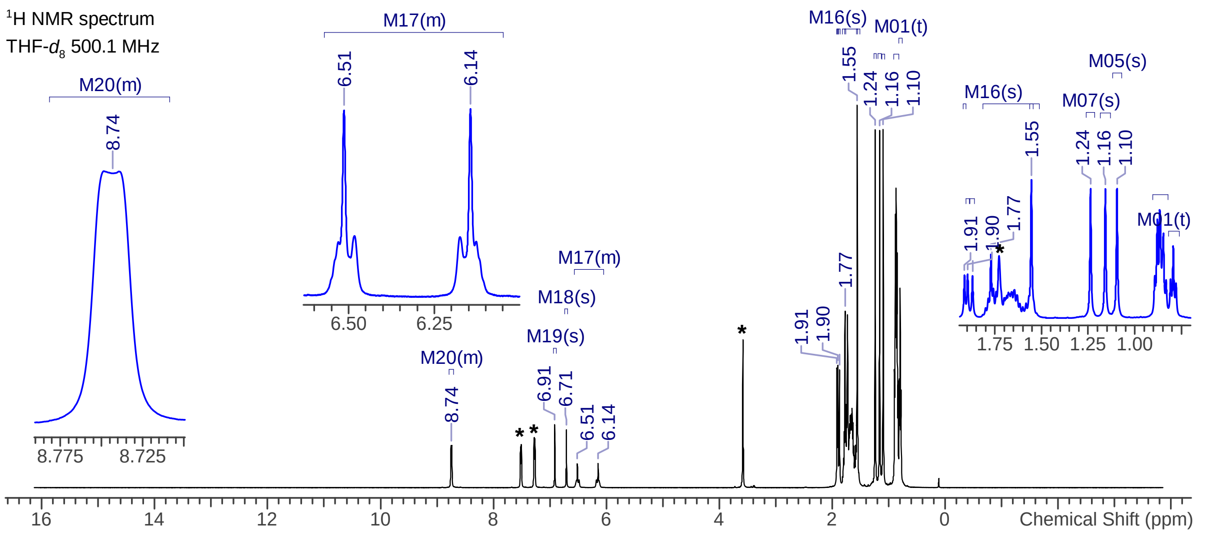

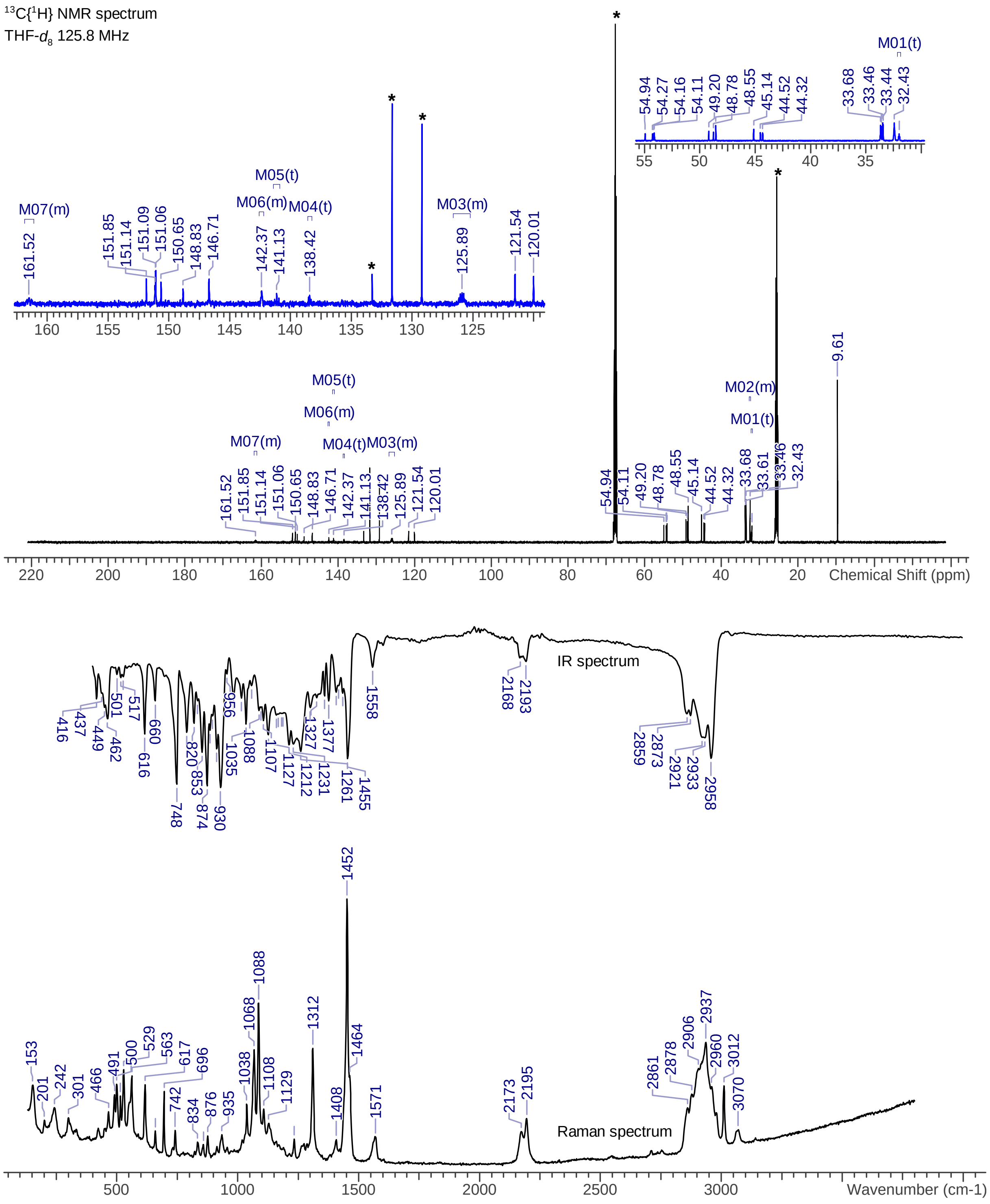

Procedure 2

A significantly faster reaction conversion could be achieved by increasing the temperature and pressure.

For this purpose, a solution of **1EMind** (200 mg, 0.228 mmol) in toluene (20 mL) was placed in a 50 mL autoclave with glass inlet and teflon stir bar was pressurized with H_2

(10 bar). The reaction mixture was warmed to 65 °C (heating block) and was stirred for 2.5 h. The reaction mixture was cooled to ambient temperature and the H_2 pressure was released. The yellow reaction mixture was transferred to a Schlenk flask. The solvent was removed *in vacuo* and the yellow residue was dried *in vacuo* (1×10^{-3} mbar) at 50 °C (water bath) over a period of 40 min yielding crude **6EMind 0.5 toluene**. Yield: 155 mg (168 mmol,73%).

The product is pure **6EMind** apart from 1% ***syn*-7EMind** and 1% ***anti*-7EMind** (determined by $^{31}P\{^{1}H\}$ NMR spectroscopy).

$^{31}P\{^{1}H\}$ NMR (C_6D_6, 121.5 MHz): δ = 57.6 (s, 4 P, *P*H (***anti*-7EMind**, 1%)), 58.2 (s, 2 P, *P*H (**6EMind**, 98%)), 58.8 (s, 4 P, *P*H (***syn*-7EMind**, 1%)), 286.6 (s, 2 P, *P*·(**6EMind**, 98%). **^{1}H NMR** (C_6D_6, 300.1 MHz): δ = 0.75 - 92 (24 H, superimposed signals of 4 different C*H*$_3$ groups (Et)), 1.21 (s, 6 H, C*H*$_3$ (Me)), 1.26 (s, 6 H, C*H*$_3$ (Me)), 1.44 (s, 6 H, C*H*$_3$ (Me)), 1.46 - 1.81 (16 H, superimposed signals of 8 different C(*H*)H and C(H)*H* protons (Et)), 1.68 (s, 2 H, C*H*$_2$ (cyclopent.)), 1.70 (s, 6 H, C*H*$_3$ (Me)), 1.74 (s, 2 H, C*H*$_2$ (cyclopent.)), 1.79 (s, 2 H, C*H*$_2$ (cyclopent.)), 1.89 (s, 2 H, C*H*$_2$ (cyclopent.)), 6.49 (m, 2 H, P*H*), 6.93 (s, 1 H, C*H* (EMind)), 6.80 (s, 1 H, C*H* (EMind)), 8.29 (m, 2 H, C*H* (EMind)).

Figure S24: NMR spectra of **6EMind** (solvent signals indicated by asterisks).

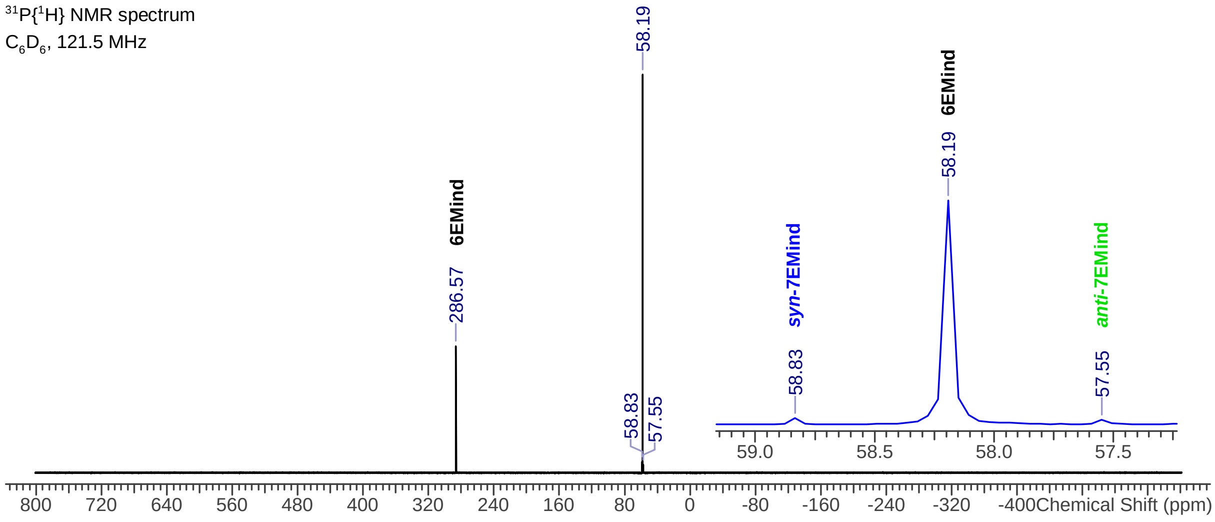

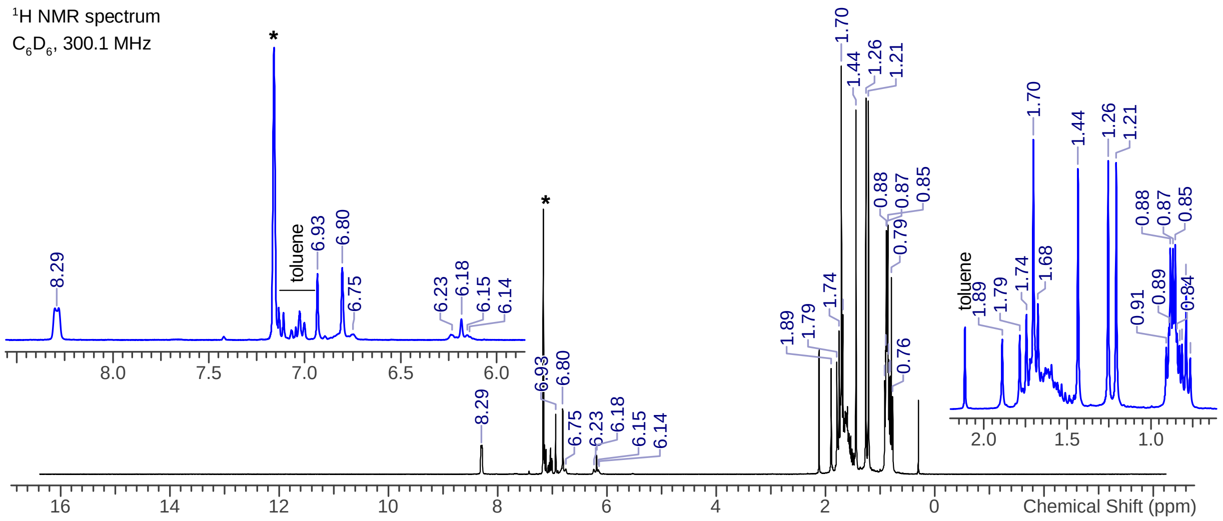

1H NMR spectrum
C6D6, 300.1 MHz
toluene
Chemical Shift (ppm)

4.10 7EMind

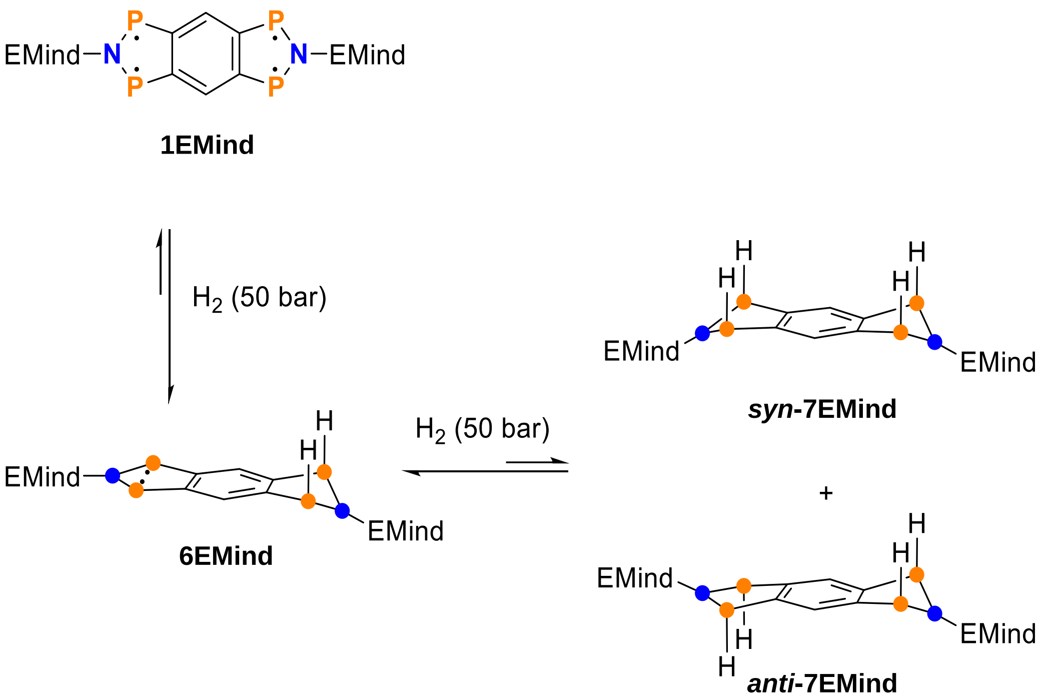

A solution of **1EMind** (50 mg, 0.057 mmol) in toluene (5 mL) was placed in a 50 mL autoclave with glass inlet and teflon stir bar was pressurized with H_2 (50 bar). The reaction mixture was warmed to 100 °C (heating block) and was stirred for 3 h. The reaction mixture was cooled to ambient temperature and the H_2 pressure was released. The resulting yellow solution is a mixture of **6EMind** (76%), ***syn*-7EMind** (12%) and ***anti*-7EMind** (12%, determined by $^{31}P\{^1H\}$ NMR spectroscopy). The ratio of the diadducts (**7EMind**) could not be increased by extending the reaction time to 48 h, so the reaction is already in equilibrium after 3 h.

$^{31}P\{^1H\}$ NMR (C_6D_6, 202.5 MHz)[2]: δ = 57.5 (s, 4 P, *P*H (***anti*-7EMind**)), 58.1 (s, 2 P, *P*H (**6EMind**)), 58.7 (s, 4 P, *P*H (***syn*-7EMind**)), 286.5 (s, 2 P, *P*·(**6EMind**). **^{31}P NMR** (C_6D_6, 101.3 MHz): *see chapter 5.2, p. S72ff.*

[2] Due to very different relaxation times in **6EMind**, the spectrum was recorded with a longer D1 time of 30 s.

Figure S25: $^{31}P\{^1H\}$ spectrum of the reaction mixture.

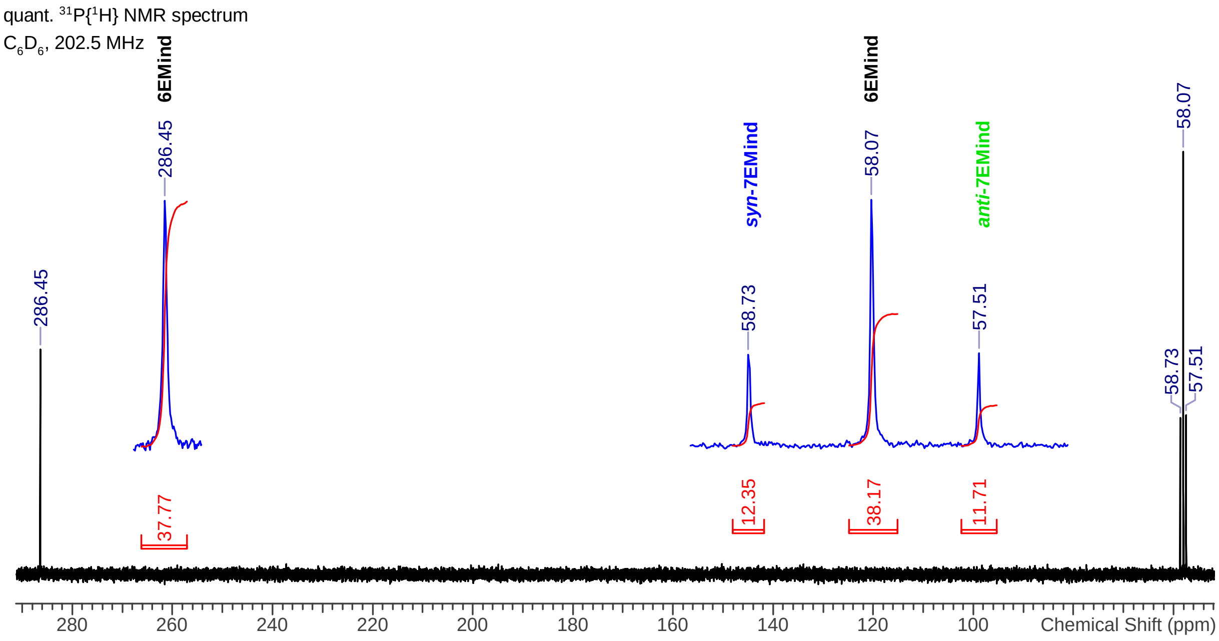

5 Additional spectroscopic details

5.1 ^{31}P NMR data of 6EMind

To describe the spin patterns in the ^{1}H and ^{31}P NMR spectra of **6EMind**, the spectra were fitted in *gNMR*[6] (full lineshape iteration procedure) using calculated NMR data (GIAO method, PBE-D3/def2-TZVP) as initial values. For this purpose, **6EMind** was approximated as an AA'BB'XX'YY' spin system ($P_aP_a'P_bP_b'H_aH_a'H_bH_b'$):

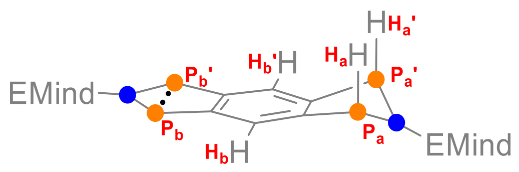

The computed values as well as the final iterated values are listed in Table S3. The simulated spectra are shown in Figure S26 and Figure S27.

Table S3. Experimental NMR data of **6EMind**, calculated values (PBE-D3/def2-TZVP, averaged according to C_s symmetry in solution) in brackets.

X	***δ* [ppm]**				***J* [Hz]**			
		***P*_a-X**	***P*_a′-X**	***P*_b-X**	***P*_b′-X**	***H*_a-X**	***H*_a′-X**	***H*_b-X**
P_a	58.1 (96.8)	-	-	-	-	-	-	-
P_a'	58.1 (96.8)	−26 (−26)	-	-	-	-	-	-
P_b	286.5 (340.7)	3 (2)	−1 (−2)	-	-	-	-	-
P_b'	286.5 (340.7)	−1 (−1)	3 (2)	10 (21)	-	-	-	-
H_a	6.40 (7.1)	182.7 (113.8)	2.4 (2.3)	2.1 (3.0)	−1.0 (−1.4)	-	-	-
H_a'	6.40 (7.1)	2.4 (2.3)	182.7 (113.8)	−1.0 (−1.4)	2.1 (3.0)	4.5 (5.2)	-	-
H_b	8.31 (8.9)	7.3 (5.4)	0.0 (−0.2)	1.1 (4.0)	1.2 (−2.1)	−2.1 (−1.3)	0.4 (0.2)	-
H_b'	8.31 (8.9)	0.0 (−0.2)	7.3 (5.4)	1.2 (−2.1)	1.1 (4.0)	0.4 (0.2)	−2.1 (−1.3)	1.8 (0.8)

Figure S26: Experimental (400.1 MHz) and simulated ^{31}P NMR spectrum in toluene-d_8 of **6EMind**.

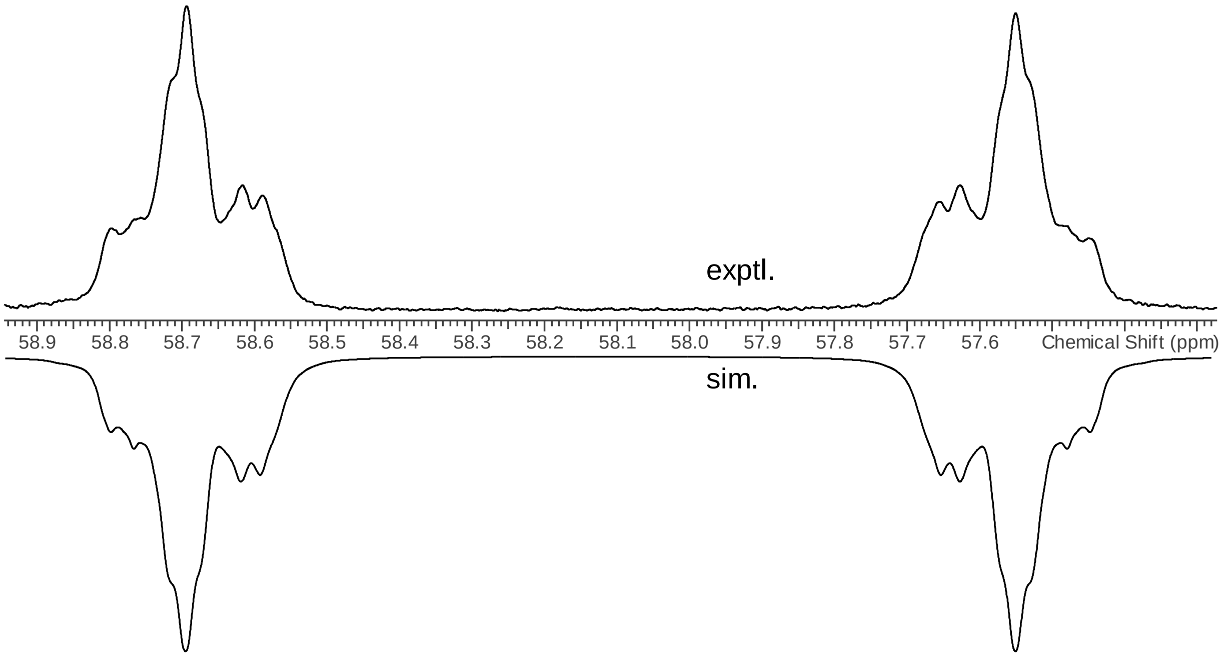

Figure S27: Experimental (162.0 MHz) and simulated ^{1}H NMR spectrum in toluene-d_8 of **6EMind**.

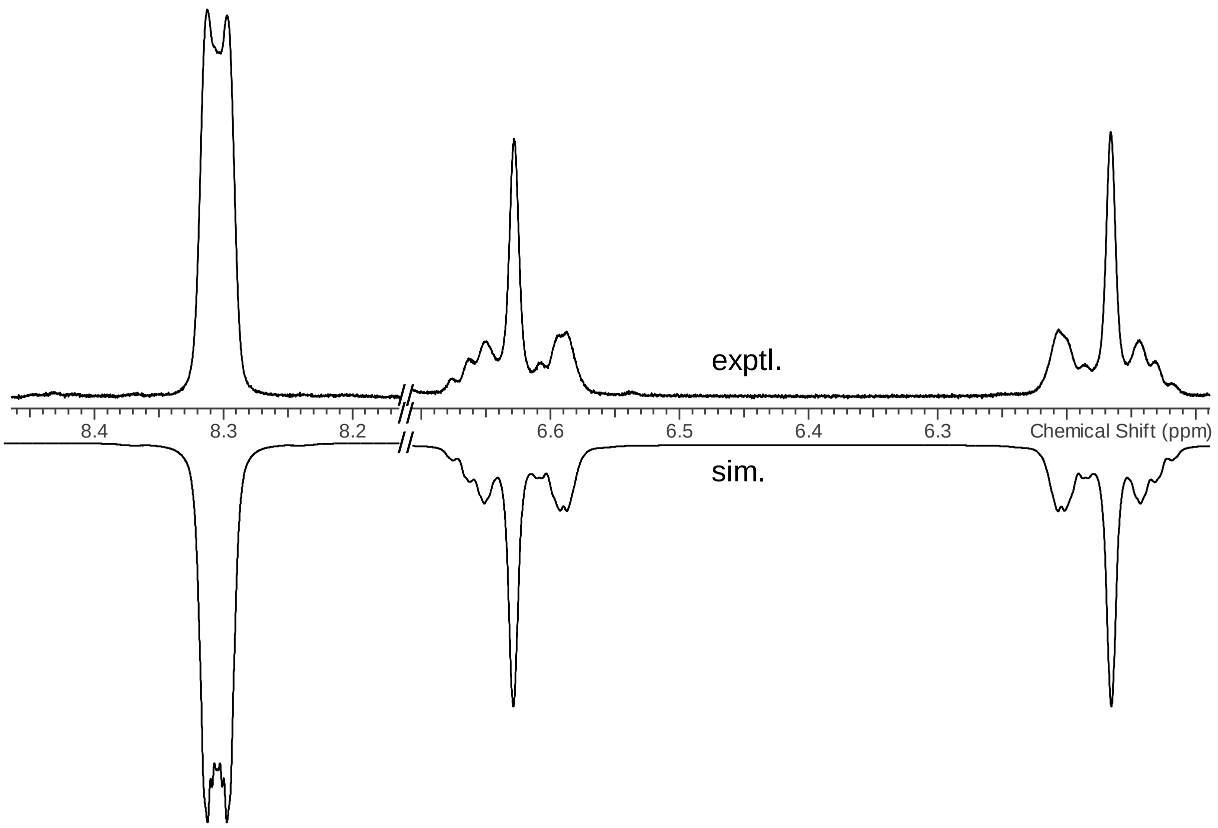

It is conceivable that the slight asymmetry in the signal intensity is due to hindered inversion at the bent cyclopentyl rings. The point group in solution is thus reduced from C_s (as in Table S3) to C_1. The spin system changes from AA'BB'XX'YY' ($P_aP_a'P_bP_b'H_aH_a'H_bH_b'$) to ABCDWXYZ ($P_aP_bP_cP_dH_aH_bH_cH_d$):

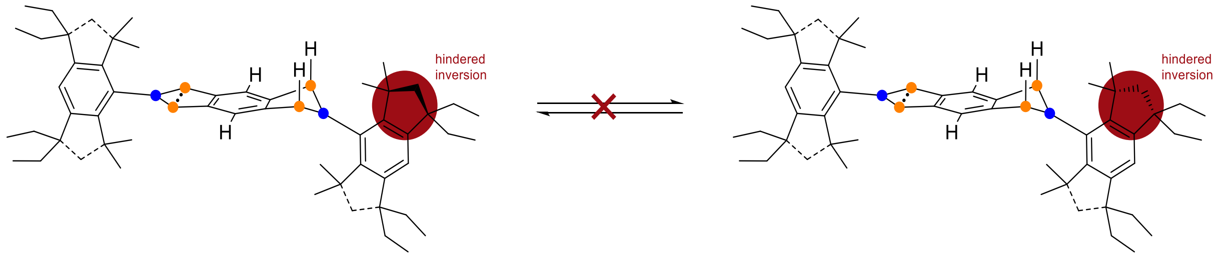

Apart from the asymmetric intensity in the signals, the approximation in C_s symmetry is sufficient to describe the spin patterns of **6EMind**.

5.2 ^{31}P NMR data of 7EMind

Additionally, calculations (GIAO method, PBE0-D3/def2-TZVP) were carried out to describe the spin patterns of ***syn*-7EMind** and ***anti*-7EMind**. An assignment in the ^{31}P NMR spectrum was made on the basis of the calculated shifts. Small coupling constants (<10 Hz) are to be considered approximate and cannot be fully resolved due to the complex spin system.

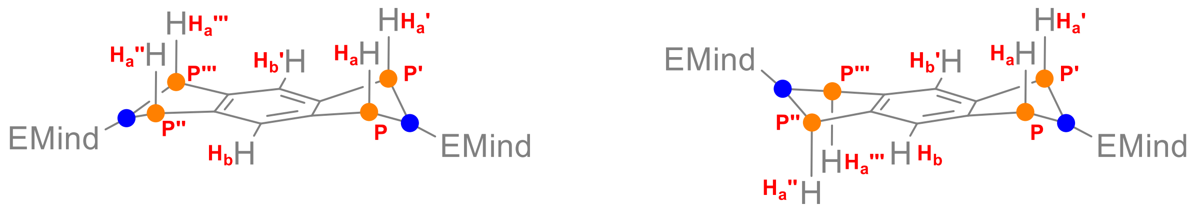

***syn*-7EMind** ***anti*-7EMind**

Table S4. Experimental NMR data of ***syn*-7EMind**, calculated values (PBE-D3/def2-TZVP, averaged according to C_{2v} symmetry in solution) in brackets.

X	***δ* [ppm]**	***J* [Hz]**								
		P-X	***P′-X***	***P′′-X***	***P′′′-X***	***H_a-X***	***H_a′-X***	***H_a′′-X***	***H_a′′′-X***	***H_b-X***
P	58.7 (69.8)	-	-	-	-	-	-	-	-	-
P′	58.7 (69.8)	−27 (−29)	-	-	-	-	-	-	-	-
P′′	58.7 (69.8)	0 (2)	2 (−1)	-	-	-	-	-	-	-
P′′′	58.7 (69.8)	1 (−1)	0 (2)	−27 (−29)	-	-	-	-	-	-
H_a	(6.9)	184 (142)	3 (2)	5 (0)	−4 (0)	-	-	-	-	-
H_a′	(6.9)	3 (2)	184 (142)	−4 (0)	5 (0)	6 (7)	-	-	-	-
H_a′′	(6.9)	5 (0)	−4 (0)	184 (142)	3 (2)	21 (−1)	21 (3)	-	-	-
H_a′′′	(6.9)	−4 (0)	5 (0)	3 (2)	184 (142)	21 (3)	21 (−1)	6 (7)	-	-
H_b	(8.5)	7 (5)	1 (0)	6 (5)	−1 (0)	−1 (−1)	1 (1)	−1 (−1)	1 (1)	-
H_b′	(8.5)	1 (0)	7 (5)	−1 (0)	6 (5)	1 (1)	−1 (−1)	1 (1)	−1 (−1)	3 (1)

Table S5. Experimental NMR data of ***anti*-7EMind**, calculated values (PBE-D3/def2-TZVP, averaged according to C_{2h} symmetry in solution) in brackets.

X	***δ* [ppm]**	***J* [Hz]**								
		P-X	***P′-X***	***P″-X***	***P‴-X***	***H_a-X***	***H_a′-X***	***H_a″-X***	***H_a‴-X***	***H_b-X***
P	57.5 (69.4)	-	-	-	-	-	-	-	-	-
P′	57.5 (69.4)	−26 (−28)	-	-	-	-	-	-	-	-
P″	57.5 (69.4)	2 (1)	1 (2)	-	-	-	-	-	-	-
P‴	57.5 (69.4)	1 (2)	2 (1)	−26 (−28)	-	-	-	-	-	-
H_a	(6.9)	183 (139)	4 (2)	2 (0)	−2 (0)	-	-	-	-	-
H_a′	(6.9)	4 (2)	183 (139)	−2 (0)	2 (0)	8 (8)	-	-	-	-
H_a″	(6.9)	2 (0)	−2 (0)	183 (139)	4 (2)	27 (−1)	27 (2)	-	-	-
H_a‴	(6.9)	−2 (0)	2 (0)	4 (2)	183 (139)	27 (2)	27 (−1)	8 (8)	-	-
H_b	(8.4)	6 (5)	1 (0)	5 (5)	0 (0)	−1 (1)	1 (1)	−1 (−1)	1 (1)	-
H_b″	(8.4)	1 (0)	6 (5)	0 (0)	5 (5)	1 (1)	−1 (−1)	1 (1)	−1 (−1)	−2 (1)

Figure S28: Experimental (162.0 MHz) and simulated ^{31}P NMR spectrum from a mixture of **6EMind**, ***syn*-7EMind** and ***anti*-7EMind**.

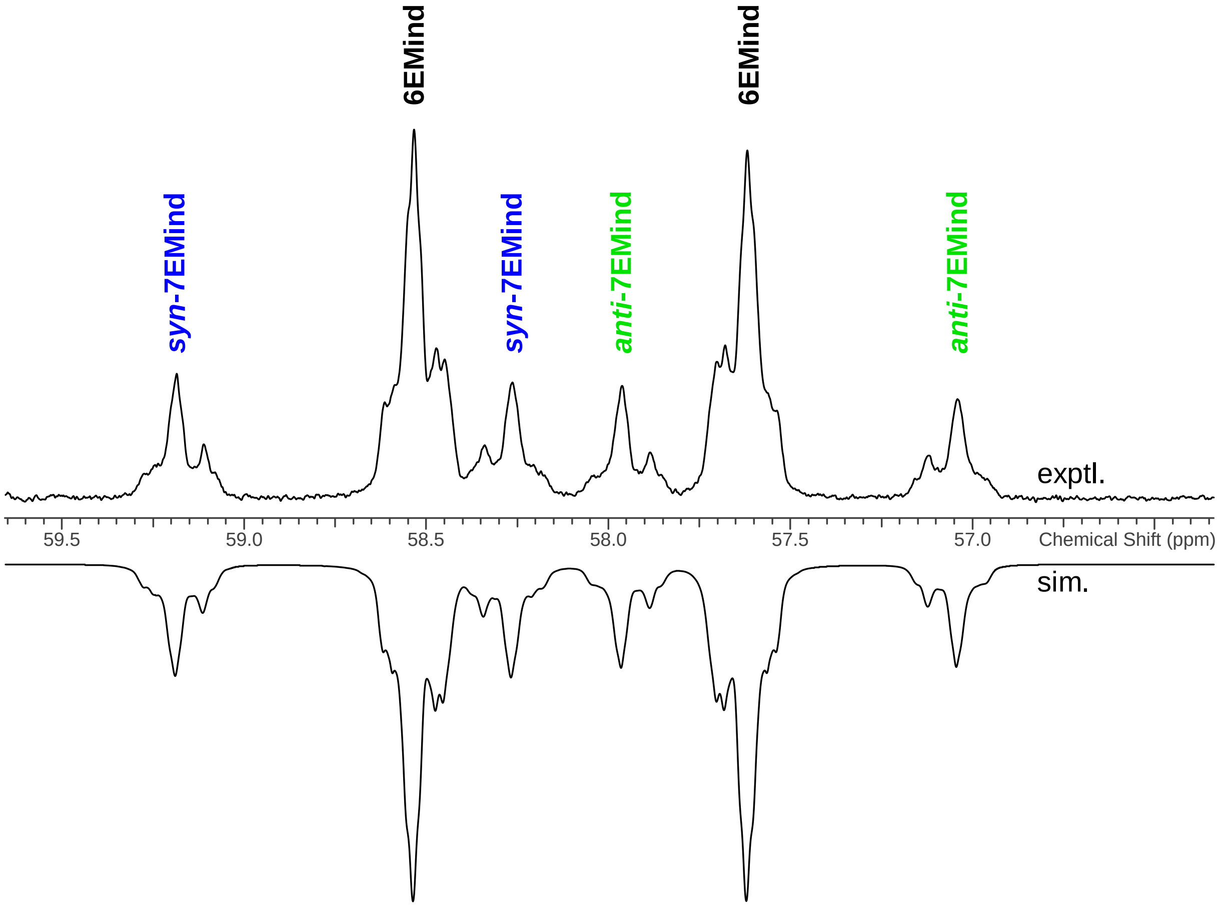

6 Parahydrogen experiments

6.1 General information

NMR experiments with parahydrogen were performed using a 400 MHz Bruker AV 400 NMR spectrometer equipped with a broad-band 5 mm radiofrequency probe and the standard temperature control unit. Parahydrogen-enriched H_2 gas (92%) referred to as simply para-H_2 in the main text was produced using a Bruker parahydrogen generator.

6.2 Reaction of 1EMind with para-H_2

For the reaction of **1EMind** with para-H_2, the NMR sample was prepared by addition of **1EMind** (30 mg, 0.034 mmol) into dry degassed toluene-d_8 (0.6 mL) in a 5 mm Wilmad gas-tight NMR tube (medium wall). Heating to ca. 60°C was required to dissolve this amount of **1EMind** in toluene-d_8. Thereafter, the sample tube was charged with 5 bar of para-H_2, avoiding gas-liquid mixing, and the tube was vigorously shaken for ca. 5 s before insertion to the NMR magnet. The reaction at room temperature was slow which resulted in no hyperpolarization and no rapid ortho-para conversion. In contrast, heating to 67 °C led to the strongly enhanced signals of **6EMind** (Figure 14a in the main text). A single tube shaking allowed measuring several ^{31}P NMR spectra before para-H_2 converted into normal H_2 and the hyperpolarization completely decayed or before the completion of the reaction. The lifetime of **1EMind** and para-H_2 was on the order of 10 min at this temperature.

6.3 Reaction of 6EMind with para-H_2

For this experiment, **6EMind** biradical was preliminary synthesized *in situ* by keeping a solution of **1EMind** (30 mg, 0.034 mmol) in toluene-d_8 (0.6 ml) in a 5 mm Wilmad gas-tight NMR tube (medium wall) at 97 °C under 5 bar of normal H_2 for 1 h. According to ^{31}P NMR, this procedure resulted in the quantitative conversion of **1EMind** to **6EMind**. Thereafter, the sample tube was depressurized, the normal H_2 atmosphere was replaced with 5 bar of para-H_2. The sample was thoroughly shaken just before NMR measurements to introduce para-H_2 into the **6EMind** solution, and the temperature was elevated to 97 °C. ^{31}P NMR spectra revealed enhanced signals corresponding to hyperpolarized ***syn*-7EMind**, ***anti*-7EMind** and **6EMind**, as shown in Figure 14b of the main text. The reaction was not fast, allowing many repeated measurements during approximately 30 min time window without the need for additional supply of fresh para-H_2.

7 Computational details

7.1 General remarks

Computations were carried out using Gaussian09[17], ORCA 4.2.1[18] or ORCA 5.0.3[19] and the standalone version of NBO 6.0.[20–23]

Structure optimizations employed either the hybrid DFT functional PBE0[24–26] or the pure DFT exchange-correlation functional PBE[24,25] in conjunction with Grimme's dispersion correction D3(BJ)[27,28] and the def2 basis set family[29] (notation e.g. PBE0-D3/def2-TZVP). The resolution of identity (RI) approximation was employed, using the appropriate Coulomb fitting basis of the Weigend group.[30]

Since polyradicals require multi-determinantal wave functions, they cannot be accurately described by typical single-reference methods such as HF, DFT or coupled cluster theory. A qualitatively correct wave function can be obtained by multi-configurational SCF (MCSCF) methods which describe non-dynamic correlation, e.g. Complete Active Space SCF (CASSCF);[31–39] better descriptions including dynamic correlation require expensive multi-reference perturbation (MRPT) or multi-reference configuration-interaction (MRCI) calculations, which limit the size of the systems that can be computed. Nonetheless, previous investigations have shown that (especially nonhybrid) DFT methods can give reasonable results if the multi-configurational character is not too large.[40,41] Structure optimizations were therefore carried out using the pure DFT exchange-correlation functional PBE[24,25]. The stability of all Kohn-Sham wavefunctions was checked to verify the validity of the single-determinantal, restricted Kohn-Sham DFT approach. In accordance with CASSCF computations, which indicate that the multi reference character is not too large (vide infra), the restricted KS wavefunctions (using pure functionals) were stable in all instances with respect to symmetry breaking. The conditions of all optimizations are listed in Table S6 (p. S80f.)

All structures were fully optimized and confirmed as minima by frequency analyses. This does not include the calculations for the dimers **4R** (R = Ter, EMind, Mes*, Oma) at PBE-D3/def2-TZVP level of theory as frequency analyses are computationally too expensive here. However, the optimisations of **4R** were based on pre-optimised structures at PBE-D3/def2-SVP that were verified as minima. In order to be able to specify free Gibbs energies for the dimerization of **1R** to **4R** at triple zeta level, the thermal corrections to the Gibbs energy from the corresponding PBE-D3/def2-SVP calculation were added to the electronic energies received from the PBE-D3/def2-TZVP optimisations.

Chemical shifts and coupling constants were derived by the GIAO method.[42–46] The calculated absolute shifts ($\sigma_{calc,X}$) were referenced to the experimental absolute shift of 85 % H_3PO_4 in the gas phase ($\sigma_{ref,1}$ = 328.35 ppm),[47] using PH_3 ($\sigma_{ref,2}$ = 594.45 ppm) as a secondary standard:[48]

$$\begin{aligned}\delta_{calc,X} &= (\sigma_{ref,1} - \sigma_{ref,2}) - (\sigma_{calc,X} - \sigma_{calc,PH_3}) \\ &= \sigma_{calc,PH_3} - \sigma_{calc,X} - 266.1\ \text{ppm}\end{aligned}$$

At the PBE-D3/def2-TZVP level of theory, σ_{calc,PH_3} amounts to +563.03 ppm, at the PBE0-D3/def2-TZVP level of theory to +572.09 ppm.

SCF energies in solution were gained by single point calculations on the optimized structures using the SMD continuum solvation model.[49]

More accurate electronic energies for optimized structures were computed by single-point DLPNO-CCSD(T) [50–52] calculations employing the def2-TZVP basis set[29,30] and def2-TZVP/C correlation fitting basis[53] (notation: DLPNO-CCSD(T)/def2-TZVP). Thermodynamic quantities at this level of theory were calculated using the DLPNO-CCSD(T) single-point energy and the thermal corrections at the PBE-D3/def2-TZVP level of theory. The T1 diagnostic was evaluated in each case to ensure reliable results (empirically, CCSD(T) results are considered reliable if T1 < 0.02).[40]

Please note that most of the computations were carried out for single, isolated molecules in the gas phase (ideal gas approximation). There may well be significant differences between gas phase and condensed phase.

7.2 Summary of calculated data

Table S6. Summary of calculated data, including electronic energies and thermal corrections (‡ describes the transition state of the hydrogenation that leads to the indicated compound).

Compd.	PG	Opt. method	E_{tot}[a]	ΔG[b]	$E_{CCSD(T)}$[c]	T_1
H_2	$D_{\infty h}$	PBE-D3/def2-TZVP	−1.1661	−0.0017	−1.1684	0.0061
PH_3	C_{3v}	PBE-D3/def2-TZVP	−342.9845	0.0032	-	-
PH_3	C_{3v}	PBE0-D3/def2-TZVP	−343.0171	0.0040	-	-
**1H**	D_{2h}	PBE-D3/def2-TZVP	−1705.1755	0.0585	−1703.5818	0.0127
**6H**‡	C_s	PBE-D3/def2-TZVP	−1706.3300	0.0706	−1704.7305	0.0135
**6H**	C_s	PBE-D3/def2-TZVP	−1706.3663	0.0734	−1704.7836	0.0128
***syn*-7H**‡	C_S	PBE-D3/def2-TZVP	−1707.5153	0.0865	−1705.9240	0.0131
***syn*-7H**	C_{2v}	PBE-D3/def2-TZVP	−1707.5479	0.0899	−1705.9718	0.0124
***anti*-7H**‡	C_s	PBE-D3/def2-TZVP	−1707.5156	0.0865	−1705.9243	0.0131
***anti*-7H**	C_{2h}	PBE-D3/def2-TZVP	−1707.5482	0.0899	−1705.9719	0.0124
**6EMind**‡	C_1	PBE-D3/def2-TZVP	−3577.0963	1.0658	−3573.6375	0.0098
**6EMind**	C_1	PBE-D3/def2-TZVP	−3577.1350	1.0685	−3573.6929	0.0097
***syn*-7EMind**‡	C_1	PBE-D3/def2-TZVP	−3578.2812	1.0821	−3574.8286	0.0098
***syn*-7EMind**	C_s	PBE-D3/def2-TZVP	−3578.3158	1.0860	−3574.8783	0.0097
***anti*-7EMind**‡	C_1	PBE-D3/def2-TZVP	−3578.2804	1.0822	−3574.8281	0.0098
***anti*-7EMind**	C_i	PBE-D3/def2-TZVP	−3578.3144	1.0853	−3574.8772	0.0097
**1Ter**	D_2	PBE-D3/def2-SVP	−3558.8315	0.7783	-	-
**1Ter**	D_2	PBE-D3/def2-TZVP	−3561.5969 (−3561.6352)[d] (−3561.6374)[e]	0.7757	-	-
**1EMind**	D_2	PBE-D3/def2-SVP	−3573.1582	1.0523	−3572.4937	0.0095
**1EMind**	D_2	PBE-D3/def2-TZVP	−3575.9454 (−3575.9729)[d] (−3575.9762)[e]	1.0507	-	-
**1Mes***	C_2	PBE-D3/def2-SVP	−3107.0873	0.8178	-	-
**1Mes***	C_2	PBE-D3/def2-TZVP	−3109.3977	0.8168	-	-

Compd.	PG	Opt. method	E_{tot}[a]	ΔG[b]	$E_{CCSD(T)}$[c]	T_1
			(−3109.4222)[d]<br>(−3109.4245)[e]			
**1Oma**	D_2	PBE-D3/def2-SVP	−3416.2025	0.9585	-	-
**1Oma**	D_2	PBE-D3/def2-TZVP	−3418.8216<br>(−3418.8496)[d]<br>(−3418.8521)[e]	0.9589	-	-
**4Ter**	C_2	PBE-D3/def2-SVP	−7117.7836	1.6143	-	-
**4Ter**	C_2	PBE-D3/def2-TZVP	−7123.2933<br>(−7123.3564)[d]<br>(−7123.3580)[e]	-	-	-
**4EMind**	C_2	PBE-D3/def2-SVP	−7146.3524	2.1342	-	-
**4EMind**	C_2	PBE-D3/def2-TZVP	−7151.9195<br>(−7151.9719)[d]<br>(−7151.9770)[e]	-	-	-
**4Mes***	C_2	PBE-D3/def2-SVP	−6214.1985	1.6679	-	-
**4Mes***	C_2	PBE-D3/def2-TZVP	−6218.8107<br>(−6218.8580)[d]<br>(−6218.8609)[e]	-	-	-
**4Oma**	C_2	PBE-D3/def2-SVP	−6832.4164	1.9438	-	-
**4Oma**	C_2	PBE-D3/def2-TZVP	−6837.6467<br>(−6837.7017)[d]<br>(−6837.7048)[e]	-	-	-

[a] Total SCF energy in a.u.; [b] thermal correction to Gibbs energy in a.u. (298 K unless stated otherwise); [c] single-point DLPNO-CCSD(T)/def2-TZVP energy; [d] single-point Energy in THF (SMD model)[49]; [e] single-point Energy in toluene (SMD model)[49].

7.3 Steric demand (cone angle, buried volume) in 1R

The cone angles in **1R** were calculated between the N atom of the central ring system and the substituent R, connected to this according to the previously described method.[54] The cone angles are listed in Table S7 and were determined from the structure optimized at PBE-D3/def2-TZVP level of theory.

Table S7. Cone angles and buried volumes[55] of the substituents in **1Dmp**, **1Ter** and **1^{tBu}Bhp**.

**R**	Ter	EMind	Mes*	Oma
cone angle [°]	222	246	265	265
V_{bur} (r = 2.0 Å) [%]	15.9	17.2	23.7	24.6
V_{bur} (r = 2.5 Å) [%]	19.6	23.4	28.2	29.7
V_{bur} (r = 3.0 Å) [%]	24.2	27.5	30.9	32.6
V_{bur} (r = 3.5 Å) [%]	28.1	29.1	31.6	33.1
V_{bur} (r = 4.0 Å) [%]	30.7	28.8	30.7	31.8
V_{bur} (r = 4.5 Å) [%]	32.1	27.6	28.5	29.5
V_{bur} (r = 5.0 Å) [%]	32.3	26.3	26.1	26.9
V_{bur} (r = 5.5 Å) [%]	31.0	24.7	23.6	24.5
V_{bur} (r = 6.0 Å) [%]	28.2	22.9	20.9	22.2

The buried volumes caused by the different substituents in **1Ter**, **1EMind**, **1Mes*** and **1Oma** were calculated with the SambVca 2.1 application.[55] The structures used for the determination of the buried volume were optimized at the PBE-D3/def2-TZVP level of theory. For the calculations, the center of the sphere was placed at the midpoint between the two P atoms in one five membered ring. The value for the mesh spacing in the numerical integration was set to 0.05. The H atoms were included and the bond radii were not scaled. The $C_6H_2P_4N_2$ fragment in **1Ter**, **1EMind**, **1Mes*** and **1Oma** was removed for the calculations and therefore does not contribute to the buried volume

In the following the contour plots of the different substituents in **1Ter**, **1EMind**, **1Mes*** and **1Oma** are illustrated for different sphere radii.

Figure S29: Orientation of the sterically demanding substituents in the contour plots in Table S8.

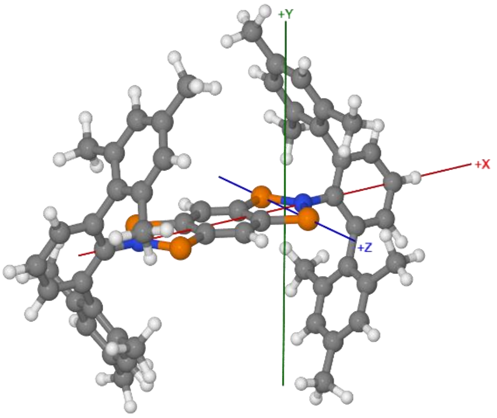

1Ter

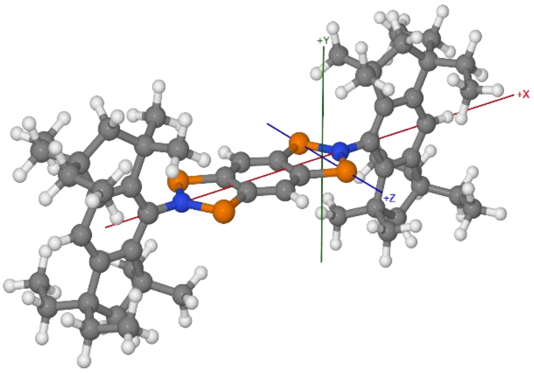

1EMind

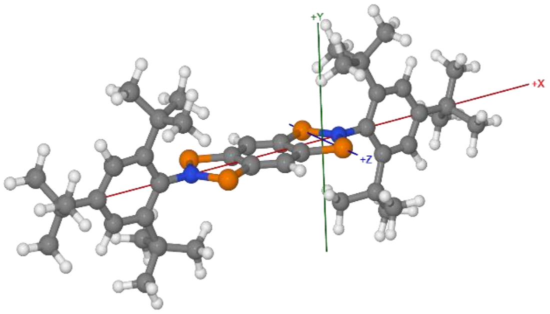

1Mes*

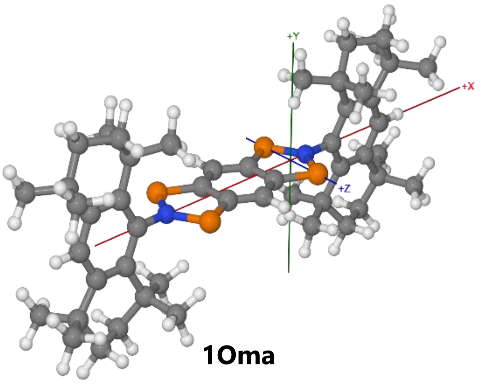

1Oma

Table S8. Contour plots of the sterically demanding substituents in **1Ter**, **1EMind**, **1Mes*** and for sphere radii of 3.0 Å, 4.5 Å and 6.0 Å.

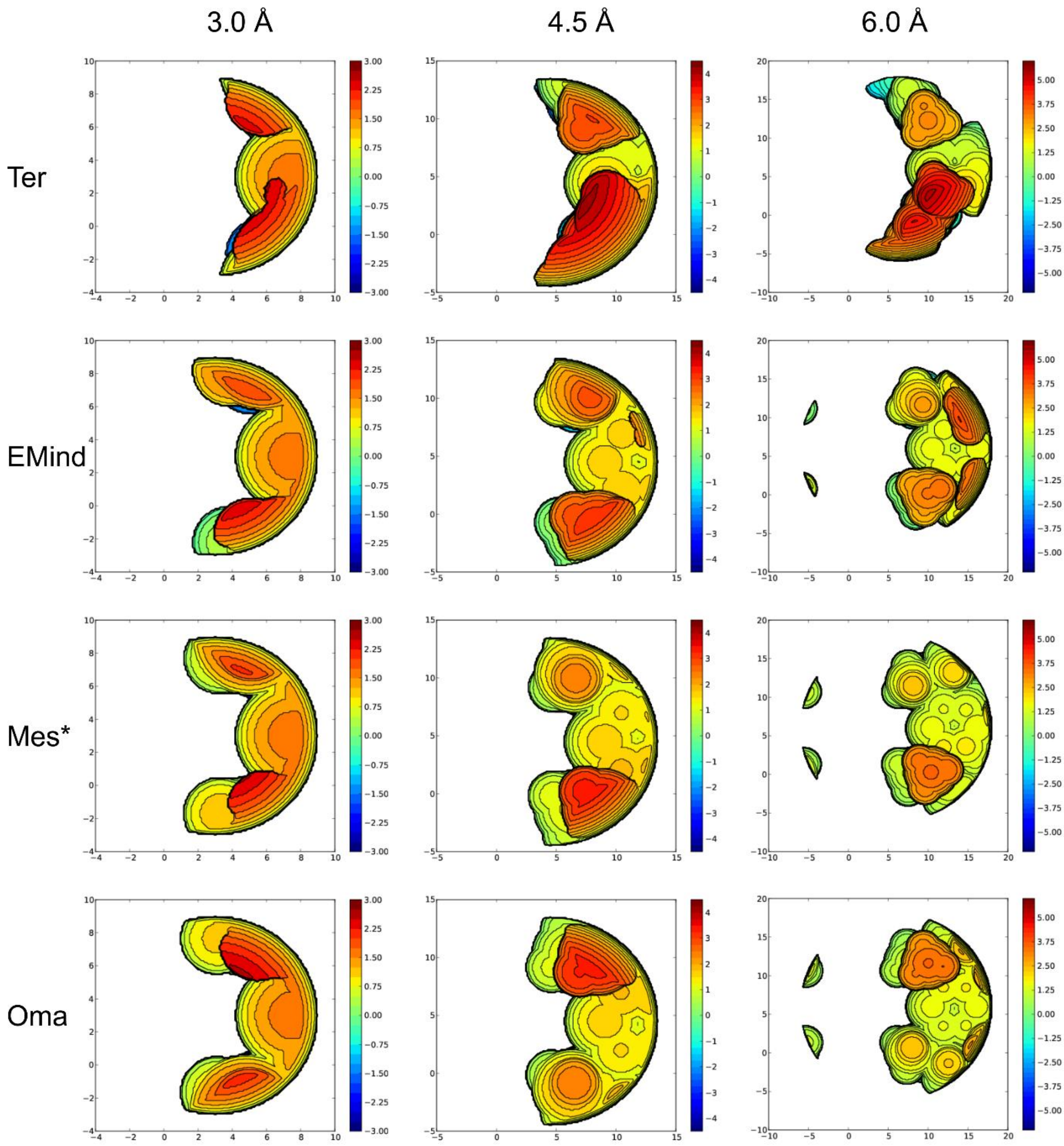

7.4 NBOs and NLMOs of 1H

Figure S30: Selected NBOs and related NLMOs of **1H** (PBE-D3/def2-TZVP). The orbital occupation numbers of the NBO calculation are given.

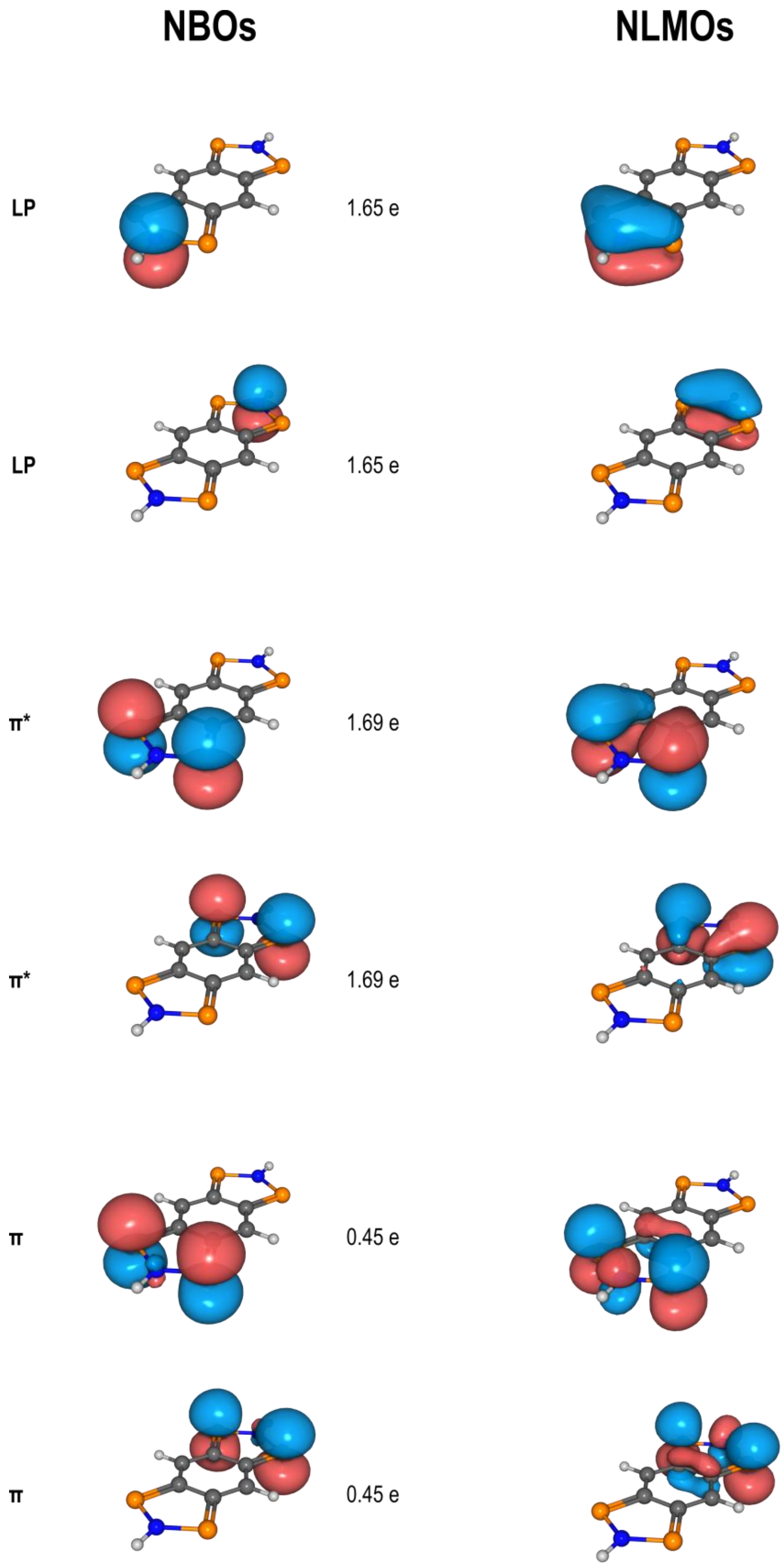

7.5 CASSCF computations of 1H, 1EMind and 6H

In the following, the orbitals of the active space of a CASSCF(4,4) and a CASSCF(14,12) computation of **1H**, the orbitals of a CASSCF(14,12) computation of **1EMind** and the orbitals of the active space of a CASSCF(2,2) computation of **6H** are illustrated and their occupation numbers are given.

Figure S31: CAS(4,4)/def2-TZVP orbitals of **1H**. The orbital occupation numbers of the CAS calculation are given.

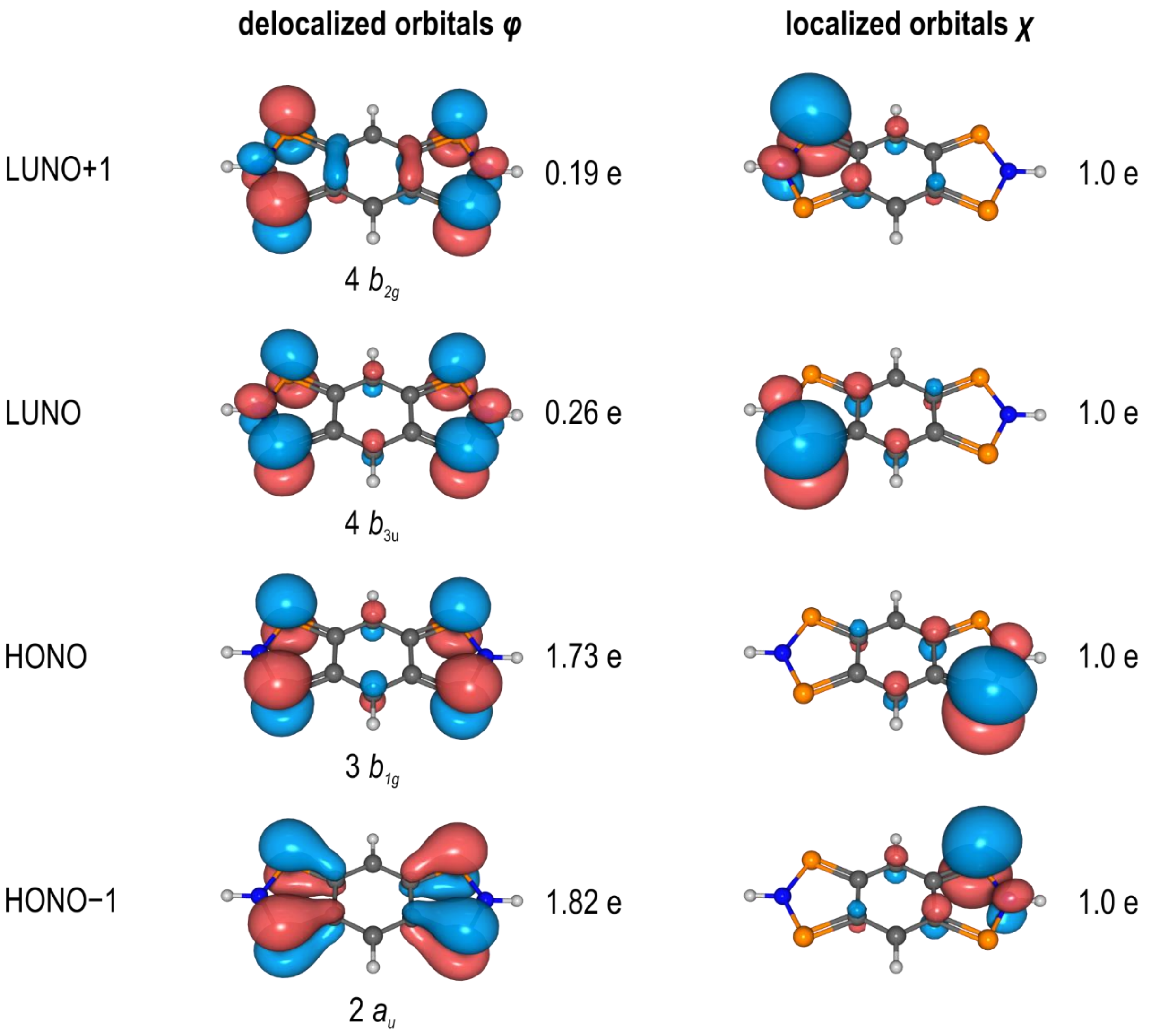

Figure S32: CAS(14,12)/def2-TZVP orbitals of **1H**. The orbital occupation numbers of the CAS calculation are given.

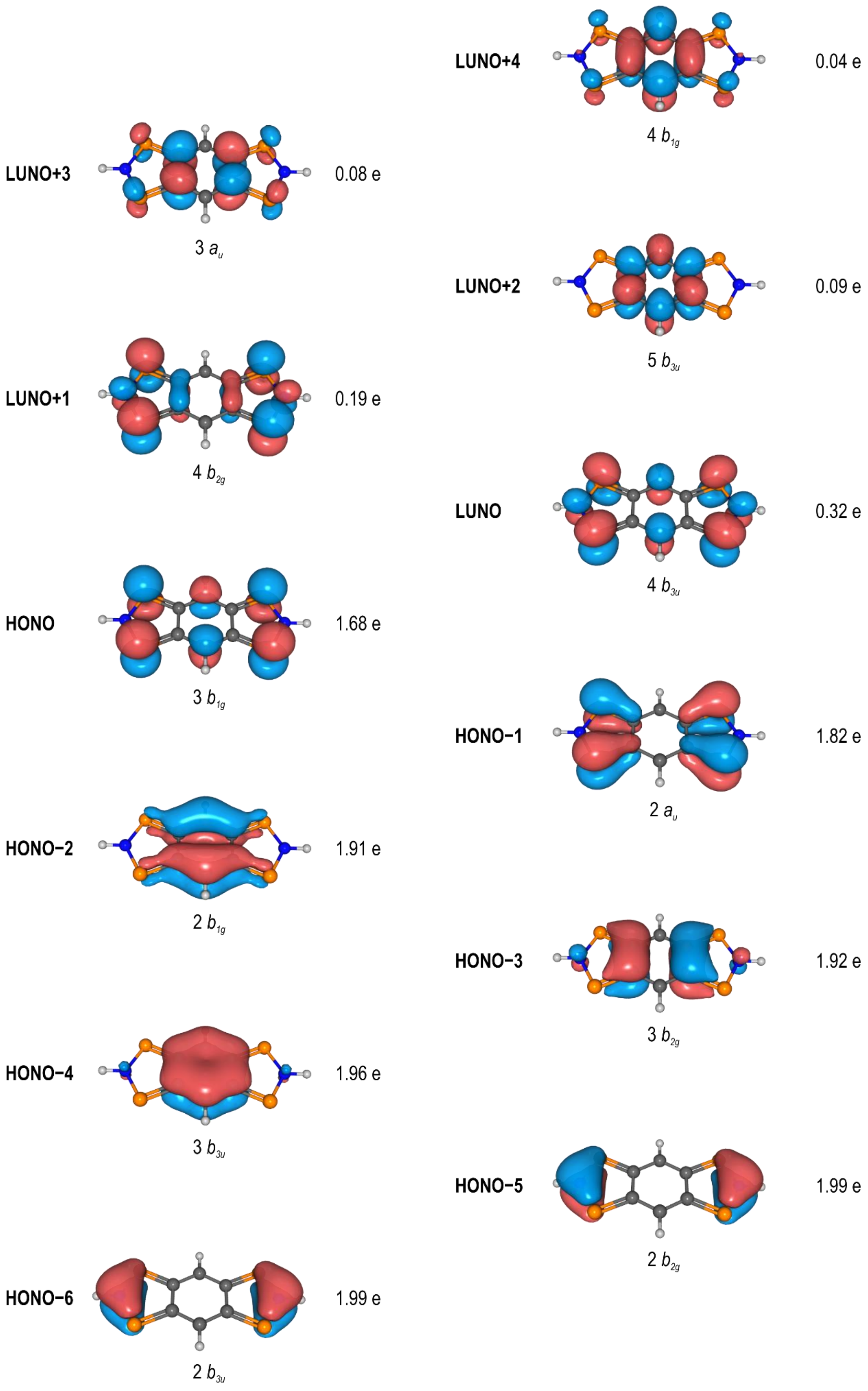

Figure S33: CAS(14,12)/def2-TZVP orbitals of **1EMind**. The orbital occupation numbers of the CAS calculation are given.

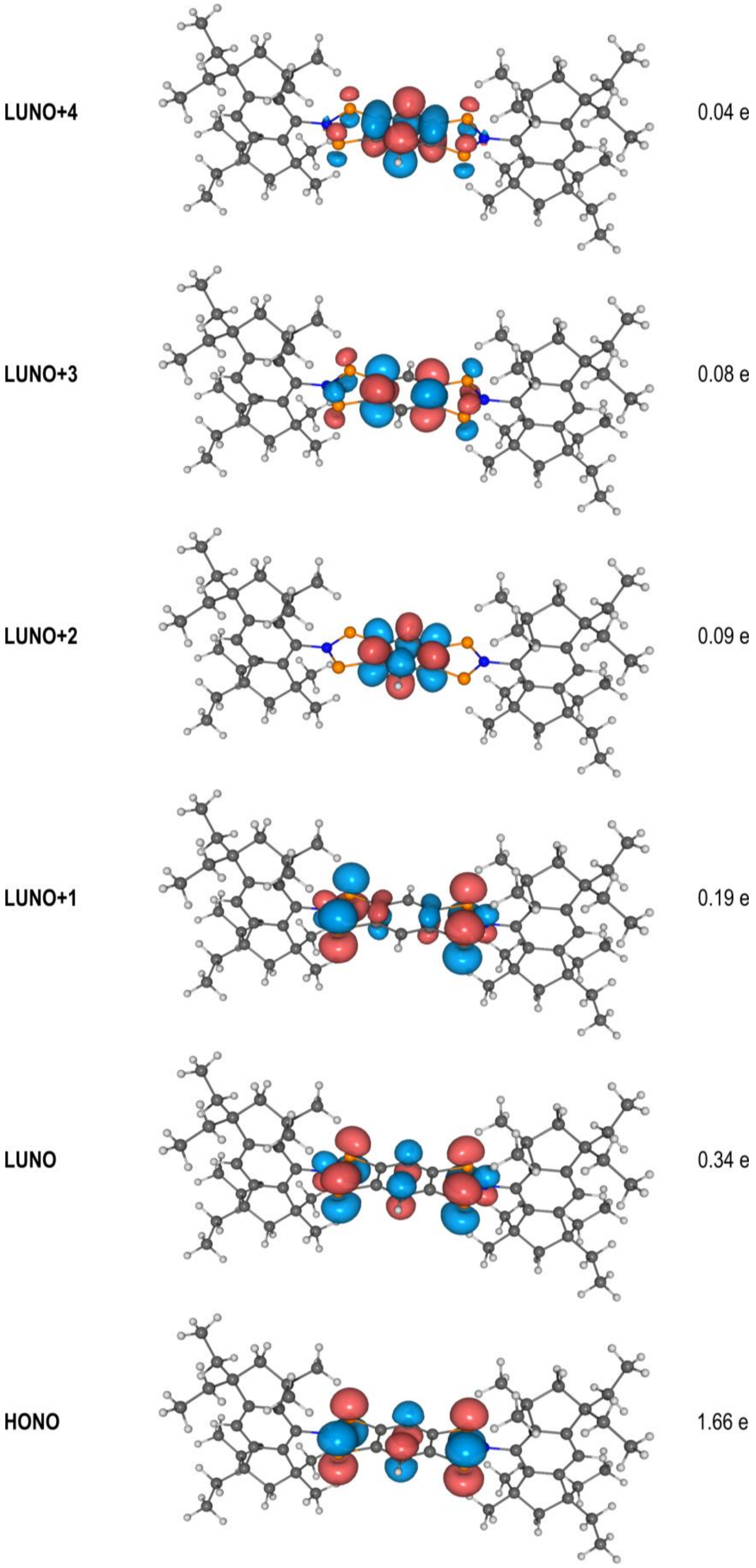

HONO−1 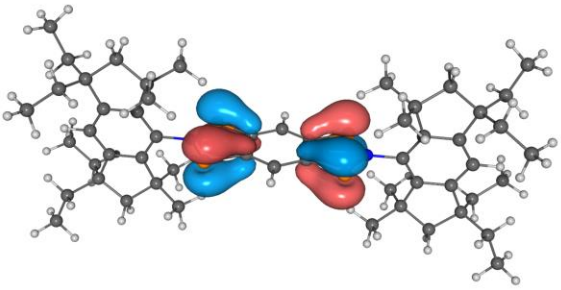 1.82 e

HONO−2 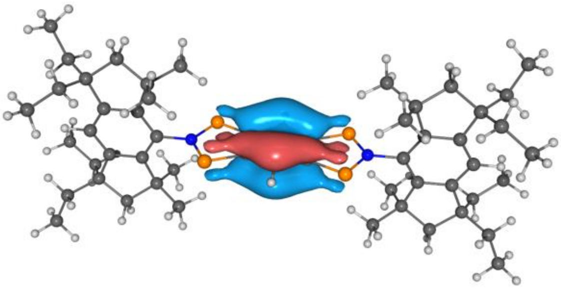 1.91 e

HONO−3 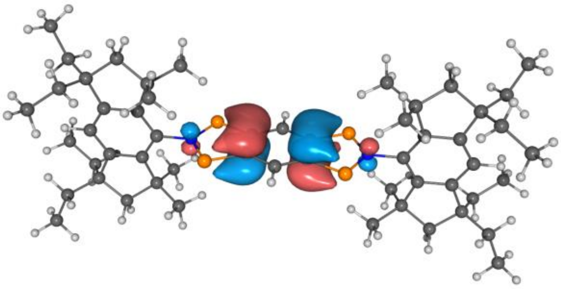 1.92 e

HONO−4 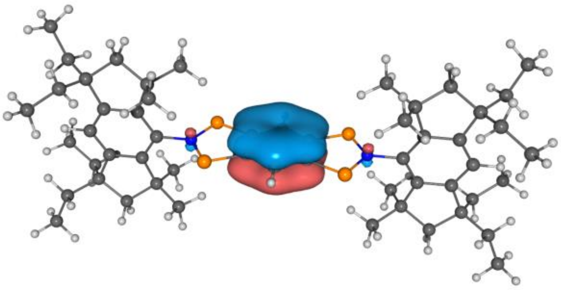 1.96 e

HONO−5 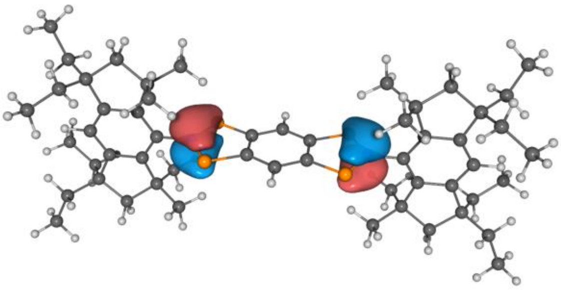 1.99 e

HONO−6 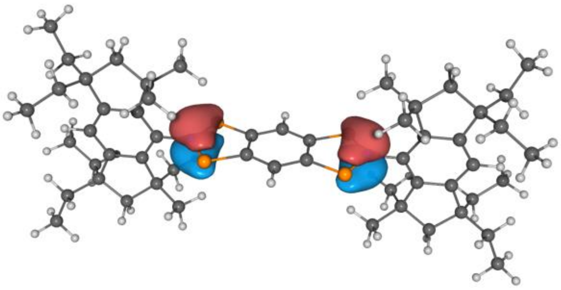 1.99 e

Figure S34: CAS(2,2)/def2-TZVP orbitals of **6H**. The orbital occupation numbers of the CAS calculation are given.

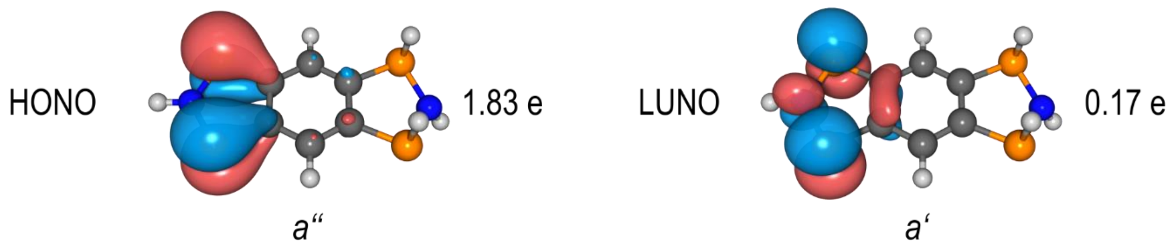

7.6 Exited states and electron exchange coupling constants

CAS(4,4), CAS(14,12), SC-NEVPT2/CAS(14,12), DLPNO-CCST(T) and DFT calculations were performed to describe different spin and excited states of **1H** and **1EMind**. Table S9 lists the first 10 exited states of **1H**. As expected, the energetic order of these states depends on the size of the active space and the inclusion of dynamic electron correlation. SC-NEVPT2/CAS(14,12) and DLPNO-CCSD(T) approaches give reasonably similar results, so these were used for the discussion in the manuscript.

It is worthy to note that no significant difference could be found between the proton substituted model system **1H** and the synthesized **1EMind** (Table S10), in which the phenyl rings of the substituents are orthogonal to the central ring system, so there is no extended conjugation that may alter the electronic structure in comparison to the model system.

Table S9. Excited states of **1H** calculated with different methods (the first ten exited states are listed).

	CAS(4, 4)/def2-TZVP			CAS(14, 12)/def2-TZVP			SC-NEVPT2/CAS(14,12)/def2-TZVP			DLPNO-CCSD(T)		
excited state	term symbol	ΔE [eV]	ΔE [kJ/mol]	term symbol	ΔE [eV]	ΔE [kJ/mol]	term symbol	ΔE [eV]	ΔE [kJ/mol]	term symbol	ΔE [eV]	ΔE [kJ/mol]
0	^1A_g	0.00	0	^1A_g	0.00	0	^1A_g	0.00	0	^1A_g	0.00	0
1	$^3B_{2u}$	0.77	74	$^3B_{2u}$	0.73	71	$^3B_{2u}$	0.97	93	$^3B_{2u}$	1.01	97
2	$^3B_{3g}$	1.52	146	$^3B_{3g}$	1.33	129	$^3B_{3g}$	1.59	154			
3	^1A_g	2.43	235	^1A_g	2.22	214	$^1B_{2u}$	1.95	188			
4	$^3B_{1u}$	2.50	242	$^3B_{1u}$	2.25	217	^1A_g	2.66	256			
5	^5A_g	2.70	260	^5A_g	2.52	243	$^3B_{1u}$	2.84	274			
6	$^1B_{2u}$	4.01	387	$^3B_{2u}$	3.10	299	$^3B_{3g}$	3.00	290			
7	$^1B_{3g}$	4.96	478	$^1B_{3g}$	3.14	303	$^1B_{3g}$	3.11	300			
8	$^3B_{3g}$	5.18	500	$^1B_{1u}$	3.26	315	$^3B_{2u}$	3.11	300			
9	$^1B_{3g}$	5.32	513	$^1B_{2u}$	3.59	346	$^1B_{3g}$	3.16	305			
10	$^3B_{1u}$	5.46	527	$^3B_{2u}$	3.86	372	^5A_g	3.22	311	^5A_g	3.03	292

Table S10. Excited states of **1EMind** calculated with different methods (the first ten exited states are listed).

	CAS(14, 12)/def2-TZVP			SC-NEVPT2/CAS(14,12)/def2-TZVP			PBE-D3-def2-TZVP		
excited state	term symbol	Δ*E* [eV]	Δ*E* [kJ/mol]	term symbol	Δ*E* [eV]	Δ*E* [kJ/mol]	term symbol	Δ*E* [eV]	Δ*E* [kJ/mol]
0	^1A_g	0.00	0	^1A_g	0.00	0	^1A_g	0.00	0
1	^3B_u	0.67	64	^3B_u	0.95	91	$^3B_{2u}$	0.78	75
2	^3B_g	1.33	128	^1B_u	1.40	135			
3	^1A_g	2.16	208	^3B_g	1.61	156			
4	^3A_u	2.19	211	^1A_g	2.63	253			
5	^5A_g	2.49	240	^3A_u	2.86	276			
6	^3B_u	3.07	296	^5A_g	3.24	313	^5A_g	3.07	296
7	^1B_g	3.08	297						
8	^1A_u	3.21	310						
9	^1B_u	3.52	339						
10	^3B_u	3.84	371						

Based on the energies of the excited states, the interaction between the radical electrons can be quantified in terms of electron exchange coupling constants (σ, μ, λ) according to the phenomenological Heisenberg-Dirac-van Vleck Hamiltonian.[56,57] Figure S35 shows two tetraradicals in D_{2h} symmetry, a system consisting of four hydrogen atoms and the model system **1H**. The energetic order of the first five excited states determines the sign of σ, μ and λ. Thus, σ and μ are negative and λ positive for the system of four hydrogen atoms. In **1H**, σ and λ are negative, but μ is positive.

Figure S35: Energetic order of the exited states in a system of four hydrogen atoms in D_{2h} symmetry and in **1H**.

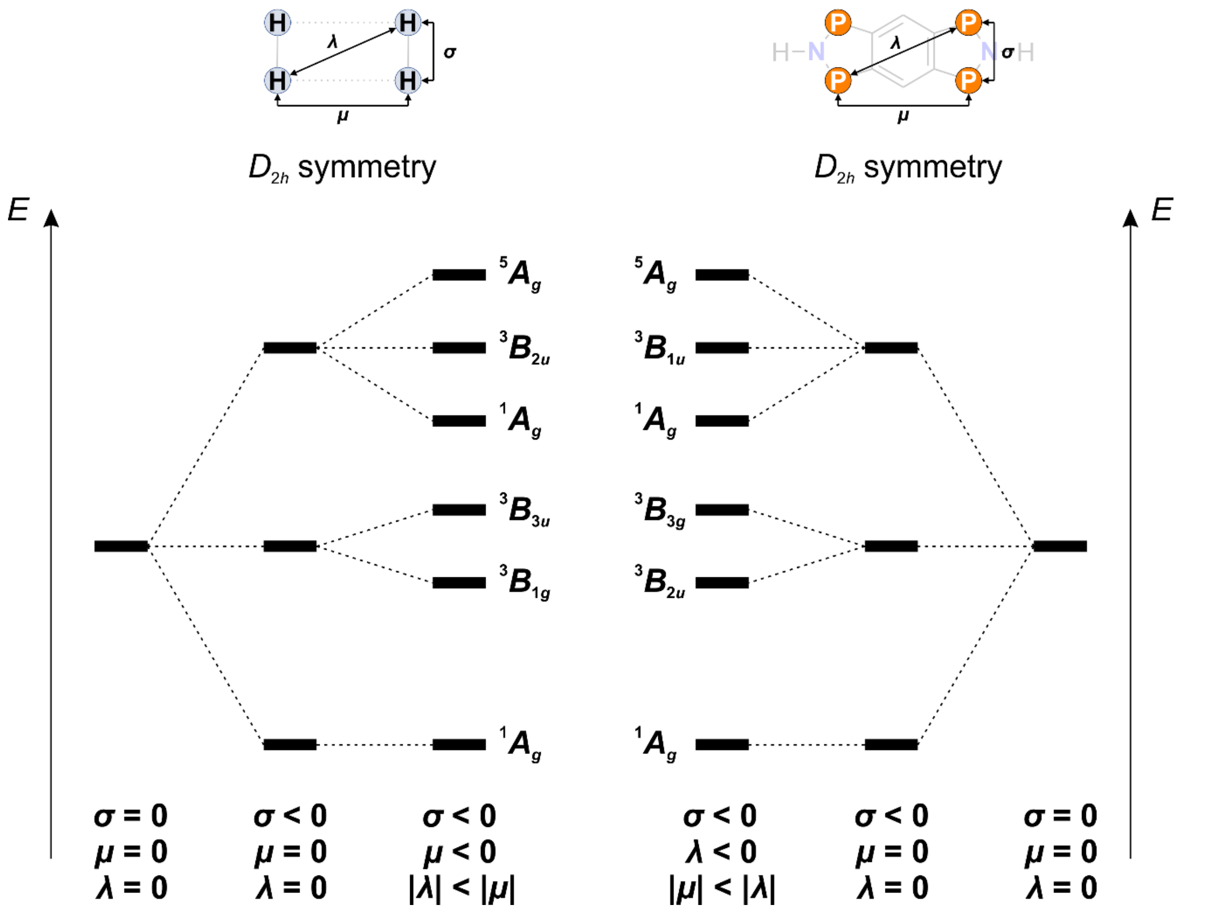

The relationship between the ground and exited state energies as well as the coupling constants can be inferred from the phenomenological Heisenberg-Dirac-van Vleck Hamiltonian ($\hat{S}$: spin operator; J: electron exchange coupling constant):

$$\hat{H}_{\mathrm{HDvV}} = -\sum_{i<j} J_{ij}\,\hat{S}_i\hat{S}_j$$

$$= -J_{1,2}\,\hat{S}_1\hat{S}_2 - J_{1,3}\hat{S}_1\hat{S}_3 - J_{1,3}\hat{S}_1\hat{S}_4 - J_{2,3}\hat{S}_2\hat{S}_3 - J_{2,4}\hat{S}_2\hat{S}_4 - J_{3,4}\hat{S}_3\hat{S}_4$$

When the coupling constants are designated as in Figure S35 ($J_{1,2} = J_{3,4} = \sigma$; $J_{1,3} = J_{2,4} = \mu$; $J_{1,4} = J_{2,3} = \lambda$), the expression can be written as:

$$\hat{H}_{\mathrm{HDvV}} = -\sigma\left(\hat{S}_1\hat{S}_2 + \hat{S}_3\hat{S}_4\right) - \mu\left(\hat{S}_1\hat{S}_3 + \hat{S}_2\hat{S}_4\right) - \lambda(\hat{S}_1\hat{S}_4 + \hat{S}_2\hat{S}_3)$$

In order to obtain the relationship between the coupling constants and the energy of a specific state, the Heisenberg-Dirac-van Vleck Hamiltonian must be applied to the wave function (ψ) of that state. This will be exemplified by the ^5A_g state, since only one determinant must be considered here (and all electrons have the same spin):

$$\langle\psi|-\ \sum_{i<j}(J_{i,j}\hat{S}_i\hat{S}_j)\ |\psi\rangle = \langle 1\ 2\ 3\ 4|-\ \sum_{i<j}(J_{i,j}\hat{S}_i\hat{S}_j)\ |1\ 2\ 3\ 4\ \rangle$$

Using the Condon Slater rules,[58] we can show that

$$\langle 1\ 2\ 3\ 4|-\sum_{i<j}\left(J_{i,j}\hat{S}_i\hat{S}_j\right)|1\ 2\ 3\ 4\ \rangle =$$

$$\begin{aligned}
&-\sigma\left[\langle 1\ 2|\hat{S}_1\hat{S}_2|1\ 2\rangle - \langle 1\ 2|\hat{S}_1\hat{S}_2|2\ 1\rangle\right]\\
&-\mu\left[\langle 1\ 3|\hat{S}_1\hat{S}_2|1\ 3\rangle - \langle 1\ 3|\hat{S}_1\hat{S}_2|3\ 1\rangle\right]\\
&-\lambda\left[\langle 1\ 4|\hat{S}_1\hat{S}_2|1\ 4\rangle - \langle 1\ 4|\hat{S}_1\hat{S}_2|4\ 1\rangle\right]\\
&-\lambda\left[\langle 2\ 3|\hat{S}_1\hat{S}_2|2\ 3\rangle - \langle 2\ 3|\hat{S}_1\hat{S}_2|3\ 2\rangle\right]\\
&-\mu\left[\langle 2\ 4|\hat{S}_1\hat{S}_2|2\ 4\rangle - \langle 2\ 4|\hat{S}_1\hat{S}_2|4\ 2\rangle\right]\\
&-\sigma\left[\langle 3\ 4|\hat{S}_1\hat{S}_2|3\ 4\rangle - \langle 3\ 4|\hat{S}_1\hat{S}_2|4\ 3\rangle\right]\\
= &-\frac{\sigma}{2}-\frac{\mu}{2}-\frac{\lambda}{2}
\end{aligned}$$

Regardless of the state, there are the following types of 2-electron integrals that lead to the following results:

$$\langle i\ j|\hat{S}_1\hat{S}_2|i\ j\rangle = \langle \bar{i}\ \bar{j}|\hat{S}_1\hat{S}_2|\bar{i}\ \bar{j}\rangle = +\frac{1}{4}$$

$$\langle i\ \bar{j}|\hat{S}_1\hat{S}_2|i\ \bar{j}\rangle = \langle \bar{i}\ j|\hat{S}_1\hat{S}_2|\bar{i}\ j\rangle = -\frac{1}{4}$$

$$\langle i\ \bar{j}|\hat{S}_1\hat{S}_2|\bar{i}\ j\rangle = \langle \bar{i}\ j|\hat{S}_1\hat{S}_2|i\ \bar{j}\rangle = +\frac{1}{2}$$

$$\langle i\ j|\hat{S}_1\hat{S}_2|j\ i\rangle = 0$$

The notation $\bar{i} \rightarrow i\cdot\beta$ and $i \rightarrow i\cdot\alpha$ is used here to distinguish the spins.

For the ^3B_u state, the wave function becomes more complex due to several determinants and the necessity to distinguish between different spins. We use localized orbitals in the following for ease of notation and a more compact representation of the state:

$$\psi = \frac{1}{2}\left[-|\overline{1}\ 2\ 3\ 4\rangle - |1\ \overline{2}\ 3\ 4\rangle - |1\ 2\ \overline{3}\ 4\rangle - |1\ 2\ 3\ \overline{4}\rangle\right]$$

$$\begin{aligned}\langle\psi|\widehat{H}_{\mathrm{HDvV}}|\psi\rangle = \frac{1}{4}\Big[&\langle\overline{1}\ 2\ 3\ 4|\widehat{H}_{\mathrm{HDvV}}|\overline{1}\ 2\ 3\ 4\rangle + \langle\overline{1}\ 2\ 3\ 4|\widehat{H}_{\mathrm{HDvV}}|1\ \overline{2}\ 3\ 4\rangle\\ &- \langle\overline{1}\ 2\ 3\ 4|\widehat{H}_{\mathrm{HDvV}}|1\ 2\ \overline{3}\ 4\rangle - \langle\overline{1}\ 2\ 3\ 4|\widehat{H}_{\mathrm{HDvV}}|1\ 2\ 3\ \overline{4}\rangle\\ &+ \langle 1\ \overline{2}\ 3\ 4|\widehat{H}_{\mathrm{HDvV}}|\overline{1}\ 2\ 3\ 4\rangle + \langle 1\ \overline{2}\ 3\ 4|\widehat{H}_{\mathrm{HDvV}}|1\ \overline{2}\ 3\ 4\rangle\\ &- \langle 1\ \overline{2}\ 3\ 4|\widehat{H}_{\mathrm{HDvV}}|1\ 2\ \overline{3}\ 4\rangle - \langle 1\ \overline{2}\ 3\ 4|\widehat{H}_{\mathrm{HDvV}}|1\ 2\ 3\ \overline{4}\rangle\\ &- \langle 1\ 2\ \overline{3}\ 4|\widehat{H}_{\mathrm{HDvV}}|\overline{1}\ 2\ 3\ 4\rangle - \langle 1\ 2\ \overline{3}\ 4|\widehat{H}_{\mathrm{HDvV}}|1\ \overline{2}\ 3\ 4\rangle\\ &+ \langle 1\ 2\ \overline{3}\ 4|\widehat{H}_{\mathrm{HDvV}}|1\ 2\ \overline{3}\ 4\rangle + \langle 1\ 2\ \overline{3}\ 4|\widehat{H}_{\mathrm{HDvV}}|1\ 2\ 3\ \overline{4}\rangle\\ &- \langle 1\ 2\ 3\ \overline{4}|\widehat{H}_{\mathrm{HDvV}}|\overline{1}\ 2\ 3\ 4\rangle - \langle 1\ 2\ 3\ \overline{4}|\widehat{H}_{\mathrm{HDvV}}|1\ \overline{2}\ 3\ 4\rangle\\ &+ \langle 1\ 2\ 3\ \overline{4}|\widehat{H}_{\mathrm{HDvV}}|1\ 2\ \overline{3}\ 4\rangle + \langle 1\ 2\ 3\ \overline{4}|\widehat{H}_{\mathrm{HDvV}}|1\ 2\ 3\ \overline{4}\rangle\Big]\end{aligned}$$

The equation can be transformed into two electron integrals using the Condon-Slater rules and simplified into the following expression:

$$\langle\psi|\widehat{H}_{\mathrm{HDvV}}|\psi\rangle = -\frac{1}{2}\sigma + \frac{1}{2}\mu + \frac{1}{2}\lambda$$

Analogously, the relative energies of the remaining states can be expressed in terms of σ, μ and λ as listed in Table S11.

Table S11. Energetic dependency of the electron exchange coupling constants σ, μ and λ.

State	Irrep.	$E_{rel.}$ =
**Q1**	A_g	$-\frac{\sigma}{2}-\frac{\mu}{2}-\frac{\lambda}{2}$
**T3**	B_{1u}	$-\frac{\sigma}{2}+\frac{\mu}{2}+\frac{\lambda}{2}$
**S1**	A_g	$-\frac{\sigma}{2}+\mu+\lambda$
**T2**	B_{3g}	$+\frac{\sigma}{2}+\frac{\mu}{2}-\frac{\lambda}{2}$
**T1**	B_{2u}	$+\frac{\sigma}{2}-\frac{\mu}{2}+\frac{\lambda}{2}$
**S0**	A_g	$+\frac{3}{2}\sigma$

Using a least square procedure, we can now fit the computed energies of the excited states to extract the coupling parameters σ, μ and λ (Table S12).

Table S12. Coupling constants σ, μ and λ calculated with different methods.

method	R =	σ in [eV]	σ in [kJ/mol]	μ in [eV]	μ in [kJ/mol]	λ in [eV]	λ in [kJ/mol]
CAS(4, 4)/def2-TZVP	H	-1.33	-128.1	0.30	29.0	-0.45	-43.5
CAS(14, 12)/def2-TZVP	H	-1.23	-118.6	0.21	20.1	-0.39	-37.7
SC-NEVPT2/CAS(14,12)/def2-TZVP	H	-1.55	-149.9	0.14	13.8	-0.48	-46.5
CAS(14, 12)/def2-TZVP	EMind	-1.21	-116.5	0.23	22.1	-0.43	-41.4
SC-NEVPT2/CAS(14,12)/def2-TZVP	EMind	-1.56	-150.8	0.15	14.4	-0.52	-49.9

7.7 Induced ring currents

To estimate the aromaticity of the tetraradical **1EMind**, the magnetically induced ring current density was computed using the gauge-including magnetically induced current (GIMIC) model,[59,60] as implemented in the GIMIC 2.1.4 code,[61,62] which was used in conjunction with the Gaussian09 interface.[63] To exclude any substituent effects on the ring current, a simple model system with R=H (i.e. **1H**) was employed. Nuclear shielding parameters were computed at the PBE-D3/def2-TZVP level of theory. Graphical representations of the current density were generated using ParaView's[64] streamline animation feature. Additionally, NICS(0) NICS(0)$_{zz}$, NICS(1) and NICS(1)$_{zz}$ values[65–67] were computed (PBE/def2-TZVP), which are often used as indicators for aromaticity. The calculated NICS values nicely agree with the results obtained using the GIMIC method.

For comparison, we here list previously reported values for benzene and **EH**, as reference molecules.[4]

Table S13. Integrated ring current susceptibilities and NICS values. For condensed ring systems, values are given for the five-membered (⬟) and six-membered part (⬢).

compound	ring current susceptibility [nA/T][a]			NICS(0)[a]	NICS(0)$_{zz}$	NICS(1)[a]	NICS(1)$_{zz}$
	diatropic	paratropic	net induced				
**benzene**	17.1	−5.0	**12.1**	−8.2	−16.1	−10.1	**−30.2**
**EH**	16.1 (⬟)<br>16.3 (⬢)	−2.6 (⬟)<br>−5.1 (⬢)	**13.5** (⬟)<br>**11.2** (⬢)	−11.7 (⬟)<br>−7.0 (⬢)	−15.3 (⬟)<br>−9.5 (⬢)	−11.2 (⬟)<br>−8.9 (⬢)	**−31.1** (⬟)<br>**−24.9** (⬢)
**1H**	16.0 (⬟)<br>17.8 (⬢)	−2.8 (⬟)<br>−4.2 (⬢)	**13.2** (⬟)<br>**13.5** (⬢)	−11.0 (⬟)<br>−9.6 (⬢)	−12.9 (⬟)<br>−14.2 (⬢)	−10.6 (⬟)<br>−10.7 (⬢)	**−29.5** (⬟)<br>**−29.4** (⬢)

[a]: values for benzene and **EH** were taken from reference[4]

Figure S36: Streamline representations of the current densities of benzene (A), naphthalene (B), indole (C), borazine (D), [P(μ-NH)]$_2$ (E) as model system for the biradical [P(μ-NTer)]$_2$, **AH** (F) and **EH** (G) and **1H** (H). All illustrations expect the one for **1H** (H) were taken from our recent publication.[4] NICS values and integrated ring current susceptibilities can be found in the reference as well.

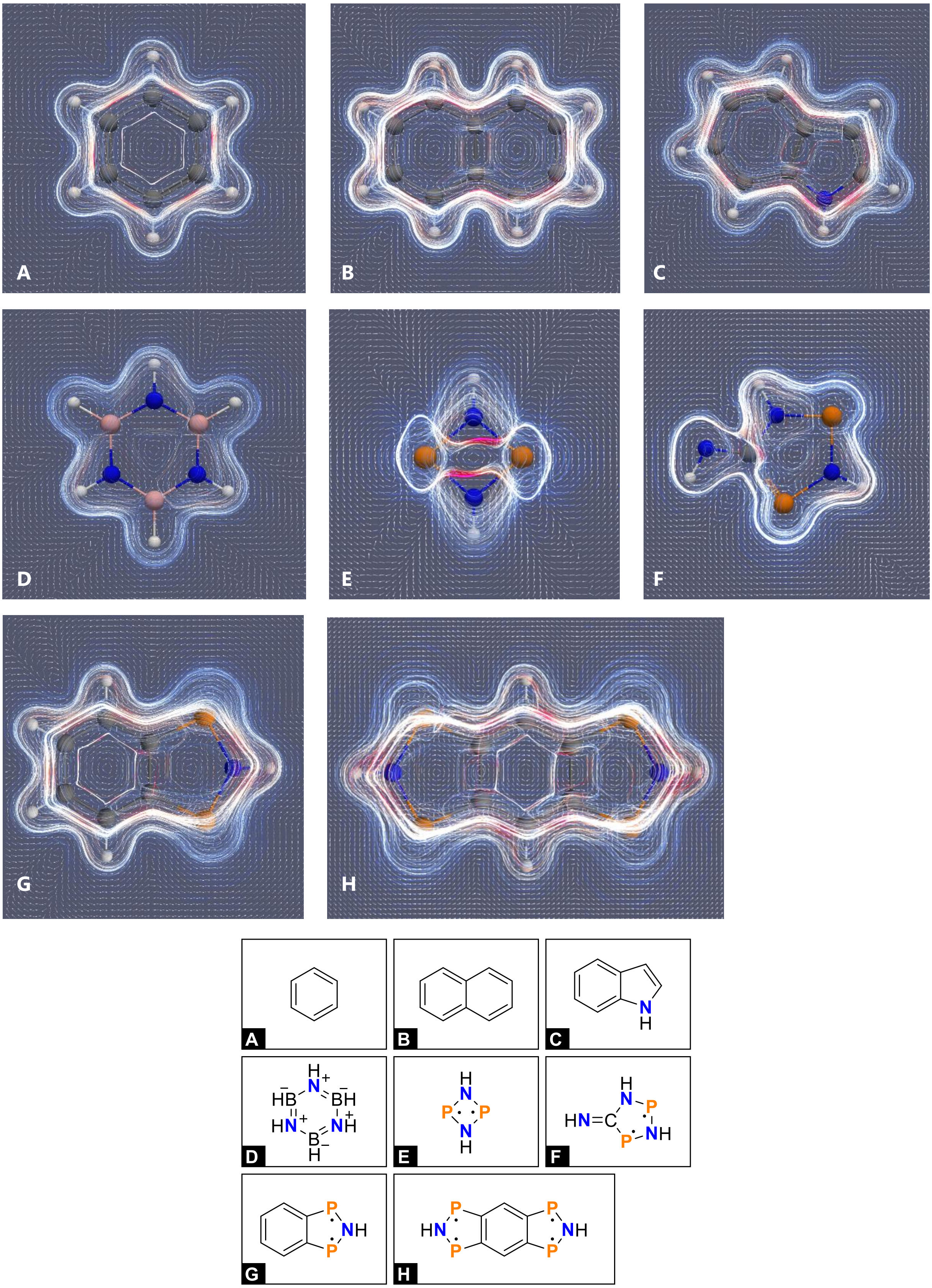

7.8 Optimized structures (.xyz-files)

7.8.1 H_2

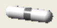

```
2
H2 @ PBE-D3/def2-TZVP
H          0.00000         0.00000         0.37573
H          0.00000         0.00000        -0.37573
```

7.8.2 PH_3

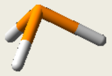

```
4
PH3 @ PBE-D3/def2-TZVP
P         -0.00000         0.00000         0.13202
H         -0.00000         1.19102        -0.66012
H         -1.03145        -0.59551        -0.66012
H          1.03145        -0.59551        -0.66012
```

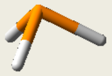

```
4
PH3 @ PBE0-D3/PBE0-D3/def2-TZVP
P         -0.00000         0.00000         0.12903
H          0.00000         1.18980        -0.64513
H         -1.03039        -0.59490        -0.64513
H          1.03039        -0.59490        -0.64513
```

7.8.3 1H

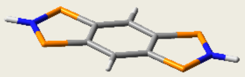

```
16
C6H4N2P4 @ PBE-D3/def2-TZVP
H          0.00000         2.51645         0.00000
C          0.00000         1.42318         0.00000
C          0.00000         0.72723         1.21700
P          0.00000         1.50537         2.80589
C          0.00000        -0.72723         1.21700
P          0.00000        -1.50537         2.80589
C          0.00000        -1.42318         0.00000
H          0.00000        -2.51645         0.00000
C          0.00000        -0.72723        -1.21700
P          0.00000        -1.50537        -2.80589
C          0.00000         0.72723        -1.21700
P          0.00000         1.50537        -2.80589
N          0.00000         0.00000         3.60025
N          0.00000         0.00000        -3.60025
H          0.00000         0.00000         4.61986
H          0.00000         0.00000        -4.61986
```

7.8.4 6H‡

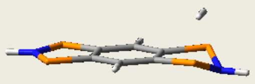

```
18
C6H6N2P4 @ PBE-D3/def2-TZVP
C          0.02069        -1.14214         0.71984
P         -0.05916        -2.79079         1.43986
C          0.02069        -1.14214        -0.71984
N          0.50897        -3.52486        -0.00000
P         -0.05916        -2.79079        -1.43986
H         -1.63029        -2.82662         0.47782
H         -1.63029        -2.82662        -0.47782
C          0.01119         0.05309         1.42406
C          0.00453         1.28125         0.72418
H          0.01814         0.05024         2.51700
P          0.01095         2.86180         1.50189
C          0.00453         1.28125        -0.72418
N          0.01067         3.66392         0.00000
C          0.01119         0.05309        -1.42406
P          0.01095         2.86180        -1.50189
H          0.01814         0.05024        -2.51700
H          0.00962         4.68327         0.00000
H          0.58684        -4.54072        -0.00000
```

7.8.5 6H

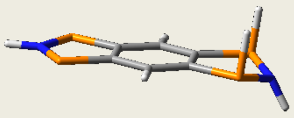

```
18
C6H6N2P4 @ PBE-D3/def2-TZVP
C          0.02832         1.12646         0.71704
P          0.02137         2.80355         1.47996
C          0.02832         1.12646        -0.71704
N         -0.30489         3.64633        -0.00000
P          0.02137         2.80355        -1.47996
H          1.45432         2.94360         1.46434
H          1.45432         2.94360        -1.46434
C          0.00494        -0.06117         1.41739
C          0.00387        -1.30100         0.72122
H         -0.01428        -0.05866         2.51056
P         -0.01533        -2.87457         1.49949
C          0.00387        -1.30100        -0.72122
N         -0.02226        -3.68331         0.00000
C          0.00494        -0.06117        -1.41739
P         -0.01533        -2.87457        -1.49949
H         -0.01428        -0.05866        -2.51056
H         -0.02906        -4.70235         0.00000
H         -1.18783         4.15070        -0.00000
```

7.8.6 *syn*-7H‡

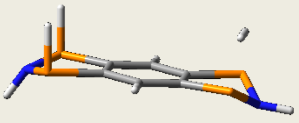

```
20
C6H8N2P4 @ PBE-D3/def2-TZVP
C         -0.02038         1.22340         0.70995
P          0.05180         2.86879         1.43087
C         -0.02038         1.22340        -0.70995
N         -0.54641         3.60240        -0.00000
P          0.05180         2.86879        -1.43087
H          1.58390         2.92814         0.49571
```

```
H           1.58390          2.92814         -0.49571
C          -0.00392          0.00705          1.41555
C           0.01933         -1.19182          0.70981
H          -0.02364          0.00749          2.50819
P           0.00569         -2.86678          1.48119
C           0.01933         -1.19182         -0.70981
N          -0.29448         -3.71459          0.00000
C          -0.00392          0.00705         -1.41555
P           0.00569         -2.86678         -1.48119
H          -0.02364          0.00749         -2.50819
H           1.44057         -2.99142          1.49623
H           1.44057         -2.99142         -1.49623
H          -1.16142         -4.24577          0.00000
H          -0.61898          4.61880         -0.00000
```

7.8.7 *syn*-7H

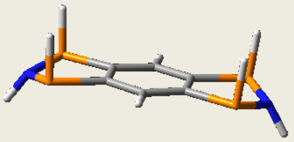

```
20
C6H8N2P4 @ PBE-D3/def2-TZVP
C          -0.70508          1.21003          0.02356
P          -1.48246          2.88097         -0.00104
C           0.70508          1.21003          0.02356
N           0.00000          3.73123         -0.28593
P           1.48246          2.88097         -0.00104
H          -1.52210          3.00440          1.43398
H           1.52210          3.00440          1.43398
C          -1.40684          0.00000          0.00780
C          -0.70508         -1.21003          0.02356
H          -2.49966          0.00000         -0.02220
P          -1.48246         -2.88097         -0.00104
C           0.70508         -1.21003          0.02356
N          -0.00000         -3.73123         -0.28593
C           1.40684         -0.00000          0.00780
P           1.48246         -2.88097         -0.00104
H           2.49966         -0.00000         -0.02220
H          -1.52210         -3.00440          1.43398
H           1.52210         -3.00440          1.43398
H           0.00000         -4.27896         -1.14238
H           0.00000          4.27896         -1.14238
```

7.8.8 *anti*-7H‡

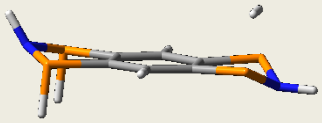

```
20
C6H8N2P4 @ PBE-D3/def2-TZVP
C           0.03408         -1.22352          0.71019
P          -0.08070         -2.86665          1.43067
C           0.03408         -1.22352         -0.71019
N           0.50195         -3.61323         -0.00000
P          -0.08070         -2.86665         -1.43067
H          -1.61281         -2.89139          0.49567
H          -1.61281         -2.89139         -0.49567
C           0.01499         -0.00722          1.41561
C           0.02484          1.19145          0.70991
H           0.00165         -0.00771          2.50860
P           0.02609          2.86571          1.48123
C           0.02484          1.19145         -0.70991
N          -0.26931          3.71501          0.00000
```

```
C           0.01499         -0.00722        -1.41561
P           0.02609          2.86571        -1.48123
H           0.00165         -0.00771        -2.50860
H           1.46157          2.98147         1.49703
H           1.46157          2.98147        -1.49703
H          -1.13192          4.25310         0.00000
H           0.55382         -4.63084        -0.00000
```

7.8.9 *anti*-7H

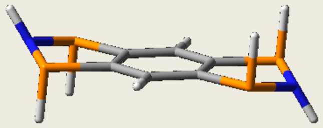

```
20
C6H8N2P4 @ PBE-D3/def2-TZVP
C           0.00110          1.20979         0.70515
P           0.00000          2.88067         1.48218
C           0.00110          1.20979        -0.70515
N          -0.27834          3.73361        -0.00000
P           0.00000          2.88067        -1.48218
H           1.43622          2.98868         1.51903
H           1.43622          2.98868        -1.51903
C           0.00000         -0.00000         1.40708
C          -0.00110         -1.20979         0.70515
H           0.00000         -0.00000         2.50050
P          -0.00000         -2.88067         1.48218
C          -0.00110         -1.20979        -0.70515
N           0.27834         -3.73361        -0.00000
C           0.00000         -0.00000        -1.40708
P          -0.00000         -2.88067        -1.48218
H           0.00000         -0.00000        -2.50050
H          -1.43622         -2.98868         1.51903
H          -1.43622         -2.98868        -1.51903
H           1.13020         -4.28843         0.00000
H          -1.13020          4.28843         0.00000
```

7.8.10 6EMind‡

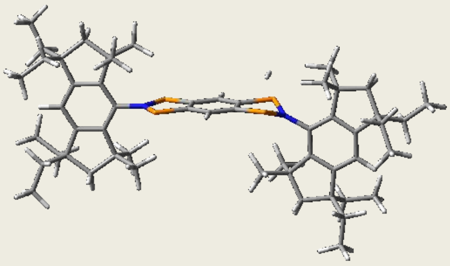

```
138
C54H78N2P4 @ PBE-D3/def2-TZVP
P           2.87257         -1.45467        -1.21903
N           3.70475         -0.90746         0.18341
C           1.23977         -1.11468        -0.54485
P           2.84339         -1.16938         1.64807
C           5.06106         -0.40051         0.12274
C           1.22584         -0.96206         0.88699
C           0.06218         -1.03472        -1.27439
C           6.19014         -1.24871         0.06419
C           5.24502          1.00077         0.08653
C           0.03427         -0.72841         1.55910
C          -1.16071         -0.79248        -0.60725
H           0.07678         -1.14220        -2.36214
C           6.29736         -2.77874         0.13686
C           7.47029         -0.68078        -0.08070
C           6.54120          1.53311        -0.01712
C           4.21210          2.13463         0.17211
C          -1.17439         -0.63317         0.83132
H           0.02677         -0.60342         2.64507
P          -2.71393         -0.63729        -1.41442
C           7.83127         -2.98751         0.31408
```

C	5.79750	-3.46168	-1.15058
C	5.58034	-3.39655	1.34544
C	8.56825	-1.72518	-0.18984
C	7.65118	0.69866	-0.11837
C	6.55661	3.05240	-0.02423
C	5.05957	3.36614	-0.26293
C	3.00784	2.00936	-0.77288
C	3.69225	2.30496	1.61355
P	-2.74293	-0.30963	1.55502
N	-3.55617	-0.34953	0.04835
H	8.04221	-3.11187	1.38829
H	8.16812	-3.90574	-0.18911
H	6.26984	-3.04095	-2.04780
H	4.71086	-3.35475	-1.26388
H	6.02643	-4.53780	-1.11464
H	5.83743	-2.86189	2.27127
H	5.89456	-4.44574	1.45998
H	4.49077	-3.38980	1.23379
C	9.76738	-1.37262	0.71761
C	9.01185	-1.91517	-1.66767
H	8.65088	1.12020	-0.22496
C	7.01886	3.62109	1.34650
C	7.43278	3.58833	-1.17876
H	4.90218	3.52770	-1.34157
H	4.72773	4.27959	0.25248
H	2.26982	1.28139	-0.41878
H	3.32349	1.71938	-1.78562
H	2.50168	2.98510	-0.84132
H	3.09330	1.43643	1.92357
H	3.05232	3.19842	1.67723
H	4.51087	2.41469	2.33631
C	-4.99176	-0.13087	0.00808
H	10.18549	-0.39932	0.41554
H	9.37753	-1.22724	1.73855
C	10.88513	-2.41443	0.73943
H	9.66578	-2.80162	-1.70995
H	8.12190	-2.17135	-2.26136
C	9.71716	-0.73050	-2.32366
H	6.83499	4.70792	1.33705
H	6.36001	3.21397	2.12740
C	8.47017	3.35448	1.73927
H	8.46661	3.22882	-1.05465
H	7.06605	3.12929	-2.11156
C	7.44308	5.10934	-1.32561
C	-5.50152	1.18252	-0.01170
C	-5.87104	-1.23146	-0.01465
H	11.66373	-2.13365	1.46287
H	11.37040	-2.51607	-0.24196
H	10.50726	-3.40645	1.02825
H	9.98572	-0.97324	-3.36184
H	10.64599	-0.45968	-1.80044
H	9.07220	0.15915	-2.34985
H	8.68952	3.80521	2.71787
H	9.18063	3.78124	1.01611
H	8.67546	2.27772	1.81973
H	8.02011	5.41007	-2.21163
H	7.89804	5.60262	-0.45462
H	6.42535	5.51075	-1.44151
C	-4.77590	2.53390	0.04411
C	-6.89287	1.37782	-0.06874

C	-7.25795	-1.00101	-0.04615
C	-5.58076	-2.73838	-0.03581
C	-5.89768	3.50093	-0.43328
C	-4.33914	2.86854	1.48568
C	-3.55678	2.67531	-0.87831
C	-7.27333	2.84856	-0.13982
C	-7.76974	0.29472	-0.08004
C	-8.06433	-2.29004	-0.01519
C	-6.96854	-3.32088	0.35982
C	-4.52152	-3.22566	0.96299
C	-5.17196	-3.20499	-1.44848
H	-5.79631	3.63420	-1.52256
H	-5.79063	4.49421	0.02683
H	-5.15953	2.74997	2.20458
H	-3.51957	2.21094	1.80896
H	-3.98318	3.90901	1.53496
H	-3.78421	2.33385	-1.89783
H	-3.26443	3.73566	-0.93129
H	-2.68871	2.11241	-0.51006
C	-8.26518	3.09106	-1.30099
C	-7.84577	3.37051	1.20708
H	-8.84669	0.45961	-0.11529
C	-8.67727	-2.62918	-1.40212
C	-9.15699	-2.21438	1.07626
H	-6.98169	-3.46716	1.45205
H	-7.13413	-4.30539	-0.10175
H	-3.50343	-2.95080	0.65680
H	-4.70234	-2.81970	1.96816
H	-4.56219	-4.32424	1.02658
H	-4.17447	-2.82564	-1.71253
H	-5.14027	-4.30475	-1.48283
H	-5.87150	-2.85553	-2.21820
H	-9.17912	2.49940	-1.13309
H	-7.80750	2.68097	-2.21616
C	-8.64533	4.55331	-1.52906
H	-7.93565	4.46621	1.12811
H	-7.09941	3.19005	1.99396
C	-9.18272	2.78377	1.65489
H	-9.09477	-3.64745	-1.34137
H	-7.86136	-2.68633	-2.13683
C	-9.74599	-1.67391	-1.92877
H	-9.83865	-1.37733	0.85642
H	-8.65833	-1.95274	2.02393
C	-9.97181	-3.49354	1.26220
H	-9.28461	4.65351	-2.41767
H	-9.20017	4.97142	-0.67691
H	-7.75653	5.18180	-1.68850
H	-9.49843	3.23812	2.60501
H	-9.98296	2.96750	0.92304
H	-9.11461	1.69889	1.81689
H	-10.12151	-2.02209	-2.90170
H	-10.60970	-1.60288	-1.25152
H	-9.34620	-0.66071	-2.07518
H	-10.66760	-3.39057	2.10696
H	-10.57032	-3.73264	0.37146
H	-9.32438	-4.35830	1.47046
H	2.65287	-2.93801	-0.09385
H	2.62982	-2.84085	0.83130

7.8.11 6EMind

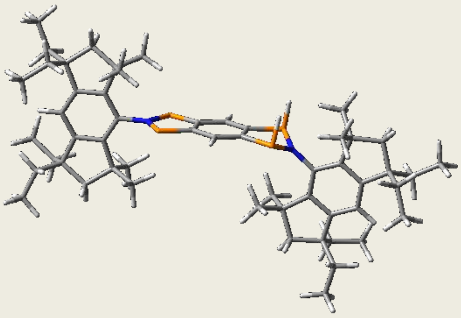

```
138
C54H78N2P4 @ PBE-D3/def2-TZVP
P          2.90361      -1.70493      -1.35867
N          3.75551      -1.16549       0.05228
C          1.24966      -1.39611      -0.61639
P          2.93106      -1.36793       1.56712
C          5.02324      -0.48202      -0.01254
C          1.26533      -1.21649       0.80742
C          0.07424      -1.27844      -1.32607
C          6.22687      -1.21869      -0.04092
C          5.08948       0.93013      -0.01928
C          0.10637      -0.91495       1.48915
C         -1.14118      -0.98935      -0.64503
H          0.06962      -1.39180      -2.41342
C          6.43757      -2.73305      -0.11997
C          7.46515      -0.55458      -0.03490
C          6.34241       1.56898       0.01059
C          3.96207       1.97130      -0.07026
C         -1.12411      -0.80026       0.78378
H          0.12540      -0.75396       2.57036
P         -2.69525      -0.79882      -1.42903
C          7.94493      -2.86344       0.24193
C          6.16507      -3.24295      -1.54982
C          5.60401      -3.56312       0.86441
C          8.64569      -1.51263      -0.05628
C          7.52750       0.83625       0.00943
C          6.22628       3.08403       0.03931
C          4.75150       3.27674      -0.38743
C          2.93510       1.73461      -1.18817
C          3.21065       2.09632       1.26896
P         -2.66030      -0.41112       1.52832
N         -3.50672      -0.44979       0.03910
H          8.02343      -3.07060       1.32146
H          8.41139      -3.70984      -0.28391
H          6.75445      -2.69804      -2.29793
H          5.10630      -3.12692      -1.81901
H          6.41573      -4.31251      -1.62420
H          5.64791      -3.14077       1.87903
H          6.00001      -4.58976       0.90695
H          4.55150      -3.62758       0.56033
C          9.65998      -1.15149       1.05255
C          9.32785      -1.55445      -1.45205
H          8.49076       1.34634       0.03691
C          6.44865       3.63611       1.47499
C          7.19475       3.73623      -0.97166
H          4.72133       3.44237      -1.47647
H          4.28186       4.15178       0.08576
H          2.23683       0.92404      -0.94831
H          3.43057       1.49976      -2.14082
H          2.33496       2.64712      -1.33091
H          2.60784       1.20240       1.47612
H          2.52589       2.95762       1.23166
H          3.89459       2.23592       2.11586
C         -4.93168      -0.17080       0.01784
H         10.03143      -0.12704       0.89119
H          9.10824      -1.12220       2.00649
C         10.84765      -2.10508       1.17633
```

H	10.04743	-2.38956	-1.44800
H	8.56650	-1.82098	-2.19969
C	10.03431	-0.27854	-1.90472
H	6.17172	4.70320	1.47207
H	5.73052	3.14218	2.14592
C	7.85276	3.47606	2.05330
H	8.23160	3.46512	-0.71685
H	6.99970	3.28056	-1.95643
C	7.08502	5.25661	-1.07990
C	-5.38490	1.16269	-0.02191
C	-5.85777	-1.23220	0.03350
H	11.47799	-1.83110	2.03429
H	11.48538	-2.08503	0.28085
H	10.51773	-3.14390	1.32657
H	10.48894	-0.42534	-2.89506
H	10.83941	0.01632	-1.21578
H	9.33336	0.56397	-1.98620
H	7.89863	3.89764	3.06776
H	8.61143	3.99478	1.44896
H	8.14185	2.41787	2.12303
H	7.74649	5.63875	-1.87043
H	7.37079	5.75622	-0.14303
H	6.05978	5.57113	-1.32543
C	-4.60049	2.48195	-0.00880
C	-6.76742	1.41702	-0.05892
C	-7.23378	-0.94295	0.01770
C	-5.63155	-2.74999	0.04390
C	-5.68896	3.48749	-0.48394
C	-4.11891	2.82687	1.41588
C	-3.39636	2.55100	-0.95896
C	-7.08530	2.90130	-0.15141
C	-7.69004	0.37280	-0.03437
C	-8.09366	-2.19538	0.08547
C	-7.03780	-3.26432	0.46825
C	-4.58220	-3.25753	1.04327
C	-5.25858	-3.26627	-1.36127
H	-5.60377	3.59543	-1.57739
H	-5.53006	4.48379	-0.04595
H	-4.92779	2.75824	2.15407
H	-3.32069	2.14231	1.73650
H	-3.71807	3.85184	1.43562
H	-3.66003	2.19815	-1.96587
H	-3.06043	3.59640	-1.04174
H	-2.54488	1.95969	-0.59647
C	-8.08907	3.16593	-1.29728
C	-7.60691	3.47173	1.19645
H	-8.75937	0.58324	-0.05575
C	-8.73730	-2.53517	-1.28736
C	-9.16807	-2.05159	1.18791
H	-7.04307	-3.38782	1.56335
H	-7.25131	-4.25002	0.02943
H	-3.55689	-3.03575	0.71873
H	-4.73286	-2.81838	2.03948
H	-4.66988	-4.35124	1.13631
H	-4.24952	-2.93530	-1.64573
H	-5.27206	-4.36691	-1.36951
H	-5.95244	-2.90695	-2.13158
H	-9.02417	2.61791	-1.10051
H	-7.66829	2.71997	-2.21339
C	-8.41021	4.63904	-1.54535

```
H          -7.65066          4.56871          1.09883
H          -6.85326          3.27263          1.97181
C          -8.95874          2.95207          1.68092
H          -9.19599         -3.53371         -1.20196
H          -7.93345         -2.64035         -2.03009
C          -9.77158         -1.54612         -1.82038
H          -9.81690         -1.19127          0.95886
H          -8.64683         -1.79196          2.12392
C         -10.03333         -3.29123          1.41016
H          -9.06291          4.75094         -2.42271
H          -8.92860          5.09628         -0.69036
H          -7.49864          5.22525         -1.73447
H          -9.23615          3.43814          2.62728
H          -9.76449          3.15585          0.96053
H          -8.93424          1.86865          1.86389
H         -10.17256         -1.89637         -2.78233
H         -10.62365         -1.42659         -1.13527
H          -9.33192         -0.55332         -1.99048
H         -10.71397         -3.14152          2.26022
H         -10.65198         -3.52345          0.53144
H          -9.42021         -4.17797          1.62955
H           2.86558         -3.12538         -1.07367
H           2.87380         -2.81276          1.61165
```

7.8.12 *syn*-7EMind‡

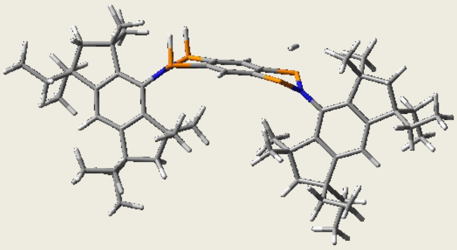

```
140
C54H80N2P4 @ PBE-D3/def2-TZVP
P          -2.83603         -2.17705         -1.17627
N          -3.54578         -1.41351          0.19329
C          -1.18154         -2.18845         -0.47711
P          -2.78563         -1.86117          1.67020
C          -4.72402         -0.57760          0.10899
C          -1.15578         -2.02190          0.93295
C           0.02026         -2.32772         -1.19558
C          -6.02938         -1.11449          0.04523
C          -4.54901          0.82509          0.06681
C           0.07210         -1.99470          1.61975
C           1.22938         -2.27733         -0.51113
H           0.00223         -2.42172         -2.28412
C          -6.51255         -2.56998          0.10513
C          -7.12696         -0.24524         -0.09812
C          -5.67122          1.66484         -0.03579
C          -3.26378          1.66500          0.13890
C           1.25530         -2.11214          0.89971
H           0.09378         -1.83818          2.70111
P           2.88909         -2.25893         -1.31006
C          -8.04937         -2.39248          0.28818
C          -6.19915         -3.33709         -1.19391
C          -5.96753         -3.36397          1.30031
C          -8.45124         -0.98246         -0.20438
C          -6.95561          1.13564         -0.13248
C          -5.30604          3.13966         -0.04430
C          -3.78017          3.06866         -0.29448
C          -2.14042          1.23980         -0.81837
C          -2.69831          1.70532          1.57258
P           2.94291         -1.94478          1.61293
N           3.66344         -1.57018          0.07926
```

H	-8.28087	-2.46832	1.36268
H	-8.60564	-3.19455	-0.21886
H	-6.55762	-2.80196	-2.08276
H	-5.11937	-3.49638	-1.31360
H	-6.68232	-4.32586	-1.16977
H	-6.07057	-2.79250	2.23405
H	-6.53892	-4.29896	1.40918
H	-4.91403	-3.63676	1.17529
C	-9.51767	-0.34716	0.71485
C	-8.93890	-1.04591	-1.67923
H	-7.81781	1.79509	-0.23348
C	-5.59979	3.80313	1.33021
C	-6.02892	3.87969	-1.19202
H	-3.59592	3.18436	-1.37472
H	-3.22655	3.87090	0.21549
H	-1.60287	0.35265	-0.46753
H	-2.52984	1.03543	-1.82634
H	-1.40665	2.05746	-0.89793
H	-2.33235	0.71623	1.88301
H	-1.85203	2.40799	1.62077
H	-3.45194	2.02244	2.30467
C	4.74874	-0.62587	-0.01545
H	-9.68021	0.70202	0.42121
H	-9.09676	-0.31123	1.73317
C	-10.86087	-1.07535	0.74103
H	-9.79401	-1.74041	-1.71955
H	-8.14601	-1.51270	-2.28226
C	-9.33057	0.28153	-2.32388
H	-5.14830	4.80869	1.31994
H	-5.05868	3.24115	2.10551
C	-7.06827	3.90945	1.73493
H	-7.11886	3.79006	-1.05993
H	-5.79586	3.34507	-2.12757
C	-5.65952	5.35510	-1.33939
C	4.50889	0.76785	-0.01267
C	6.08063	-1.08716	-0.08254
H	-11.53880	-0.61328	1.47265
H	-11.36323	-1.04498	-0.23653
H	-10.74219	-2.13248	1.02161
H	-9.66102	0.11981	-3.35997
H	-10.15694	0.77355	-1.79023
H	-8.48347	0.98127	-2.35413
H	-7.15909	4.39687	2.71627
H	-7.65368	4.50467	1.01888
H	-7.53806	2.91900	1.81508
H	-6.15012	5.79200	-2.22076
H	-5.96976	5.94506	-0.46498
H	-4.57474	5.48961	-1.46391
C	3.18396	1.54593	-0.02627
C	5.59719	1.65961	-0.01046
C	7.14761	-0.17365	-0.10818
C	6.60672	-2.52184	-0.17570
C	3.66913	2.99199	-0.34515
C	2.45305	1.49934	1.32922
C	2.20414	1.10566	-1.12468
C	5.16197	3.11468	0.04136
C	6.91121	1.19793	-0.05323
C	8.50413	-0.85712	-0.17382
C	8.11833	-2.32652	0.13705
C	6.00248	-3.50840	0.83182

```
C           6.40124       -3.08146       -1.59789
H           3.57418        3.15644       -1.43062
H           3.03726        3.74290        0.15191
H           3.10761        1.78415        2.16280
H           2.06497        0.49489        1.54244
H           1.59441        2.18864        1.31203
H           2.71449        0.98848       -2.09102
H           1.42066        1.87103       -1.24227
H           1.69785        0.16511       -0.87917
C           5.94101        3.96758       -0.98370
C           5.30077        3.68848        1.47924
H           7.74284        1.90305       -0.04926
C           9.13255       -0.75247       -1.59134
C           9.45421       -0.28759        0.90401
H           8.27438       -2.51214        1.21205
H           8.73762       -3.05350       -0.40925
H           4.98073       -3.80147        0.55915
H           5.98388       -3.08204        1.84559
H           6.61318       -4.42427        0.86044
H           5.33326       -3.19135       -1.83152
H           6.86854       -4.07457       -1.68524
H           6.83777       -2.42672       -2.36253
H           7.01843        3.92189       -0.75883
H           5.82057        3.48997       -1.97002
C           5.50659        5.43038       -1.06600
H           4.80168        4.67129        1.49923
H           4.72410        3.04574        2.16062
C           6.72203        3.82819        2.01957
H          10.01404       -1.41397       -1.61629
H           8.42150       -1.17629       -2.31561
C           9.53370        0.64508       -2.05797
H           9.59289        0.79252        0.73769
H           8.94102       -0.37668        1.87562
C          10.82138       -0.96562        0.98213
H           6.04978        5.95288       -1.86626
H           5.70426        5.97004       -0.12861
H           4.43161        5.52096       -1.28181
H           6.70451        4.23824        3.03960
H           7.33402        4.50508        1.40544
H           7.23377        2.85640        2.06395
H           9.97499        0.59914       -3.06402
H          10.28021        1.10545       -1.39422
H           8.66634        1.31803       -2.10968
H          11.40687       -0.56366        1.82124
H          11.41010       -0.80951        0.06672
H          10.72604       -2.05096        1.13529
H          -2.98351       -3.61406       -0.05321
H          -2.95303       -3.50707        0.89543
H           3.13544       -3.66051       -1.03682
H           3.16835       -3.37353        1.63517
```

7.8.13 *syn*-7EMind

```
140
C54H80N2P4 @ PBE-D3/def2-TZVP
P           2.34597       -1.29352        2.87673
N           1.61395        0.09314        3.61422
C           2.39735       -0.51338        1.20808
```

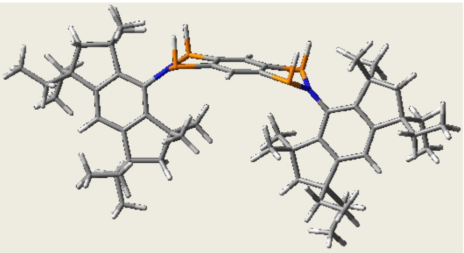

P	1.96356	1.62336	2.87516
C	0.64312	-0.00217	4.67628
C	2.20171	0.88325	1.20789
C	2.49472	-1.21153	0.00000
C	1.07062	-0.02823	6.02061
C	-0.74338	-0.03669	4.39999
C	2.10099	1.58028	0.00000
C	2.39735	-0.51338	-1.20808
H	2.60013	-2.29930	0.00000
C	2.49254	-0.08210	6.58490
C	0.13015	-0.04755	7.06392
C	-1.66328	-0.02582	5.46460
C	-1.48587	-0.10421	3.05632
C	2.20171	0.88325	-1.20789
H	1.90814	2.65612	0.00000
P	2.34597	-1.29352	-2.87673
C	2.25264	0.25806	8.08418
C	3.08289	-1.49753	6.42296
C	3.47569	0.93127	5.98482
C	0.77902	-0.07012	8.43863
C	-1.23561	-0.02551	6.79074
C	-3.10704	-0.01610	4.99059
C	-2.93690	-0.44193	3.51335
C	-0.99682	-1.22050	2.12087
C	-1.45003	1.22996	2.28664
P	1.96356	1.62336	-2.87516
N	1.61395	0.09314	-3.61422
H	2.41650	1.33896	8.22262
H	2.97209	-0.26305	8.73322
H	2.43101	-2.26508	6.85872
H	3.22396	-1.75046	5.36298
H	4.06527	-1.55723	6.91652
H	3.03200	1.93657	5.93586
H	4.37545	0.98854	6.61705
H	3.79864	0.64418	4.97614
C	0.16590	1.01837	9.34848
C	0.68273	-1.47495	9.09626
H	-1.96222	-0.01439	7.60363
C	-3.71237	1.41313	5.07223
C	-3.96033	-1.03555	5.77645
H	-3.07512	-1.53308	3.44565
H	-3.68157	0.02001	2.84833
H	-0.05124	-0.96713	1.62769
H	-0.86855	-2.16799	2.66276
H	-1.74096	-1.38017	1.32465
H	-0.44244	1.45121	1.91160
H	-2.12102	1.17418	1.41531
H	-1.76396	2.07689	2.90990
C	0.64312	-0.00217	-4.67628
H	-0.91449	0.83588	9.46243
H	0.25179	1.97966	8.81569
C	0.80566	1.13962	10.73091
H	1.32074	-1.46775	9.99519
H	1.13852	-2.20699	8.41343
C	-0.71636	-1.95842	9.47119
H	-4.68242	1.39824	4.54866
H	-3.06892	2.09041	4.49155
C	-3.89837	1.99180	6.47298
H	-3.94897	-0.77818	6.84746
H	-3.45991	-2.01481	5.69848

C	-5.40867	-1.16123	5.30543
C	-0.74338	-0.03669	-4.39999
C	1.07062	-0.02823	-6.02061
H	0.37305	1.98393	11.28627
H	0.65046	0.23484	11.33606
H	1.89022	1.31090	10.66072
H	-0.66387	-2.95286	9.93732
H	-1.20865	-1.28634	10.18932
H	-1.36478	-2.04312	8.58788
H	-4.32819	3.00214	6.41517
H	-4.57784	1.38183	7.08623
H	-2.94072	2.07122	7.00668
H	-5.92963	-1.95575	5.85841
H	-5.97251	-0.22999	5.45998
H	-5.46534	-1.41091	4.23548
C	-1.48587	-0.10421	-3.05632
C	-1.66328	-0.02582	-5.46460
C	0.13015	-0.04755	-7.06392
C	2.49254	-0.08210	-6.58490
C	-2.93690	-0.44193	-3.51335
C	-1.45003	1.22996	-2.28664
C	-0.99682	-1.22050	-2.12087
C	-3.10704	-0.01610	-4.99059
C	-1.23561	-0.02551	-6.79074
C	0.77902	-0.07012	-8.43863
C	2.25264	0.25806	-8.08418
C	3.47569	0.93127	-5.98482
C	3.08289	-1.49753	-6.42296
H	-3.07512	-1.53308	-3.44565
H	-3.68157	0.02001	-2.84833
H	-1.76396	2.07689	-2.90990
H	-0.44244	1.45121	-1.91160
H	-2.12102	1.17418	-1.41531
H	-0.86855	-2.16799	-2.66276
H	-1.74096	-1.38017	-1.32465
H	-0.05124	-0.96713	-1.62769
C	-3.96033	-1.03555	-5.77645
C	-3.71237	1.41313	-5.07223
H	-1.96222	-0.01439	-7.60363
C	0.68273	-1.47495	-9.09626
C	0.16590	1.01837	-9.34848
H	2.41650	1.33896	-8.22262
H	2.97209	-0.26305	-8.73322
H	3.79864	0.64418	-4.97614
H	3.03200	1.93657	-5.93586
H	4.37545	0.98854	-6.61705
H	3.22396	-1.75046	-5.36298
H	4.06527	-1.55723	-6.91652
H	2.43101	-2.26508	-6.85872
H	-3.94897	-0.77818	-6.84746
H	-3.45991	-2.01481	-5.69848
C	-5.40867	-1.16123	-5.30543
H	-4.68242	1.39824	-4.54866
H	-3.06892	2.09041	-4.49155
C	-3.89837	1.99180	-6.47298
H	1.32074	-1.46775	-9.99519
H	1.13852	-2.20699	-8.41343
C	-0.71636	-1.95842	-9.47119
H	-0.91449	0.83588	-9.46243
H	0.25179	1.97966	-8.81569

```
C          0.80566          1.13962        -10.73091
H         -5.92963         -1.95575         -5.85841
H         -5.97251         -0.22999         -5.45998
H         -5.46534         -1.41091         -4.23548
H         -4.32819          3.00214         -6.41517
H         -4.57784          1.38183         -7.08623
H         -2.94072          2.07122         -7.00668
H         -0.66387         -2.95286         -9.93732
H         -1.20865         -1.28634        -10.18932
H         -1.36478         -2.04312         -8.58788
H          0.37305          1.98393        -11.28627
H          0.65046          0.23484        -11.33606
H          1.89022          1.31090        -10.66072
H          3.73729         -0.99730          3.15347
H          3.38446          1.69792          3.13748
H          3.73729         -0.99730         -3.15347
H          3.38446          1.69792         -3.13748
```

7.8.14 *anti*-7EMind‡

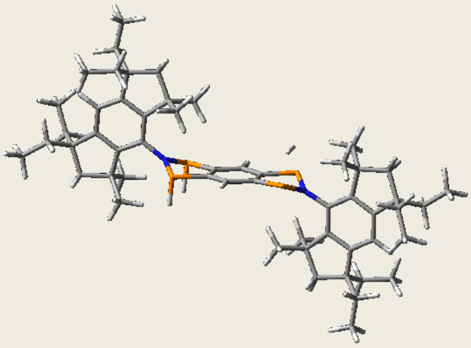

```
140
C54H80N2P4 @ PBE-D3/def2-TZVP
P          2.76636          1.15479         -1.40903
N          3.65436          0.76628          0.02767
C          1.16654          0.56630         -0.71650
P          2.75967          0.77918          1.51455
C          5.02957          0.33230         -0.00354
C          1.16705          0.37342          0.69073
C          0.02805          0.29726         -1.46728
C          6.07432          1.28127          0.01483
C          5.35830         -1.04242         -0.02928
C          0.02961         -0.09229          1.34025
C         -1.13903         -0.13959         -0.81345
H          0.03429          0.42949         -2.55234
C          6.00119          2.80994         -0.03286
C          7.41432          0.85928          0.04470
C          6.70746         -1.43697          0.02394
C          4.44693         -2.27455         -0.12456
C         -1.13778         -0.33567          0.59304
H          0.03512         -0.25550          2.42109
P         -2.68054         -0.61514         -1.60462
C          7.44821          3.20978          0.37507
C          5.67580          3.29301         -1.46091
C          5.00292          3.44890          0.94068
C          8.39565          2.02039          0.07190
C          7.73427         -0.49617          0.06648
C          6.87648         -2.94727          0.02276
C          5.47304         -3.40275         -0.44361
C          3.41596         -2.21247         -1.26194
C          3.70601         -2.56405          1.19490
P         -2.68264         -0.99461          1.23091
N         -3.59949         -0.53712         -0.15216
H          7.45965          3.40164          1.46022
H          7.76145          4.14066         -0.12070
H          6.37373          2.88220         -2.20110
H          4.66326          2.99227         -1.76246
H          5.72800          4.39176         -1.50927
H          5.09908          3.02013          1.94903
```

H	5.19947	4.53004	1.01190
H	3.96498	3.32327	0.60730
C	9.43400	1.83017	1.20092
C	9.09027	2.21883	-1.30421
H	8.77504	-0.81818	0.10988
C	7.16908	-3.48084	1.45302
C	7.97085	-3.38418	-0.97579
H	5.49742	-3.54843	-1.53562
H	5.16545	-4.35966	0.00337
H	2.57201	-1.55422	-1.02354
H	3.87621	-1.86783	-2.19867
H	3.00273	-3.21877	-1.43424
H	2.94530	-1.80040	1.40420
H	3.19330	-3.53596	1.12788
H	4.38777	-2.59204	2.05449
C	-5.01956	-0.26112	-0.10078
H	9.99355	0.89645	1.03179
H	8.87607	1.67825	2.13949
C	10.41961	2.98538	1.37133
H	9.64060	3.17326	-1.26677
H	8.31048	2.35433	-2.06786
C	10.03345	1.10719	-1.75873
H	7.09959	-4.58061	1.42242
H	6.35652	-3.14801	2.11547
C	8.50484	-3.07212	2.06974
H	8.93308	-2.93034	-0.69012
H	7.71481	-2.95081	-1.95665
C	8.14960	-4.89555	-1.11417
C	-5.99304	-1.28592	-0.09394
C	-5.43599	1.08808	-0.03006
H	11.07046	2.81571	2.24086
H	11.06989	3.10309	0.49259
H	9.89824	3.94119	1.52981
H	10.47560	1.35741	-2.73386
H	10.86315	0.95320	-1.05331
H	9.50470	0.15056	-1.87315
H	8.60797	-3.50125	3.07679
H	9.36086	-3.42371	1.47519
H	8.58644	-1.98029	2.16695
H	8.88845	-5.12979	-1.89373
H	8.50242	-5.35411	-0.17914
H	7.20698	-5.38998	-1.39225
C	-5.84026	-2.80995	-0.19896
C	-7.35333	-0.94367	0.02554
C	-6.80524	1.39472	0.04532
C	-4.60400	2.37897	-0.04493
C	-7.31175	-3.26745	-0.42846
C	-5.26967	-3.42692	1.09254
C	-4.99527	-3.27477	-1.39315
C	-8.26366	-2.15886	0.07733
C	-7.76298	0.38508	0.08822
C	-7.07355	2.88948	0.09157
C	-5.65784	3.43898	0.39146
C	-3.43016	2.41897	0.94471
C	-4.06965	2.68235	-1.45876
H	-7.46884	-3.39791	-1.51115
H	-7.50358	-4.24159	0.04489
H	-5.83330	-3.11324	1.98072
H	-4.22099	-3.13961	1.24428
H	-5.30949	-4.52524	1.03070

```
H          -5.30262        -2.76367        -2.31694
H          -5.13534        -4.35699        -1.54066
H          -3.92457        -3.10119        -1.24067
C          -9.47813        -1.98714        -0.86157
C          -8.71200        -2.45791         1.53553
H          -8.82184         0.63134         0.17008
C          -7.57965         3.41715        -1.27984
C          -8.06267         3.23610         1.22699
H          -5.56297         3.59096         1.47872
H          -5.46726         4.41038        -0.08828
H          -2.57093         1.83355         0.60025
H          -3.73084         2.04616         1.93473
H          -3.09388         3.46105         1.06246
H          -3.32471         1.93592        -1.76982
H          -3.58408         3.67022        -1.47146
H          -4.86965         2.68182        -2.21016
H         -10.06052        -1.10425        -0.55376
H          -9.08863        -1.75364        -1.86617
C         -10.40613        -3.19860        -0.94045
H          -9.20710        -3.44283         1.53848
H          -7.80952        -2.57484         2.15364
C          -9.62731        -1.42637         2.19082
H          -7.58021         4.51853        -1.23482
H          -6.83691         3.15032        -2.04594
C          -8.95256         2.92651        -1.73396
H          -9.01784         2.71485         1.05552
H          -7.65465         2.81409         2.16017
C          -8.33049         4.72886         1.41386
H         -11.19960        -3.03245        -1.68284
H         -10.89546        -3.40368         0.02257
H          -9.86065        -4.10688        -1.23675
H          -9.88158        -1.73732         3.21429
H         -10.57262        -1.30336         1.64216
H          -9.14400        -0.44136         2.25669
H          -9.21143         3.36569        -2.70812
H          -9.74765         3.20669        -1.02749
H          -8.97332         1.83371        -1.85010
H          -8.97702         4.90109         2.28605
H          -8.83380         5.16796         0.54050
H          -7.39799         5.28925         1.57737
H           2.46599         2.54604        -1.13485
H           2.44780         2.18946         1.60058
H          -2.20841        -2.20274        -0.86021
H          -2.22105        -2.33745         0.09455
```

7.8.15 *anti*-7EMind

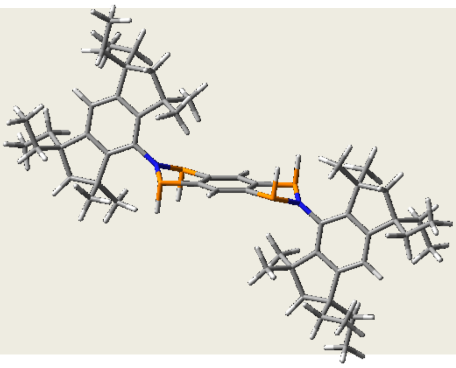

```
140
C54H80N2P4 @ PBE-D3/def2-TZVP
P           1.39595         1.20822         2.65661
N          -0.02185         0.87382         3.59477
C           0.66902         0.52816         1.10861
P          -1.52554         0.81915         2.73135
C           0.03868         0.51997         4.99216
C          -0.72677         0.33187         1.14836
C           1.39509         0.19725        -0.03984
C           0.03030         1.52824         5.97966
C           0.08254        -0.83301         5.39966
```

C	-1.39509	-0.19725	0.03984
C	0.72677	-0.33187	-1.14836
H	2.47749	0.34765	-0.06946
C	0.06352	3.05026	5.81579
C	0.02738	1.18554	7.34239
C	0.05619	-1.14804	6.77049
C	0.17302	-2.11613	4.56074
C	-0.66902	-0.52816	-1.10861
H	-2.47749	-0.34765	0.06946
P	1.52554	-0.81915	-2.73135
C	-0.32317	3.53140	7.24380
C	1.48173	3.52345	5.43723
C	-0.93223	3.62293	4.79927
C	0.00577	2.40210	8.25419
C	0.02295	-0.14903	7.74113
C	0.07237	-2.64582	7.02755
C	0.51970	-3.17917	5.64565
C	1.29170	-2.10632	3.50776
C	-1.15620	-2.45978	3.86169
P	-1.39595	-1.20822	-2.65661
N	0.02185	-0.87382	-3.59477
H	-1.40952	3.71566	7.26261
H	0.16988	4.48274	7.49343
H	2.23723	3.16055	6.14523
H	1.76818	3.16501	4.43915
H	1.52179	4.62364	5.42289
H	-1.93482	3.19181	4.93646
H	-1.01057	4.71280	4.93567
H	-0.61455	3.44191	3.76466
C	-1.10489	2.26385	9.32009
C	1.39104	2.65228	8.91289
H	-0.00033	-0.40974	8.79950
C	-1.34881	-3.17072	7.37470
C	1.09207	-3.01124	8.12855
H	1.61332	-3.31371	5.66003
H	0.07705	-4.15641	5.40234
H	1.03173	-1.50243	2.63012
H	2.23287	-1.72622	3.92928
H	1.46658	-3.13420	3.15282
H	-1.38737	-1.74235	3.06325
H	-1.08785	-3.45849	3.40357
H	-2.00323	-2.45767	4.55949
C	-0.03868	-0.51997	-4.99216
H	-0.91820	1.36620	9.93075
H	-2.05057	2.07120	8.78731
C	-1.27112	3.47349	10.23891
H	1.35423	3.63772	9.40555
H	2.14074	2.74676	8.11372
C	1.87044	1.60294	9.91339
H	-1.31236	-4.27249	7.36508
H	-2.02777	-2.88672	6.55728
C	-1.94446	-2.69250	8.69686
H	0.81883	-2.50364	9.06721
H	2.06531	-2.58726	7.83109
C	1.24460	-4.50859	8.39323
C	-0.03030	-1.52824	-5.97966
C	-0.08254	0.83301	-5.39966
H	-2.12852	3.33453	10.91269
H	-0.38344	3.63718	10.86673
H	-1.44735	4.39553	9.66508

H	2.85063	1.88665	10.32287
H	1.18030	1.49429	10.76289
H	1.98423	0.61690	9.44160
H	-2.94660	-3.12106	8.84159
H	-1.33243	-2.99310	9.55992
H	-2.04773	-1.59842	8.72024
H	2.03812	-4.69389	9.13109
H	0.31909	-4.95157	8.78841
H	1.51061	-5.05592	7.47671
C	-0.06352	-3.05026	-5.81579
C	-0.02738	-1.18554	-7.34239
C	-0.05619	1.14804	-6.77049
C	-0.17302	2.11613	-4.56074
C	0.32317	-3.53140	-7.24380
C	-1.48173	-3.52345	-5.43723
C	0.93223	-3.62293	-4.79927
C	-0.00577	-2.40210	-8.25419
C	-0.02295	0.14903	-7.74113
C	-0.07237	2.64582	-7.02755
C	-0.51970	3.17917	-5.64565
C	-1.29170	2.10632	-3.50776
C	1.15620	2.45978	-3.86169
H	1.40952	-3.71566	-7.26261
H	-0.16988	-4.48274	-7.49343
H	-2.23723	-3.16055	-6.14523
H	-1.76818	-3.16501	-4.43915
H	-1.52179	-4.62364	-5.42289
H	1.93482	-3.19181	-4.93646
H	1.01057	-4.71280	-4.93567
H	0.61455	-3.44191	-3.76466
C	1.10489	-2.26385	-9.32009
C	-1.39104	-2.65228	-8.91289
H	0.00033	0.40974	-8.79950
C	1.34881	3.17072	-7.37470
C	-1.09207	3.01124	-8.12855
H	-1.61332	3.31371	-5.66003
H	-0.07705	4.15641	-5.40234
H	-1.03173	1.50243	-2.63012
H	-2.23287	1.72622	-3.92928
H	-1.46658	3.13420	-3.15282
H	1.38737	1.74235	-3.06325
H	1.08785	3.45849	-3.40357
H	2.00323	2.45767	-4.55949
H	0.91820	-1.36620	-9.93075
H	2.05057	-2.07120	-8.78731
C	1.27112	-3.47349	-10.23891
H	-1.35423	-3.63772	-9.40555
H	-2.14074	-2.74676	-8.11372
C	-1.87044	-1.60294	-9.91339
H	1.31236	4.27249	-7.36508
H	2.02777	2.88672	-6.55728
C	1.94446	2.69250	-8.69686
H	-0.81883	2.50364	-9.06721
H	-2.06531	2.58726	-7.83109
C	-1.24460	4.50859	-8.39323
H	2.12852	-3.33453	-10.91269
H	0.38344	-3.63718	-10.86673
H	1.44735	-4.39553	-9.66508
H	-2.85063	-1.88665	-10.32287
H	-1.18030	-1.49429	-10.76289

```
H          -1.98423        -0.61690        -9.44160
H           2.94660         3.12106        -8.84159
H           1.33243         2.99310        -9.55992
H           2.04773         1.59842        -8.72024
H          -2.03812         4.69389        -9.13109
H          -0.31909         4.95157        -8.78841
H          -1.51061         5.05592        -7.47671
H           1.11836         2.57950         2.27728
H          -1.63809         2.20701         2.33724
H           1.63809        -2.20701        -2.33724
H          -1.11836        -2.57950        -2.27728
```

7.8.16 1Ter

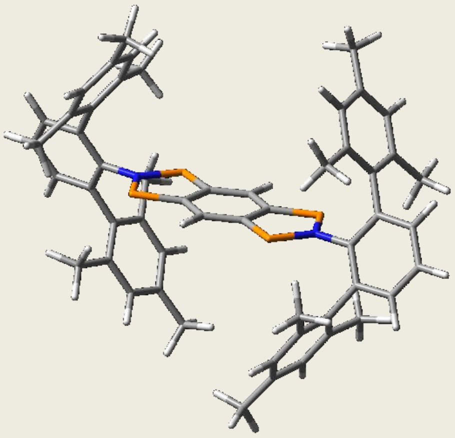

```
112
C54H52N2P4 @ PBE-D3/def2-SVP
C          -1.42976         0.00000         0.00000
C          -0.72970         0.00208        -1.22349
H          -2.53225         0.00000         0.00000
C           1.42976        -0.00000         0.00000
P          -1.51257         0.01505        -2.81236
C           0.72970        -0.00208        -1.22349
H           2.53225        -0.00000         0.00000
N           0.00000         0.00000        -3.66053
P           1.51257        -0.01505        -2.81236
C           0.00000         0.00000        -5.10439
C          -0.48853         1.13562        -5.80455
C           0.48853        -1.13562        -5.80455
C          -0.47751         1.11241        -7.21339
C          -0.99601         2.34899        -5.09297
C           0.47751        -1.11241        -7.21339
C           0.99601        -2.34899        -5.09297
H          -0.84748         1.99738        -7.75309
C           0.00000         0.00000        -7.91701
C          -0.08156         3.31103        -4.59378
C          -2.39296         2.54940        -4.95253
H           0.84748        -1.99738        -7.75309
C           2.39296        -2.54940        -4.95253
C           0.08156        -3.31103        -4.59378
H           0.00000         0.00000        -9.01744
C          -0.58422         4.43485        -3.91488
C           1.40356         3.14867        -4.80154
C          -2.84868         3.68685        -4.26187
C          -3.37908         1.56183        -5.52553
C           2.84868        -3.68685        -4.26187
C           3.37908        -1.56183        -5.52553
C           0.58422        -4.43485        -3.91488
C          -1.40356        -3.14867        -4.80154
H           0.12893         5.18017        -3.52605
C          -1.96283         4.63586        -3.72256
H           1.95390         4.05616        -4.48727
H           1.64559         2.94305        -5.86419
H           1.80205         2.29320        -4.21409
H          -3.93457         3.83311        -4.14155
H          -3.28929         1.48566        -6.62854
H          -4.42005         1.85050        -5.28444
```

H	-3.20591	0.54109	-5.12535
H	3.93457	-3.83311	-4.14155
C	1.96283	-4.63586	-3.72256
H	3.28929	-1.48566	-6.62854
H	4.42005	-1.85050	-5.28444
H	3.20591	-0.54109	-5.12535
H	-0.12893	-5.18017	-3.52605
H	-1.95390	-4.05616	-4.48727
H	-1.64559	-2.94305	-5.86419
H	-1.80205	-2.29320	-4.21409
C	-2.47154	5.82257	-2.94316
C	2.47154	-5.82257	-2.94316
H	-3.50841	6.08739	-3.22957
H	-1.83181	6.71517	-3.09283
H	-2.47734	5.60734	-1.85262
H	1.83181	-6.71517	-3.09283
H	2.47734	-5.60734	-1.85262
H	3.50841	-6.08739	-3.22957
C	-0.72970	-0.00208	1.22349
P	-1.51257	-0.01505	2.81236
C	0.72970	0.00208	1.22349
N	-0.00000	0.00000	3.66053
P	1.51257	0.01505	2.81236
C	-0.00000	0.00000	5.10439
C	-0.48853	-1.13562	5.80455
C	0.48853	1.13562	5.80455
C	-0.47751	-1.11241	7.21339
C	-0.99601	-2.34899	5.09297
C	0.47751	1.11241	7.21339
C	0.99601	2.34899	5.09297
H	-0.84748	-1.99738	7.75309
C	-0.00000	0.00000	7.91701
C	-0.08156	-3.31103	4.59378
C	-2.39296	-2.54940	4.95253
H	0.84748	1.99738	7.75309
C	2.39296	2.54940	4.95253
C	0.08156	3.31103	4.59378
H	-0.00000	0.00000	9.01744
C	-0.58422	-4.43485	3.91488
C	1.40356	-3.14867	4.80154
C	-2.84868	-3.68685	4.26187
C	-3.37908	-1.56183	5.52553
C	2.84868	3.68685	4.26187
C	3.37908	1.56183	5.52553
C	0.58422	4.43485	3.91488
C	-1.40356	3.14867	4.80154
H	0.12893	-5.18017	3.52605
C	-1.96283	-4.63586	3.72256
H	1.95390	-4.05616	4.48727
H	1.64559	-2.94305	5.86419
H	1.80205	-2.29320	4.21409
H	-3.93457	-3.83311	4.14155
H	-3.28929	-1.48566	6.62854
H	-4.42005	-1.85050	5.28444
H	-3.20591	-0.54109	5.12535
H	3.93457	3.83311	4.14155
C	1.96283	4.63586	3.72256
H	3.28929	1.48566	6.62854
H	4.42005	1.85050	5.28444
H	3.20591	0.54109	5.12535

```
H          -0.12893         5.18017         3.52605
H          -1.95390         4.05616         4.48727
H          -1.64559         2.94305         5.86419
H          -1.80205         2.29320         4.21409
C          -2.47154        -5.82257         2.94316
C           2.47154         5.82257         2.94316
H          -3.50841        -6.08739         3.22957
H          -1.83181        -6.71517         3.09283
H          -2.47734        -5.60734         1.85262
H           1.83181         6.71517         3.09283
H           2.47734         5.60734         1.85262
H           3.50841         6.08739         3.22957
```

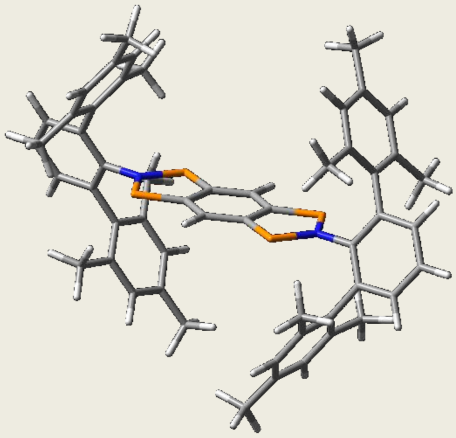

```
112
C54H52N2P4 @ PBE-D3/def2-TZVP
C          -1.42430         0.00000         0.00000
C          -0.72706         0.00182        -1.21651
H          -2.51766         0.00000         0.00000
C           1.42430        -0.00000         0.00000
P          -1.49775         0.01285        -2.79960
C           0.72706        -0.00182        -1.21651
H           2.51766        -0.00000         0.00000
N           0.00000         0.00000        -3.63615
P           1.49775        -0.01285        -2.79960
C           0.00000         0.00000        -5.08099
C          -0.46022         1.14014        -5.77547
C           0.46022        -1.14014        -5.77547
C          -0.45209         1.11593        -7.17668
C          -0.94021         2.36462        -5.07099
C           0.45209        -1.11593        -7.17668
C           0.94021        -2.36462        -5.07099
H          -0.80166         2.00028        -7.71232
C           0.00000         0.00000        -7.87669
C          -0.01281         3.31150        -4.59003
C          -2.32469         2.59354        -4.93055
H           0.80166        -2.00028        -7.71232
C           2.32469        -2.59354        -4.93055
C           0.01281        -3.31150        -4.59003
H           0.00000         0.00000        -8.96782
C          -0.48928         4.44583        -3.92462
C           1.46682         3.13165        -4.80233
C          -2.75543         3.73961        -4.25378
C          -3.33502         1.63255        -5.49905
C           2.75543        -3.73961        -4.25378
C           3.33502        -1.63255        -5.49905
C           0.48928        -4.44583        -3.92462
C          -1.46682        -3.13165        -4.80233
H           0.23271         5.17753        -3.55139
C          -1.85511         4.67130        -3.72904
H           2.01268         4.04519        -4.53259
H           1.69659         2.88497        -5.84934
H           1.86391         2.30888        -4.18558
H          -3.82938         3.90652        -4.13341
H          -3.29213         1.60602        -6.59858
H          -4.35294         1.91946        -5.20493
H          -3.15523         0.60485        -5.14990
H           3.82938        -3.90652        -4.13341
C           1.85511        -4.67130        -3.72904
```

H	3.29213	-1.60602	-6.59858
H	4.35294	-1.91946	-5.20493
H	3.15523	-0.60485	-5.14990
H	-0.23271	-5.17753	-3.55139
H	-2.01268	-4.04519	-4.53259
H	-1.69659	-2.88497	-5.84934
H	-1.86391	-2.30888	-4.18558
C	-2.33978	5.87219	-2.96185
C	2.33978	-5.87219	-2.96185
H	-3.34749	6.17299	-3.28012
H	-1.66586	6.73063	-3.09102
H	-2.38776	5.65422	-1.88269
H	1.66586	-6.73063	-3.09102
H	2.38776	-5.65422	-1.88269
H	3.34749	-6.17299	-3.28012
C	-0.72706	-0.00182	1.21651
P	-1.49775	-0.01285	2.79960
C	0.72706	0.00182	1.21651
N	-0.00000	0.00000	3.63615
P	1.49775	0.01285	2.79960
C	-0.00000	0.00000	5.08099
C	-0.46022	-1.14014	5.77547
C	0.46022	1.14014	5.77547
C	-0.45209	-1.11593	7.17668
C	-0.94021	-2.36462	5.07099
C	0.45209	1.11593	7.17668
C	0.94021	2.36462	5.07099
H	-0.80166	-2.00028	7.71232
C	-0.00000	0.00000	7.87669
C	-0.01281	-3.31150	4.59003
C	-2.32469	-2.59354	4.93055
H	0.80166	2.00028	7.71232
C	2.32469	2.59354	4.93055
C	0.01281	3.31150	4.59003
H	-0.00000	0.00000	8.96782
C	-0.48928	-4.44583	3.92462
C	1.46682	-3.13165	4.80233
C	-2.75543	-3.73961	4.25378
C	-3.33502	-1.63255	5.49905
C	2.75543	3.73961	4.25378
C	3.33502	1.63255	5.49905
C	0.48928	4.44583	3.92462
C	-1.46682	3.13165	4.80233
H	0.23271	-5.17753	3.55139
C	-1.85511	-4.67130	3.72904
H	2.01268	-4.04519	4.53259
H	1.69659	-2.88497	5.84934
H	1.86391	-2.30888	4.18558
H	-3.82938	-3.90652	4.13341
H	-3.29213	-1.60602	6.59858
H	-4.35294	-1.91946	5.20493
H	-3.15523	-0.60485	5.14990
H	3.82938	3.90652	4.13341
C	1.85511	4.67130	3.72904
H	3.29213	1.60602	6.59858
H	4.35294	1.91946	5.20493
H	3.15523	0.60485	5.14990
H	-0.23271	5.17753	3.55139
H	-2.01268	4.04519	4.53259
H	-1.69659	2.88497	5.84934

```
H          -1.86391         2.30888         4.18558
C          -2.33978        -5.87219         2.96185
C           2.33978         5.87219         2.96185
H          -3.34749        -6.17299         3.28012
H          -1.66586        -6.73063         3.09102
H          -2.38776        -5.65422         1.88269
H           1.66586         6.73063         3.09102
H           2.38776         5.65422         1.88269
H           3.34749         6.17299         3.28012
```

7.8.17 1EMind

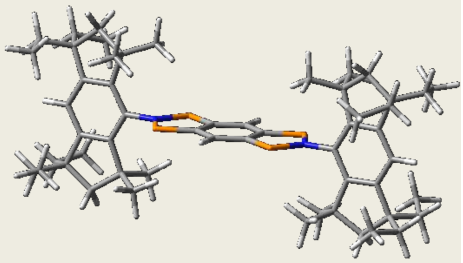

```
136
C54H76N2P4 @ PBE-D3/def2-SVP
C          -1.42980         0.00000        -0.00000
C          -0.72957         0.00017        -1.22363
H          -2.53235         0.00000        -0.00000
C          -0.72957        -0.00017         1.22363
P          -1.51218        -0.00139         2.81741
C           1.42980        -0.00000        -0.00000
P          -1.51218         0.00139        -2.81741
C           0.72957        -0.00017        -1.22363
C           0.72957         0.00017         1.22363
N           0.00000         0.00000         3.65904
P           1.51218         0.00139         2.81741
H           2.53235        -0.00000        -0.00000
N           0.00000        -0.00000        -3.65904
P           1.51218        -0.00139        -2.81741
C           0.00000         0.00000         5.11250
C           0.04397         1.22749         5.82280
C          -0.04397        -1.22749         5.82280
C          -0.01026         1.20522         7.23231
C           0.01026        -1.20522         7.23231
C           0.00000         0.00000         7.94207
H           0.00000         0.00000         9.04392
C           0.00000        -0.00000        -5.11250
C           0.04397        -1.22749        -5.82280
C          -0.04397         1.22749        -5.82280
C          -0.01026        -1.20522        -7.23231
C           0.01026         1.20522        -7.23231
C           0.00000        -0.00000        -7.94207
H           0.00000        -0.00000        -9.04392
C          -0.21169         2.67122        -5.31234
C           0.05946         3.52525        -6.60008
H           1.01190         4.07961        -6.48418
H          -0.72698         4.29610        -6.72467
C           0.13803         2.59066        -7.84295
C          -0.13803        -2.59066        -7.84295
C          -0.05946        -3.52525        -6.60008
H           0.72698        -4.29610        -6.72467
H          -1.01190        -4.07961        -6.48418
C           0.21169        -2.67122        -5.31234
C          -0.21169        -2.67122         5.31234
C           0.05946        -3.52525         6.60008
H           1.01190        -4.07961         6.48418
H          -0.72698        -4.29610         6.72467
```

C	0.13803	-2.59066	7.84295
C	-0.13803	2.59066	7.84295
C	-0.05946	3.52525	6.60008
H	0.72698	4.29610	6.72467
H	-1.01190	4.07961	6.48418
C	0.21169	2.67122	5.31234
C	-1.66401	-2.89760	4.83559
H	-1.88786	-2.30772	3.92391
H	-1.82030	-3.97033	4.59684
H	-2.39535	-2.60416	5.61244
C	0.75561	-3.08589	4.18887
H	0.68485	-4.18110	4.02386
H	0.51711	-2.59082	3.22787
H	1.80608	-2.84403	4.44693
C	1.66401	2.89760	4.83559
H	1.88786	2.30772	3.92391
H	1.82030	3.97033	4.59684
H	2.39535	2.60416	5.61244
C	-0.75561	3.08589	4.18887
H	-0.68485	4.18110	4.02386
H	-0.51711	2.59082	3.22787
H	-1.80608	2.84403	4.44693
C	0.96044	2.87328	8.90016
H	0.79987	3.90220	9.29158
H	0.78009	2.19776	9.76531
C	-1.51693	2.71693	8.55627
H	-1.48907	2.08740	9.47276
H	-1.61610	3.76613	8.91378
C	1.51693	-2.71693	8.55627
H	1.48907	-2.08740	9.47276
H	1.61610	-3.76613	8.91378
C	-0.96044	-2.87328	8.90016
H	-0.79987	-3.90220	9.29158
H	-0.78009	-2.19776	9.76531
C	0.75561	3.08589	-4.18887
H	0.51711	2.59082	-3.22787
H	1.80608	2.84403	-4.44693
H	0.68485	4.18110	-4.02386
C	-1.66401	2.89760	-4.83559
H	-2.39535	2.60416	-5.61244
H	-1.88786	2.30772	-3.92391
H	-1.82030	3.97033	-4.59684
C	1.66401	-2.89760	-4.83559
H	2.39535	-2.60416	-5.61244
H	1.88786	-2.30772	-3.92391
H	1.82030	-3.97033	-4.59684
C	-0.75561	-3.08589	-4.18887
H	-0.51711	-2.59082	-3.22787
H	-1.80608	-2.84403	-4.44693
H	-0.68485	-4.18110	-4.02386
C	-1.51693	-2.71693	-8.55627
H	-1.48907	-2.08740	-9.47276
H	-1.61610	-3.76613	-8.91378
C	0.96044	-2.87328	-8.90016
H	0.79987	-3.90220	-9.29158
H	0.78009	-2.19776	-9.76531
C	-0.96044	2.87328	-8.90016
H	-0.79987	3.90220	-9.29158
H	-0.78009	2.19776	-9.76531
C	1.51693	2.71693	-8.55627

```
H          1.48907        2.08740       -9.47276
H          1.61610        3.76613       -8.91378
C         -2.73885        2.33010        7.72378
H         -2.82396        2.93734        6.79922
H         -2.69940        1.26555        7.41536
H         -3.67266        2.48073        8.30161
C          2.73885       -2.33010        7.72378
H          2.82396       -2.93734        6.79922
H          2.69940       -1.26555        7.41536
H          3.67266       -2.48073        8.30161
C         -2.73885       -2.33010       -7.72378
H         -2.82396       -2.93734       -6.79922
H         -2.69940       -1.26555       -7.41536
H         -3.67266       -2.48073       -8.30161
C          2.73885        2.33010       -7.72378
H          2.82396        2.93734       -6.79922
H          2.69940        1.26555       -7.41536
H          3.67266        2.48073       -8.30161
C          2.40754        2.71263        8.43930
H          2.59338        1.69444        8.03898
H          2.67559        3.43869        7.64582
H          3.10965        2.87550        9.28148
C          2.40754       -2.71263       -8.43930
H          2.59338       -1.69444       -8.03898
H          2.67559       -3.43869       -7.64582
H          3.10965       -2.87550       -9.28148
C         -2.40754        2.71263       -8.43930
H         -2.59338        1.69444       -8.03898
H         -2.67559        3.43869       -7.64582
H         -3.10965        2.87550       -9.28148
C         -2.40754       -2.71263        8.43930
H         -2.59338       -1.69444        8.03898
H         -2.67559       -3.43869        7.64582
H         -3.10965       -2.87550        9.28148
```

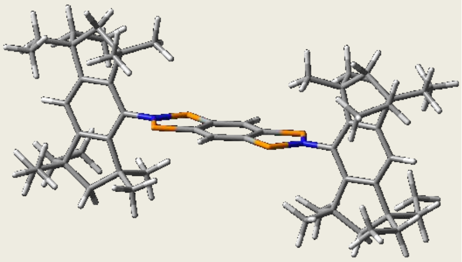

```
136
C54H76N2P4 @ PBE-D3/def2-TZVP
C         -1.42422        0.00000        0.00000
C         -0.72705        0.00081       -1.21688
H         -2.51766        0.00000        0.00000
C         -0.72705       -0.00081        1.21688
P         -1.49748       -0.00098        2.80360
C          1.42422       -0.00000       -0.00000
P         -1.49748        0.00098       -2.80360
C          0.72705       -0.00081       -1.21688
C          0.72705        0.00081        1.21688
N          0.00000       -0.00000        3.63605
P          1.49748        0.00098        2.80360
H          2.51766       -0.00000       -0.00000
N          0.00000       -0.00000       -3.63605
P          1.49748       -0.00098       -2.80360
C          0.00000       -0.00000        5.09059
C          0.04417        1.22246        5.79555
C         -0.04417       -1.22246        5.79555
C         -0.01034        1.19900        7.19787
C          0.01034       -1.19900        7.19787
C          0.00000       -0.00000        7.90296
H          0.00000       -0.00000        8.99639
```

```
C 0.00000 -0.00000 -5.09059
C 0.04417 -1.22246 -5.79555
C -0.04417 1.22246 -5.79555
C -0.01034 -1.19900 -7.19787
C 0.01034 1.19900 -7.19787
C 0.00000 -0.00000 -7.90296
H 0.00000 -0.00000 -8.99639
C -0.19790 2.66499 -5.28738
C 0.05044 3.51505 -6.57791
H 0.98998 4.07577 -6.47348
H -0.74500 4.26432 -6.69709
C 0.12677 2.57800 -7.81210
C -0.12677 -2.57800 -7.81210
C -0.05044 -3.51505 -6.57791
H 0.74500 -4.26432 -6.69709
H -0.98998 -4.07577 -6.47348
C 0.19790 -2.66499 -5.28738
C -0.19790 -2.66499 5.28738
C 0.05044 -3.51505 6.57791
H 0.98998 -4.07577 6.47348
H -0.74500 -4.26432 6.69709
C 0.12677 -2.57800 7.81210
C -0.12677 2.57800 7.81210
C -0.05044 3.51505 6.57791
H 0.74500 4.26432 6.69709
H -0.98998 4.07577 6.47348
C 0.19790 2.66499 5.28738
C -1.63336 -2.90945 4.78135
H -1.83567 -2.34853 3.85829
H -1.77209 -3.98068 4.56791
H -2.37838 -2.60561 5.52807
C 0.79274 -3.08324 4.19068
H 0.72618 -4.17120 4.03626
H 0.57618 -2.59864 3.22986
H 1.82744 -2.83960 4.47037
C 1.63336 2.90945 4.78135
H 1.83567 2.34853 3.85829
H 1.77209 3.98068 4.56791
H 2.37838 2.60561 5.52807
C -0.79274 3.08324 4.19068
H -0.72618 4.17120 4.03626
H -0.57618 2.59864 3.22986
H -1.82744 2.83960 4.47037
C 0.97854 2.85147 8.85881
H 0.82391 3.86951 9.25547
H 0.81291 2.16975 9.71036
C -1.49265 2.71345 8.53898
H -1.46474 2.07647 9.43928
H -1.57662 3.75251 8.90151
C 1.49265 -2.71345 8.53898
H 1.46474 -2.07647 9.43928
H 1.57662 -3.75251 8.90151
C -0.97854 -2.85147 8.85881
H -0.82391 -3.86951 9.25547
H -0.81291 -2.16975 9.71036
C 0.79274 3.08324 -4.19068
H 0.57618 2.59864 -3.22986
H 1.82744 2.83960 -4.47037
H 0.72618 4.17120 -4.03626
C -1.63336 2.90945 -4.78135
```

H	-2.37838	2.60561	-5.52807
H	-1.83567	2.34853	-3.85829
H	-1.77209	3.98068	-4.56791
C	1.63336	-2.90945	-4.78135
H	2.37838	-2.60561	-5.52807
H	1.83567	-2.34853	-3.85829
H	1.77209	-3.98068	-4.56791
C	-0.79274	-3.08324	-4.19068
H	-0.57618	-2.59864	-3.22986
H	-1.82744	-2.83960	-4.47037
H	-0.72618	-4.17120	-4.03626
C	-1.49265	-2.71345	-8.53898
H	-1.46474	-2.07647	-9.43928
H	-1.57662	-3.75251	-8.90151
C	0.97854	-2.85147	-8.85881
H	0.82391	-3.86951	-9.25547
H	0.81291	-2.16975	-9.71036
C	-0.97854	2.85147	-8.85881
H	-0.82391	3.86951	-9.25547
H	-0.81291	2.16975	-9.71036
C	1.49265	2.71345	-8.53898
H	1.46474	2.07647	-9.43928
H	1.57662	3.75251	-8.90151
C	-2.72404	2.35055	7.71223
H	-2.81449	2.97377	6.81055
H	-2.69175	1.30117	7.38624
H	-3.64069	2.49307	8.30186
C	2.72404	-2.35055	7.71223
H	2.81449	-2.97377	6.81055
H	2.69175	-1.30117	7.38624
H	3.64069	-2.49307	8.30186
C	-2.72404	-2.35055	-7.71223
H	-2.81449	-2.97377	-6.81055
H	-2.69175	-1.30117	-7.38624
H	-3.64069	-2.49307	-8.30186
C	2.72404	2.35055	-7.71223
H	2.81449	2.97377	-6.81055
H	2.69175	1.30117	-7.38624
H	3.64069	2.49307	-8.30186
C	2.41796	2.69941	8.37566
H	2.59600	1.69533	7.96291
H	2.66924	3.43229	7.59615
H	3.12250	2.85062	9.20584
C	2.41796	-2.69941	-8.37566
H	2.59600	-1.69533	-7.96291
H	2.66924	-3.43229	-7.59615
H	3.12250	-2.85062	-9.20584
C	-2.41796	2.69941	-8.37566
H	-2.59600	1.69533	-7.96291
H	-2.66924	3.43229	-7.59615
H	-3.12250	2.85062	-9.20584
C	-2.41796	-2.69941	8.37566
H	-2.59600	-1.69533	7.96291
H	-2.66924	-3.43229	7.59615
H	-3.12250	-2.85062	9.20584

7.8.18 1Mes*

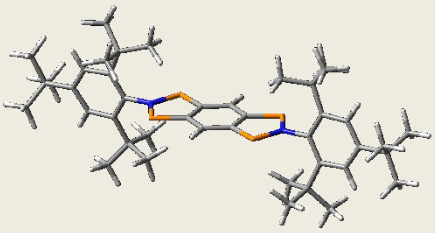

```
108
C42H60N2P4 @ PBE-D3/def2-SVP
C          0.00000          0.00000         -1.43840
C          0.00601          1.22334         -0.73792
H          0.00000          0.00000         -2.54089
C         -0.00601         -1.22334         -0.73792
P         -0.01896         -2.81846         -1.51868
C          0.00000          0.00000          1.42145
P          0.01896          2.81846         -1.51868
C          0.00668          1.22339          0.72101
C         -0.00668         -1.22339          0.72101
N         -0.01862         -3.65788         -0.00895
P         -0.01099         -2.81882          1.50140
H          0.00000          0.00000          2.52394
N          0.01862          3.65788         -0.00895
P          0.01099          2.81882          1.50140
C         -0.02551         -5.10940         -0.00560
C          0.02551          5.10940         -0.00560
C          1.15434         -7.21612         -0.12679
C         -1.21997         -7.20916          0.15117
C          1.21997          7.20916          0.15117
C         -1.15434          7.21612         -0.12679
H          2.08647         -7.78000         -0.24037
H         -2.15760         -7.75708          0.27406
H          2.15760          7.75708          0.27406
H         -2.08647          7.78000         -0.24037
C         -1.21673          5.81092         -0.14139
C          1.21673         -5.81092         -0.14139
C          1.26873          5.79854          0.13891
C         -1.26873         -5.79854          0.13891
C          0.03658          7.94139          0.01953
C         -0.03658         -7.94139          0.01953
C         -2.62374          5.18325         -0.40034
C          2.62374         -5.18325         -0.40034
C          2.67347          5.15987          0.38317
C         -2.67347         -5.15987          0.38317
C         -3.74627          6.13843          0.08070
H         -4.72317          5.62691         -0.02484
H         -3.81431          7.06896         -0.51519
H         -3.61999          6.41537          1.14684
C          3.74627         -6.13843          0.08070
H          4.72317         -5.62691         -0.02484
H          3.81431         -7.06896         -0.51519
H          3.61999         -6.41537          1.14684
C          3.79749          6.10598         -0.11344
H          4.77158          5.58660         -0.02077
H          3.88158          7.03675          0.47984
H          3.65884          6.38277         -1.17805
C         -3.79749         -6.10598         -0.11344
H         -4.77158         -5.58660         -0.02077
H         -3.88158         -7.03675          0.47984
H         -3.65884         -6.38277         -1.17805
C          2.85270          4.97810          1.90956
H          3.87073          4.59621          2.13351
H          2.11768          4.25580          2.31808
H          2.71739          5.94109          2.44189
C         -2.78891          5.00026         -1.92813
```

```
H           -3.80720         4.62480        -2.16153
H           -2.05529         4.27250        -2.32929
H           -2.64190         5.96183        -2.45998
C            2.78891        -5.00026        -1.92813
H            3.80720        -4.62480        -2.16153
H            2.05529        -4.27250        -2.32929
H            2.64190        -5.96183        -2.45998
C           -2.85270        -4.97810         1.90956
H           -3.87073        -4.59621         2.13351
H           -2.11768        -4.25580         2.31808
H           -2.71739        -5.94109         2.44189
C            2.94038         3.81042        -0.32317
H            2.73757         3.87047        -1.40992
H            2.36128         2.97083         0.10100
H            4.01129         3.55249        -0.19605
C           -2.90640         3.83750         0.30732
H           -2.71033         3.89871         1.39529
H           -2.33052         2.99261        -0.11059
H           -3.97830         3.58717         0.17343
C            2.90640        -3.83750         0.30732
H            2.71033        -3.89871         1.39529
H            2.33052        -2.99261        -0.11059
H            3.97830        -3.58717         0.17343
C           -2.94038        -3.81042        -0.32317
H           -2.73757        -3.87047        -1.40992
H           -2.36128        -2.97083         0.10100
H           -4.01129        -3.55249        -0.19605
C            0.00000        -9.47970         0.02813
C           -0.00000         9.47970         0.02813
C           -0.59070         9.97942        -1.31009
H           -0.63265        11.08826        -1.32583
H           -1.61989         9.60184        -1.47224
H            0.02982         9.64496        -2.16591
C            0.59070        -9.97942        -1.31009
H            0.63265       -11.08826        -1.32583
H            1.61989        -9.60184        -1.47224
H           -0.02982        -9.64496        -2.16591
C           -0.89056         9.95895         1.19701
H           -0.48754         9.61138         2.16971
H           -1.92759         9.57882         1.10727
H           -0.93856        11.06744         1.21903
C            0.89056        -9.95895         1.19701
H            0.48754        -9.61138         2.16971
H            1.92759        -9.57882         1.10727
H            0.93856       -11.06744         1.21903
C            1.40017        10.09346         0.20006
H            2.08231         9.80343        -0.62442
H            1.86856         9.79075         1.15834
H            1.32804        11.19964         0.19966
C           -1.40017       -10.09346         0.20006
H           -2.08231        -9.80343        -0.62442
H           -1.86856        -9.79075         1.15834
H           -1.32804       -11.19964         0.19966
```

```
108
C42H60N2P4 @ PBE-D3/def2-TZVP
C            0.00000         0.00000        -1.44298
C            0.00624         1.21653        -0.74556
```

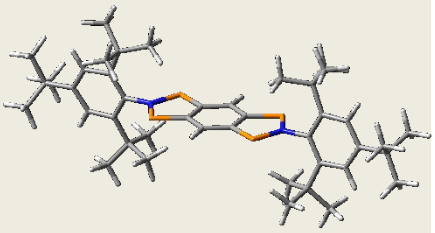

H	0.00000	0.00000	-2.53639
C	-0.00624	-1.21653	-0.74556
P	-0.02062	-2.80503	-1.51386
C	0.00000	0.00000	1.40549
P	0.02062	2.80503	-1.51386
C	0.00663	1.21663	0.70815
C	-0.00663	-1.21663	0.70815
N	-0.01859	-3.63399	-0.01964
P	-0.01026	-2.80566	1.47572
H	0.00000	0.00000	2.49889
N	0.01859	3.63399	-0.01964
P	0.01026	2.80566	1.47572
C	-0.02636	-5.08591	-0.01271
C	0.02636	5.08591	-0.01271
C	1.14613	-7.18123	-0.12870
C	-1.21287	-7.17179	0.17281
C	1.21287	7.17179	0.17281
C	-1.14613	7.18123	-0.12870
H	2.06892	-7.74285	-0.24391
H	-2.14090	-7.71593	0.30890
H	2.14090	7.71593	0.30890
H	-2.06892	7.74285	-0.24391
C	-1.20868	5.78369	-0.15165
C	1.20868	-5.78369	-0.15165
C	1.26201	5.76806	0.14354
C	-1.26201	-5.76806	0.14354
C	0.03723	7.90015	0.03712
C	-0.03723	-7.90015	0.03712
C	-2.61336	5.16425	-0.41565
C	2.61336	-5.16425	-0.41565
C	2.66683	5.13662	0.37776
C	-2.66683	-5.13662	0.37776
C	-3.73157	6.11743	0.07061
H	-4.69756	5.60234	-0.02305
H	-3.80645	7.03517	-0.52762
H	-3.59611	6.39738	1.12530
C	3.73157	-6.11743	0.07061
H	4.69756	-5.60234	-0.02305
H	3.80645	-7.03517	-0.52762
H	3.59611	-6.39738	1.12530
C	3.77984	6.07968	-0.14137
H	4.74385	5.55662	-0.07433
H	3.88005	6.99881	0.45054
H	3.61603	6.35804	-1.19239
C	-3.77984	-6.07968	-0.14137
H	-4.74385	-5.55662	-0.07433
H	-3.88005	-6.99881	0.45054
H	-3.61603	-6.35804	-1.19239
C	2.86828	4.97087	1.89952
H	3.88710	4.60798	2.10591
H	2.15475	4.24811	2.31962
H	2.72982	5.92990	2.41921
C	-2.78428	4.99379	-1.94061
H	-3.80123	4.63725	-2.16642
H	-2.06835	4.26471	-2.34499
H	-2.62860	5.95038	-2.45998
C	2.78428	-4.99379	-1.94061
H	3.80123	-4.63725	-2.16642
H	2.06835	-4.26471	-2.34499
H	2.62860	-5.95038	-2.45998

```
C          -2.86828        -4.97087         1.89952
H          -3.88710        -4.60798         2.10591
H          -2.15475        -4.24811         2.31962
H          -2.72982        -5.92990         2.41921
C           2.93726         3.78434        -0.31318
H           2.74397         3.83651        -1.39271
H           2.35991         2.95613         0.11211
H           3.99959         3.53415        -0.17638
C          -2.90583         3.81782         0.27861
H          -2.72549         3.87425         1.36025
H          -2.32963         2.98265        -0.13415
H          -3.96832         3.57554         0.12930
C           2.90583        -3.81782         0.27861
H           2.72549        -3.87425         1.36025
H           2.32963        -2.98265        -0.13415
H           3.96832        -3.57554         0.12930
C          -2.93726        -3.78434        -0.31318
H          -2.74397        -3.83651        -1.39271
H          -2.35991        -2.95613         0.11211
H          -3.99959        -3.53415        -0.17638
C           0.00000        -9.43248         0.06161
C          -0.00000         9.43248         0.06161
C          -0.57543         9.94693        -1.27376
H          -0.61697        11.04671        -1.27252
H          -1.59329         9.57130        -1.44799
H           0.05145         9.62640        -2.11841
C           0.57543        -9.94693        -1.27376
H           0.61697       -11.04671        -1.27252
H           1.59329        -9.57130        -1.44799
H          -0.05145        -9.62640        -2.11841
C          -0.90312         9.90011         1.22100
H          -0.51291         9.54721         2.18661
H          -1.92897         9.52130         1.11389
H          -0.94949        10.99935         1.24852
C           0.90312        -9.90011         1.22100
H           0.51291        -9.54721         2.18661
H           1.92897        -9.52130         1.11389
H           0.94949       -10.99935         1.24852
C           1.39260        10.04758         0.25569
H           2.07850         9.77057        -0.55829
H           1.84523         9.73934         1.20963
H           1.31203        11.14413         0.26477
C          -1.39260       -10.04758         0.25569
H          -2.07850        -9.77057        -0.55829
H          -1.84523        -9.73934         1.20963
H          -1.31203       -11.14413         0.26477
```

7.8.19 10ma

```
124
C50H68N2P4 @ PBE-D3/def2-SVP
C          -1.43015         0.00000        -0.00000
C          -0.72940         0.00104         1.22326
H          -2.53263         0.00000        -0.00000
C          -0.72940        -0.00104        -1.22326
P          -1.50873         0.00134        -2.81820
C           1.43015        -0.00000        -0.00000
P          -1.50873        -0.00134         2.81820
```

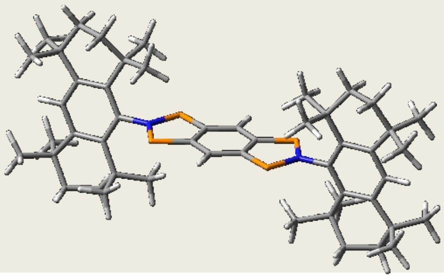

C	0.72940	-0.00104	1.22326
C	0.72940	0.00104	-1.22326
N	-0.00000	0.00000	-3.66149
P	1.50873	-0.00134	-2.81820
H	2.53263	-0.00000	-0.00000
N	-0.00000	0.00000	3.66149
P	1.50873	0.00134	2.81820
C	-0.00000	0.00000	-5.11669
C	-0.10468	-1.26657	-5.78237
C	0.10468	1.26657	-5.78237
C	-0.09056	-1.23878	-7.20657
C	0.09056	1.23878	-7.20657
C	-0.00000	0.00000	-7.84852
H	-0.00000	0.00000	-8.94586
C	-0.00000	0.00000	5.11669
C	-0.10468	1.26657	5.78237
C	0.10468	-1.26657	5.78237
C	-0.09056	1.23878	7.20657
C	0.09056	-1.23878	7.20657
C	-0.00000	0.00000	7.84852
H	-0.00000	0.00000	8.94586
C	0.34123	-2.63575	5.07319
C	-0.17262	-3.77335	5.99327
H	0.04161	-4.73934	5.49043
H	-1.27998	-3.70149	6.06337
C	0.44551	-3.75803	7.37552
H	1.54812	-3.85180	7.29005
H	0.11128	-4.63496	7.96927
C	0.09428	-2.47058	8.13910
C	-0.09428	2.47058	8.13910
C	-0.44551	3.75803	7.37552
H	-0.11128	4.63496	7.96927
H	-1.54812	3.85180	7.29005
C	0.17262	3.77335	5.99327
H	1.27998	3.70149	6.06337
H	-0.04161	4.73934	5.49043
C	-0.34123	2.63575	5.07319
C	0.34123	2.63575	-5.07319
C	-0.17262	3.77335	-5.99327
H	0.04161	4.73934	-5.49043
H	-1.27998	3.70149	-6.06337
C	0.44551	3.75803	-7.37552
H	1.54812	3.85180	-7.29005
H	0.11128	4.63496	-7.96927
C	0.09428	2.47058	-8.13910
C	-0.09428	-2.47058	-8.13910
C	-0.44551	-3.75803	-7.37552
H	-0.11128	-4.63496	-7.96927
H	-1.54812	-3.85180	-7.29005
C	0.17262	-3.77335	-5.99327
H	1.27998	-3.70149	-6.06337
H	-0.04161	-4.73934	-5.49043
C	-0.34123	-2.63575	-5.07319
C	-0.37300	2.88582	-3.72586
H	0.05983	2.32479	-2.87861
H	-0.26791	3.96197	-3.47743
H	-1.45568	2.66515	-3.78934
C	1.86486	2.80996	-4.85919
H	2.08580	3.83865	-4.50402
H	2.23820	2.09760	-4.09663

H	2.44117	2.62853	-5.78676
C	0.37300	-2.88582	-3.72586
H	-0.05983	-2.32479	-2.87861
H	0.26791	-3.96197	-3.47743
H	1.45568	-2.66515	-3.78934
C	-1.86486	-2.80996	-4.85919
H	-2.08580	-3.83865	-4.50402
H	-2.23820	-2.09760	-4.09663
H	-2.44117	-2.62853	-5.78676
C	1.32292	-2.60921	-8.74643
H	1.35400	-3.45662	-9.46261
H	1.62110	-1.68942	-9.28773
H	2.08363	-2.79128	-7.96138
C	-1.11667	-2.30877	-9.28462
H	-0.85129	-1.49812	-9.99146
H	-1.16764	-3.24878	-9.87198
H	-2.12996	-2.09798	-8.88685
C	1.11667	2.30877	-9.28462
H	0.85129	1.49812	-9.99146
H	1.16764	3.24878	-9.87198
H	2.12996	2.09798	-8.88685
C	-1.32292	2.60921	-8.74643
H	-1.35400	3.45662	-9.46261
H	-1.62110	1.68942	-9.28773
H	-2.08363	2.79128	-7.96138
C	1.86486	-2.80996	4.85919
H	2.23820	-2.09760	4.09663
H	2.44117	-2.62853	5.78676
H	2.08580	-3.83865	4.50402
C	-0.37300	-2.88582	3.72586
H	-1.45568	-2.66515	3.78934
H	0.05983	-2.32479	2.87861
H	-0.26791	-3.96197	3.47743
C	0.37300	2.88582	3.72586
H	1.45568	2.66515	3.78934
H	-0.05983	2.32479	2.87861
H	0.26791	3.96197	3.47743
C	-1.86486	2.80996	4.85919
H	-2.23820	2.09760	4.09663
H	-2.44117	2.62853	5.78676
H	-2.08580	3.83865	4.50402
C	-1.11667	2.30877	9.28462
H	-0.85129	1.49812	9.99146
H	-1.16764	3.24878	9.87198
H	-2.12996	2.09798	8.88685
C	1.32292	2.60921	8.74643
H	1.35400	3.45662	9.46261
H	1.62110	1.68942	9.28773
H	2.08363	2.79128	7.96138
C	-1.32292	-2.60921	8.74643
H	-1.35400	-3.45662	9.46261
H	-1.62110	-1.68942	9.28773
H	-2.08363	-2.79128	7.96138
C	1.11667	-2.30877	9.28462
H	0.85129	-1.49812	9.99146
H	1.16764	-3.24878	9.87198
H	2.12996	-2.09798	8.88685

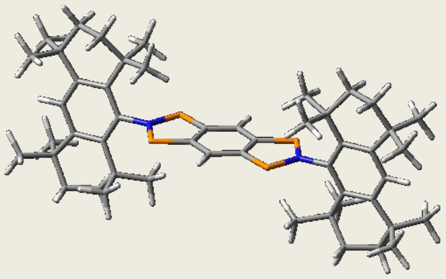

```
124
C50H68N2P4 @ PBE-D3/def2-TZVP
C          -1.42443         0.00000         0.00000
C          -0.72677         0.00061         1.21654
H          -2.51786         0.00000         0.00000
C          -0.72677        -0.00061        -1.21654
P          -1.49349         0.00424        -2.80474
C           1.42443        -0.00000         0.00000
P          -1.49349        -0.00424         2.80474
C           0.72677        -0.00061         1.21654
C           0.72677         0.00061        -1.21654
N          -0.00000        -0.00000        -3.63810
P           1.49349        -0.00424        -2.80474
H           2.51786        -0.00000         0.00000
N          -0.00000        -0.00000         3.63810
P           1.49349         0.00424         2.80474
C          -0.00000        -0.00000        -5.09406
C          -0.13167        -1.25751        -5.75541
C           0.13167         1.25751        -5.75541
C          -0.12412        -1.22893        -7.17194
C           0.12412         1.22893        -7.17194
C          -0.00000        -0.00000        -7.81018
H          -0.00000        -0.00000        -8.89932
C          -0.00000        -0.00000         5.09406
C          -0.13167         1.25751         5.75541
C           0.13167        -1.25751         5.75541
C          -0.12412         1.22893         7.17194
C           0.12412        -1.22893         7.17194
C          -0.00000        -0.00000         7.81018
H          -0.00000        -0.00000         8.89932
C           0.36540        -2.62448         5.05222
C          -0.13732        -3.75462         5.98104
H           0.06169        -4.71379         5.47804
H          -1.23351        -3.67405         6.07529
C           0.51345        -3.73981         7.34515
H           1.60431        -3.83999         7.23441
H           0.18698        -4.60404         7.94571
C           0.18581        -2.45043         8.10727
C          -0.18581         2.45043         8.10727
C          -0.51345         3.73981         7.34515
H          -0.18698         4.60404         7.94571
H          -1.60431         3.83999         7.23441
C           0.13732         3.75462         5.98104
H           1.23351         3.67405         6.07529
H          -0.06169         4.71379         5.47804
C          -0.36540         2.62448         5.05222
C           0.36540         2.62448        -5.05222
C          -0.13732         3.75462        -5.98104
H           0.06169         4.71379        -5.47804
H          -1.23351         3.67405        -6.07529
C           0.51345         3.73981        -7.34515
H           1.60431         3.83999        -7.23441
H           0.18698         4.60404        -7.94571
C           0.18581         2.45043        -8.10727
C          -0.18581        -2.45043        -8.10727
C          -0.51345        -3.73981        -7.34515
H          -0.18698        -4.60404        -7.94571
H          -1.60431        -3.83999        -7.23441
C           0.13732        -3.75462        -5.98104
H           1.23351        -3.67405        -6.07529
```

H	-0.06169	-4.71379	-5.47804
C	-0.36540	-2.62448	-5.05222
C	-0.36261	2.88407	-3.71833
H	0.05212	2.32514	-2.87239
H	-0.25174	3.95237	-3.47683
H	-1.43701	2.67285	-3.79640
C	1.88170	2.80884	-4.81975
H	2.08889	3.84355	-4.50368
H	2.23849	2.13273	-4.03056
H	2.46794	2.59619	-5.72313
C	0.36261	-2.88407	-3.71833
H	-0.05212	-2.32514	-2.87239
H	0.25174	-3.95237	-3.47683
H	1.43701	-2.67285	-3.79640
C	-1.88170	-2.80884	-4.81975
H	-2.08889	-3.84355	-4.50368
H	-2.23849	-2.13273	-4.03056
H	-2.46794	-2.59619	-5.72313
C	1.19369	-2.60532	-8.78459
H	1.16890	-3.43691	-9.50551
H	1.47960	-1.69150	-9.32356
H	1.97995	-2.81256	-8.04491
C	-1.25882	-2.26429	-9.19723
H	-1.02106	-1.45235	-9.89788
H	-1.34557	-3.19056	-9.78571
H	-2.23973	-2.05145	-8.74822
C	1.25882	2.26429	-9.19723
H	1.02106	1.45235	-9.89788
H	1.34557	3.19056	-9.78571
H	2.23973	2.05145	-8.74822
C	-1.19369	2.60532	-8.78459
H	-1.16890	3.43691	-9.50551
H	-1.47960	1.69150	-9.32356
H	-1.97995	2.81256	-8.04491
C	1.88170	-2.80884	4.81975
H	2.23849	-2.13273	4.03056
H	2.46794	-2.59619	5.72313
H	2.08889	-3.84355	4.50368
C	-0.36261	-2.88407	3.71833
H	-1.43701	-2.67285	3.79640
H	0.05212	-2.32514	2.87239
H	-0.25174	-3.95237	3.47683
C	0.36261	2.88407	3.71833
H	1.43701	2.67285	3.79640
H	-0.05212	2.32514	2.87239
H	0.25174	3.95237	3.47683
C	-1.88170	2.80884	4.81975
H	-2.23849	2.13273	4.03056
H	-2.46794	2.59619	5.72313
H	-2.08889	3.84355	4.50368
C	-1.25882	2.26429	9.19723
H	-1.02106	1.45235	9.89788
H	-1.34557	3.19056	9.78571
H	-2.23973	2.05145	8.74822
C	1.19369	2.60532	8.78459
H	1.16890	3.43691	9.50551
H	1.47960	1.69150	9.32356
H	1.97995	2.81256	8.04491
C	-1.19369	-2.60532	8.78459
H	-1.16890	-3.43691	9.50551

```
H          -1.47960        -1.69150         9.32356
H          -1.97995        -2.81256         8.04491
C           1.25882        -2.26429         9.19723
H           1.02106        -1.45235         9.89788
H           1.34557        -3.19056         9.78571
H           2.23973        -2.05145         8.74822
```

7.8.20 4Ter

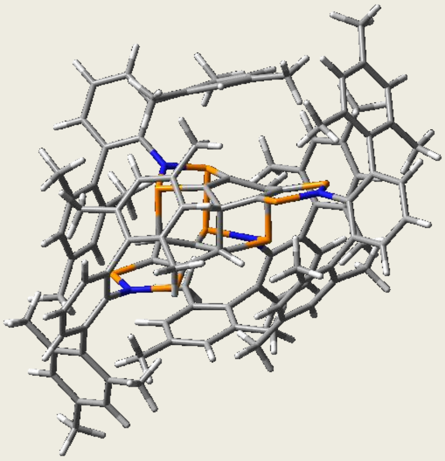

```
224
C108H104N4P8 @ PBE-D3/def2-SVP
C           0.39765        -2.18206        -1.11047
C           0.67726        -2.64448         0.28851
H           0.51105        -2.97540        -1.87298
C           1.09195        -0.90242        -1.44469
P           1.15043        -0.33511        -3.17190
C           0.78743        -0.20328         0.92119
P           0.58521        -4.29978         0.81713
C           0.90538        -1.66041         1.28596
C           1.30391         0.05303        -0.47882
N           1.82695         1.23217        -2.74089
P           1.55046         1.77024        -1.09212
H           1.27539         0.44094         1.67942
N           0.79663        -3.87372         2.49595
P           1.06155        -2.20835         2.92106
C           2.87187         1.79951        -3.53548
C           0.48132        -4.80845         3.53703
C           2.74027         3.12315        -4.02867
C           4.04917         1.04361        -3.80503
C           1.29394        -5.94973         3.74405
C          -0.68151        -4.57696         4.32863
C           3.75781         3.65007        -4.84802
C           1.55105         3.94789        -3.65613
C           5.03521         1.59825        -4.64243
C           4.26208        -0.26367        -3.10857
C           0.95785        -6.83244         4.79044
C           2.47045        -6.23434         2.86626
C          -0.97359        -5.48101         5.36709
C          -1.59884        -3.43054         4.02392
H           3.64498         4.67341        -5.23812
C           4.88833         2.88815        -5.17324
C           0.32251         3.77558        -4.34065
C           1.65795         4.90097        -2.60891
H           5.94469         1.01286        -4.84805
C           4.06712        -1.50385        -3.77009
C           4.64775        -0.24356        -1.74113
H           1.59508        -7.71385         4.95976
C          -0.15749        -6.59608         5.60468
C           3.70257        -5.56757         3.08325
C           2.34582        -7.18233         1.81805
H          -1.87293        -5.30999         5.97800
C          -1.57643        -2.25894         4.82936
C          -2.47045        -3.50004         2.90514
H           5.66884         3.30928        -5.82474
C          -0.79343         4.53684        -3.94092
C           0.22182         2.80277        -5.48708
```

C	0.51795	5.63909	-2.24235
C	2.96697	5.11651	-1.89092
C	4.15621	-2.69367	-3.02293
C	3.75466	-1.54866	-5.24468
C	4.72503	-1.45619	-1.03723
C	4.98002	1.05293	-1.04707
H	-0.40174	-7.29347	6.42014
C	4.79280	-5.86595	2.24655
C	3.84326	-4.54818	4.18532
C	3.46031	-7.43930	0.99882
C	1.03588	-7.88532	1.55932
C	-2.38409	-1.16402	4.46898
C	-0.68151	-2.16913	6.04048
C	-3.25412	-2.37734	2.57920
C	-2.61843	-4.74592	2.06521
H	-1.74906	4.39213	-4.46903
C	-0.72101	5.47011	-2.89032
H	-0.80766	2.75424	-5.88866
H	0.50952	1.77861	-5.17384
H	0.91132	3.08353	-6.31011
H	0.60045	6.36982	-1.42197
H	2.86410	5.87547	-1.09232
H	3.76477	5.44836	-2.58585
H	3.32483	4.17371	-1.42789
H	3.97987	-3.65386	-3.53385
C	4.44744	-2.69208	-1.64716
H	3.59381	-2.58878	-5.58884
H	4.57413	-1.11013	-5.85007
H	2.83920	-0.96438	-5.47345
H	5.00244	-1.43398	0.02919
H	5.42514	0.86718	-0.05060
H	4.07501	1.68198	-0.89308
H	5.68650	1.66516	-1.64275
H	5.75044	-5.34810	2.42041
C	4.69574	-6.79836	1.19825
H	4.89961	-4.24827	4.32526
H	3.45651	-4.93239	5.15080
H	3.25879	-3.63081	3.95468
H	3.35697	-8.16686	0.17716
H	1.11395	-8.57064	0.69350
H	0.22788	-7.15380	1.34440
H	0.70080	-8.47314	2.43771
H	-2.34414	-0.25014	5.08325
C	-3.22276	-1.19525	3.33911
H	0.37572	-2.37786	5.77427
H	-0.96313	-2.90985	6.81680
H	-0.72731	-1.16058	6.49496
H	-3.91144	-2.43233	1.69619
H	-3.63996	-5.16747	2.17961
H	-1.89797	-5.53701	2.34311
H	-2.47817	-4.51360	0.99131
C	-1.92150	6.27098	-2.45381
C	4.43671	-3.95845	-0.83457
C	5.88715	-7.12257	0.33234
C	-4.05536	-0.00307	2.93682
H	-2.16378	6.08036	-1.38706
H	-2.81804	6.02666	-3.05549
H	-1.73342	7.36100	-2.54915
H	5.17884	-3.92688	-0.01319
H	4.63903	-4.85454	-1.45216

H	3.44509	-4.10880	-0.35308
H	5.57786	-7.45248	-0.67925
H	6.49006	-7.94524	0.77419
H	6.56014	-6.24992	0.21887
H	-5.11548	-0.28821	2.77464
H	-3.68962	0.43648	1.98412
H	-4.02374	0.79782	3.70022
C	-0.39765	2.18206	-1.11047
C	-0.67726	2.64448	0.28851
H	-0.51105	2.97540	-1.87298
C	-1.09195	0.90242	-1.44469
P	-1.15043	0.33511	-3.17190
C	-0.78743	0.20328	0.92119
P	-0.58521	4.29978	0.81713
C	-0.90538	1.66041	1.28596
C	-1.30391	-0.05303	-0.47882
N	-1.82695	-1.23217	-2.74089
P	-1.55046	-1.77024	-1.09212
H	-1.27539	-0.44094	1.67942
N	-0.79663	3.87372	2.49595
P	-1.06155	2.20835	2.92106
C	-2.87187	-1.79951	-3.53548
C	-0.48132	4.80845	3.53703
C	-2.74027	-3.12315	-4.02867
C	-4.04917	-1.04361	-3.80503
C	-1.29394	5.94973	3.74405
C	0.68151	4.57696	4.32863
C	-3.75781	-3.65007	-4.84802
C	-1.55105	-3.94789	-3.65613
C	-5.03521	-1.59825	-4.64243
C	-4.26208	0.26367	-3.10857
C	-0.95785	6.83244	4.79044
C	-2.47045	6.23434	2.86626
C	0.97359	5.48101	5.36709
C	1.59884	3.43054	4.02392
H	-3.64498	-4.67341	-5.23812
C	-4.88833	-2.88815	-5.17324
C	-0.32251	-3.77558	-4.34065
C	-1.65795	-4.90097	-2.60891
H	-5.94469	-1.01286	-4.84805
C	-4.06712	1.50385	-3.77009
C	-4.64775	0.24356	-1.74113
H	-1.59508	7.71385	4.95976
C	0.15749	6.59608	5.60468
C	-3.70257	5.56757	3.08325
C	-2.34582	7.18233	1.81805
H	1.87293	5.30999	5.97800
C	1.57643	2.25894	4.82936
C	2.47045	3.50004	2.90514
H	-5.66884	-3.30928	-5.82474
C	0.79343	-4.53684	-3.94092
C	-0.22182	-2.80277	-5.48708
C	-0.51795	-5.63909	-2.24235
C	-2.96697	-5.11651	-1.89092
C	-4.15621	2.69367	-3.02293
C	-3.75466	1.54866	-5.24468
C	-4.72503	1.45619	-1.03723
C	-4.98002	-1.05293	-1.04707
H	0.40174	7.29347	6.42014
C	-4.79280	5.86595	2.24655

C	-3.84326	4.54818	4.18532
C	-3.46031	7.43930	0.99882
C	-1.03588	7.88532	1.55932
C	2.38409	1.16402	4.46898
C	0.68151	2.16913	6.04048
C	3.25412	2.37734	2.57920
C	2.61843	4.74592	2.06521
H	1.74906	-4.39213	-4.46903
C	0.72101	-5.47011	-2.89032
H	0.80766	-2.75424	-5.88866
H	-0.50952	-1.77861	-5.17384
H	-0.91132	-3.08353	-6.31011
H	-0.60045	-6.36982	-1.42197
H	-2.86410	-5.87547	-1.09232
H	-3.76477	-5.44836	-2.58585
H	-3.32483	-4.17371	-1.42789
H	-3.97987	3.65386	-3.53385
C	-4.44744	2.69208	-1.64716
H	-3.59381	2.58878	-5.58884
H	-4.57413	1.11013	-5.85007
H	-2.83920	0.96438	-5.47345
H	-5.00244	1.43398	0.02919
H	-5.42514	-0.86718	-0.05060
H	-4.07501	-1.68198	-0.89308
H	-5.68650	-1.66516	-1.64275
H	-5.75044	5.34810	2.42041
C	-4.69574	6.79836	1.19825
H	-4.89961	4.24827	4.32526
H	-3.45651	4.93239	5.15080
H	-3.25879	3.63081	3.95468
H	-3.35697	8.16686	0.17716
H	-1.11395	8.57064	0.69350
H	-0.22788	7.15380	1.34440
H	-0.70080	8.47314	2.43771
H	2.34414	0.25014	5.08325
C	3.22276	1.19525	3.33911
H	-0.37572	2.37786	5.77427
H	0.96313	2.90985	6.81680
H	0.72731	1.16058	6.49496
H	3.91144	2.43233	1.69619
H	3.63996	5.16747	2.17961
H	1.89797	5.53701	2.34311
H	2.47817	4.51360	0.99131
C	1.92150	-6.27098	-2.45381
C	-4.43671	3.95845	-0.83457
C	-5.88715	7.12257	0.33234
C	4.05536	0.00307	2.93682
H	2.16378	-6.08036	-1.38706
H	2.81804	-6.02666	-3.05549
H	1.73342	-7.36100	-2.54915
H	-5.17884	3.92688	-0.01319
H	-4.63903	4.85454	-1.45216
H	-3.44509	4.10880	-0.35308
H	-5.57786	7.45248	-0.67925
H	-6.49006	7.94524	0.77419
H	-6.56014	6.24992	0.21887
H	5.11548	0.28821	2.77464
H	3.68962	-0.43648	1.98412
H	4.02374	-0.79782	3.70022

```
224
C108H104N4P8 @ PBE-D3/def2-TZVP
C         -0.32613       -2.18063       -1.10258
C         -0.20389       -2.70927        0.29191
H         -0.46928       -2.96405       -1.85778
C          0.74148       -1.19498       -1.43370
P          0.98242       -0.67374       -3.14698
C          0.68145       -0.44251        0.92564
P         -0.79406       -4.24578        0.82021
C          0.33064       -1.85866        1.28473
C          1.24709       -0.36391       -0.47334
N          2.11540        0.57768       -2.72091
P          2.02363        1.16898       -1.09146
H          1.34521        0.00906        1.67695
N         -0.42320       -3.93553        2.47749
P          0.33656       -2.45139        2.90021
C          3.28165        0.79332       -3.52231
C         -0.99375       -4.74185        3.51942
C          3.55478        2.08164       -4.02745
C          4.16465       -0.28208       -3.78552
C         -0.54644       -6.06067        3.72495
C         -2.03122       -4.18708        4.30983
C          4.67882        2.26651       -4.84304
C          2.67550        3.23491       -3.68244
C          5.26547       -0.06218       -4.62209
C          3.97353       -1.59357       -3.09937
C         -1.12534       -6.80770        4.76107
C          0.50763       -6.68000        2.86989
C         -2.57244       -4.96569        5.33923
C         -2.57078       -2.82231        4.02358
H          4.88058        3.26384       -5.23888
C          5.51944        1.20023       -5.15823
C          1.47035        3.43950       -4.38276
C          3.05762        4.12438       -2.65459
H          5.94524       -0.89259       -4.82295
C          3.41390       -2.70300       -3.76730
C          4.35866       -1.70993       -1.74519
H         -0.77332       -7.82746        4.92767
C         -2.12091       -6.26476        5.57043
C          1.87014       -6.41798        3.12111
C          0.13535       -7.55773        1.83080
H         -3.37393       -4.54411        5.94834
C         -2.19382       -1.72142        4.82522
C         -3.44755       -2.62758        2.93421
H          6.38395        1.35698       -5.80523
C          0.64362        4.50896       -4.01431
C          1.08946        2.54234       -5.52885
C          2.20345        5.18071       -2.32074
C          4.35924        3.94878       -1.91849
C          3.15017       -3.86920       -3.03918
C          3.09521       -2.64136       -5.23690
C          4.07728       -2.89335       -1.05876
C          5.07575       -0.59056       -1.04021
H         -2.55517       -6.85927        6.37576
C          2.83753       -7.04396        2.32860
C          2.28873       -5.48168        4.22263
C          1.13717       -8.15176        1.05450
```

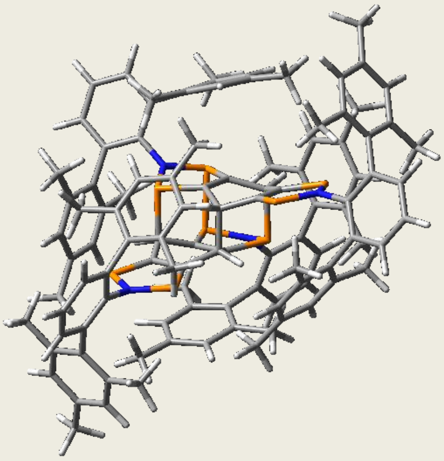

C	-1.31262	-7.85044	1.53755
C	-2.64834	-0.44188	4.48372
C	-1.30139	-1.90086	6.02459
C	-3.86830	-1.32975	2.62320
C	-3.99580	-3.77542	2.12682
H	-0.29758	4.65395	-4.55038
C	0.98664	5.38750	-2.98143
H	0.08883	2.78726	-5.90576
H	1.08914	1.48277	-5.23408
H	1.80863	2.63812	-6.35731
H	2.49407	5.85718	-1.51296
H	4.45339	4.69111	-1.11547
H	5.22195	4.05862	-2.59241
H	4.42938	2.94538	-1.47205
H	2.69099	-4.71735	-3.55375
C	3.43831	-3.97297	-1.67509
H	2.58166	-3.55560	-5.56329
H	4.00726	-2.53011	-5.84270
H	2.44834	-1.78146	-5.46648
H	4.35433	-2.96941	-0.00361
H	5.44442	-0.92366	-0.06109
H	4.41502	0.27638	-0.86903
H	5.92797	-0.22366	-1.63077
H	3.89331	-6.84117	2.53006
C	2.49408	-7.91565	1.29041
H	3.37260	-5.53302	4.38984
H	1.77825	-5.71466	5.16849
H	2.03497	-4.43803	3.97368
H	0.84450	-8.82177	0.24122
H	-1.40633	-8.51975	0.67234
H	-1.86802	-6.92582	1.31536
H	-1.81509	-8.32350	2.39418
H	-2.32993	0.40959	5.09080
C	-3.47209	-0.21984	3.37510
H	-0.37961	-2.44094	5.76110
H	-1.79769	-2.48446	6.81478
H	-1.01948	-0.92710	6.44637
H	-4.52437	-1.18552	1.76031
H	-5.02290	-4.01000	2.45226
H	-3.40232	-4.69063	2.23652
H	-4.03613	-3.51661	1.06072
C	0.08900	6.52760	-2.58259
C	3.05319	-5.18687	-0.87882
C	3.55478	-8.59819	0.46914
C	-3.91381	1.16664	2.98833
H	-0.15598	6.48366	-1.51025
H	-0.85228	6.51334	-3.14753
H	0.57744	7.49782	-2.76373
H	3.76275	-5.37499	-0.06187
H	2.99717	-6.08768	-1.50467
H	2.06381	-5.04895	-0.41141
H	3.16305	-8.91389	-0.50752
H	3.93115	-9.49887	0.98030
H	4.41656	-7.93846	0.29595
H	-5.00587	1.21578	2.86172
H	-3.46197	1.47304	2.03131
H	-3.61950	1.90744	3.74323
C	0.32613	2.18063	-1.10258
C	0.20389	2.70927	0.29191
H	0.46928	2.96405	-1.85778

C	-0.74148	1.19498	-1.43370
P	-0.98242	0.67374	-3.14698
C	-0.68145	0.44251	0.92564
P	0.79406	4.24578	0.82021
C	-0.33064	1.85866	1.28473
C	-1.24709	0.36391	-0.47334
N	-2.11540	-0.57768	-2.72091
P	-2.02363	-1.16898	-1.09146
H	-1.34521	-0.00906	1.67695
N	0.42320	3.93553	2.47749
P	-0.33656	2.45139	2.90021
C	-3.28165	-0.79332	-3.52231
C	0.99375	4.74185	3.51942
C	-3.55478	-2.08164	-4.02745
C	-4.16465	0.28208	-3.78552
C	0.54644	6.06067	3.72495
C	2.03122	4.18708	4.30983
C	-4.67882	-2.26651	-4.84304
C	-2.67550	-3.23491	-3.68244
C	-5.26547	0.06218	-4.62209
C	-3.97353	1.59357	-3.09937
C	1.12534	6.80770	4.76107
C	-0.50763	6.68000	2.86989
C	2.57244	4.96569	5.33923
C	2.57078	2.82231	4.02358
H	-4.88058	-3.26384	-5.23888
C	-5.51944	-1.20023	-5.15823
C	-1.47035	-3.43950	-4.38276
C	-3.05762	-4.12438	-2.65459
H	-5.94524	0.89259	-4.82295
C	-3.41390	2.70300	-3.76730
C	-4.35866	1.70993	-1.74519
H	0.77332	7.82746	4.92767
C	2.12091	6.26476	5.57043
C	-1.87014	6.41798	3.12111
C	-0.13535	7.55773	1.83080
H	3.37393	4.54411	5.94834
C	2.19382	1.72142	4.82522
C	3.44755	2.62758	2.93421
H	-6.38395	-1.35698	-5.80523
C	-0.64362	-4.50896	-4.01431
C	-1.08946	-2.54234	-5.52885
C	-2.20345	-5.18071	-2.32074
C	-4.35924	-3.94878	-1.91849
C	-3.15017	3.86920	-3.03918
C	-3.09521	2.64136	-5.23690
C	-4.07728	2.89335	-1.05876
C	-5.07575	0.59056	-1.04021
H	2.55517	6.85927	6.37576
C	-2.83753	7.04396	2.32860
C	-2.28873	5.48168	4.22263
C	-1.13717	8.15176	1.05450
C	1.31262	7.85044	1.53755
C	2.64834	0.44188	4.48372
C	1.30139	1.90086	6.02459
C	3.86830	1.32975	2.62320
C	3.99580	3.77542	2.12682
H	0.29758	-4.65395	-4.55038
C	-0.98664	-5.38750	-2.98143
H	-0.08883	-2.78726	-5.90576

```
H          -1.08914       -1.48277       -5.23408
H          -1.80863       -2.63812       -6.35731
H          -2.49407       -5.85718       -1.51296
H          -4.45339       -4.69111       -1.11547
H          -5.22195       -4.05862       -2.59241
H          -4.42938       -2.94538       -1.47205
H          -2.69099        4.71735       -3.55375
C          -3.43831        3.97297       -1.67509
H          -2.58166        3.55560       -5.56329
H          -4.00726        2.53011       -5.84270
H          -2.44834        1.78146       -5.46648
H          -4.35433        2.96941       -0.00361
H          -5.44442        0.92366       -0.06109
H          -4.41502       -0.27638       -0.86903
H          -5.92797        0.22366       -1.63077
H          -3.89331        6.84117        2.53006
C          -2.49408        7.91565        1.29041
H          -3.37260        5.53302        4.38984
H          -1.77825        5.71466        5.16849
H          -2.03497        4.43803        3.97368
H          -0.84450        8.82177        0.24122
H           1.40633        8.51975        0.67234
H           1.86802        6.92582        1.31536
H           1.81509        8.32350        2.39418
H           2.32993       -0.40959        5.09080
C           3.47209        0.21984        3.37510
H           0.37961        2.44094        5.76110
H           1.79769        2.48446        6.81478
H           1.01948        0.92710        6.44637
H           4.52437        1.18552        1.76031
H           5.02290        4.01000        2.45226
H           3.40232        4.69063        2.23652
H           4.03613        3.51661        1.06072
C          -0.08900       -6.52760       -2.58259
C          -3.05319        5.18687       -0.87882
C          -3.55478        8.59819        0.46914
C           3.91381       -1.16664        2.98833
H           0.15598       -6.48366       -1.51025
H           0.85228       -6.51334       -3.14753
H          -0.57744       -7.49782       -2.76373
H          -3.76275        5.37499       -0.06187
H          -2.99717        6.08768       -1.50467
H          -2.06381        5.04895       -0.41141
H          -3.16305        8.91389       -0.50752
H          -3.93115        9.49887        0.98030
H          -4.41656        7.93846        0.29595
H           5.00587       -1.21578        2.86172
H           3.46197       -1.47304        2.03131
H           3.61950       -1.90744        3.74323
```

7.8.21 4EMind

```
272
C108H152N4P8 @ PBE-D3/def2-SVP
P          -1.77773        1.58998        1.08456
N          -2.04490        1.03323        2.72753
C          -1.30402       -0.08221        0.48973
C           0.14596        2.18378        1.14819
```

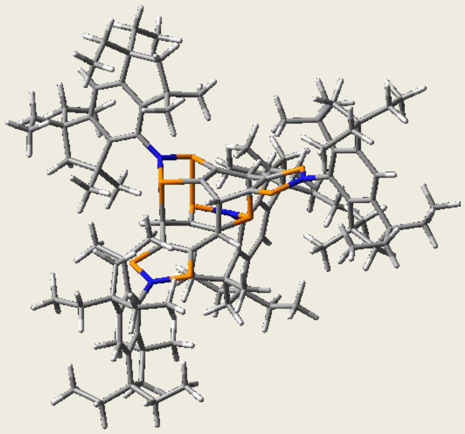

P	-1.15609	-0.40729	3.20421
C	-0.97404	-0.98293	1.47553
C	-0.75543	-0.28988	-0.90918
C	0.43408	2.69698	-0.23146
H	0.18464	2.97854	1.91567
C	0.97404	0.98293	1.47553
P	1.15609	0.40729	3.20421
C	-0.14596	-2.18378	1.14819
C	-0.75267	-1.76283	-1.24950
H	-1.31836	0.28932	-1.66964
C	0.75543	0.28988	-0.90918
P	0.37352	4.37692	-0.68886
C	0.75267	1.76283	-1.24950
C	1.30402	0.08221	0.48973
N	2.04490	-1.03323	2.72753
P	1.77773	-1.58998	1.08456
H	-0.18464	-2.97854	1.91567
C	-0.43408	-2.69698	-0.23146
P	-1.05377	-2.40016	-2.83761
H	1.31836	-0.28932	-1.66964
N	0.77355	4.04264	-2.34310
P	1.05377	2.40016	-2.83761
P	-0.37352	-4.37692	-0.68886
N	-0.77355	-4.04264	-2.34310
C	-0.87809	-5.07041	-3.35508
C	0.25481	-5.40820	-4.13603
C	-2.13308	-5.66115	-3.64309
C	0.09535	-6.25640	-5.25056
C	-2.25469	-6.50854	-4.76296
C	-1.15379	-6.79706	-5.57850
H	-1.27180	-7.42846	-6.47388
C	-3.44954	-5.60127	-2.85136
C	-4.45529	-6.34561	-3.79767
H	-5.22372	-5.63413	-4.15975
H	-5.00672	-7.12618	-3.23642
C	-3.68594	-6.95926	-5.00625
C	1.37326	-6.40974	-6.05944
C	2.41389	-5.60535	-5.22327
H	2.81060	-4.76186	-5.82240
H	3.29108	-6.23184	-4.96555
C	1.73571	-5.05528	-3.92009
C	-3.28489	-6.38447	-1.52920
H	-2.92173	-7.41398	-1.71266
H	-2.56148	-5.88624	-0.85231
H	-4.25864	-6.44761	-0.99980
C	-3.96653	-4.18572	-2.53150
H	-3.34577	-3.67491	-1.77007
H	-3.99337	-3.54778	-3.43744
H	-4.99821	-4.25137	-2.12723
C	-3.80187	-8.50375	-5.08461
H	-4.87223	-8.75846	-5.24995
H	-3.27250	-8.83767	-6.00415
C	-4.19899	-6.37910	-6.35644
H	-3.66480	-6.90082	-7.18081
H	-5.26896	-6.66613	-6.46247
C	1.76239	-7.89806	-6.25499
H	0.99304	-8.36702	-6.90730
H	2.70728	-7.93028	-6.84149
C	1.17985	-5.78448	-7.47189
H	2.15548	-5.84492	-8.00386

H	0.48407	-6.43614	-8.04496
C	2.26623	-5.81828	-2.68485
H	1.79003	-5.45682	-1.75091
H	2.06977	-6.90456	-2.76613
H	3.36166	-5.66804	-2.58637
C	2.04519	-3.55562	-3.74856
H	1.57978	-2.94832	-4.55046
H	1.69578	-3.16602	-2.77231
H	3.14294	-3.39786	-3.79122
C	3.15527	-1.48829	3.54490
C	4.41000	-0.80420	3.52784
C	3.00925	-2.60876	4.41065
C	5.43107	-1.20833	4.41625
C	4.04478	-2.96261	5.29878
C	5.24970	-2.25830	5.32051
H	6.05112	-2.53586	6.02379
C	-3.15527	1.48829	3.54490
C	-4.41000	0.80420	3.52784
C	-3.00925	2.60876	4.41065
C	-5.43107	1.20833	4.41625
C	-4.04478	2.96261	5.29878
C	-5.24970	2.25830	5.32051
H	-6.05112	2.53586	6.02379
C	0.87809	5.07041	-3.35508
C	-0.25481	5.40820	-4.13603
C	2.13308	5.66115	-3.64309
C	-0.09535	6.25640	-5.25056
C	2.25469	6.50854	-4.76296
C	1.15379	6.79706	-5.57850
H	1.27180	7.42846	-6.47388
C	3.44954	5.60127	-2.85136
C	4.45529	6.34561	-3.79767
H	5.22372	5.63413	-4.15975
H	5.00672	7.12618	-3.23642
C	3.68594	6.95926	-5.00625
C	-1.37326	6.40974	-6.05944
C	-2.41389	5.60535	-5.22327
H	-2.81060	4.76186	-5.82240
H	-3.29108	6.23184	-4.96555
C	-1.73571	5.05528	-3.92009
C	-1.86751	3.63338	4.49723
C	-2.27876	4.54349	5.70626
H	-2.26926	5.61018	5.40570
H	-1.53136	4.45320	6.51920
C	-3.68594	4.11842	6.21719
C	-6.70953	0.39955	4.29155
C	-6.39710	-0.55715	3.11088
H	-7.11708	-0.40478	2.28231
H	-6.51993	-1.61251	3.42453
C	-4.93284	-0.31561	2.59755
C	1.86751	-3.63338	4.49723
C	2.27876	-4.54349	5.70626
H	2.26926	-5.61018	5.40570
H	1.53136	-4.45320	6.51920
C	3.68594	-4.11842	6.21719
C	6.70953	-0.39955	4.29155
C	6.39710	0.55715	3.11088
H	7.11708	0.40478	2.28231
H	6.51993	1.61251	3.42453
C	4.93284	0.31561	2.59755

C	-0.49258	3.00895	4.76374
H	-0.16559	2.36315	3.93068
H	0.27801	3.79591	4.89780
H	-0.50385	2.37676	5.67396
C	-1.84306	4.47474	3.20177
H	-1.02104	5.22035	3.23229
H	-1.70779	3.84916	2.29854
H	-2.79841	5.01734	3.06729
C	-4.15520	-1.64375	2.66845
H	-3.18272	-1.58688	2.14794
H	-3.97635	-1.95849	3.71508
H	-4.73984	-2.43983	2.16170
C	-4.97335	0.16632	1.13208
H	-3.95737	0.31124	0.71702
H	-5.48091	-0.59055	0.49817
H	-5.52122	1.12453	1.03784
C	0.49258	-3.00895	4.76374
H	0.16559	-2.36315	3.93068
H	-0.27801	-3.79591	4.89780
H	0.50385	-2.37676	5.67396
C	1.84306	-4.47474	3.20177
H	1.02104	-5.22035	3.23229
H	1.70779	-3.84916	2.29854
H	2.79841	-5.01734	3.06729
C	4.97335	-0.16632	1.13208
H	3.95737	-0.31124	0.71702
H	5.48091	0.59055	0.49817
H	5.52122	-1.12453	1.03784
C	4.15520	1.64375	2.66845
H	4.73984	2.43983	2.16170
H	3.18272	1.58688	2.14794
H	3.97635	1.95849	3.71508
C	7.92832	-1.31727	4.00269
H	8.82108	-0.66605	3.87203
H	8.13113	-1.91972	4.91541
C	6.99801	0.37151	5.60994
H	7.26026	-0.37415	6.39261
H	7.91729	0.97928	5.45484
C	4.73086	-5.26252	6.13915
H	5.68063	-4.88731	6.57982
H	4.39398	-6.08003	6.81451
C	3.63621	-3.62734	7.69284
H	3.31095	-4.48376	8.32477
H	4.67640	-3.39613	8.01168
C	3.96653	4.18572	-2.53150
H	3.34577	3.67491	-1.77007
H	3.99337	3.54778	-3.43744
H	4.99821	4.25137	-2.12723
C	3.28489	6.38447	-1.52920
H	2.92173	7.41398	-1.71266
H	2.56148	5.88624	-0.85231
H	4.25864	6.44761	-0.99980
C	-2.04519	3.55562	-3.74856
H	-1.57978	2.94832	-4.55046
H	-1.69578	3.16602	-2.77231
H	-3.14294	3.39786	-3.79122
C	-2.26623	5.81828	-2.68485
H	-1.79003	5.45682	-1.75091
H	-2.06977	6.90456	-2.76613
H	-3.36166	5.66804	-2.58637

C	-1.76239	7.89806	-6.25499
H	-0.99304	8.36702	-6.90730
H	-2.70728	7.93028	-6.84149
C	-1.17985	5.78448	-7.47189
H	-2.15548	5.84492	-8.00386
H	-0.48407	6.43614	-8.04496
C	3.80187	8.50375	-5.08461
H	4.87223	8.75846	-5.24995
H	3.27250	8.83767	-6.00415
C	4.19899	6.37910	-6.35644
H	3.66480	6.90082	-7.18081
H	5.26896	6.66613	-6.46247
C	-4.73086	5.26252	6.13915
H	-5.68063	4.88731	6.57982
H	-4.39398	6.08003	6.81451
C	-3.63621	3.62734	7.69284
H	-3.31095	4.48376	8.32477
H	-4.67640	3.39613	8.01168
C	-6.99801	-0.37151	5.60994
H	-7.26026	0.37415	6.39261
H	-7.91729	-0.97928	5.45484
C	-7.92832	1.31727	4.00269
H	-8.82108	0.66605	3.87203
H	-8.13113	1.91972	4.91541
C	0.65651	-4.34891	-7.50159
H	-0.34586	-4.27266	-7.03301
H	1.32605	-3.64870	-6.96122
H	0.57134	-3.98178	-8.54391
C	-0.65651	4.34891	-7.50159
H	0.34586	4.27266	-7.03301
H	-1.32605	3.64870	-6.96122
H	-0.57134	3.98178	-8.54391
C	1.90993	-8.73189	-4.98384
H	2.73296	-8.36510	-4.33799
H	0.97857	-8.71617	-4.38089
H	2.13416	-9.78938	-5.22920
C	-1.90993	8.73189	-4.98384
H	-2.73296	8.36510	-4.33799
H	-0.97857	8.71617	-4.38089
H	-2.13416	9.78938	-5.22920
C	-4.04237	-4.86971	-6.53776
H	-4.57829	-4.29709	-5.75325
H	-2.97683	-4.56456	-6.49736
H	-4.45016	-4.54559	-7.51609
C	4.04237	4.86971	-6.53776
H	4.57829	4.29709	-5.75325
H	2.97683	4.56456	-6.49736
H	4.45016	4.54559	-7.51609
C	-3.25955	-9.28424	-3.88890
H	-2.19096	-9.04895	-3.70472
H	-3.81698	-9.05988	-2.95717
H	-3.33873	-10.37635	-4.06150
C	3.25955	9.28424	-3.88890
H	2.19096	9.04895	-3.70472
H	3.81698	9.05988	-2.95717
H	3.33873	10.37635	-4.06150
C	-2.75141	2.41147	7.96434
H	-3.08242	1.53040	7.37754
H	-1.68988	2.60138	7.70452
H	-2.78036	2.13437	9.03716

C	2.75141	-2.41147	7.96434
H	3.08242	-1.53040	7.37754
H	1.68988	-2.60138	7.70452
H	2.78036	-2.13437	9.03716
C	-5.01773	5.82181	4.74689
H	-4.12349	6.29812	4.29632
H	-5.35709	5.02450	4.05381
H	-5.81199	6.59396	4.78706
C	5.01773	-5.82181	4.74689
H	4.12349	-6.29812	4.29632
H	5.35709	-5.02450	4.05381
H	5.81199	-6.59396	4.78706
C	-7.78838	2.25745	2.80632
H	-6.91944	2.93668	2.92643
H	-7.64720	1.70369	1.85589
H	-8.69430	2.88512	2.68788
C	7.78838	-2.25745	2.80632
H	6.91944	-2.93668	2.92643
H	7.64720	-1.70369	1.85589
H	8.69430	-2.88512	2.68788
C	-5.86613	-1.25864	6.12599
H	-5.60544	-2.06130	5.40654
H	-4.94437	-0.67051	6.31320
H	-6.15230	-1.75143	7.07675
C	5.86613	1.25864	6.12599
H	5.60544	2.06130	5.40654
H	4.94437	0.67051	6.31320
H	6.15230	1.75143	7.07675

272

C108H152N4P8 @ PBE-D3/def2-TZVP

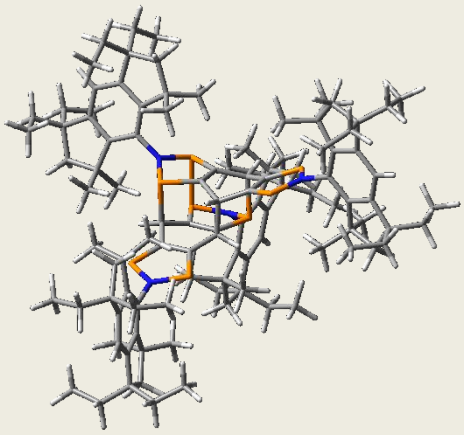

P	0.86932	-2.20443	-1.07922
N	0.26294	-2.26091	-2.70480
C	-0.52248	-1.19117	-0.47758
C	2.08567	-0.62103	-1.12819
P	-0.77043	-0.93878	-3.16948
C	-1.24937	-0.57063	-1.45525
C	-0.52802	-0.61144	0.92022
C	2.66555	-0.52176	0.24821
H	2.84393	-0.85230	-1.88590
C	1.24937	0.57063	-1.45525
P	0.77043	0.93878	-3.16948
C	-2.08567	0.62103	-1.12819
C	-1.90703	-0.10722	1.25793
H	-0.17747	-1.33414	1.67256
C	0.52802	0.61144	0.92022
P	4.21756	-1.13305	0.70746
C	1.90703	0.10722	1.25793
C	0.52248	1.19117	-0.47758
N	-0.26294	2.26091	-2.70480
P	-0.86932	2.20443	-1.07922
H	-2.84393	0.85230	-1.88590
C	-2.66555	0.52176	0.24821
P	-2.62558	-0.20263	2.82377
H	0.17747	1.33414	1.67256
N	4.05884	-0.61735	2.33746
P	2.62558	0.20263	2.82377
P	-4.21756	1.13305	0.70746

N	-4.05884	0.61735	2.33746
C	-5.07663	0.83851	3.34254
C	-5.02542	1.99318	4.15010
C	-6.05680	-0.14564	3.58479
C	-5.88935	2.09642	5.25005
C	-6.90578	-0.00529	4.69227
C	-6.81746	1.09882	5.53587
H	-7.46089	1.17627	6.41666
C	-6.42650	-1.38758	2.76356
C	-7.49929	-2.09040	3.66058
H	-7.11729	-3.06324	4.00051
H	-8.40347	-2.30274	3.07240
C	-7.82946	-1.19059	4.88251
C	-5.63394	3.33282	6.08702
C	-4.51886	4.06721	5.29376
H	-3.61478	4.15981	5.91187
H	-4.82518	5.09209	5.04032
C	-4.18598	3.26738	3.99055
C	-7.06223	-0.96112	1.42538
H	-7.89350	-0.26134	1.58369
H	-6.32809	-0.47124	0.77019
H	-7.44951	-1.84689	0.89824
C	-5.26879	-2.35597	2.47326
H	-4.55463	-1.94259	1.74892
H	-4.71889	-2.61125	3.39005
H	-5.67269	-3.28697	2.04618
C	-9.31485	-0.76061	4.93403
H	-9.92715	-1.66995	5.06047
H	-9.46295	-0.16742	5.85241
C	-7.50889	-1.91026	6.21951
H	-7.81893	-1.24901	7.04645
H	-8.15586	-2.80217	6.28338
C	-6.91303	4.18447	6.26550
H	-7.62455	3.60162	6.87485
H	-6.65025	5.06801	6.87219
C	-5.14682	2.92613	7.50336
H	-4.90723	3.85225	8.05387
H	-5.99507	2.46480	8.03710
C	-4.66499	4.05706	2.75582
H	-4.43501	3.52281	1.82312
H	-5.74885	4.22990	2.78817
H	-4.15804	5.03372	2.71897
C	-2.66809	3.05304	3.87854
H	-2.29262	2.40281	4.68124
H	-2.38426	2.60991	2.91468
H	-2.15753	4.02510	3.95800
C	-0.29711	3.46198	-3.52298
C	0.77043	4.40164	-3.49008
C	-1.38421	3.70558	-4.39724
C	0.74820	5.49467	-4.37334
C	-1.34680	4.79058	-5.28518
C	-0.28048	5.67887	-5.28939
H	-0.25792	6.52062	-5.98686
C	0.29711	-3.46198	-3.52298
C	-0.77043	-4.40164	-3.49008
C	1.38421	-3.70558	-4.39724
C	-0.74820	-5.49467	-4.37334
C	1.34680	-4.79058	-5.28518
C	0.28048	-5.67887	-5.28939
H	0.25792	-6.52062	-5.98686

C	5.07663	-0.83851	3.34254
C	5.02542	-1.99318	4.15010
C	6.05680	0.14564	3.58479
C	5.88935	-2.09642	5.25005
C	6.90578	0.00529	4.69227
C	6.81746	-1.09882	5.53587
H	7.46089	-1.17627	6.41666
C	6.42650	1.38758	2.76356
C	7.49929	2.09040	3.66058
H	7.11729	3.06324	4.00051
H	8.40347	2.30274	3.07240
C	7.82946	1.19059	4.88251
C	5.63394	-3.33282	6.08702
C	4.51886	-4.06721	5.29376
H	3.61478	-4.15981	5.91187
H	4.82518	-5.09209	5.04032
C	4.18598	-3.26738	3.99055
C	2.73449	-2.98765	-4.50400
C	3.42544	-3.68072	-5.72396
H	4.42543	-4.03830	-5.43967
H	3.57855	-2.94979	-6.53041
C	2.53578	-4.84956	-6.22054
C	-1.92670	-6.43110	-4.22397
C	-2.71207	-5.81064	-3.04588
H	-2.78708	-6.52385	-2.21280
H	-3.74387	-5.58318	-3.34807
C	-1.99852	-4.50344	-2.56052
C	-2.73449	2.98765	-4.50400
C	-3.42544	3.68072	-5.72396
H	-4.42543	4.03830	-5.43967
H	-3.57855	2.94979	-6.53041
C	-2.53578	4.84956	-6.22054
C	1.92670	6.43110	-4.22397
C	2.71207	5.81064	-3.04588
H	2.78708	6.52385	-2.21280
H	3.74387	5.58318	-3.34807
C	1.99852	4.50344	-2.56052
C	2.63241	-1.48377	-4.76415
H	2.21007	-0.94724	-3.90902
H	3.62991	-1.06062	-4.95768
H	1.99253	-1.26816	-5.63146
C	3.55733	-3.25520	-3.22862
H	4.53217	-2.74606	-3.28657
H	3.04190	-2.90985	-2.32301
H	3.73654	-4.33068	-3.09992
C	-2.99231	-3.33507	-2.66533
H	-2.61742	-2.42808	-2.17897
H	-3.22982	-3.09873	-3.71132
H	-3.92728	-3.61051	-2.15261
C	-1.57923	-4.67050	-1.08892
H	-1.13947	-3.75161	-0.68093
H	-2.46384	-4.90880	-0.47784
H	-0.84801	-5.48216	-0.97216
C	-2.63241	1.48377	-4.76415
H	-2.21007	0.94724	-3.90902
H	-3.62991	1.06062	-4.95768
H	-1.99253	1.26816	-5.63146
C	-3.55733	3.25520	-3.22862
H	-4.53217	2.74606	-3.28657
H	-3.04190	2.90985	-2.32301

H	-3.73654	4.33068	-3.09992
C	1.57923	4.67050	-1.08892
H	1.13947	3.75161	-0.68093
H	2.46384	4.90880	-0.47784
H	0.84801	5.48216	-0.97216
C	2.99231	3.33507	-2.66533
H	3.92728	3.61051	-2.15261
H	2.61742	2.42808	-2.17897
H	3.22982	3.09873	-3.71132
C	1.45625	7.87543	-3.91950
H	2.35501	8.49882	-3.77230
H	0.95826	8.27072	-4.82113
C	2.76348	6.47916	-5.52752
H	2.15336	6.96929	-6.30529
H	3.62498	7.14635	-5.35171
C	-3.25028	6.22089	-6.16329
H	-2.56307	6.97789	-6.57779
H	-4.10965	6.18446	-6.85466
C	-2.07216	4.63221	-7.68557
H	-2.97159	4.62316	-8.32529
H	-1.48824	5.51678	-7.99169
C	5.26879	2.35597	2.47326
H	4.55463	1.94259	1.74892
H	4.71889	2.61125	3.39005
H	5.67269	3.28697	2.04618
C	7.06223	0.96112	1.42538
H	7.89350	0.26134	1.58369
H	6.32809	0.47124	0.77019
H	7.44951	1.84689	0.89824
C	2.66809	-3.05304	3.87854
H	2.29262	-2.40281	4.68124
H	2.38426	-2.60991	2.91468
H	2.15753	-4.02510	3.95800
C	4.66499	-4.05706	2.75582
H	4.43501	-3.52281	1.82312
H	5.74885	-4.22990	2.78817
H	4.15804	-5.03372	2.71897
C	6.91303	-4.18447	6.26550
H	7.62455	-3.60162	6.87485
H	6.65025	-5.06801	6.87219
C	5.14682	-2.92613	7.50336
H	4.90723	-3.85225	8.05387
H	5.99507	-2.46480	8.03710
C	9.31485	0.76061	4.93403
H	9.92715	1.66995	5.06047
H	9.46295	0.16742	5.85241
C	7.50889	1.91026	6.21951
H	7.81893	1.24901	7.04645
H	8.15586	2.80217	6.28338
C	3.25028	-6.22089	-6.16329
H	2.56307	-6.97789	-6.57779
H	4.10965	-6.18446	-6.85466
C	2.07216	-4.63221	-7.68557
H	2.97159	-4.62316	-8.32529
H	1.48824	-5.51678	-7.99169
C	-2.76348	-6.47916	-5.52752
H	-2.15336	-6.96929	-6.30529
H	-3.62498	-7.14635	-5.35171
C	-1.45625	-7.87543	-3.91950
H	-2.35501	-8.49882	-3.77230

H	-0.95826	-8.27072	-4.82113
C	-3.95464	1.97316	7.54712
H	-4.18387	1.02246	7.04484
H	-3.06708	2.39958	7.05734
H	-3.67793	1.74750	8.58669
C	3.95464	-1.97316	7.54712
H	4.18387	-1.02246	7.04484
H	3.06708	-2.39958	7.05734
H	3.67793	-1.74750	8.58669
C	-7.60788	4.62760	4.98103
H	-6.97299	5.29334	4.37945
H	-7.87585	3.76370	4.35500
H	-8.53234	5.17648	5.21017
C	7.60788	-4.62760	4.98103
H	6.97299	-5.29334	4.37945
H	7.87585	-3.76370	4.35500
H	8.53234	-5.17648	5.21017
C	-6.05003	-2.31149	6.42449
H	-5.69810	-3.00132	5.64359
H	-5.38830	-1.43367	6.41211
H	-5.92046	-2.81811	7.39132
C	6.05003	2.31149	6.42449
H	5.69810	3.00132	5.64359
H	5.38830	1.43367	6.41211
H	5.92046	2.81811	7.39132
C	-9.82169	0.04207	3.73905
H	-9.22312	0.95263	3.58866
H	-9.78543	-0.54053	2.80762
H	-10.86631	0.34791	3.89278
C	9.82169	-0.04207	3.73905
H	9.22312	-0.95263	3.58866
H	9.78543	0.54053	2.80762
H	10.86631	-0.34791	3.89278
C	1.24100	-3.37720	-7.94062
H	0.32168	-3.37778	-7.33766
H	1.79566	-2.45903	-7.69833
H	0.95127	-3.31392	-8.99908
C	-1.24100	3.37720	-7.94062
H	-0.32168	3.37778	-7.33766
H	-1.79566	2.45903	-7.69833
H	-0.95127	3.31392	-8.99908
C	3.71597	-6.67838	-4.78379
H	4.48428	-6.01158	-4.36708
H	2.87856	-6.71225	-4.07139
H	4.15325	-7.68556	-4.83684
C	-3.71597	6.67838	-4.78379
H	-4.48428	6.01158	-4.36708
H	-2.87856	6.71225	-4.07139
H	-4.15325	7.68556	-4.83684
C	-0.51716	-8.02951	-2.72568
H	0.39584	-7.43090	-2.85773
H	-0.99058	-7.71020	-1.78607
H	-0.21716	-9.07966	-2.60093
C	0.51716	8.02951	-2.72568
H	-0.39584	7.43090	-2.85773
H	0.99058	7.71020	-1.78607
H	0.21716	9.07966	-2.60093
C	-3.25028	-5.13150	-6.05387
H	-3.92215	-4.63102	-5.34193
H	-2.40807	-4.45200	-6.24954

```
H          -3.80593        -5.26023        -6.99360
C           3.25028         5.13150        -6.05387
H           3.92215         4.63102        -5.34193
H           2.40807         4.45200        -6.24954
H           3.80593         5.26023        -6.99360
```

7.8.22 4Mes*

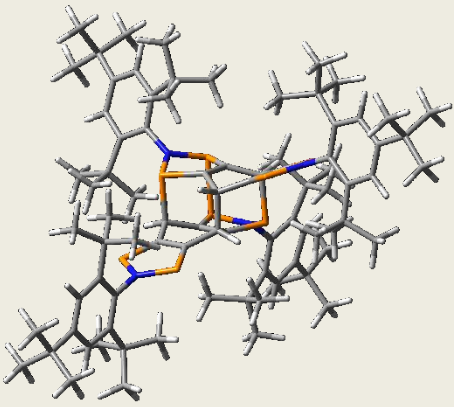

```
216
C84H120N4P8 @ PBE-D3/def2-SVP
P           1.70474         1.67416         0.91997
N           1.17089         2.01334         2.56235
C           0.00315         1.30288         0.33572
C           2.16636        -0.28423         0.99972
P          -0.32296         1.21010         3.04491
C           1.69982         3.05812         3.43053
C          -0.91523         1.03960         1.32463
C          -0.24049         0.77126        -1.06343
C           2.66114        -0.60419        -0.38044
H           2.95798        -0.37557         1.76369
C           0.91523        -1.03960         1.32463
P           0.32296        -1.21010         3.04491
C          -2.16636         0.28423         0.99972
C          -1.71075         0.86305        -1.40020
H           0.37239         1.29865        -1.82284
C           0.24049        -0.77126        -1.06343
P           4.33966        -0.64091        -0.84014
C           1.71075        -0.86305        -1.40020
C          -0.00315        -1.30288         0.33572
N          -1.17089        -2.01334         2.56235
C           1.40594         5.20218         4.55347
C           3.13767         3.74690         5.28858
P          -1.70474        -1.67416         0.91997
H          -2.95798         0.37557         1.76369
C          -2.66114         0.60419        -0.38044
P          -2.33123         1.21161        -2.98575
H          -0.37239        -1.29865        -1.82284
N           3.98439        -1.02567        -2.49376
P           2.33123        -1.21161        -2.98575
C          -1.69982        -3.05812         3.43053
H           0.87244         6.15310         4.65223
C           2.41738         4.92853         5.48095
H           3.97690         3.53544         5.95574
P          -4.33966         0.64091        -0.84014
N          -3.98439         1.02567        -2.49376
C           5.05403        -1.18751        -3.45256
C          -2.82518        -2.80439         4.28958
C          -5.05403         1.18751        -3.45256
C          -3.13767        -3.74690         5.28858
C          -1.40594        -5.20218         4.55347
C           6.74975        -2.55655        -4.50281
C           6.46275        -0.28795        -5.20614
H          -3.97690        -3.53544         5.95574
C          -2.41738        -4.92853         5.48095
H          -0.87244        -6.15310         4.65223
C          -6.46275         0.28795        -5.20614
C          -6.74975         2.55655        -4.50281
H           7.28213        -3.50956        -4.59465
```

C	7.14269	-1.50253	-5.34051
H	6.75124	0.54242	-5.85563
H	-6.75124	-0.54242	-5.85563
C	-7.14269	1.50253	-5.34051
H	-7.28213	3.50956	-4.59465
C	-5.41967	0.08012	-4.27853
C	-5.71806	2.45322	-3.55232
C	1.03872	4.33883	3.50336
C	2.82518	2.80439	4.28958
C	5.71806	-2.45322	-3.55232
C	5.41967	-0.08012	-4.27853
C	-1.03872	-4.33883	3.50336
C	-3.77424	-1.58134	4.17320
C	0.03088	-4.90710	2.51350
C	-5.47360	3.70064	-2.64555
C	-4.71594	-1.31336	-4.32585
C	-0.03088	4.90710	2.51350
C	3.77424	1.58134	4.17320
C	5.47360	-3.70064	-2.64555
C	4.71594	1.31336	-4.32585
C	8.28216	-1.71427	-6.35298
C	-2.71581	-5.92228	6.61569
C	2.71581	5.92228	6.61569
C	-8.28216	1.71427	-6.35298
C	-5.68209	-2.39706	-4.86936
H	-6.61814	-2.44555	-4.27712
H	-5.95055	-2.25001	-5.93327
H	-5.18679	-3.38579	-4.80437
C	-3.52625	-1.21366	-5.31048
H	-2.76154	-0.49538	-4.95256
H	-3.03765	-2.20401	-5.42431
H	-3.86685	-0.87657	-6.31020
C	-4.21028	-1.86281	-2.97077
H	-3.33096	-1.32483	-2.57356
H	-5.00587	-1.85089	-2.20033
H	-3.89905	-2.91799	-3.10992
C	-4.00449	3.95450	-2.23134
H	-3.61938	3.22535	-1.49630
H	-3.32855	3.96214	-3.10883
H	-3.93969	4.95277	-1.75232
C	-5.92606	4.99748	-3.36360
H	-5.42606	5.11800	-4.34586
H	-7.02061	5.04993	-3.52140
H	-5.65738	5.86953	-2.73544
C	-6.34226	3.54538	-1.37433
H	-7.41123	3.41770	-1.63916
H	-6.03287	2.66553	-0.77544
H	-6.24715	4.44587	-0.73199
C	-9.57154	2.09802	-5.59160
H	-9.44141	3.02938	-5.00536
H	-10.40803	2.26168	-6.30232
H	-9.86881	1.29506	-4.88715
C	-7.89488	2.85532	-7.32096
H	-6.97009	2.60400	-7.87860
H	-8.70586	3.03124	-8.05783
H	-7.71561	3.80784	-6.78387
C	-8.56633	0.44956	-7.18159
H	-7.68228	0.13642	-7.77293
H	-8.87335	-0.40266	-6.54236
H	-9.39175	0.64560	-7.89516

C	4.00449	-3.95450	-2.23134
H	3.61938	-3.22535	-1.49630
H	3.32855	-3.96214	-3.10883
H	3.93969	-4.95277	-1.75232
C	5.92606	-4.99748	-3.36360
H	5.42606	-5.11800	-4.34586
H	7.02061	-5.04993	-3.52140
H	5.65738	-5.86953	-2.73544
C	6.34226	-3.54538	-1.37433
H	7.41123	-3.41770	-1.63916
H	6.03287	-2.66553	-0.77544
H	6.24715	-4.44587	-0.73199
C	3.52625	1.21366	-5.31048
H	3.86685	0.87657	-6.31020
H	2.76154	0.49538	-4.95256
H	3.03765	2.20401	-5.42431
C	5.68209	2.39706	-4.86936
H	6.61814	2.44555	-4.27712
H	5.95055	2.25001	-5.93327
H	5.18679	3.38579	-4.80437
C	4.21028	1.86281	-2.97077
H	3.33096	1.32483	-2.57356
H	5.00587	1.85089	-2.20033
H	3.89905	2.91799	-3.10992
C	7.89488	-2.85532	-7.32096
H	8.70586	-3.03124	-8.05783
H	7.71561	-3.80784	-6.78387
H	6.97009	-2.60400	-7.87860
C	8.56633	-0.44956	-7.18159
H	7.68228	-0.13642	-7.77293
H	8.87335	0.40266	-6.54236
H	9.39175	-0.64560	-7.89516
C	9.57154	-2.09802	-5.59160
H	9.86881	-1.29506	-4.88715
H	9.44141	-3.02938	-5.00536
H	10.40803	-2.26168	-6.30232
C	3.00651	0.26427	4.37446
H	2.57368	0.21374	5.39359
H	2.16702	0.16850	3.67087
H	3.67108	-0.61382	4.23973
C	4.50534	1.64429	2.81138
H	5.12588	0.73755	2.65220
H	3.82285	1.74871	1.94633
H	5.17614	2.52644	2.78226
C	4.88912	1.57642	5.24392
H	5.55026	2.46281	5.17537
H	4.48424	1.51681	6.27378
H	5.52356	0.68118	5.08642
C	3.86248	5.44060	7.52125
H	3.63023	4.46615	7.99634
H	4.81359	5.33308	6.96164
H	4.03556	6.17585	8.33275
C	1.44672	6.09124	7.48168
H	0.59186	6.46970	6.88667
H	1.14236	5.12404	7.93026
H	1.63082	6.81216	8.30520
C	3.11241	7.28779	6.00958
H	2.30484	7.70779	5.37763
H	3.32746	8.02207	6.81356
H	4.01880	7.19164	5.37819

```
C          -0.10444         6.45424         2.60372
H          -0.51997         6.81783         3.56392
H           0.88595         6.92761         2.44829
H          -0.78397         6.81993         1.80870
C           0.31524         4.60591         1.03936
H           1.38239         4.81059         0.82207
H           0.08545         3.56681         0.75361
H          -0.29635         5.24960         0.37456
C          -1.44672         4.38994         2.84971
H          -1.52941         3.29056         2.75922
H          -1.72592         4.65809         3.88876
H          -2.19196         4.83964         2.16075
C           0.10444        -6.45424         2.60372
H           0.51997        -6.81783         3.56392
H          -0.88595        -6.92761         2.44829
H           0.78397        -6.81993         1.80870
C           1.44672        -4.38994         2.84971
H           1.52941        -3.29056         2.75922
H           1.72592        -4.65809         3.88876
H           2.19196        -4.83964         2.16075
C          -0.31524        -4.60591         1.03936
H          -1.38239        -4.81059         0.82207
H          -0.08545        -3.56681         0.75361
H           0.29635        -5.24960         0.37456
C          -1.44672        -6.09124         7.48168
H          -1.63082        -6.81216         8.30520
H          -0.59186        -6.46970         6.88667
H          -1.14236        -5.12404         7.93026
C          -3.11241        -7.28779         6.00958
H          -4.01880        -7.19164         5.37819
H          -2.30484        -7.70779         5.37763
H          -3.32746        -8.02207         6.81356
C          -3.86248        -5.44060         7.52125
H          -3.63023        -4.46615         7.99634
H          -4.81359        -5.33308         6.96164
H          -4.03556        -6.17585         8.33275
C          -4.50534        -1.64429         2.81138
H          -5.12588        -0.73755         2.65220
H          -3.82285        -1.74871         1.94633
H          -5.17614        -2.52644         2.78226
C          -4.88912        -1.57642         5.24392
H          -5.55026        -2.46281         5.17537
H          -4.48424        -1.51681         6.27378
H          -5.52356        -0.68118         5.08642
C          -3.00651        -0.26427         4.37446
H          -2.57368        -0.21374         5.39359
H          -2.16702        -0.16850         3.67087
H          -3.67108         0.61382         4.23973
```

```
216
C84H120N4P8 @ PBE-D3/def2-TZVP
P           1.68394         1.66940         0.91178
N           1.15587         1.99859         2.53643
C          -0.00069         1.29901         0.32204
C           2.15736        -0.26894         0.97807
P          -0.31776         1.19215         3.01114
C           1.66249         3.04808         3.41432
C          -0.91337         1.02913         1.30318
```

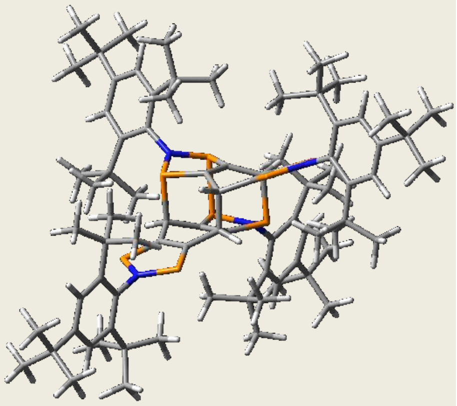

C	-0.23920	0.77100	-1.07553
C	2.64983	-0.59342	-0.39818
H	2.94588	-0.35868	1.73351
C	0.91337	-1.02913	1.30318
P	0.31776	-1.19215	3.01114
C	-2.15736	0.26894	0.97807
C	-1.70482	0.86120	-1.41056
H	0.37068	1.29429	-1.82747
C	0.23920	-0.77100	-1.07553
P	4.31552	-0.65147	-0.85908
C	1.70482	-0.86120	-1.41056
C	0.00069	-1.29901	0.32204
N	-1.15587	-1.99859	2.53643
C	1.30295	5.15581	4.55344
C	3.02909	3.72427	5.31033
P	-1.68394	-1.66940	0.91178
H	-2.94588	0.35868	1.73351
C	-2.64983	0.59342	-0.39818
P	-2.32922	1.23827	-2.97527
H	-0.37068	-1.29429	-1.82747
N	3.96599	-1.05959	-2.48912
P	2.32922	-1.23827	-2.97527
C	-1.66249	-3.04808	3.41432
H	0.76005	6.09116	4.65080
C	2.28112	4.87876	5.50439
H	3.84575	3.51875	5.99333
P	-4.31552	0.65147	-0.85908
N	-3.96599	1.05959	-2.48912
C	5.03564	-1.25117	-3.44352
C	-2.76465	-2.80020	4.28970
C	-5.03564	1.25117	-3.44352
C	-3.02909	-3.72427	5.31033
C	-1.30295	-5.15581	4.55344
C	6.71046	-2.65032	-4.45646
C	6.43552	-0.41166	-5.21534
H	-3.84575	-3.51875	5.99333
C	-2.28112	-4.87876	5.50439
H	-0.76005	-6.09116	4.65080
C	-6.43552	0.41166	-5.21534
C	-6.71046	2.65032	-4.45646
H	7.23178	-3.60106	-4.52668
C	7.10282	-1.62685	-5.31916
H	6.72603	0.39218	-5.88267
H	-6.72603	-0.39218	-5.88267
C	-7.10282	1.62685	-5.31916
H	-7.23178	3.60106	-4.52668
C	-5.40515	0.17278	-4.29124
C	-5.68577	2.51758	-3.51306
C	0.98626	4.31252	3.48101
C	2.76465	2.80020	4.28970
C	5.68577	-2.51758	-3.51306
C	5.40515	-0.17278	-4.29124
C	-0.98626	-4.31252	3.48101
C	-3.73987	-1.60380	4.17593
C	0.03248	-4.89332	2.45194
C	-5.43241	3.74353	-2.58739
C	-4.72682	-1.22679	-4.36765
C	-0.03248	4.89332	2.45194
C	3.73987	1.60380	4.17593
C	5.43241	-3.74353	-2.58739

C	4.72682	1.22679	-4.36765
C	8.23154	-1.87192	-6.32733
C	-2.51844	-5.84640	6.66786
C	2.51844	5.84640	6.66786
C	-8.23154	1.87192	-6.32733
C	-5.71367	-2.28383	-4.91889
H	-6.64475	-2.31242	-4.33458
H	-5.96968	-2.12664	-5.97500
H	-5.24120	-3.27370	-4.85379
C	-3.54666	-1.13621	-5.35891
H	-2.76717	-0.45211	-4.99529
H	-3.09260	-2.13015	-5.49435
H	-3.88712	-0.77376	-6.33963
C	-4.21889	-1.80985	-3.03228
H	-3.34208	-1.28893	-2.63274
H	-5.00548	-1.81112	-2.26556
H	-3.91896	-2.85469	-3.20062
C	-3.96599	3.98184	-2.16875
H	-3.58698	3.23718	-1.46043
H	-3.29821	4.01124	-3.04057
H	-3.90242	4.95965	-1.66836
C	-5.87318	5.05445	-3.28072
H	-5.38890	5.17853	-4.26016
H	-6.96053	5.12448	-3.41730
H	-5.58076	5.90310	-2.64698
C	-6.29991	3.58093	-1.32070
H	-7.36006	3.46137	-1.58701
H	-5.99520	2.70387	-0.73308
H	-6.20045	4.47156	-0.68085
C	-9.52119	2.23965	-5.56549
H	-9.38789	3.14477	-4.95686
H	-10.34253	2.42673	-6.27384
H	-9.82415	1.42328	-4.89395
C	-7.83799	3.03715	-7.25779
H	-6.91970	2.79925	-7.81398
H	-8.64100	3.23246	-7.98466
H	-7.65976	3.96349	-6.69436
C	-8.51886	0.63833	-7.19405
H	-7.64055	0.34256	-7.78648
H	-8.83259	-0.22338	-6.58680
H	-9.33346	0.86474	-7.89709
C	3.96599	-3.98184	-2.16875
H	3.58698	-3.23718	-1.46043
H	3.29821	-4.01124	-3.04057
H	3.90242	-4.95965	-1.66836
C	5.87318	-5.05445	-3.28072
H	5.38890	-5.17853	-4.26016
H	6.96053	-5.12448	-3.41730
H	5.58076	-5.90310	-2.64698
C	6.29991	-3.58093	-1.32070
H	7.36006	-3.46137	-1.58701
H	5.99520	-2.70387	-0.73308
H	6.20045	-4.47156	-0.68085
C	3.54666	1.13621	-5.35891
H	3.88712	0.77376	-6.33963
H	2.76717	0.45211	-4.99529
H	3.09260	2.13015	-5.49435
C	5.71367	2.28383	-4.91889
H	6.64475	2.31242	-4.33458
H	5.96968	2.12664	-5.97500

H	5.24120	3.27370	-4.85379
C	4.21889	1.80985	-3.03228
H	3.34208	1.28893	-2.63274
H	5.00548	1.81112	-2.26556
H	3.91896	2.85469	-3.20062
C	7.83799	-3.03715	-7.25779
H	8.64100	-3.23246	-7.98466
H	7.65976	-3.96349	-6.69436
H	6.91970	-2.79925	-7.81398
C	8.51886	-0.63833	-7.19405
H	7.64055	-0.34256	-7.78648
H	8.83259	0.22338	-6.58680
H	9.33346	-0.86474	-7.89709
C	9.52119	-2.23965	-5.56549
H	9.82415	-1.42328	-4.89395
H	9.38789	-3.14477	-4.95686
H	10.34253	-2.42673	-6.27384
C	3.01278	0.26713	4.36918
H	2.55960	0.21225	5.36944
H	2.20680	0.13185	3.64707
H	3.71079	-0.57705	4.26226
C	4.47635	1.67935	2.82207
H	5.10994	0.79051	2.67667
H	3.80205	1.76420	1.96051
H	5.12478	2.56738	2.79864
C	4.84945	1.62417	5.24730
H	5.47356	2.52716	5.18906
H	4.44778	1.53588	6.26704
H	5.50636	0.75905	5.07600
C	3.63212	5.36470	7.60779
H	3.39844	4.38318	8.04564
H	4.59987	5.29041	7.09031
H	3.75153	6.08007	8.43435
C	1.21696	5.97805	7.48482
H	0.38938	6.35472	6.86786
H	0.91406	5.00415	7.89585
H	1.36092	6.67725	8.32263
C	2.91500	7.22997	6.11369
H	2.13186	7.64609	5.46491
H	3.08170	7.93842	6.93945
H	3.84102	7.16342	5.52434
C	-0.17411	6.42613	2.61371
H	-0.67882	6.71424	3.54669
H	0.79694	6.93968	2.56476
H	-0.79657	6.79979	1.78874
C	0.44142	4.68487	1.00090
H	1.49833	4.96086	0.88097
H	0.30431	3.65467	0.66112
H	-0.15518	5.31657	0.32565
C	-1.45101	4.32086	2.63315
H	-1.50835	3.25479	2.38877
H	-1.79578	4.45236	3.66881
H	-2.15027	4.84815	1.96536
C	0.17411	-6.42613	2.61371
H	0.67882	-6.71424	3.54669
H	-0.79694	-6.93968	2.56476
H	0.79657	-6.79979	1.78874
C	1.45101	-4.32086	2.63315
H	1.50835	-3.25479	2.38877
H	1.79578	-4.45236	3.66881

```
H          2.15027       -4.84815        1.96536
C         -0.44142       -4.68487        1.00090
H         -1.49833       -4.96086        0.88097
H         -0.30431       -3.65467        0.66112
H          0.15518       -5.31657        0.32565
C         -1.21696       -5.97805        7.48482
H         -1.36092       -6.67725        8.32263
H         -0.38938       -6.35472        6.86786
H         -0.91406       -5.00415        7.89585
C         -2.91500       -7.22997        6.11369
H         -3.84102       -7.16342        5.52434
H         -2.13186       -7.64609        5.46491
H         -3.08170       -7.93842        6.93945
C         -3.63212       -5.36470        7.60779
H         -3.39844       -4.38318        8.04564
H         -4.59987       -5.29041        7.09031
H         -3.75153       -6.08007        8.43435
C         -4.47635       -1.67935        2.82207
H         -5.10994       -0.79051        2.67667
H         -3.80205       -1.76420        1.96051
H         -5.12478       -2.56738        2.79864
C         -4.84945       -1.62417        5.24730
H         -5.47356       -2.52716        5.18906
H         -4.44778       -1.53588        6.26704
H         -5.50636       -0.75905        5.07600
C         -3.01278       -0.26713        4.36918
H         -2.55960       -0.21225        5.36944
H         -2.20680       -0.13185        3.64707
H         -3.71079        0.57705        4.26226
```

7.8.23 4Oma

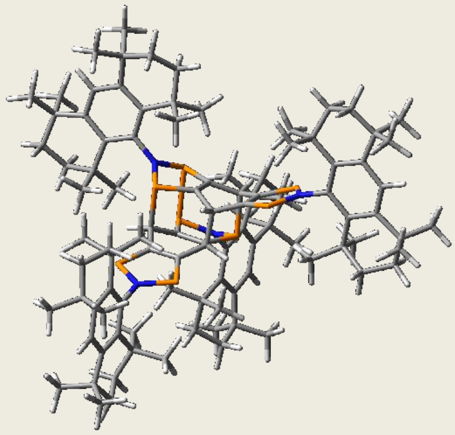

```
248
C100H136N4P8 @ PBE-D3/def2-SVP
P          1.85205        1.52736        1.04311
N          1.34418        1.97678        2.66744
C          0.12677        1.30070        0.46691
P         -0.18352        1.25806        3.18982
C         -0.80032        1.11207        1.46472
C         -0.16785        0.78935       -0.92807
C         -2.11760        0.47607        1.14670
C         -1.62398        1.02706       -1.25389
H          0.48856        1.25631       -1.69067
H         -2.89601        0.64567        1.91095
C         -2.58664        0.85589       -0.22716
P         -2.21795        1.47670       -2.82235
P         -4.25080        1.12335       -0.65651
N         -3.87563        1.50051       -2.30814
C         -4.93178        1.84816       -3.23681
C         -5.62013        0.77493       -3.89425
C         -5.21903        3.24027       -3.43337
C         -6.65131        1.14112       -4.80551
C         -6.26897        3.55239       -4.34364
C         -6.92984        2.49984       -4.98523
H         -7.73222        2.76138       -5.68679
C         -4.40158        4.41343       -2.80972
C         -5.29351        5.68093       -2.76439
H         -4.67815        6.51492       -2.36707
```

H	-6.10598	5.51777	-2.02302
C	-5.87713	6.05895	-4.10992
H	-5.05540	6.23616	-4.83477
H	-6.43636	7.01622	-4.04473
C	-6.81339	4.96520	-4.64929
C	-7.46610	0.17111	-5.68893
C	-7.27496	-1.28903	-5.24696
H	-7.58450	-1.96237	-6.07407
H	-7.95716	-1.51417	-4.40077
C	-5.84465	-1.56492	-4.83180
H	-5.15079	-1.36044	-5.67631
H	-5.71949	-2.63968	-4.58453
C	-5.38649	-0.73887	-3.60212
C	-3.90061	4.24784	-1.35628
H	-4.71354	3.92253	-0.67852
H	-3.04679	3.55558	-1.24734
H	-3.55066	5.23865	-0.99876
C	-3.18068	4.69182	-3.72009
H	-2.45582	3.85481	-3.67855
H	-3.47067	4.81986	-4.78080
H	-2.65648	5.61209	-3.38585
C	-8.19841	5.09843	-3.97093
H	-8.64844	6.08613	-4.20333
H	-8.89537	4.31125	-4.32098
H	-8.12054	5.00387	-2.86955
C	-6.97620	5.19145	-6.16780
H	-7.75858	4.55045	-6.61968
H	-7.26894	6.24492	-6.35699
H	-6.02454	4.99875	-6.70318
C	-8.97605	0.48787	-5.63023
H	-9.23072	1.46717	-6.08120
H	-9.53998	-0.28571	-6.19134
H	-9.34233	0.48739	-4.58367
C	-6.97620	0.33117	-7.14857
H	-7.56802	-0.31760	-7.82746
H	-7.08027	1.37846	-7.49542
H	-5.90779	0.05489	-7.24974
C	-6.24825	-1.13812	-2.37953
H	-5.86580	-0.66532	-1.45317
H	-7.30471	-0.82742	-2.49322
H	-6.21869	-2.23859	-2.23418
C	-3.93380	-1.20394	-3.34821
H	-3.24064	-0.82242	-4.12297
H	-3.54259	-0.93393	-2.35075
H	-3.91342	-2.31192	-3.40352
C	2.02834	3.04328	3.40324
C	1.64193	4.42409	3.16657
C	3.10615	2.69580	4.29840
C	2.42491	5.43583	3.79692
C	3.78253	3.74586	4.98400
C	3.41498	5.05948	4.70651
H	3.95482	5.86046	5.22519
C	3.67823	1.26103	4.44270
C	4.53510	1.10666	5.72494
H	5.02988	0.11386	5.67621
H	3.85467	1.06274	6.60214
C	5.55749	2.20166	5.92597
H	6.26482	2.22768	5.06967
H	6.17473	2.00585	6.82848
C	4.87728	3.57044	6.06039

C	2.32116	6.96537	3.58355
C	1.36064	7.29356	2.43497
H	1.87698	7.15515	1.46169
H	1.07520	8.36560	2.48361
C	0.14966	6.39516	2.51014
H	-0.61677	6.70044	1.76793
H	-0.33229	6.49651	3.50682
C	0.46141	4.89598	2.25564
C	2.62607	0.15569	4.58004
H	2.11205	-0.04213	3.63280
H	3.10597	-0.79364	4.89303
H	1.85356	0.41208	5.33257
C	4.58475	1.00318	3.21403
H	4.94728	-0.04660	3.20234
H	4.05853	1.20355	2.26041
H	5.46745	1.67164	3.22209
C	-0.88854	4.20450	2.54182
H	-0.99720	3.24171	2.02148
H	-1.05225	4.05422	3.62659
H	-1.71052	4.84663	2.16222
C	0.81854	4.74363	0.76064
H	0.96412	3.68811	0.47104
H	-0.00545	5.14183	0.13173
H	1.74907	5.28499	0.50133
C	5.98222	4.64368	5.95297
H	5.63472	5.65798	6.23107
H	6.80966	4.38769	6.64620
H	6.39747	4.69150	4.92617
C	4.20187	3.70013	7.44665
H	4.95420	3.60392	8.25754
H	3.70740	4.68691	7.54906
H	3.42404	2.92670	7.60039
C	1.81712	7.63439	4.88363
H	2.48084	7.40829	5.74150
H	1.78455	8.73693	4.76059
H	0.80019	7.28692	5.15354
C	3.70565	7.55090	3.22415
H	3.59880	8.62576	2.96890
H	4.43505	7.47982	4.05472
H	4.13857	7.03090	2.34592
P	-1.85205	-1.52736	1.04311
N	-1.34418	-1.97678	2.66744
C	-0.12677	-1.30070	0.46691
P	0.18352	-1.25806	3.18982
C	0.80032	-1.11207	1.46472
C	0.16785	-0.78935	-0.92807
C	2.11760	-0.47607	1.14670
C	1.62398	-1.02706	-1.25389
H	-0.48856	-1.25631	-1.69067
H	2.89601	-0.64567	1.91095
C	2.58664	-0.85589	-0.22716
P	2.21795	-1.47670	-2.82235
P	4.25080	-1.12335	-0.65651
N	3.87563	-1.50051	-2.30814
C	4.93178	-1.84816	-3.23681
C	5.62013	-0.77493	-3.89425
C	5.21903	-3.24027	-3.43337
C	6.65131	-1.14112	-4.80551
C	6.26897	-3.55239	-4.34364
C	6.92984	-2.49984	-4.98523

H	7.73222	-2.76138	-5.68679
C	4.40158	-4.41343	-2.80972
C	5.29351	-5.68093	-2.76439
H	4.67815	-6.51492	-2.36707
H	6.10598	-5.51777	-2.02302
C	5.87713	-6.05895	-4.10992
H	5.05540	-6.23616	-4.83477
H	6.43636	-7.01622	-4.04473
C	6.81339	-4.96520	-4.64929
C	7.46610	-0.17111	-5.68893
C	7.27496	1.28903	-5.24696
H	7.58450	1.96237	-6.07407
H	7.95716	1.51417	-4.40077
C	5.84465	1.56492	-4.83180
H	5.15079	1.36044	-5.67631
H	5.71949	2.63968	-4.58453
C	5.38649	0.73887	-3.60212
C	3.90061	-4.24784	-1.35628
H	4.71354	-3.92253	-0.67852
H	3.04679	-3.55558	-1.24734
H	3.55066	-5.23865	-0.99876
C	3.18068	-4.69182	-3.72009
H	2.45582	-3.85481	-3.67855
H	3.47067	-4.81986	-4.78080
H	2.65648	-5.61209	-3.38585
C	8.19841	-5.09843	-3.97093
H	8.64844	-6.08613	-4.20333
H	8.89537	-4.31125	-4.32098
H	8.12054	-5.00387	-2.86955
C	6.97620	-5.19145	-6.16780
H	7.75858	-4.55045	-6.61968
H	7.26894	-6.24492	-6.35699
H	6.02454	-4.99875	-6.70318
C	8.97605	-0.48787	-5.63023
H	9.23072	-1.46717	-6.08120
H	9.53998	0.28571	-6.19134
H	9.34233	-0.48739	-4.58367
C	6.97620	-0.33117	-7.14857
H	7.56802	0.31760	-7.82746
H	7.08027	-1.37846	-7.49542
H	5.90779	-0.05489	-7.24974
C	6.24825	1.13812	-2.37953
H	5.86580	0.66532	-1.45317
H	7.30471	0.82742	-2.49322
H	6.21869	2.23859	-2.23418
C	3.93380	1.20394	-3.34821
H	3.24064	0.82242	-4.12297
H	3.54259	0.93393	-2.35075
H	3.91342	2.31192	-3.40352
C	-2.02834	-3.04328	3.40324
C	-1.64193	-4.42409	3.16657
C	-3.10615	-2.69580	4.29840
C	-2.42491	-5.43583	3.79692
C	-3.78253	-3.74586	4.98400
C	-3.41498	-5.05948	4.70651
H	-3.95482	-5.86046	5.22519
C	-3.67823	-1.26103	4.44270
C	-4.53510	-1.10666	5.72494
H	-5.02988	-0.11386	5.67621
H	-3.85467	-1.06274	6.60214

C	-5.55749	-2.20166	5.92597
H	-6.26482	-2.22768	5.06967
H	-6.17473	-2.00585	6.82848
C	-4.87728	-3.57044	6.06039
C	-2.32116	-6.96537	3.58355
C	-1.36064	-7.29356	2.43497
H	-1.87698	-7.15515	1.46169
H	-1.07520	-8.36560	2.48361
C	-0.14966	-6.39516	2.51014
H	0.61677	-6.70044	1.76793
H	0.33229	-6.49651	3.50682
C	-0.46141	-4.89598	2.25564
C	-2.62607	-0.15569	4.58004
H	-2.11205	0.04213	3.63280
H	-3.10597	0.79364	4.89303
H	-1.85356	-0.41208	5.33257
C	-4.58475	-1.00318	3.21403
H	-4.94728	0.04660	3.20234
H	-4.05853	-1.20355	2.26041
H	-5.46745	-1.67164	3.22209
C	0.88854	-4.20450	2.54182
H	0.99720	-3.24171	2.02148
H	1.05225	-4.05422	3.62659
H	1.71052	-4.84663	2.16222
C	-0.81854	-4.74363	0.76064
H	-0.96412	-3.68811	0.47104
H	0.00545	-5.14183	0.13173
H	-1.74907	-5.28499	0.50133
C	-5.98222	-4.64368	5.95297
H	-5.63472	-5.65798	6.23107
H	-6.80966	-4.38769	6.64620
H	-6.39747	-4.69150	4.92617
C	-4.20187	-3.70013	7.44665
H	-4.95420	-3.60392	8.25754
H	-3.70740	-4.68691	7.54906
H	-3.42404	-2.92670	7.60039
C	-1.81712	-7.63439	4.88363
H	-2.48084	-7.40829	5.74150
H	-1.78455	-8.73693	4.76059
H	-0.80019	-7.28692	5.15354
C	-3.70565	-7.55090	3.22415
H	-3.59880	-8.62576	2.96890
H	-4.43505	-7.47982	4.05472
H	-4.13857	-7.03090	2.34592

248
C100H136N4P8 @ PBE-D3/def2-TZVP

P	-1.86182	-1.49133	1.06510
N	-1.36960	-1.93798	2.67267
C	-0.14814	-1.29235	0.48404
P	0.15395	-1.24803	3.18420
C	0.77773	-1.11818	1.47447
C	0.15209	-0.79128	-0.90971
C	2.10016	-0.49927	1.15673
C	1.59939	-1.05103	-1.23363
H	-0.50868	-1.24348	-1.66488
H	2.87197	-0.68407	1.91194
C	2.56054	-0.88844	-0.21441

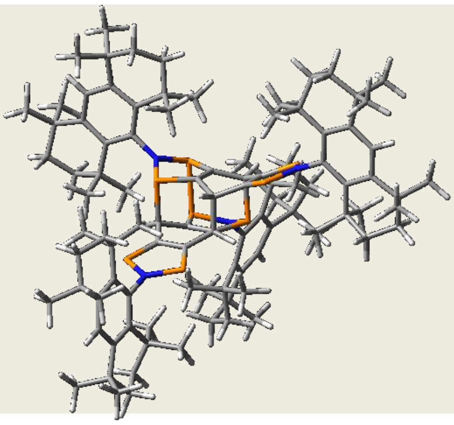

P	2.18662	-1.52115	-2.78562
P	4.20592	-1.18756	-0.64902
N	3.82774	-1.56715	-2.28071
C	4.87672	-1.93490	-3.21135
C	5.58887	-0.88025	-3.85707
C	5.12621	-3.32502	-3.41738
C	6.61001	-1.26457	-4.76016
C	6.15723	-3.65515	-4.33090
C	6.84655	-2.62106	-4.95516
H	7.63577	-2.89586	-5.65375
C	4.30559	-4.48250	-2.78196
C	5.18192	-5.75640	-2.74728
H	4.57005	-6.57151	-2.33010
H	6.01157	-5.59605	-2.03799
C	5.71690	-6.15131	-4.10494
H	4.87755	-6.32251	-4.79671
H	6.26580	-7.10517	-4.04882
C	6.64609	-5.07358	-4.67532
C	7.47236	-0.31629	-5.61223
C	7.28998	1.14790	-5.19624
H	7.61508	1.79895	-6.02374
H	7.95306	1.37854	-4.34792
C	5.85673	1.43857	-4.81283
H	5.18100	1.22648	-5.65872
H	5.73686	2.50829	-4.58048
C	5.37789	0.63470	-3.58146
C	3.83464	-4.30919	-1.32361
H	4.65913	-3.99880	-0.66776
H	3.00185	-3.60763	-1.20426
H	3.47912	-5.28790	-0.96496
C	3.06589	-4.75772	-3.66174
H	2.33834	-3.93865	-3.58132
H	3.32608	-4.86050	-4.72329
H	2.57092	-5.68490	-3.33148
C	8.05669	-5.25617	-4.07336
H	8.45960	-6.24323	-4.34787
H	8.75042	-4.48742	-4.44092
H	8.03486	-5.18445	-2.97676
C	6.72466	-5.28106	-6.20009
H	7.48939	-4.65378	-6.67796
H	6.98053	-6.33037	-6.41312
H	5.75752	-5.06164	-6.67508
C	8.96937	-0.65765	-5.48365
H	9.22206	-1.63871	-5.90833
H	9.56350	0.09630	-6.02256
H	9.28295	-0.65079	-4.42974
C	7.04875	-0.47695	-7.08877
H	7.68311	0.14982	-7.73431
H	7.14542	-1.52005	-7.42048
H	6.00155	-0.17840	-7.23830
C	6.22941	1.04478	-2.35964
H	5.81751	0.61989	-1.43405
H	7.26805	0.69956	-2.44470
H	6.23160	2.14141	-2.25604
C	3.93330	1.12321	-3.34978
H	3.25154	0.74988	-4.12586
H	3.53059	0.86363	-2.36449
H	3.93386	2.22243	-3.41155
C	-2.07666	-3.00012	3.39758
C	-1.73261	-4.37826	3.13014

C	-3.13989	-2.64285	4.29212
C	-2.56054	-5.37308	3.70975
C	-3.85077	-3.68316	4.94179
C	-3.54052	-4.99236	4.61679
H	-4.11584	-5.78320	5.09354
C	-3.65776	-1.20046	4.48950
C	-4.46893	-1.05792	5.79736
H	-4.91227	-0.04929	5.79842
H	-3.76853	-1.08436	6.64854
C	-5.54026	-2.10465	5.97853
H	-6.26985	-2.04938	5.15451
H	-6.10967	-1.92551	6.90514
C	-4.93309	-3.50787	6.02338
C	-2.52928	-6.88952	3.42820
C	-1.52380	-7.22611	2.32748
H	-1.97188	-7.03924	1.33886
H	-1.28581	-8.30131	2.36304
C	-0.28880	-6.38010	2.50393
H	0.50519	-6.69165	1.80783
H	0.11913	-6.51711	3.51967
C	-0.53505	-4.87119	2.26284
C	-2.57538	-0.12905	4.62403
H	-2.13153	0.12503	3.66402
H	-3.01259	0.79677	5.02666
H	-1.76674	-0.44437	5.29873
C	-4.58454	-0.87378	3.29744
H	-4.90235	0.18065	3.33115
H	-4.08893	-1.05945	2.33533
H	-5.48334	-1.50444	3.30796
C	0.81717	-4.23306	2.63258
H	0.97983	-3.27102	2.14393
H	0.92252	-4.10900	3.71878
H	1.62874	-4.89323	2.28820
C	-0.80042	-4.68395	0.75610
H	-0.79602	-3.62807	0.46801
H	-0.00489	-5.17892	0.17635
H	-1.76641	-5.10839	0.45271
C	-6.09339	-4.50883	5.85910
H	-5.80781	-5.54406	6.08991
H	-6.90242	-4.23661	6.55396
H	-6.49728	-4.48244	4.83682
C	-4.27008	-3.75295	7.39645
H	-5.01689	-3.67019	8.20158
H	-3.82808	-4.75868	7.43742
H	-3.46537	-3.03165	7.59405
C	-2.14580	-7.65360	4.71296
H	-2.83698	-7.43286	5.53777
H	-2.17579	-8.73824	4.52736
H	-1.13401	-7.39004	5.05125
C	-3.92093	-7.36421	2.96314
H	-3.86711	-8.42429	2.67028
H	-4.68533	-7.27325	3.74673
H	-4.25741	-6.78418	2.09176
P	1.86182	1.49133	1.06510
N	1.36960	1.93798	2.67267
C	0.14814	1.29235	0.48404
P	-0.15395	1.24803	3.18420
C	-0.77773	1.11818	1.47447
C	-0.15209	0.79128	-0.90971
C	-2.10016	0.49927	1.15673

C	-1.59939	1.05103	-1.23363
H	0.50868	1.24348	-1.66488
H	-2.87197	0.68407	1.91194
C	-2.56054	0.88844	-0.21441
P	-2.18662	1.52115	-2.78562
P	-4.20592	1.18756	-0.64902
N	-3.82774	1.56715	-2.28071
C	-4.87672	1.93490	-3.21135
C	-5.58887	0.88025	-3.85707
C	-5.12621	3.32502	-3.41738
C	-6.61001	1.26457	-4.76016
C	-6.15723	3.65515	-4.33090
C	-6.84655	2.62106	-4.95516
H	-7.63577	2.89586	-5.65375
C	-4.30559	4.48250	-2.78196
C	-5.18192	5.75640	-2.74728
H	-4.57005	6.57151	-2.33010
H	-6.01157	5.59605	-2.03799
C	-5.71690	6.15131	-4.10494
H	-4.87755	6.32251	-4.79671
H	-6.26580	7.10517	-4.04882
C	-6.64609	5.07358	-4.67532
C	-7.47236	0.31629	-5.61223
C	-7.28998	-1.14790	-5.19624
H	-7.61508	-1.79895	-6.02374
H	-7.95306	-1.37854	-4.34792
C	-5.85673	-1.43857	-4.81283
H	-5.18100	-1.22648	-5.65872
H	-5.73686	-2.50829	-4.58048
C	-5.37789	-0.63470	-3.58146
C	-3.83464	4.30919	-1.32361
H	-4.65913	3.99880	-0.66776
H	-3.00185	3.60763	-1.20426
H	-3.47912	5.28790	-0.96496
C	-3.06589	4.75772	-3.66174
H	-2.33834	3.93865	-3.58132
H	-3.32608	4.86050	-4.72329
H	-2.57092	5.68490	-3.33148
C	-8.05669	5.25617	-4.07336
H	-8.45960	6.24323	-4.34787
H	-8.75042	4.48742	-4.44092
H	-8.03486	5.18445	-2.97676
C	-6.72466	5.28106	-6.20009
H	-7.48939	4.65378	-6.67796
H	-6.98053	6.33037	-6.41312
H	-5.75752	5.06164	-6.67508
C	-8.96937	0.65765	-5.48365
H	-9.22206	1.63871	-5.90833
H	-9.56350	-0.09630	-6.02256
H	-9.28295	0.65079	-4.42974
C	-7.04875	0.47695	-7.08877
H	-7.68311	-0.14982	-7.73431
H	-7.14542	1.52005	-7.42048
H	-6.00155	0.17840	-7.23830
C	-6.22941	-1.04478	-2.35964
H	-5.81751	-0.61989	-1.43405
H	-7.26805	-0.69956	-2.44470
H	-6.23160	-2.14141	-2.25604
C	-3.93330	-1.12321	-3.34978
H	-3.25154	-0.74988	-4.12586

H	-3.53059	-0.86363	-2.36449
H	-3.93386	-2.22243	-3.41155
C	2.07666	3.00012	3.39758
C	1.73261	4.37826	3.13014
C	3.13989	2.64285	4.29212
C	2.56054	5.37308	3.70975
C	3.85077	3.68316	4.94179
C	3.54052	4.99236	4.61679
H	4.11584	5.78320	5.09354
C	3.65776	1.20046	4.48950
C	4.46893	1.05792	5.79736
H	4.91227	0.04929	5.79842
H	3.76853	1.08436	6.64854
C	5.54026	2.10465	5.97853
H	6.26985	2.04938	5.15451
H	6.10967	1.92551	6.90514
C	4.93309	3.50787	6.02338
C	2.52928	6.88952	3.42820
C	1.52380	7.22611	2.32748
H	1.97188	7.03924	1.33886
H	1.28581	8.30131	2.36304
C	0.28880	6.38010	2.50393
H	-0.50519	6.69165	1.80783
H	-0.11913	6.51711	3.51967
C	0.53505	4.87119	2.26284
C	2.57538	0.12905	4.62403
H	2.13153	-0.12503	3.66402
H	3.01259	-0.79677	5.02666
H	1.76674	0.44437	5.29873
C	4.58454	0.87378	3.29744
H	4.90235	-0.18065	3.33115
H	4.08893	1.05945	2.33533
H	5.48334	1.50444	3.30796
C	-0.81717	4.23306	2.63258
H	-0.97983	3.27102	2.14393
H	-0.92252	4.10900	3.71878
H	-1.62874	4.89323	2.28820
C	0.80042	4.68395	0.75610
H	0.79602	3.62807	0.46801
H	0.00489	5.17892	0.17635
H	1.76641	5.10839	0.45271
C	6.09339	4.50883	5.85910
H	5.80781	5.54406	6.08991
H	6.90242	4.23661	6.55396
H	6.49728	4.48244	4.83682
C	4.27008	3.75295	7.39645
H	5.01689	3.67019	8.20158
H	3.82808	4.75868	7.43742
H	3.46537	3.03165	7.59405
C	2.14580	7.65360	4.71296
H	2.83698	7.43286	5.53777
H	2.17579	8.73824	4.52736
H	1.13401	7.39004	5.05125
C	3.92093	7.36421	2.96314
H	3.86711	8.42429	2.67028
H	4.68533	7.27325	3.74673
H	4.25741	6.78418	2.09176

8 References

[1] C. B. Fischer, S. Xu, H. Zipse, *Chem. Eur. J.* **2006**, *12*, 5779–5784.

[2] N. Burford, J. A. C. Clyburne, P. Losier, T. M. Parks, in *Synthetic Methods of Organometallic and Inorganic Chemistry* (Ed.: H.H. Karsch), Georg Thieme Verlag, Stuttgart, **1996**.

[3] F. Reiß, A. Schulz, A. Villinger, N. Weding, *Dalton Trans.* **2010**, *39*, 9962.

[4] J. Bresien, D. Michalik, A. Schulz, A. Villinger, E. Zander, *Angew. Chem. Int. Ed.* **2021**, *60*, 1507–1512.

[5] E. Zander, L. Schweidt, S. Purschke, D. Michalik, A. Villinger, J. Bresien, A. Schulz, *ChemPlusChem* **2023**, *88*, e2023000.

[6] P. H. M. Budzelaar, *gNMR for Windows*, IvorySoft, **2006**.

[7] G. M. Sheldrick, *Acta Crystallogr. Sect. A: Found. Adv.* **2015**, *71*, 3–8.

[8] G. M. Sheldrick, *Acta Crystallogr. Sect. C: Struct. Chem.* **2015**, *71*, 3–8.

[9] G. M. Sheldrick, *SADABS Version 2*, University of Göttingen, Germany, **2004**.

[10] S. Zhang, W. Wang, S. Liu, Y. Sui, Z. Zhang, G. Tan, Q. Sun, X. Wang, *Science China Chemistry* **2017**, *60*, 1439–1443.

[11] J. Bresien, C. Hering-Junghans, A. Schulz, M. Thomas, A. Villinger, *Organometallics* **2018**, *37*, 2571–2580.

[12] M. Hachiya, M. Ito, T. Matsuo, D. Hashizume, H. Fueno, K. Tanaka, K. Tamao, *Org. Lett.* **2011**, *13*, 2666–2669.

[13] K. M. Carsch, I. M. DiMucci, D. A. Iovan, A. Li, S.-L. Zheng, C. J. Titus, S. J. Lee, K. D. Irwin, D. Nordlund, K. M. Lancaster, T. A. Betley, *Science* **2019**, *365*, 1138–1143.

[14] T. Matsuo, K. Suzuki, T. Fukawa, B. Li, M. Ito, Y. Shoji, T. Otani, L. Li, M. Kobayashi, M. Hachiya, Y. Tahara, D. Hashizume, T. Fukunaga, A. Fukazawa, Y. Li, H. Tsuji, K. Tamao, *Bull. Chem. Soc. Jpn.* **2011**, *84*, 1178–1191.

[15] M. Tsimerman, D. Mallik, T. Matsuo, T. Otani, K. Tamao, M. G. Organ, *Chem. Commun.* **2012**, *48*, 10352–10354.

[16] J. Bresien, A. Hinz, A. Schulz, T. Suhrbier, M. Thomas, A. Villinger, *Chem. Eur. J.* **2017**, *23*, 14738–14742.

[17] *Gaussian 09, Revision E.01*, M. J. Frisch, G. W. Trucks, H. B. Schlegel, G. E. Scuseria, M. A. Robb, J. R. Cheeseman, G. Scalmani, V. Barone, B. Mennucci, G. A. Petersson, H. Nakatsuji, M. Caricato, X. Li, H. P. Hratchian, A. F. Izmaylov, J. Bloino, G. Zheng, J. L. Sonnenberg, M. Hada, M. Ehara, K. Toyota, R. Fukuda, J. Hasegawa, M. Ishida, T. Nakajima, Y. Honda, O. Kitao, H. Nakai, T. Vreven, J. A. Montgomery Jr., J. E. Peralta, F. Ogliaro, M. Bearpark, J. J. Heyd, E. Brothers, K. N. Kudin, V. N. Staroverov, T. Keith, R. Kobayashi, J. Normand, K. Raghavachari, A. Rendell, J. C.

Burant, S. S. Iyengar, J. Tomasi, M. Cossi, N. Rega, J. M. Millam, M. Klene, J. E. Knox, J. B. Cross, V. Bakken, C. Adamo, J. Jaramillo, R. Gomperts, R. E. Stratmann, O. Yazyev, A. J. Austin, R. Cammi, C. Pomelli, J. W. Ochterski, R. L. Martin, K. Morokuma, V. G. Zakrzewski, G. A. Voth, P. Salvador, J. J. Dannenberg, S. Dapprich, A. D. Daniels, O. Farkas, J. B. Foresman, J. V. Ortiz, J. Cioslowski, D. J. Fox, Gaussian, Inc., Wallingford CT, **2013**.

[18] F. Neese, *Wiley Interdiscip. Rev.: Comput. Mol. Sci.* **2018**, *8*, e1327.

[19] F. Neese, *Wiley Interdiscip. Rev.: Comput. Mol. Sci.* **2022**, *12*, e1606.

[20] E. D. Glendening, J. K. Badenhoop, A. E. Reed, J. E. Carpenter, J. A. Bohmann, C. M. Morales, C. R. Landis, F. Weinhold, *NBO 6.0*, Theoretical Chemistry Institute, University of Wisconsin, Madison, **2013**.

[21] J. E. Carpenter, F. Weinhold, *J. Mol. Struct.: THEOCHEM* **1988**, *169*, 41–62.

[22] F. Weinhold, J. E. Carpenter, in *The Structure of Small Molecules and Ions* (Eds.: R. Naaman, Z. Vager), Springer, Boston, MA, **1988**, pp. 227–236.

[23] F. Weinhold, C. R. Landis, *Valency and Bonding. A Natural Bond Orbital Donor-Acceptor Perspective*, Cambridge University Press, **2005**.

[24] J. P. Perdew, K. Burke, M. Ernzerhof, *Phys. Rev. Lett.* **1996**, *77*, 3865–3868.

[25] J. P. Perdew, K. Burke, M. Ernzerhof, *Phys. Rev. Lett.* **1997**, *78*, 1396–1396.

[26] C. Adamo, V. Barone, *J. Chem. Phys.* **1999**, *110*, 6158–6170.

[27] S. Grimme, J. Antony, S. Ehrlich, H. Krieg, *J. Chem. Phys.* **2010**, *132*, 154104.

[28] S. Grimme, S. Ehrlich, L. Goerigk, *J. Comput. Chem.* **2011**, *32*, 1456–1465.

[29] F. Weigend, R. Ahlrichs, *Phys. Chem. Chem. Phys.* **2005**, *7*, 3297–305.

[30] F. Weigend, *Phys. Chem. Chem. Phys.* **2006**, *8*, 1057–1065.

[31] D. Hegarty, M. A. Robb, *Mol. Phys.* **1979**, *38*, 1795–1812.

[32] R. H. A. Eade, M. A. Robb, *Chem. Phys. Lett.* **1981**, *83*, 362–368.

[33] H. B. Schlegel, M. A. Robb, *Chem. Phys. Lett.* **1982**, *93*, 43–46.

[34] P. E. M. Siegbahn, *Chem. Phys. Lett.* **1984**, *109*, 417–423.

[35] F. Bernardi, A. Bottoni, J. J. W. McDouall, M. A. Robb, H. B. Schlegel, *Faraday Symp. Chem. Soc.* **1984**, *19*, 137–147.

[36] M. A. Robb, U. Niazi, *Rep. Mol. Theory* **1990**, *1*, 23–55.

[37] M. Frisch, I. N. Ragazos, M. A. Robb, H. Bernhard Schlegel, *Chem. Phys. Lett.* **1992**, *189*, 524–528.

[38] N. Yamamoto, T. Vreven, M. A. Robb, M. J. Frisch, H. Bernhard Schlegel, *Chem. Phys. Lett.* **1996**, *250*, 373–378.

[39] M. Klene, M. A. Robb, M. J. Frisch, P. Celani, *J. Chem. Phys.* **2000**, *113*, 5653–5665.

[40] C. J. Cramer, *Essentials of Computational Chemistry, Theories and Models*, John Wiley & Sons, Ltd, Chichester, UK, **2004**.

[41] J. Bresien, T. Kröger-Badge, S. Lochbrunner, D. Michalik, H. Müller, A. Schulz, E. Zander, *Chem. Sci.* **2019**, *10*, 3486–3493.

[42] F. London, *Journal de Physique et le Radium* **1937**, *8*, 397–409.

[43] R. McWeeny, *Physical Review* **1962**, *126*, 1028–1034.

[44] R. Ditchfield, *Mol. Phys.* **1974**, *27*, 789–807.

[45] K. Wolinski, J. F. Hinton, P. Pulay, *J. Am. Chem. Soc.* **1990**, *112*, 8251.

[46] J. R. Cheeseman, G. W. Trucks, T. a Keith, M. J. Frisch, *J. Chem. Phys.* **1996**, *104*, 5497–5509.

[47] C. J. Jameson, A. De Dios, A. Keith Jameson, *Chem. Phys. Lett.* **1990**, *167*, 575–582.

[48] C. van Wüllen, *Phys. Chem. Chem. Phys.* **2000**, *2*, 2137–2144.

[49] A. V. Marenich, C. J. Cramer, D. G. Truhlar, *J. Phys. Chem. B* **2009**, *113*, 6378–6396.

[50] C. Riplinger, F. Neese, *J. Chem. Phys.* **2013**, *138*, 034106.

[51] D. G. Liakos, M. Sparta, M. K. Kesharwani, J. M. L. Martin, F. Neese, *J. Chem. Theory Comput.* **2015**, *11*, 1525–1539.

[52] C. Riplinger, P. Pinski, U. Becker, E. F. Valeev, F. Neese, *J. Chem. Phys.* **2016**, *144*, 024109.

[53] A. Hellweg, C. Hättig, S. Höfener, W. Klopper, *Theoretical Chemistry Accounts* **2007**, *117*, 587–597.

[54] A. Schulz, *Z. Anorg. Allg. Chem.* **2014**, *640*, 2183–2192.

[55] L. Falivene, Z. Cao, A. Petta, L. Serra, A. Poater, R. Oliva, V. Scarano, L. Cavallo, *Nature Chemistry* **2019**, *11*, 872–879.

[56] W. Heisenberg, *Z. Phys.* **1928**, *49*, 619–636.

[57] P. A. M. Dirac, *Proc. R. Soc. London, Ser. A* **1926**, *112*, 661–677.

[58] I. N. Levine, *Quantum Chemistry Pearson Advanced Chemistry Series*, **2014**.

[59] H. Fliegl, S. Taubert, O. Lehtonen, D. Sundholm, *Phys. Chem. Chem. Phys.* **2011**, *13*, 20500–20518.

[60] D. Sundholm, H. Fliegl, R. J. F. Berger, *Wiley Interdiscip. Rev.: Comput. Mol. Sci.* **2016**, *6*, 639–678.

[61] J. Jusélius, D. Sundholm, J. Gauss, *J. Chem. Phys.* **2004**, *121*, 3952–3963.

[62] S. Taubert, D. Sundholm, J. Jusélius, *J. Chem. Phys.* **2011**, *134*, 054123.

[63] M. Rauhalahti, S. Taubert, D. Sundholm, V. Liégeois, *Phys. Chem. Chem. Phys.* **2017**, *19*, 7124–7131.

[64] J. Ahrens, B. Geveci, C. Law, *ParaView: An End-User Tool for Large Data Visualization*, Visualization Handbook, Elsevier, **2005**.

[65] Z. Chen, C. S. Wannere, C. Corminboeuf, R. Puchta, P. von Ragué Schleyer, *Chem.*

Rev. **2005**, *105*, 3842–3888.

[66] P. von R. Schleyer, C. Maerker, A. Dransfeld, H. Jiao, N. J. R. van E. Hommes, *J. Am. Chem. Soc.* **1996**, *118*, 6317–6318.

[67] H. Fallah-Bagher-Shaidaei, C. S. Wannere, C. Corminboeuf, R. Puchta, P. v. R. Schleyer, *Org. Lett.* **2006**, *8*, 863–866.
